# On-Demand Thio-Succinimide Hydrolysis for the Assembly of Stable Protein–Protein Conjugates

**DOI:** 10.1021/jacs.4c03721

**Published:** 2024-07-16

**Authors:** Aldrin V. Vasco, Ross J. Taylor, Yanira Méndez, Gonçalo J. L. Bernardes

**Affiliations:** Yusuf Hamied Department of Chemistry, University of Cambridge, CB2 1EW Cambridge, U.K.

## Abstract

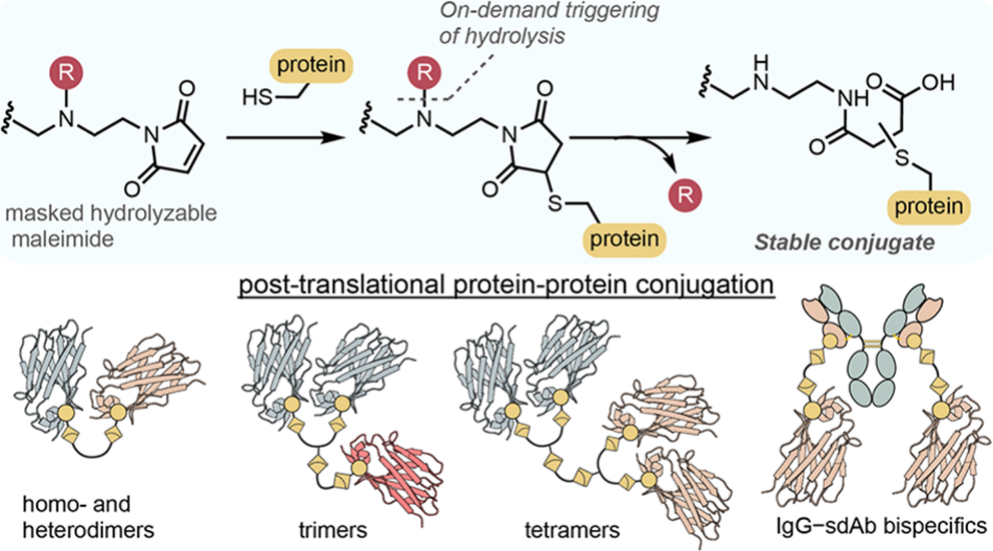

Chemical post-translational protein–protein conjugation is an important technique with growing applications in biotechnology and pharmaceutical research. Maleimides represent one of the most widely employed bioconjugation reagents. However, challenges associated with the instability of first- and second-generation maleimide technologies are yet to be fully addressed. We report the development of a novel class of maleimide reagents that can undergo on-demand ring-opening hydrolysis of the resulting thio-succinimide. This strategy enables rapid post-translational assembly of protein–protein conjugates. Thio-succinimide hydrolysis, triggered upon application of chemical, photochemical, or enzymatic stimuli, allowed homobifunctional bis-maleimide reagents to be applied in the production of stable protein–protein conjugates, with complete temporal control. Bivalent and bispecific protein–protein dimers constructed from small binders targeting antigens of oncological importance, PD-L1 and HER2, were generated with high purity, stability, and improved functionality compared to monomeric building blocks. The modularity of the approach was demonstrated through elaboration of the linker moiety through a bioorthogonal propargyl handle to produce protein–protein–fluorophore conjugates. Furthermore, extending the functionality of the homobifunctional reagents by temporarily masking reactive thiols included in the linker allowed the assembly of higher order trimeric and tetrameric single-domain antibody conjugates. The potential for the approach to be extended to proteins of greater biochemical complexity was demonstrated in the production of immunoglobulin single-domain antibody conjugates. On-demand control of thio-succinimide hydrolysis combined with the facile assembly of chemically defined homo- and heterodimers constitutes an important expansion of the chemical methods available for generating stable protein–protein conjugates.

## Introduction

Protein–protein conjugates stand as a unique class of biomolecules that combine two native proteins into one single scaffold, unlocking novel modes of actions with increasing impact in biotechnology and biopharmaceutical research and development.^1,2^ Applications include the generation of bifunctional engineered enzymes, antibody–enzyme conjugates, immunotoxins, immunocytokines, bispecific antibodies, and imaging, using fluorescent protein fusions.^3−10^ Traditionally, these protein–protein conjugates have been derived from the recombinant expression of fusion proteins.^1,11−13^ Although this represents an indispensable strategy, there remain several key drawbacks. These include the restrictive requirement for N-to-C terminal ligation, potential for incorrect protein folding, poor expression yields, and incompatibility of constituent protein expression systems, thus prohibiting coexpression.^7,11,13^

Post-translational protein–protein conjugation offers an alternative strategy in which constituent proteins are independently expressed prior to post-translational ligation. Expression followed by subsequent conjugation at preselected amino acid residues obviates the requirement for N-to-C terminal conjugates, allowing greater topological diversity to be explored.^14,15^ Furthermore, the ability to produce incompatible constituent proteins in separate expression hosts gives the potential to create protein–protein conjugates that are inaccessible in the form of a recombinantly expressed fusion protein.^7^ Examples of post-translational approaches include enzymatic and tag-based methods, the incorporation of noncanonical amino acids with bioorthogonal reactivity profiles, as well as heterobifunctional and homobifunctional chemical linking strategies.^16−19^ The latter represents a popular approach due to the inherent simplicity of linker synthesis and its application in the production of protein–protein conjugates.

**Figure 1:**
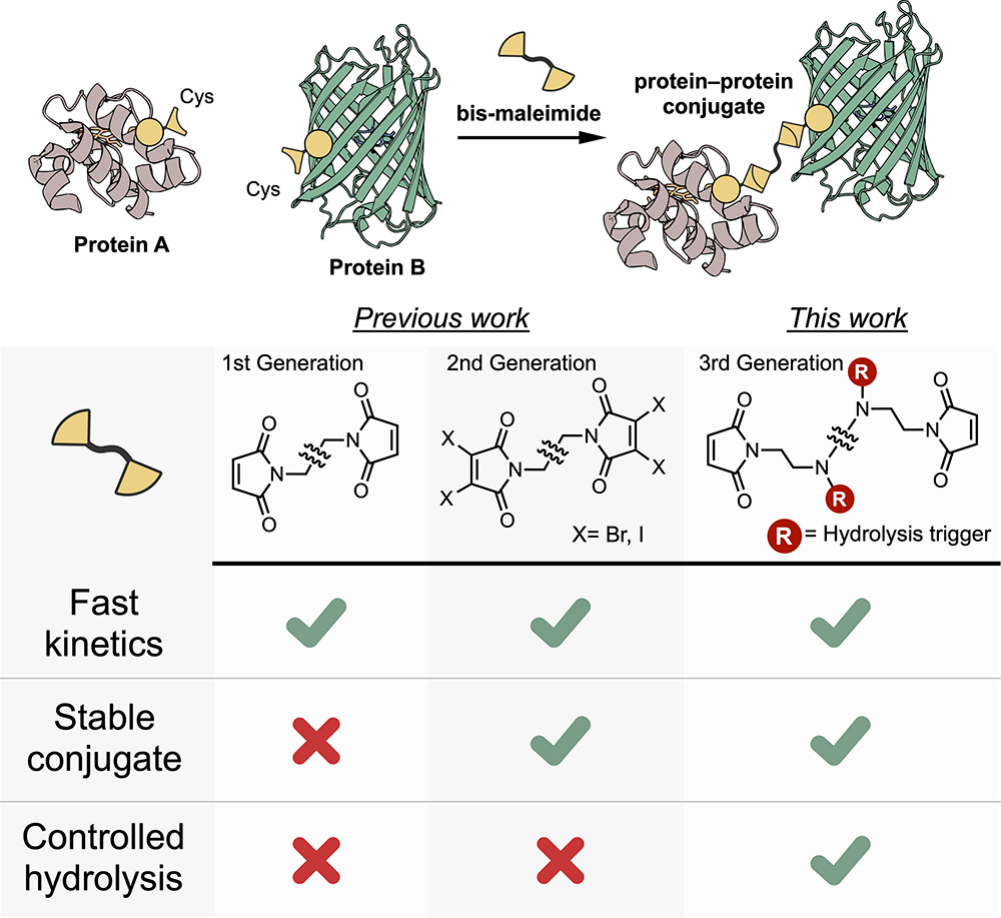
Overview of maleimide-based homobifunctional linker strategies in protein–protein conjugation.

Cysteine residues represent one of the most frequently targeted canonical amino acids in site-selective bioconjugation.^20−22^ This popularity can be attributed to the low abundance of cysteine residues in the proteome (<2%),^23^ further limited by many being unavailable for conjugation due to being involved in disulfide bonding,^24^ coupled with the inherent nucleophilicity of the thiolate group. The site-selective Michael addition reaction of cysteine residues with maleimide reagents remains the most reliable reaction when producing protein–small molecule conjugates.^25^ Naturally, the popularity of the cysteine–maleimide reaction holds true when considering homobifunctional reagents in the context of chemically mediated protein–protein conjugation, in the form of bis-maleimide reagents (Figure 1).^26−29^ The high second order rate constants (*k*_2_ = 10^2^ – 10^4^ M^–1^ s^–1^), relative to other common cysteine modifying reagents, helps to overcome the protein–protein coupling problem.^30−32^ In brief, the protein–protein coupling problem relates to the challenge associated with ligating two sterically encumbered coupling partners at low concentrations typically associated with reactions involving biomolecules (usually below 100 μM).^19^ Biorthogonal reactions such as copper-catalyzed azide–alkyne cycloaddition (CuAAC) and inverse electron-demand Diels–Alder reaction (IEDDA) have successfully been utilized in the preparation of protein–protein conjugates due to their favorable reaction rates.^19^ However, the requirement for installation of biorthogonal handles onto protein monomers adds additional steps, making these approaches more cumbersome and less attractive than direct conjugation through cysteine residues via a homobifunctional linking strategy.

Although maleimides offer an attractive reactivity profile, their utility comes with an important caveat. The retro-Michael deconjugation of maleimides and subsequent trapping by endogenous thiols leads to degradation of the resulting conjugate, and first generation maleimides do not represent a suitable approach for producing stable protein–protein conjugates (Figure 1).^25^ Various cysteine-based protein–protein conjugation technologies have been devised to overcome this issue, although mostly at the cost of slower kinetics compared to maleimide–cysteine conjugation. These include cysteine alkynylation using bis-5-(alkynyl)dibenzothiophenium triflate reagents,^33^ cysteine arylation using homobifunctional perfluoroaromatic reagents,^34^ and palladium-mediated protein–protein cross coupling.^35^ It is also known that ring-opening hydrolysis of the thio-succinimide prevents retro-Michael deconjugation, leading to a stable bioconjugate.^36^ In this vein, several “self-stabilizing” or “next generation maleimides,” which undergo uncontrolled ring-opening hydrolysis, have been developed (Figure 1).^37−42^

Self-stabilizing maleimides present an opportunity for overcoming the protein–protein coupling problem while generating stable conjugates. There are three fundamental characteristics a bis-maleimide reagent must possess to be useful in the production of stable protein–protein conjugates (Figure 1): (i) The maleimide–thiol reaction must have kinetics in line with first generation maleimides. (ii) Ring-opening hydrolysis of the thio-succinimide must occur within a reasonable time frame (typically minutes to hours) to form a stable conjugate. (iii) Premature ring-opening hydrolysis of the unreacted maleimide to unreactive maleamic acid must be minimal and occur in a controlled fashion.

To the best of our knowledge, the only previous attempts to produce protein–protein conjugates from self-stabilizing bis-maleimides have involved dihalomaleimides-based homobifunctional reagents (Figure 1).^43,44^ These reagents meet the first criterion of maintaining high reactivity with cysteine residues. However, the third criterion is not fulfilled and the second criterion only partially met, as the resulting thio-maleimide conjugates do not undergo sufficiently rapid hydrolysis, taking up to 72 h at 37 °C for complete hydrolytic stabilization.^44^ Additionally, dihalomaleimides have been shown to hydrolyze in an uncontrolled manner into unreactive maleamic acids more rapidly than standard maleimides, prior to conjugation.^44^ These factors render dihalomaleimides unsuitable for the production of protein–protein conjugates with complete control of conjugation and hydrolysis. It is therefore clear that an alternative strategy that meets all three criteria set out is necessary to fulfill the potential of next generation maleimides in controlled protein–protein conjugation.

In this work, we report on-demand stabilized maleimides which allow triggered hydrolysis of the resulting thio-succinimide conjugates (Figure 1). By temporarily masking a self-stabilizing maleimide in a less hydrolytically susceptible form, complex protein–protein conjugates could be built without the competing presence of rapid maleimide hydrolysis, fulfilling the first and third criteria. Upon application of an external trigger, the resulting conjugates could be fully stabilized by rapid ring-opening hydrolysis of the thio-succinimide, thus fulfilling the second criterion.

A homobifunctional linking strategy was developed that meets the essential criteria for an ideal maleimide reagent in the context of protein–protein conjugation. This approach leverages the advantages of favorable cysteine–maleimide reaction kinetics while addressing the instability of resulting conjugates by incorporating a rapid on-demand hydrolysis mechanism. The homobifunctional linking strategy described allowed the production of stable dimeric, trimeric, and tetrameric protein–protein conjugates with complete control. The scope of the present homobifunctional linking strategy was demonstrated in the production of functional protein–protein conjugates targeting a variety of cancer-related antigens. Binder formats ranged from small affibody (∼7 kDa) and single-domain antibody (sdAb) (∼15 kDa) binders to full immunoglobulins (∼150 kDa), demonstrating the modularity and versatility of the on-demand stabilization approach, which can be precisely tuned depending on the specific application.

## Results and Discussion

### Identification of an On-Demand Hydrolyzing Thio-Succinimide

Our investigations began by identifying a suitable self-stabilizing maleimide to temporarily mask, with the aim to trigger thio-succinimide hydrolysis upon application of an external stimulus. A promising candidate was identified in the form of a commercially available self-stabilizing maleimide derived from the nonproteinogenic amino acid, diamino propionic acid (Dap). The enhanced hydrolysis of thio-succinimide conjugates derived from *N*^α^–maleimido-Dap (mDap) was originally described to be promoted by a “base-catalyzed” mechanism.^41^ This hypothesis has since been challenged by Santi et al., who proposed that the inductive electron withdrawing effect of protonated amines proximal to the thio-succinimide was the dominant driving force for accelerated hydrolysis.^37^ Nonetheless, it remains undebated that the amino group of mDap is essential for enhanced hydrolysis rates.

**Figure 2:**
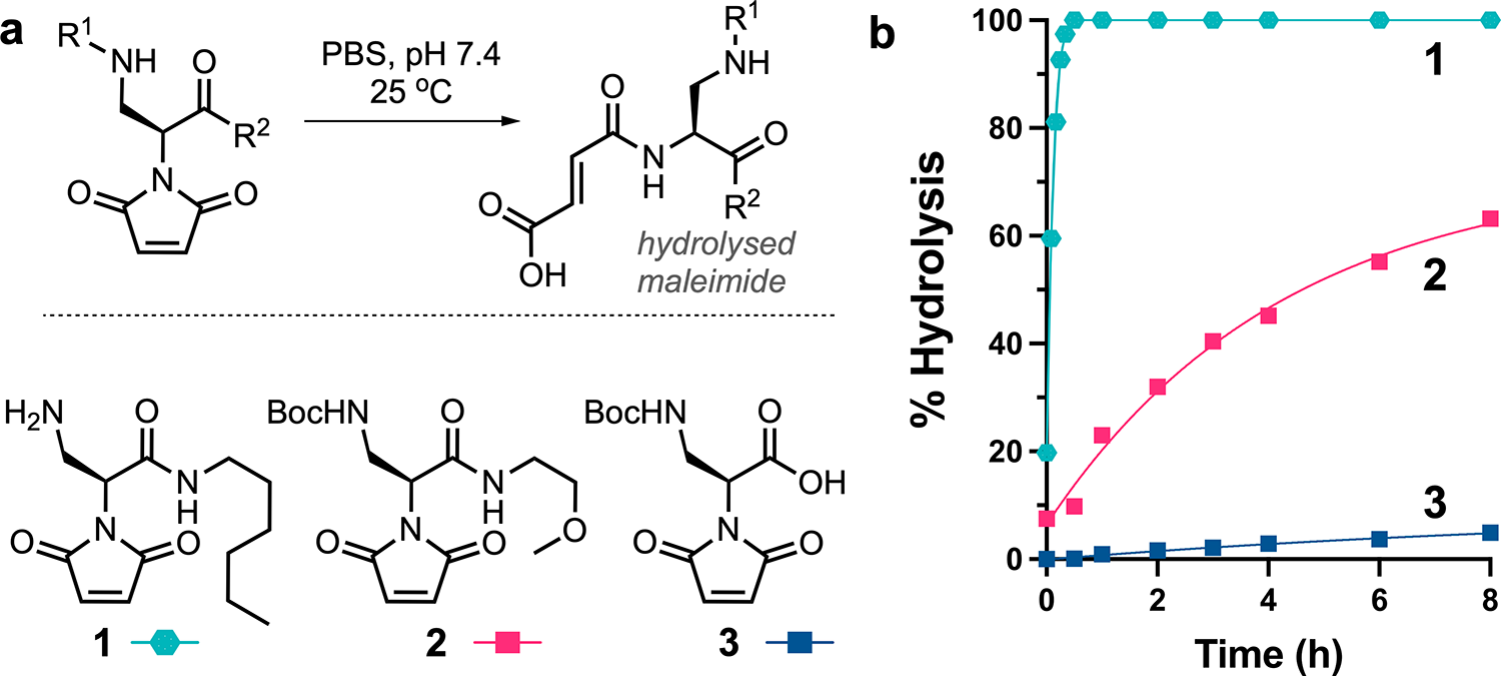
(a) General structure of Dap-based maleimides **1**-**3** and (b) kinetics of its hydrolysis as detected by HPLC analysis (single measurement).

The inductive electron withdrawing effect of a substituent can be described by the Taft σ* polar substituent constant, with larger positive values indicating a greater electron withdrawing capacity, and larger negative values indicating a greater electron donating effect.^45^ Based on the work of Santi et al., controlling the σ* value of the maleimide *N*-substituent gives quantitative control over the rate of maleimide/thio-succinimide hydrolysis. With this in mind, we studied the effect of protecting the Dap-amino group on maleimide hydrolysis in the form of a less electron withdrawing carbamate, with σ* values of 2.24 and 0.71, respectively. Maleimides **1** and **2** derived from Dap were synthesized and the hydrolysis rates, at pH 7.0 (NaP_i_ 20 mM) and 25 °C, were studied by HPLC, together with commercially available maleimide **3** (Figure 2a).

The hydrolysis of unprotected maleimide **1** had gone to completion in around 0.5 h (Figure 2b), in line with previous studies on thio-succinimide hydrolysis rates,^37,41^ making it unsuitable for bis-maleimide conjugation strategies, as the third criterion was unfulfilled. Under identical conditions, *N*^γ^-*Boc*-mDap (maleimide **2**) underwent markedly slower hydrolysis, with around 60% hydrolysis after 8 h (Figure 2b). Notably, the amide group of maleimide **2** was found to have a significant effect on the hydrolysis rate. The equivalent carboxylic acid analogue (maleimide **3**) reached slightly less than 10% hydrolysis after 8 h (Figure 2b). This difference was attributed to the positive σ* value of 1.68 for the amide, indicating an electron withdrawing, hydrolysis promoting effect.^37,45^ On the other hand, the carboxylate anion has a negative σ* value of −1.06, indicating a hydrolysis inhibiting electron donating effect, that leads to improved maleimide stability.^37,45^ These results further strengthened the argument for the inductive electron withdrawing effect being the key driver of maleimide hydrolysis rates.

**Figure 3:**
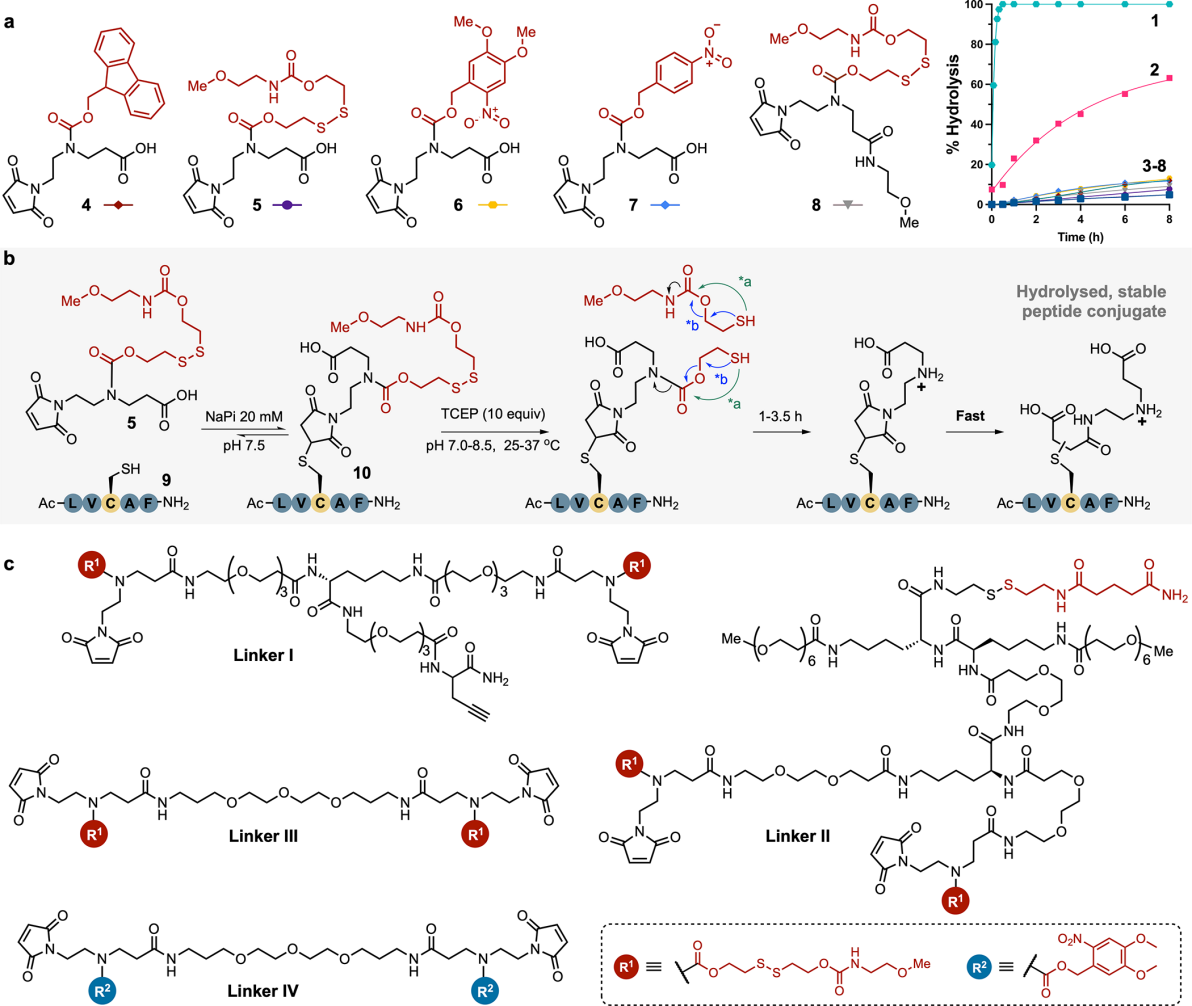
(a) Maleimides **4**–**8** bearing diverse *N*-protections and their hydrolysis kinetics—as determined by HPLC–MS analysis (single measurement)—compared to **1**-**3**. Data for maleimides **1**-**3** has been replotted from Figure 2b for comparative purposes. When compared to the corresponding protected maleimides, the hydrolysis rate of the thio-succinimide derivatives was found to be negligible prior to triggered unmasking (Figure S18). (b) Mechanism for reductively triggered immolation, via either *a or *b,^46−48^ and subsequent ring-opening hydrolysis of peptido-thio-succinimide **9** derived from reaction of Ac-LVCAF-NH_2_ with **5**. (c) Homobifunctional linkers synthesized from on-demand stabilizing maleimides **5** and **6**.

Due to the amide group being a hydrolysis promoting substituent, it became clear that removing its effect was key to minimizing preconjugation hydrolysis. To address this issue, we synthesized an alternative maleimide derivative with a carbamate protected secondary amine, two carbons away from the maleimide nitrogen (maleimide **4**) (Figure 3a). Under identical conditions, maleimide **4** underwent less than 10% hydrolysis after 8 h (Figure 3a). The reduced hydrolysis rate of maleimide **4** was deemed to provide a sufficiently long window for controlled dimerization of proteins using maleimide–thiol conjugation, typically achieved on the minutes to hours time scale.

With a sufficiently stable maleimide in hand, we hypothesized that derivatives of maleimide **4** could be temporarily masked using labile protecting groups. Using bioconjugation-compatible triggers, it was envisioned that the carbamate could subsequently be removed, unveiling an electron withdrawing secondary amine group. To explore this, maleimides **5**, **6**, and **7**, with chemical, photochemical, and enzymatic deprotection triggers, respectively, were synthesized (Figure 3a).

Disulfide reduction is a common approach used for unmasking the activity of cytotoxic payloads in the field of antibody drug conjugates (ADCs) via self-immolative or thiol release pathways.^46−50^ Taking inspiration from the field of ADC development, we envisioned a bioconjugation-compatible chemical protecting group that could be unmasked upon disulfide reduction and subsequent self-immolative release of a hydrolysis promoting secondary amine. To achieve timely amine unmasking and hydrolysis of the thio-succinimide, efficient disulfide reduction was essential. Commonly employed disulfide-based cysteine protecting groups in peptide chemistry, such as S*t*Bu, SIT, and SiPr, are sterically hindered and demand extended deprotection times with some requiring forcing conditions, making them suboptimal in the context of unmasking thiols presented on a protein substrate.^50,51^ Due to exhibiting efficient reduction rates, an unhindered primary disulfide-based protecting group was deemed most suitable for this application.^49,50^ With this in mind, we incorporated a bis-2-mercaptoethyl carbamate disulfide, which not only undergoes fast reduction but also leads to nonreactive species upon intramolecular thiol cyclization.^46−48^ In the case of maleimide **6**, the 4,5-dimethoxy-2-nitrobenzyl carbamate-protecting group was the chosen for light triggered unmasking,^52^ while 4-nitrobenzyl carbamate, a nitroreductase substrate, was incorporated as an enzymatic trigger in maleimide **7**.^53^

All three carbamate groups were found to endow the maleimide with stability consistent with **4**, under identical conditions (pH 7.0, NaP_i_, 20 mM, 25 °C) (Figure 3a). The transformation of **5** into an amide analog gave rise to maleimide **8**. Importantly, this confirmed that the electron withdrawing amide was sufficiently distant from the maleimide group to have a negligible effect on the rate of hydrolysis when compared to maleimides **5**–**7**.

Having established suitable levels of stabilization using bioconjugation-compatible protecting groups, the potential for on-demand hydrolysis of thio-succinimides upon application of chemical, photochemical, or enzymatic stimuli was investigated. Maleimides **5**–**7** were conjugated to a cysteine-containing pentapeptide (**9**) (Ac-LVCAF-NH_2_) and the rate of amine deprotection and thio-succinimide hydrolysis was assessed by HPLC–MS (Figures S20–S22). The peptido-thio-succinimide (**10**) derived from maleimide **5** contained a disulfide bond which upon application of tris(2-carboxyethyl)phosphine (TCEP) (10 equivalents) self-immolative amine deprotection via thiolate cyclization was initiated (Figure 3b). Slightly elevated pH (pH 8.0) and temperature (37 °C) were found to be optimal, with complete deprotection and accompanying maleimide hydrolysis occurring within 3.5 h (Figures 3b and S20). Both photo- and enzymatic triggers also led to efficient removal of carbamate-protecting groups and rapid hydrolysis of thio-succinimides derived from maleimides **6** and **7**, respectively (Figures S21–S22).

### Development of a Homobifunctional Linking Strategy

Considering the simplicity of adding TCEP as a trigger for stabilization of thio-succinimides, we deemed it the most accessible approach for a general audience. Initially, a homobifunctional bis-maleimide reagent (**Linker I**), derived from maleimide **5**, was synthesized using solid phase procedures (Figure 3c). The linker moiety of **Linker I** consisted of a branched poly(ethylene glycol) (PEG) to enhance solubility and achieve flexibility, in line with what may be achieved with a GS linker in fusion proteins. The addition of a bioorthogonal propargyl glycine introduced the potential for further elaboration of protein–protein conjugates via CuAAC at a later stage. This linker modularity further highlighted the adaptability of the chemical conjugation approach when compared to recombinant expression of fusion proteins, which are restricted to canonical amino acid linkers.

With **Linker I** in hand, we selected sdAbs targeting tumor-associated antigens, programmed death ligand 1 (PD-L1)^54^ and human epidermal growth factor receptor 2 (HER2; 2Rb17c),^55^ as interesting model proteins for developing homo- and heterodimerization protocols from reaction with a free cysteine residue inserted at the C-terminal region. Due to their small size compared to immunoglobulins, sdAbs represent an interesting class of biologics which can access more cryptic epitopes of tumor antigens, coupled with better penetration of solid tumors.^56,57^ However, the small size of sdAbs comes with associated drawbacks including rapid clearance rates and, as monovalent binders, sdAbs do not display the avidity effect characteristic of immunoglobulins.^57^ Dimerization of sdAbs can introduce the avidity effect in bivalent homodimers, as well as increased tumor specificity, in the case of bispecific heterodimers. These effects can be achieved while maintaining superior tumor penetration compared to immunoglobulin formats, due to maintaining the relatively small size.

**Figure 4:**
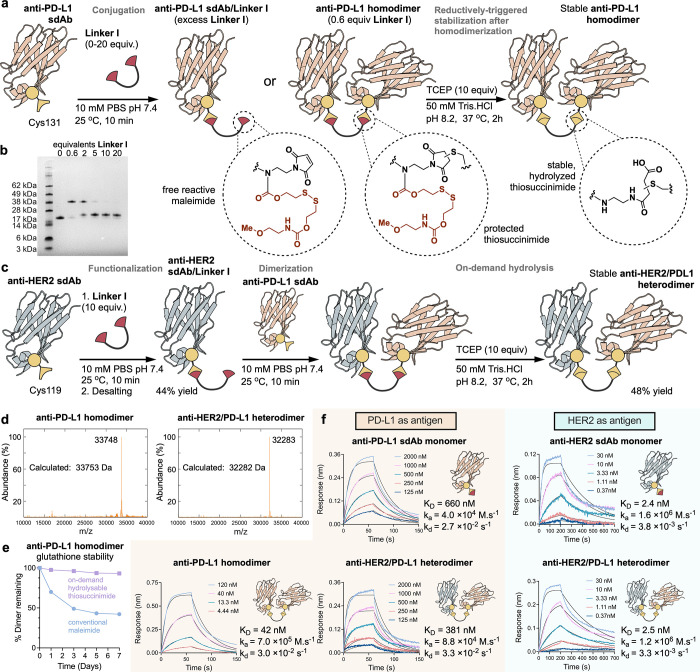
(a) General strategy for the synthesis of an **anti-PD-L1 homodimer** from a model **anti-PD-L1 sdAb** in the presence of **Linker I** and (b) SDS-Page for the modification of the model sdAb with different equivalents of **Linker I**. (**c**) Strategy for the assembly of an **anti-HER2/PD-L1 heterodimer** from **Linker I**. (d) Deconvoluted MS spectra of pure **anti-PD-L1 homodimer** and **anti-HER2/PD-L1 heterodimer**. (e) Glutathione stability for stabilized (on-demand hydrolyzed) **anti-PD-L1 homodimer** as compared with a conventional Peg-maleimide control. Dimers were incubated in PBS (pH 7.4) including reduced glutathione (2 mM) for 7 days at 37 °C. Percentage of the remaining dimer was evaluated over time using SDS-Page gel and coomassie staining. (f) BLI binding assays for homo- and heterodimers, and the corresponding monomeric sdAbs.

The first step was to determine the optimal conditions for homo- and heterodimerization. When developing a successful one-pot homobifunctional linking reaction, near-stoichiometric conjugation between **Linker I** and sdAb to **anti-PD-L1 homodimer** is essential (Figure 4a). However, in the case of heterodimerization, the first protein must efficiently undergo complete modification. That is, the sdAb monomer must be completely converted to an sdAb–**Linker I** intermediate bearing a free unreacted maleimide (**anti-PD-L1 sdAb/Linker I**) functionality for reaction with a second protein, without unwanted homodimerization occurring (Figure 4a). Upon reacting the **anti-PD-L1 sdAb** with increasing molar equivalents of **Linker I**, it was found that addition of 0.6 equivalents was optimal for achieving efficient conversion to the **anti-PD-L1 homodimer**, while 10 equivalents was sufficient for achieving complete conversion to **anti-PD-L1 sdAb/Linker I**, an essential intermediate in the production of heterodimers, as determined by SDS-PAGE (Figures 4b and S23). Complete stabilization via TCEP-triggered amine unmasking and accompanying thio-succinimide hydrolysis of the **anti-PD-L1 homodimer** occurred within 2 h (10 equivalents, Tris.HCl 50 mM, pH 8.2, 37 °C), with amine deprotection via thiolate cyclization as the rate-limiting step of the immolation (Figures 3b and 4a).

Having identified suitable conditions, the **anti-PD-L1 homodimer** was produced and isolated in 46% yield after purification by size exclusion chromatography (SEC). Identity stability of the resulting homodimer was confirmed by LC-MS, nanoDSF, and CD, respectively, and found to be consistent with the constituent monomers (Figures 4d, S25 and S27).

A key consideration was to determine the stability of the **anti-PD-L1 homodimer** derived from **Linker I** compared to a commercial PEG bis-maleimide reagent with respect to retro-Michael deconjugation and thiol exchange. When incubated in PBS (pH 7.4) including reduced glutathione (2 mM) at 37 °C for 7 days, negligible decomposition of the **Linker I** derived conjugate was observed (Figures 4e and S35). The commercial PEG bis-maleimide conjugate underwent significant deconjugation under identical conditions with <50% of the dimer remaining after 7 days (Figure S35). The stability of the linking strategy in 50% human plasma was also explored through a stable **EGFP homodimer** which remained intact over the course of 7 days at 37 °C (Figure S36). These experiments confirmed that as desired, ring-opening hydrolysis generated protein–protein conjugates with enhanced stability.

Heterodimerization of **anti-HER2 sdAb** and **anti-PD-L1 sdAb** was subsequently achieved by using a stepwise conjugation approach (Figure 4c). Upon addition of 10 equivalents of **Linker I**, **anti-HER2 sdAb/Linker I** was isolated in 44% yield after SEC. To this, **anti-PD-L1 sdAb** (1.2 equivalents) was added to generate the **anti-HER2/PD-L1 heterodimer**. The resulting conjugate was stabilized with a TCEP trigger and isolated in 48% yield (overall yield: 21%) after SEC. Identity and thermal stability were confirmed by LC–MS and nanoDSF, respectively (Figures 4d and S28).

With both the **anti-PD-L1 homodimer** and the **anti-HER2/PD-L1 heterodimer** in hand, we looked to assess the functional effect of dimerization on these binders. Using biolayer interferometry (BLI), on rate (*k*_a_), off rate (*k*_d_), and equilibrium constant (*K*_D_) values for binding the target antigens were measured and compared to parental sdAb monomers. Interestingly, the bivalent **anti-PD-L1 homodimer** showed an order of magnitude improvement in *k*_a_, while maintaining a consistent *k*_d_ relative to its monomer, leading to an overall improvement in *K*_D_ (Figure 4f). Typically avidity mediated slowing of *k*_d_ would be expected in bivalent homodimers, suggesting the mode of improved overall *K*_D_ in the **anti-PD-L1 homodimer** was via an alternative *k*_a_-driven improvement in affinity.^58^ In the case of the **anti-HER2/PD-L1 heterodimer**, the bispecific binder was found to engage with both target antigens with kinetic parameters consistent with the constituent sdAb monomers (Figure 4f). Furthermore, the simultaneous engagement of both target antigens was confirmed using a dual engagement BLI assay (Figure S40), which is an important feature of many bispecific antibodies. These binding assays confirmed protein–protein conjugation with **Linker I** generated dimers with functionality greater than their constituent parts, the central aim when generating protein–protein conjugates via post-translational or recombinant means.

### Avidity Mediated Affinity Maturation of HER2 Binders

**Figure 5:**
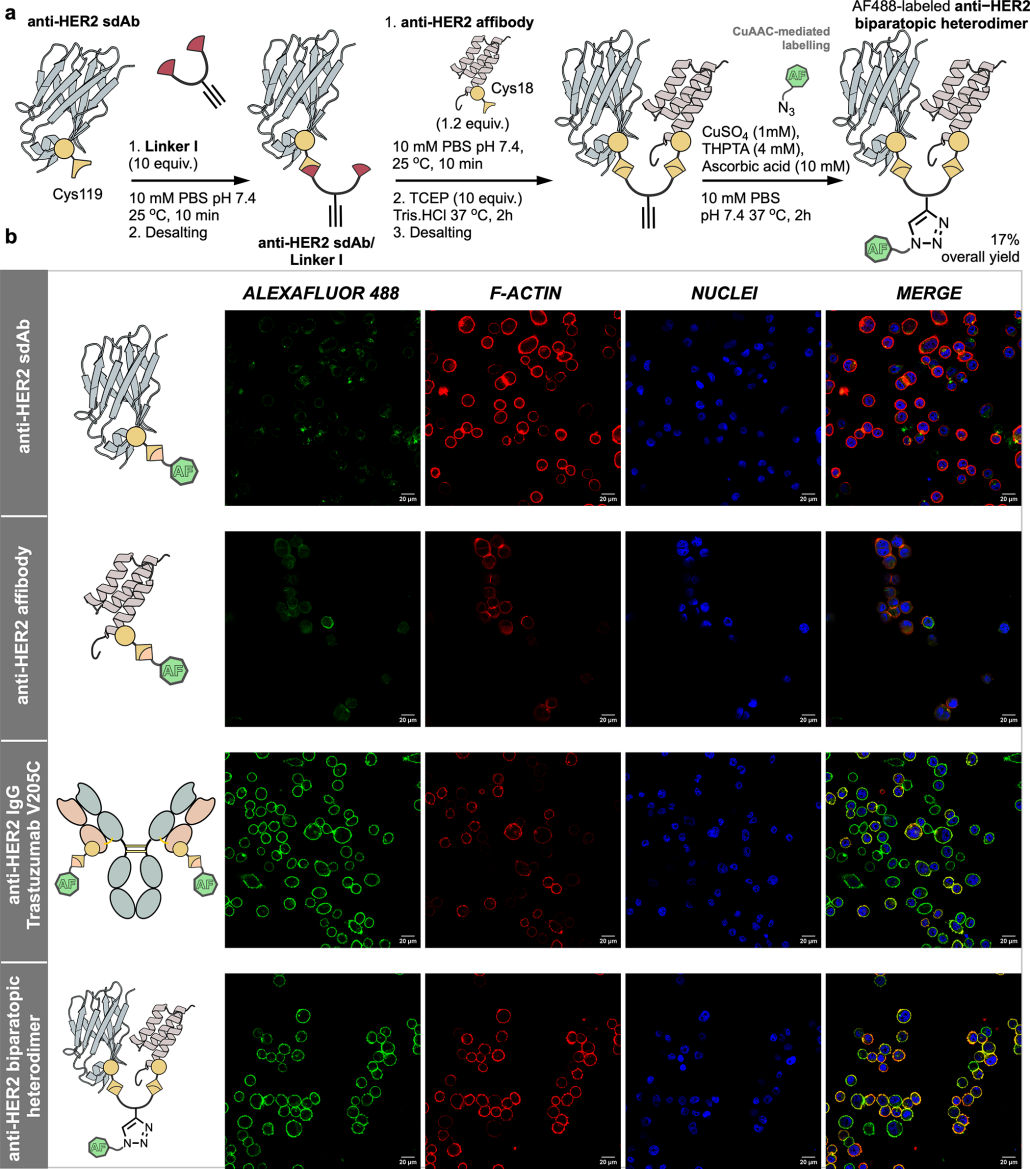
(a) Synthetic route to AF488-labaled **anti-HER2 biparatopic heterodimer** from the CuAAC click reaction post protein ligation and stabilization enabled by the propargyl group in **Linker I**. (b) Confocal microscopy imaging of HER2+ SK-BR-3 cells upon incubation with AF488-labeled **anti-HER2 biparatopic heterodimer**, its monomeric sdAb and affibody components, and the anti-HER2 IgG trastuzumab. The images were recorded at 100 nM antibody concentration after 4 h incubation with 6 × 10^4^ SK-BR-3 cells, cell fixation and F-actin and nuclei labeling on a Leica DMi8 confocal microscope with a 40× objective. For comparison purposes, the fluorescence intensity was normalized to the effective concentration of AF488.

Having proven the suitability of the homobifunctional linking strategy for the assembly of homo- and heterodimers, we aimed to assess the functional effect of dimerization on a pair of binders specific to HER2. By using **Linker I** in combination with the **anti-HER2 sdAb**(55) and an **anti-HER2 affibody** (R18C ZHER2)^59^—which bind to distinct epitopes of the extracellular domain of HER2—three possible combinations were generated, i.e., two homodimers and one heterodimer. Using conditions identified to produce **anti-PD-L1 homodimer**, the **anti-HER2 sdAb homodimer** and **anti-HER2 affibody homodimer** were isolated with high purity and stability in 43 and 57% yields after SEC, respectively (Figures S26–S27). The **anti-HER2 biparatopic heterodimer** was generated from the **anti-HER2 sdAb** and **anti-HER2 affibody** in a two-step approach analogous to that for the **anti-HER2/PD-L1 heterodimer** (Figure 5a), with an overall isolated yield of 17% after SEC.

Initially, the kinetic binding parameters of all monomers and dimers were assessed for binding to immobilized HER2 by BLI. All dimers showed an enhanced *k*_d_ compared to their parental monomers, an effect in line with what would be expected due to avidity-mediated affinity maturation (Figure S39).^58^ Both the **anti-HER2 sdAb homodimer** and the **anti-HER2 biparatopic heterodimer** displayed minimal perturbation of their *k*_a_ values, leading to an overall off-rate-driven improvement in apparent *K*_D_s. Interestingly, homodimerization of the **anti-HER2 affibody** was detrimental to the *k*_a_, causing a 1 order of magnitude decrease, when compared to the monomer. Due to the enhanced *k*_d_, however, the overall apparent *K*_D_ did not change. Following from these observations, how the in vitro binding kinetics of dimers translated to binding to cell-surface HER2 was of great interest. To enable this, the dimers were functionalized with azido-Alexa Fluor 488 (AF488) via CuAAC through the propargyl handle of **Linker I** (Figure 5a). This enabled qualitative assessment of the cell binding capability of HER2+ SK-BR-3 cells using confocal microscopy.

When SK-BR-3 cells were incubated in the presence of monomers labeled with AF488, minimal antibody remained bound to the cell surface after preimaging wash steps, consistent with the fast *k*_d_s observed in BLI experiments. When these binders were combined to generate **anti-HER2 biparatopic heterodimer**, strong membrane localization was observed (Figure 5b), in line with avidity-mediated *k*_d_ improvement. Similar results were observed for **Trastuzumab–AF488** and the **anti-HER2 sdAb homodimer** (Figure S41). Interestingly, the **anti-HER2 affibody homodimer** did not show any significant enhancement in membrane localization, indicating that the avidity-mediated *k*_d_ improvement observed in BLI did not translate to cell-surface-HER2 binding. The lack of correspondence between BLI and confocal microscopy experiments may be due to the significantly more complex environment associated with cell surface presented antigens when compared to pure protein used in BLI. Unfavorable interactions or steric clashes may occur with other cell-surface components in the case of the **anti-HER2 affibody homodimer**. The deleterious effect dimerization had on the *k*_a_ of the **anti-HER2 affibody homodimer** reinforces the importance of post-translational protein–protein conjugation approaches to rapidly explore potential combinations from a small panel of binders, giving rise to diverse functional outcomes. For all binders, the same experiment was completed using the MCF-7 cell line, expressing low levels of HER2, to which binding was negligible (Figure S42).

**Figure 6:**
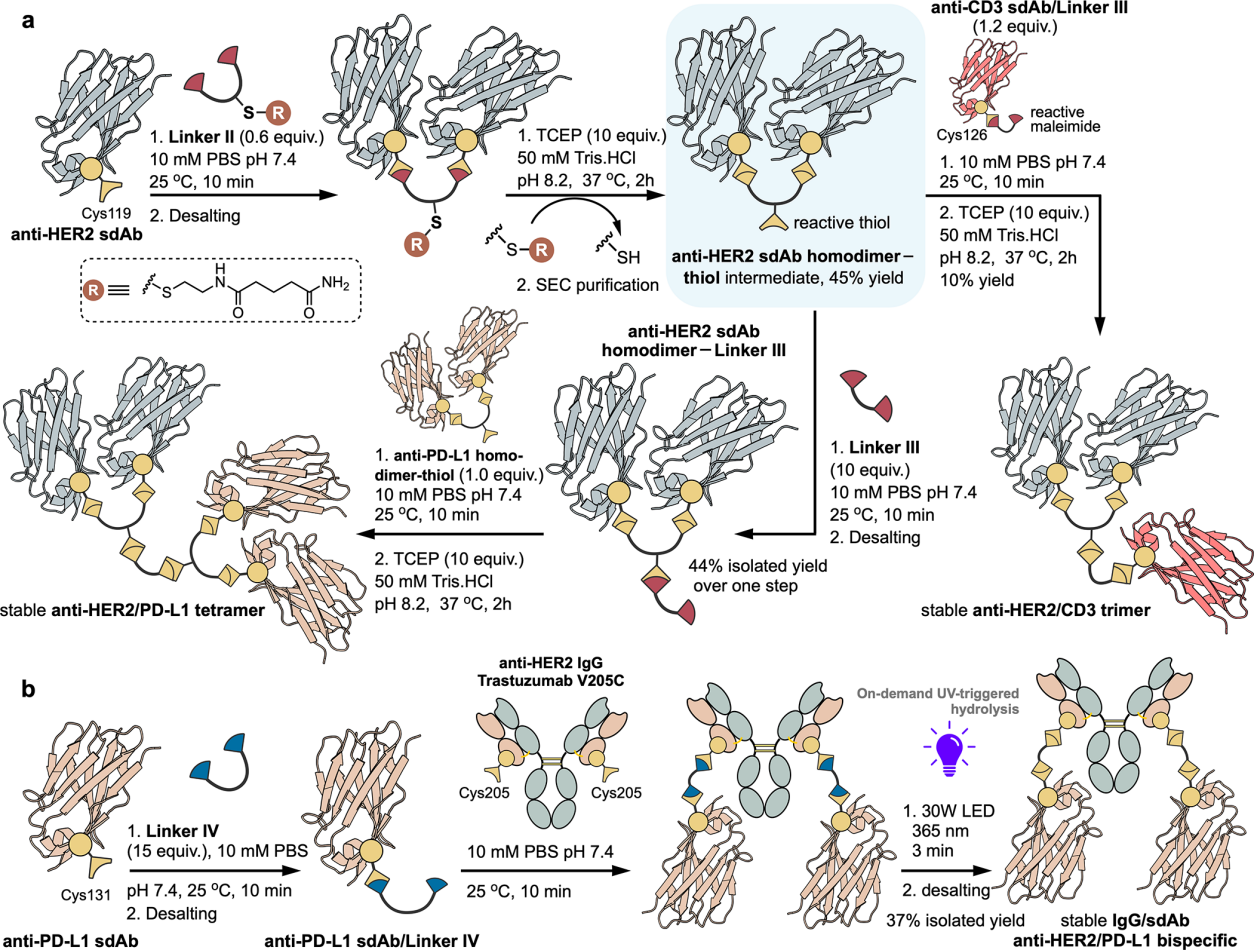
(a) Synthetic route to stable **anti-HER2/CD3 trimer** and **anti-HER2/PD-L1 tetramer** constructs from **anti-HER2 sdAb**, **anti-CD3 sdAb** and **anti-PD-L1 sdAb**. (b) Assembly of a **stable IgG/sdAb anti-HER2/PD-L1 bispecific** via the UV-stabilizing maleimide-based homobifunctional **Linker IV**.

The scope of the stabilized protein–protein conjugates was further explored in investigating the potential for generating trimeric and tetrameric constructs. The objective was to exploit the modularity of the approach for the convergent assembly of higher order constructs from dimeric building blocks. To achieve this, **Linker II** was designed to incorporate a masked thiol in the form of a disulfide (Figure 3c). Upon reductively triggered hydrolysis, the reactive thiol was unmasked (Figure 6a, top), thus enabling a second round of thiol-maleimide bioconjugation following the homobifunctional linking strategy. **Linker II** was functionalized with additional PEG-6 units to further improve the solubility of the larger molecule under aqueous conditions. Additionally, homobifunctional reagents **Linker III** and **Linker IV** were synthesized with reductive and UV-labile masking groups, respectively (Figure 3c). The intended use of **Linker III** and **Linker IV** was to act as short linkers between higher order protein–protein conjugates. For this reason, a linear PEG linker was used without the inclusion of additional functional handles.

The key intermediate **[anti-HER2 sdAb homodimer–thiol]** was prepared from the **anti-HER2 sdAb** using 0.6 equivalents**Linker II**, followed by TCEP-mediated hydrolysis and simultaneous thiol deprotection, for a 45% isolated yield after SEC purification. The reactive thiol in this intermediate could be employed to react with a free maleimide, analogous to the heterodimerization approach. An **anti-CD3 sdAb–Linker III** was obtained upon reaction of an anti-CD3 sdAb bearing a free Cys, inserted in the C-terminal region, with excess **Linker III**, and subsequently conjugated to the **anti-HER2 sdAb homodimer–thiol** intermediate after removal of excess linker (Figure 6a). Upon TCEP-mediated hydrolysis, the bispecific **anti-HER2/CD3 trimer** was isolated in a 10% yield after SEC purification.

The assembly of an **anti-HER2/PD-L1 tetramer** was also demonstrated. Reaction of the **anti-HER2 sdAb homodimer–thiol** intermediate with excess bis-maleimide **Linker III** readily afforded an **anti-HER2 sdAb homodimer–Linker III**, which was successfully isolated from the excess of **Linker III** using Pierce Strong Cation Exchange spin columns. Having the maleimide containing **anti-HER2 homodimer sdAb–Linker III** in hand, the conjugation to an **anti-PD-L1 homodimer–thiol** was attempted, generating the expected **anti-HER2/PD-L1 tetramer** (Figures 6a and S31).

The construction of trimers and tetramers from dimeric building blocks was designed to be modular and could also be extended to other chemistries beyond thiol-maleimide Michael additions. To explore this, a DBCO bearing homobifunctional maleimide reagent was prepared for use in strain-promoted azide alkyne cycloaddition (**Linker V**) (SI, Figure S32). This approach allowed an **anti-HER2 sdAb homodimer–DBCO** to be conjugated with an **azido-CD3 sdAb**. However, the DBCO-azide cycloaddition has the accompanying issue of a significantly poorer rate constant than thiol-maleimide cycloadditions and required 48 h incubation at 37 °C to achieve conversion to the desired **anti-HER2/CD3 trimer**. In this time, instability of the DBCO moiety toward hydrolysis became an important factor (Figure S33).

The main drawback arising from the use of reducing agents as triggers for thio-succinimide hydrolysis comes from its limited application in the modification of proteins with disulfides prone to reduction, e.g., IgGs. Reducing agent-based approaches are suboptimal in this context due to the requirement for disulfide bond reoxidation which adds additional processing as well as the potential of disulfide scrambling.^60^ In this scenario, a homobifunctional linker based on UV-stabilized maleimide **6** was envisioned as a suitable alternative (**Linker IV**) that obviates the requirement for reduction and reoxidation. The applicability was demonstrated through the assembly of a stable **IgG/sdAb anti-HER2/PD-L1 bispecific** construct (Figure 6b). In a similar way to the sdAb heterodimerization strategies described above, **anti-PD-L1 sdAb** was reacted with an excess of **Linker IV** to yield the **anti-PD-L1 sdAb/Linker IV** intermediate. Following this, the bispecific was assembled upon reaction with the **anti-HER2 IgG Trastuzumab V205C** with the subsequent UV-triggered ring-opening stabilization achieved within 3 min upon irradiation under a 30W LED lamp at 365 nm. The resulting **stable IgG/sdAb anti-HER2/PD-L1 bispecific** was purified by ultrafiltration/diafiltration with a 100 kDa MWCO filter and isolated in 37% yield with 64% homogeneity, as determined by SDS-PAGE.

## Conclusions

On-demand hydrolyzing maleimides offer a robust chemically driven post-translational protein–protein conjugation approach that both overcomes the protein–protein coupling problem while offering full control over maleimide and thio-succinimide stability. The intrinsic modularity of the bis-maleimide reagents enabled the generation of complex protein–protein binders ranging from homodimers to tetramers. Due to the mild, bioconjugation-compatible nature of the strategy, stable protein–protein dimers targeting a variety of cancer-related antigens were accessed. The approach yielded bivalent, biparatopic, and bispecific binders with enhanced functionality with respect to their monomeric subunits. Having demonstrated the modularity and wide-ranging applicability of the approach, we envisage further expansion of the technology for the identification of multispecific binders, rapid exploration of linkers of diverse physicochemical natures, and generation of stable antibody–drug conjugates.

## Supporting Information Available

Detailed methods, characterization data, and additional figures (PDF)
