# Supplementary material for: On-Demand Thio-Succinimide Hydrolysis for the Assembly of Stable Protein–Protein Conjugates
Source: J Am Chem Soc. 2024 Jul 16;146(30):20709–19. doi: 10.1021/jacs.4c03721 (PMC11295205; doi:10.1021/jacs.4c03721)
Supplement: Supplementary file 1 — ja4c03721_si_001.pdf [file ja4c03721_si_001.pdf]

Supporting Information

On-Demand Thio-Succinimide Hydrolysis for the Assembly of Stable Protein–Protein Conjugates

Aldrin V. Vasco,† Ross J. Taylor,† Yanira Méndez, and Gonçalo J. L. Bernardes*

Yusuf Hamied Department of Chemistry, University of Cambridge, Lensfield Road, CB2 1EW Cambridge, UK

†*These authors contributed equally to this work*

E-mail: gb453@cam.ac.uk

Contents

1 Experimental procedures **S-7**

1.1 General experimental procedures S-7

1.2 General LC–MS protocols S-8

1.3 Synthetic procedures S-10

1.4 Solid-phase peptide synthesis S-26

1.5 Maleimide hydrolysis studies S-33

1.6 Self-immolation and on-demand hydrolysis studies S-37

1.6.1 Reductively triggered immolation S-37

1.6.2 UV-decaging . . . S-39

1.6.3 Enzymatic-decaging . . . S-40

1.7 Expression and purification of proteins . . . S-42

1.7.1 Expression of Nanobodies . . . S-42

1.7.2 Expression of Affibody . . . S-44

1.8 Protein bioconjugation experiments . . . S-44

1.8.1 Optimising dimerisation conditions . . . S-44

1.8.2 Optimised method for the assembly and stabilisation of homodimeric protein–protein conjugates . . . S-45

1.8.3 Optimised method for the assembly of heterodimeric protein–protein conjugates . . . S-52

1.8.4 General method for cysteine capping with *N*-methyl maleimide . . . S-56

1.8.5 General method for cysteine modification with Alexa Fluor 488 maleimideS-56

1.8.6 Thiomab modification with Alexa Fluor 488 maleimide . . . S-57

1.8.7 General method for CuAAC labelling with Alexa Fluor 488 . . . S-57

1.8.8 Thiol-based trimerisation procedure . . . S-58

1.8.9 Thiol-based tetramerisation procedure . . . S-60

1.8.10 DBCO-based trimerisation procedure . . . S-63

1.8.11 Anti-PD-L1 sdAb–Thiomab conjugation . . . S-66

1.9 Stability assays . . . S-68

1.9.1 Glutathione stability assay . . . S-68

1.9.2 Human plasma stability assay . . . S-69

1.10 Biophysical characterisation . . . S-70

1.10.1 Circular dichroism . . . S-70

1.10.2 NanoDSF thermal denaturation assays . . . S-71

1.11 Binding assays . . . S-73

1.11.1 Biolayer Interferometry . . . S-73

1.11.2 Biolayer Interferometry dual engagement binding assay S-77
1.12 Cell imaging protocol S-79

2 Protein amino acid sequences S-82

3 LC–MS spectra S-84

3.1 LC–MS spectra of unmodified proteins S-84
3.2 Optimisation of homodimerisation and monomer-linker conjugation S-89
3.3 LC–MS spectra homodimeric protein–protein conjugates S-91
3.4 LC–MS analysis of heterodimers and their intermediates S-96
3.5 LC–MS analysis of *N*-methyl maleimide capped proteins for BLI S-99
3.6 LC–MS analysis of Alexa Fluor 488 maleimide labelled proteins S-102
3.7 LC–MS analysis of Alexa Fluor 488 labelled dimers S-105
3.8 Thiol-based trimerisation LC–MS S-108
3.9 Tetramerisation LC–MS S-111
3.10 DBCO-based trimerisation LC–MS S-113
3.11 LC–MS spectra of anti-PD-L1 nanobody–Thiomab conjugates S-116

4 SEC purification S-121

5 ^{1}H and ^{13}C NMR spectra S-122

References S-127

List of Figures

S1 Total Absorbance Chromatogram of pure maleimide **1** S-10
S2 Total Absorbance Chromatogram of pure maleimide **2** S-11
S3 Total Absorbance Chromatogram of pure maleimide **4** S-15
S4 Total Absorbance Chromatogram of pure maleimide **5** S-19

S5 Total Absorbance Chromatogram of pure maleimide **6** S-21
S6 Total Absorbance Chromatogram of pure maleimide **7** S-23
S7 Total Absorbance Chromatogram of pure maleimide **8** S-24
S8 HPLC-UV(210 nm) Chromatogram for 1-(9H-fluoren-9-yl)-3,12-dioxo-2-oxa-7,8-dithia-4,11-diazahexadecan-16-oic acid S-25
S9 Total Absorbance Chromatogram of pure **Linker I** S-27
S10 Total Absorbance Chromatogram of pure **Linker II** S-28
S11 Total Absorbance Chromatogram of pure **Linker III** S-29
S12 Total Absorbance Chromatogram of pure **Linker IV** S-31
S13 Total Absorbance Chromatogram of pure Intermediate I S-32
S14 Total Absorbance Chromatogram of pure **Linker V** S-32
S15 Total Absorbance Chromatogram of pure pentapeptide Ac-Leu-Val-Cys-Ala-Phe-NH_2 . . . . S-33
S16 Hydrolysis of maleimide **1** HPLC traces S-34
S17 Hydrolysis of maleimide **2** HPLC traces S-34
S18 Hydrolysis of maleimide **5** HPLC traces S-35
S19 Hydrolysis of maleimides plotted data S-36
S20 Maleimide **5** immolation and hydrolysis studies S-38
S21 Maleimide **6** immolation and hydrolysis HPLC S-39
S22 Maleimide **7** immolation and hydrolysis studies S-41
S23 **Linker I** bioconjugation condition screening S-45
S24 On-protein **Linker I** immolation study S-47
S25 **anti-PD-L1 sdAb** homodimerisation with **Linker I** S-48
S26 anti-HER2 sdAb homodimerisation with **Linker I** S-50
S27 anti-HER2 affibody homodimerisation with **Linker I** S-51
S28 **anti-HER2/PD-L1** heterodimerisation with **Linker I** S-54
S29 **anti-HER2 sdAb/affibody** heterodimerisation with **Linker I** S-55

S30 **anti-HER2/CD3** trimerisation with **Linker II** S-59

S31 **anti-HER2/PD-L1** tetramerisation with **Linker II** and **Linker III** S-62

S32 **anti-HER2/CD3** trimerisation with **Linker V** S-65

S33 Proposed DBCO hydrolysis mechanism S-66

S34 IgG–sdAb conjugation S-68

S35 GSH stability assays S-69

S36 Plasma stability of **EGFP homodimer** S-70

S37 Biophysical characterisation of monomers S-72

S38 BLI for **anti-PD-L1 sdAb** derived homo- and heterodimer binders S-75

S39 BLI sensograms for HER2 binders S-76

S40 Bispecific dual engagement BLI assay S-78

S41 SKBR3 confocal images S-80

S42 MCF-7 confocal images S-81

S43 LC–MS spectra of **anti-PD-L1 sdAb** monomer S-84

S44 LC–MS spectra of **anti-HER2 sdAb** (2Rb17c) monomer S-85

S45 LC–MS spectra of **anti-HER2 affibody monomer** S-86

S46 LC–MS spectra of **anti-CD3 sdAb** monomer S-87

S47 LC–MS spectra of reduced, commercially sourced Thiomab S-88

S48 LC-MS spectra of **anti-PD-L1 sdAb** homodimerisation S-89

S49 LC-MS spectra of **anti-PD-L1 sdAb/Linker I** monomer S-90

S50 LC-MS spectra of pure **anti-PD-L1 homodimer** from **Linker I** S-91

S51 LC-MS spectra of pure **anti-PD-L1 homodimer** from commercial bis-maleimideS-92

S52 LC-MS spectra of pure **anti-HER2 sdAb homodimer** S-93

S53 LC-MS spectra of pure **anti-HER2 affibody homodimer** S-94

S54 LC-MS spectra of pure **anti-HER2 sdAb homodimer–DBCO** from **Linker V** S-95

S55 LC-MS spectra of **anti-HER2 sdAb/Linker I** monomer S-96

S56 LC-MS spectra of pure **anti-HER2/PD-L1 heterodimer** S-97
S57 LC-MS spectra of stabilised **anti-HER2 biparatopic heterodimer** S-98
S58 LC-MS spectra of **anti-PD-L1 sdAb–NMM** monomer S-99
S59 LC-MS spectra of **anti-HER2 sdAb/NMM** monomer S-100
S60 LC-MS spectra of **anti-HER2 affibody–NMM** monomer S-101
S61 LC-MS spectra of Alexa Fluor 488 labelled **anti-HER2 sdAb** monomer . . S-102
S62 LC-MS spectra of Alexa Fluor 488 labelled **anti-HER2 affibody** monomer S-103
S63 LC-MS spectra of Alexa Fluor 488 labelled thiomab S-104
S64 LC-MS spectra of Alexa Fluor 488 labelled **anti-HER2 sdAb** homodimer . S-105
S65 LC-MS spectra of Alexa Fluor 488 labelled **anti-HER2 affibody homodimer** S-106
S66 LC-MS spectra of Alexa Fluor 488 labelled **anti-HER2 biparatopic heterodimer** S-107
S67 LC–MS spectra of **anti-CD3/Linker III** S-108
S68 LC–MS spectra of **anti-HER2 sdAb homodimer** S-109
S69 LC–MS spectra of **anti-HER2/CD3 trimer** S-110
S70 LC–MS spectra of **anti-HER2 sdAb homodimer–Linker II** S-111
S71 LC–MS spectra of **anti-PD-L1 homodimer** S-112
S72 LC–MS spectra of **anti-CD3 sdAb** monomer–azide S-113
S73 LC–MS spectra of **anti-HER2 sdAb homodimer** generated from **Linker V** S-114
S74 LC–MS spectra of **anti-HER2/CD3 sdAb trimer** reaction S-115
S75 LC–MS spectra of **anti-PD-L1 sdAb/Linker IV** monomer S-116
S76 LC–MS spectrum of pre-stabilised **IgG/sdAb anti-HER2/PD-L1 bispecific** S-117
S77 LC–MS spectrum of stabilised **IgG/sdAb anti-HER2/PD-L1 bispecific** S-118
S78 LC–MS spectrum of Cysteine–tagged EGFP S-119
S79 LC–MS spectrum of EGFP homodimer S-120
S80 Anti-PD-L1 homodimer SEC chromatogram S-121
S81 Maleimide **5** ^{1}H and ^{13}C NMR S-122

S82 Maleimide **6** ^{1}H and ^{13}C NMR S-123
S83 Maleimide **7** ^{1}H and ^{13}C NMR 298 K S-124
S84 Maleimide **7** ^{1}H and ^{13}C NMR 353 K S-125
S85 Maleimide **7** HSQC NMR 298 and 353 K S-126

List of Tables

S1 Homodimerisation reaction conditions and isolated yields after SEC. S-52
S2 Heterodimerisation reaction conditions and isolated yields after SEC. S-56
S3 Trimerisation and tetramerization reaction conditions and isolated yields after SEC. S-63
S4 BLI derived kinetic parameters S-77

1 Experimental procedures

1.1 General experimental procedures

All solvents and reagents were purchased from commercial sources and used without further purification. EtOAc and dichloromethane were distilled on site. Commercially obtained LC–MS hypergrade ACN and MQ water prepared on site were used as solvents for the mobile phase in LC–MS experiments. NMR: ^{1}H and ^{13}C NMR spectra were recorded on Bruker 400-Neo Prodigy cryoprobe, Bruker 500-AVIII Dual 13C 1H cryoprobe, Bruker 500-AVIII or Bruker 700-AVII+ TCO cryoprobe NMR machines. Chemical shifts are reported in parts per million (ppm) with spectra calibrated to the residual solvent peaks (^{1}H NMR: DMSO-d6 δ_H 2.50 ppm, ^{13}C NMR: DMSO-d6 δ_C 39.5 ppm). Unless otherwise stated, all NMR measurements were acquired at 298 K (acquisition at 353 K was used to confirm the presence of rotamers at 298 K). MestReNova software (v. 14.2.0) was used for spectral processing. Chromatography: Purification via flash chromatography was performed over silica gel (Geduran silica gel 60, 40-63 µm; Merck). Reaction progress and elution of products

during chromatography were monitored by TLC (silica gel 60 on glass, with indicator F254; Merck) and visualised under UV light, or by staining with suitable solution prepared by known procedures. All buffers for bioconjugation were prepared on site. TCEP and reduced glutathione were prepared in the relevant buffer for each reaction and the pH adjusted as required. HPLC purification was performed using a Thermo Scientific Ultimate 3000 equipped with a YMC-Triart C18 column (250 × 10 mm, 5 μm) at 30 °C. Solvents A (water + 0.1% Formic acid) and B (ACN + 0.1% formic acid) were employed as mobile phase at a flow rate of 4.5 mL/min; HRMS was carried out on a Waters Xevo G2-S TOF mass spectrometer. SDS-PAGE was carried out using pre-cast NuPAGE^{TM} 4 to 12%, Bis-Tris, 1.0–1.5 mm, Mini Protein Gels and NuPAGE^{TM} MES SDS running buffer, at a constant voltage of 200 V, using SeeBlue^{TM} Plus2 Pre-stained Protein Standard and Instant Blue^{TM} Coomassie protein stain. Gels were imaged using a Bio-Rad ChemiDoc and quantified, when required, using Fiji ImageJ2 (version 2.14.0/1.54f).[S1]

1.2 General LC–MS protocols

LC–MS analysis of protein samples was carried out using a Waters SQD2 mass spectrometer using inlet method A for protein and B for peptides, or a Waters Xevo G2-S TOF mass spectrometer using inlet method C, in combination with an Acquity UPLC system with an Acquity UPLC BEH300 C4 column (130 Å1.7 µm, 2.1 × 50 mm) for proteins or an Acquity UPLC BEH C18 column (130 Å1.7 µm, 2.1 × 50 mm) for peptides and small molecules. The SQD2 mass spectrometer mobile phase consisted of solvent A (99.9% water with 0.1% formic acid), solvent B (99.9% ACN with 0.1% formic acid) and the following gradients were programmed. Inlet method A: 5% to 72% B in 6 min, then 72% B for 1.5 min followed by a gradient from 72% to 5% B over 0.25 mins and finally, 95% A for 1.25 mins. Inlet method B: 95% A for 0.5 mins, followed by a gradient for 5% to 90% A over 5.5 mins, then 90% B for 2.5 min followed a gradient from 90% to 5% B over 0.25 min and finally, 95% A for 3.25 min. The Xevo G2-S TOF mass spectrometer mobile phase consisted of solvent A

(99.9% water with 0.1% formic acid), solvent B (95% ACN and 5% water with 0.1% formic acid) and the following gradients were programmed. Inlet method C: 85% A for 1 min, then 15% to 80% B in 4 min, then 95% B for 2 min, then 95% to 15% B in 0.1 min followed by 0.7 min at 85% A. The capillary voltage of the electrospray source for the Waters SQD2 mass spectrometer was 3.0 kV with a cone voltage of 30 V and the desolvation gas used was nitrogen, with a flow rate of 800 L h^{-1}. For the Waters Xevo G2-S TOF mass spectrometer the capillary voltage of the electrospray source was 2.0 kV with a cone voltage of 40 V and the desolvation gas used was nitrogen, with a flow rate of 850 L h^{-1}. The ion series was obtained through integration of the major peaks of the chromatogram. Following this, the total mass spectra were reconstructed using the MaxEnt1 algorithm on the MassLynx software (v. 4.1), according to manufacturers guidelines.

1.3 Synthetic procedures

maleimide 1

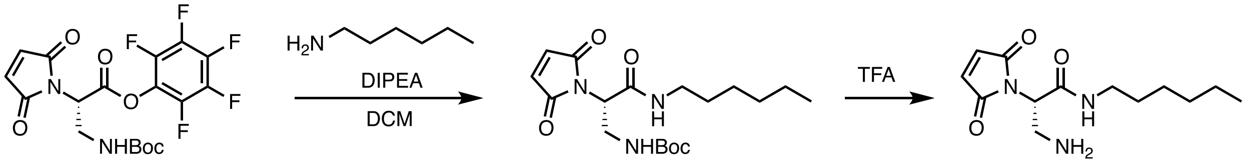

To a solution of perfluorophenyl (S)-3-((*tert*-butoxycarbonyl)amino)-2-(2,5-dioxo-2,5-dihydro-1H-pyrrol-1-yl)propanoate (50 mg, 0.11 mmol, 1 equivalent) in 2 mL dry DCM, DIPEA (20 μL, 0.11 mmol, 1 equivalent) and 1-hexylamine (15 μL, 0.11 mmol, 1 equivalent) were added consecutively at room temperature. The reaction mixture was stirred for one hour and completion was confirmed by LC–MS. Following this, TFA (2 mL) was added and the mixture was stirred for 2 h at room temperature. The solvent was removed under reduced pressure and the title compound was isolated as a white amorphous solid by semi-preparative HPLC using a linear gradient 5% B(1 min)>20 min>80% B (16.1 mg, 54%).

^{1}H NMR δ_H (500 MHz, DMSO-d6) 8.22 (t, J = 5.7 Hz, 1H), 8.03 (s, 3H), 7.09 (s, 2H), 4.74 (dd, J = 4.9, 9.1 Hz, 1H), 3.53–3.47 (m, 1H), 3.29–3.19 (m, 1H), 3.13–3.02 (m, 1H), 3.02–2.92 (m, 1H); ^{13}C NMR δ_C (126 MHz, DMSO-d6) 170.5, 163.1, 135.1, 49.5, 39.1, 38.1, 31.0, 28.8, 25.9, 22.1, 13.9; HRMS (ESI+): $[M+H]^+$ Calculated: $[C_{13}H_{21}N_3O_3]^{1+}$ 268.1661, Found: 268.1659.

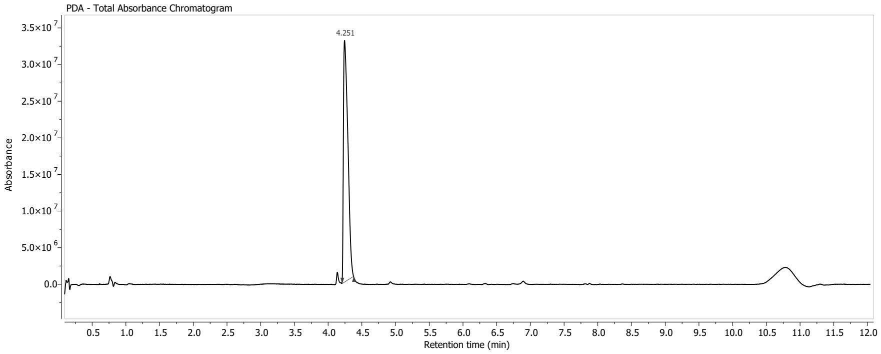

Figure S1: Total Absorbance Chromatogram of pure maleimide **1**

maleimide 2

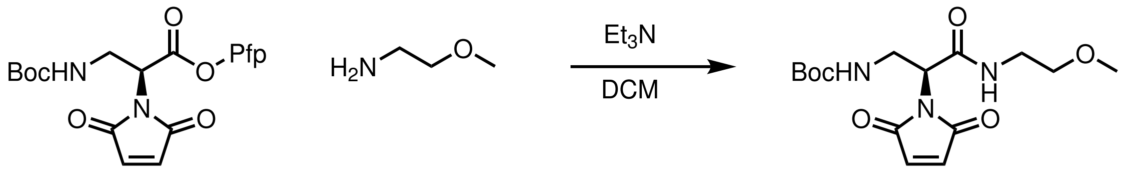

To a solution of perfluorophenyl (S)-3-((*tert*-butoxycarbonyl)amino)-2-(2,5-dioxo-2,5-dihydro-1H-pyrrol-1-yl)propanoate (50 mg, 0.11 mmol, 1 equivalent) and *N*-ethyl-*N*-isopropylpropan-2-amine (19 μL, 0.11 mmol, 1 equivalent) in dry DCM (5 mL), a solution 2-methoxyethan-1-amine (9.7 μL, 0.11 mmol, 1 equivalent) in dry DCM (1 mL) was added over a period of 10 min. The reaction mixture was stirred at room temperature for 2 h and the solvent was subsequently removed under reduced pressure. The title compound was isolated as an amorphous white solid by semi-preparative HPLC using a linear gradient 5% B(1 min)>20 min>80% B (29.4 mg, 78%).

^{1}H NMR δ_H (400 MHz, DMSO-d6) 8.07 (t, J = 5.6 Hz, 1H), 7.01 (s, 2H), 6.83 (t, J = 6.3 Hz, 1H), 4.46–4.40 (m, 1H), 3.62–3.03 (m, 9H), 1.29 (s, 9H); ^{13}C NMR δ_C (101 MHz, DMSO-d6) 171.0, 167.4, 156.1, 135.2, 78.2, 58.3, 52.6, 28.6; HRMS (ESI+): $[M+H]^+$ Calculated: $[C_{15}H_{24}N_3O_6]^{1+}$ 342.1665, Found: 342.1664.

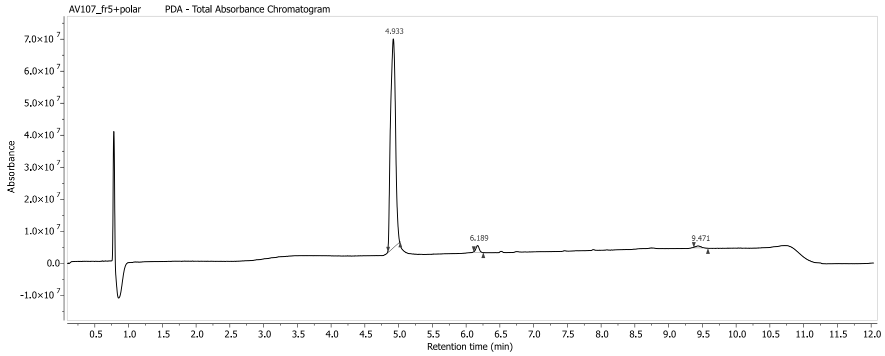

Figure S2: Total Absorbance Chromatogram of pure maleimide **2**

***Tert*-butyl 3-((2-((*tert*-butoxycarbonyl)amino)ethyl)amino)propanoate**

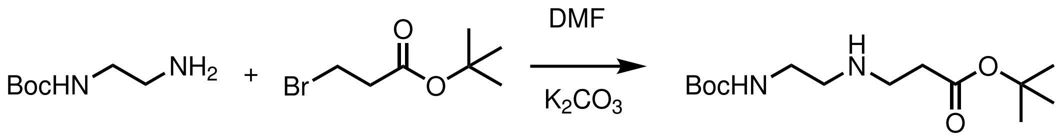

Potassium carbonate (611 mg, 4.42 mmol, 1 equivalent) and *tert*-butyl (2-aminoethyl)-carbamate (1.06 g, 6.63 mmol, 1.5 equivalents) were mixed in DMF (10 mL) and *tert*-butyl 3-bromopropanoate (924 mg, 4.42 mmol, 1 equivalent) was added with stirring. The reaction mixture was stirred at room temperature for 18 h and washed with water before extracting from the aqueous phase with EtOAc (3 × 25 mL) and purified by flash chromatography (9:1 DCM/MeOH) to afford the title compound as a colourless oil (404 mg, 32%), $R_f = 0.3$.

^{1}H NMR δ_H (500 MHz, DMSO-d6) 6.74 (t, J = 5.6 Hz, 1H), 3.13–2.92 (m, 3H), 2.77 (t, J = 6.9 Hz, 2H), 2.61 (t, J = 6.5 Hz, 2H), 2.38 (t, J = 6.9 Hz, 2H), 1.40 (s, 9H), 1.37 (s, 9H); ^{13}C NMR δ_C (126 MHz, DMSO-d6) 171.0, 155.6, 79.9, 77.7, 48.1, 44.1, 39.1, 34.7, 28.2, 27.8; HRMS (ESI+): $[M+H]^+$ Calculated: $[C_{14}H_{29}N_2O_4]^{1+}$ 298.2127, Found: 289.2136.

Ethyl 3-((((9H-fluoren-9-yl)methoxy)carbonyl)(2-((*tert*-butoxycarbonyl)amino)ethyl)amino)propanoate

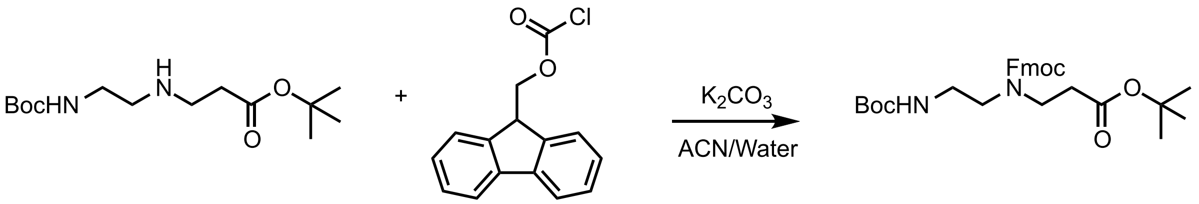

To a cold solution of *tert*-butyl 3-((2-((*tert*-butoxycarbonyl)amino)ethyl)amino)propanoate (40 mg, 0.14 mmol, 1 equivalent) and potassium carbonate (38 mg, 0.28 mmol, 2 equivalents) in ACN/Water (7:3) (3.6 mL), Fmoc-Cl (43 mg, 0.17 mmol, 1.2 equivalents) was added. The mixture was brought to room temperature and was stirred for 3 h. The crude reaction was diluted with water (5 mL) and extracted with EtOAc (3 × 20 mL). The organic layers were collected, dried over $MgSO_4$ and concentrated under reduced pressure. The title compound was isolated as a colourless oil by flash chromatography (Petrol/EtOAc 4:1) R_f= 0.61 (44 mg, 62%).

^{1}H NMR δ_H (500 MHz, 353 K, DMSO-d6) 7.82–7.87 (m, 2H), 7.59–7.64 (m, 2H), 7.37–7.43 (m, 2H), 7.27–7.35 (m, 2H), 6.42 (s, 1H), 4.38 (d, J = 6.1 Hz, 2H), 4.26 (t, J = 6.1 Hz, 1H), 3.28–3.32 (m, 2H), 2.94–3.01 (m, 2H), 1.39 (s, 9H), 1.36 (s, 9H); ^{13}C NMR δ_C (126 MHz, DMSO-d6) 170.7, 156.0, 155.7, 144.5, 141.3, 128.0, 127.5, 125.3, 120.4, 80.4, 78.2, 67.1, 47.6, 47.5, 44.3, 39.3, 34.6, 28.7, 28.3; HRMS (ESI+): $[M+H]^+$ Calculated: $[C_{29}H_{39}N_2O_6]^{1+}$ 511.2806, Found: 511.2808.

maleimide 4

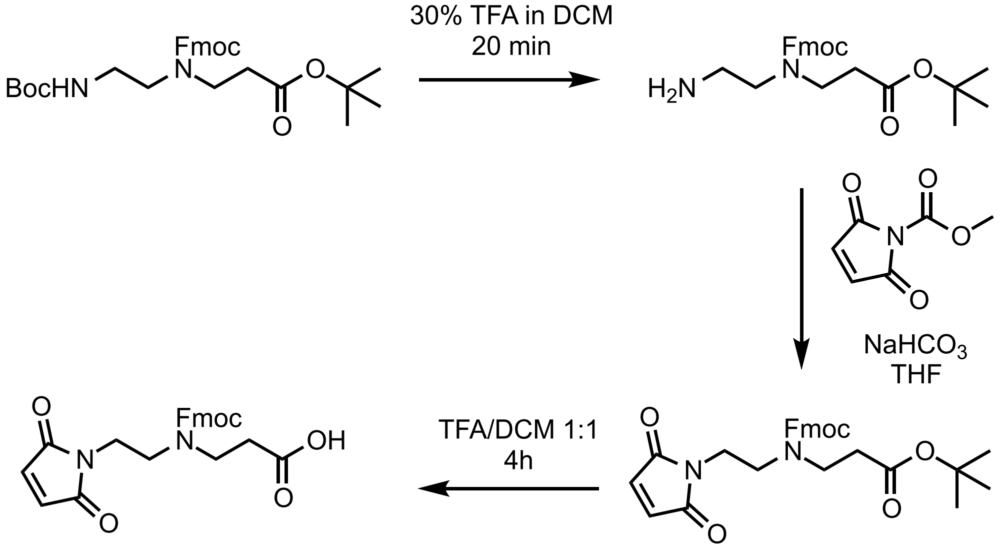

Tert-butyl-3-((((9H-fluoren-9-yl)methoxy)carbonyl)(2-((*tert*-butoxycarbonyl)amino)ethyl)-amino)propanoate (22 mg, 43 μmol, 1 equivalent) was dissolved in TFA/DCM (3:7) (10 mL) and stirred at room temperature for 20 min until completion of the *N*-Boc deprotection was confirmed by LC–MS. The solvents were removed under reduced pressure and the resulting pale-yellow oil was dissolved in THF (3 mL) and cooled to 0 °C before adding methyl 2,5-dioxo-2,5-dihydro-1H-pyrrole-1-carboxylate (10 mg, 65 μmol, 1.5 equivalents). When fully solubilised, cold, saturated $NaHCO_3$ solution (6 mL) was added and stirred at 0 °C. After 40 min, the reaction was brought to room temperature and the mixture was stirred for 50 min. The THF was removed under reduced pressure and the resulting residue was diluted in water (10 mL). The intermediate was extracted with EtOAc (3 × 20 mL), and the organic phase dried over anhydrous $MgSO_4$, and concentrated under reduced pressure. The resulting residue was dissolved in TFA/DCM (9:1) and stirred for 4 h. The solvent was removed under reduced pressure and the target compound was isolated as a colourless oil by semi-preparative HPLC using a linear gradient 5% B(1 min)>20 min>80% B allowed to isolate (6.8 mg, 36%).

^{1}H NMR (mixture of rotamers at 298 K) δ_H (700 MHz, DMSO-d6) 12.30 (s, 1H), 7.91–7.86

(m, 2H), 7.60 (d, J = 7.5 Hz, 2H), 7.45–7.39 (m, 2H), 7.34 (t, J = 7.4, 7.4 Hz, 2H), 7.09–6.93 (m, 2H), 4.36–4.11 (m, 3H), 3.59–3.45 (m, 2H), 3.42–3.35 (m, 2H), 3.34–3.25 (m, 2H), 2.46–2.21 (m, 2H); ^{13}C NMR δ_C (176 MHz, DMSO-d6) 173.3, 173.2, 171.4, 155.9, 155.4, 144.3, 144.1, 141.2, 141.2, 135.1, 135.0, 128.2, 128.1, 127.6, 127.6, 125.5, 125.4, 120.6, 67.5, 67.2, 47.1, 45.6, 43.8, 43.0, 36.0, 35.7, 33.3, 32.9; HRMS (ESI+): $[M+H]^+$ Calculated: $[C_{24}H_{23}N_2O_6S_2]^{1+}$ 435.1556, Found: 435.1379.

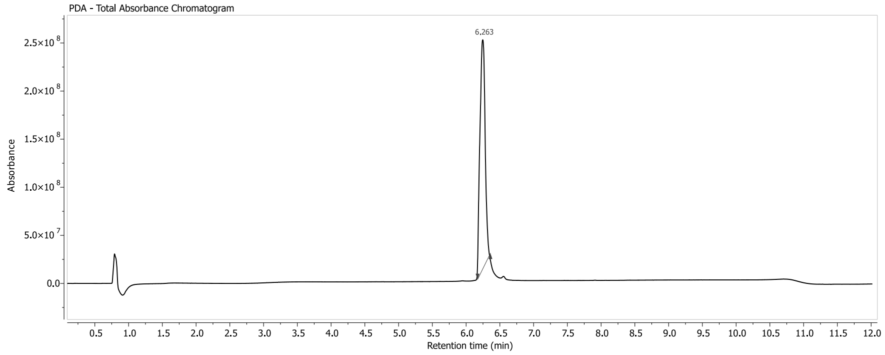

Figure S3: Total Absorbance Chromatogram of pure of maleimide **4**

Bis(2,5-dioxopyrrolidin-1-yl)(disulfanediylbis(ethane-2,1-diyl)) bis(carbonate)

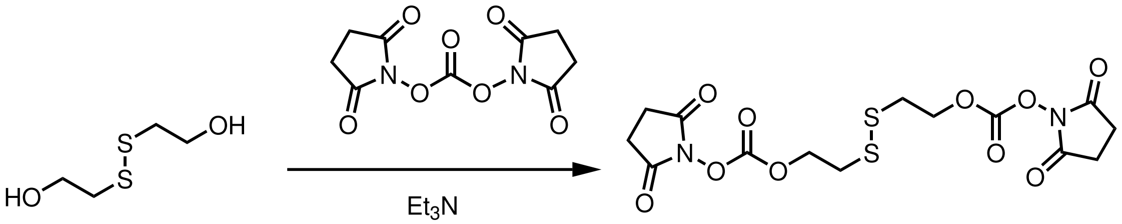

The title compound compound was synthesised following a reported protocol with minor changes.[S2] 2-hydroxyethyl disulfide (601.5 mg, 3.90 mmol, 1 equivalent) was dissolved in dry ACN (15 mL) under nitrogen. *N, N′*-disuccinimidyl carbonate (4 g, 15.6 mmol, 4 equivalents) was added, followed by triethylamine (3.26 mL, 23.4 mmol, 6 equivalents). The mixture was stirred at room temperature for 3 h, followed by concentrating under reduced pressure. The crude material was dissolved in DCM (100 mL) and the organic phase was washed with saturated $NaHCO_3$ solution (2 × 20 mL), saturated NH_4Cl solution (2 × 20 mL), and brine

(1 × 20 mL), before drying over Na_2SO_4. The solvent was subsequently evaporated and the intermediate was used directly in the next synthetic step without further purification.

2-((2-((((2,5-dioxopyrrolidin-1-yl)oxy)carbonyl)oxy)ethyl)disulfaneyl)ethyl (2-methoxyethyl)carbamate

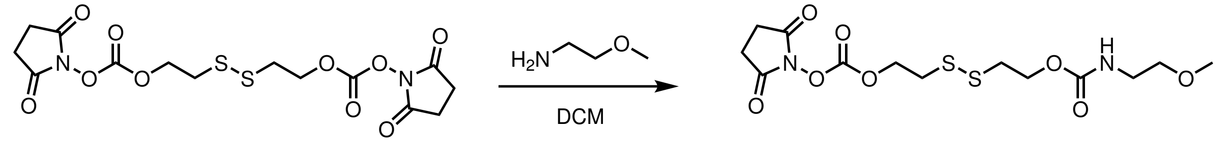

Bis(2,5-dioxopyrrolidin-1-yl)(disulfanediylbis(ethane-2,1-diyl))bis(carbonate) (1 g, 2.29 mmol, 1 equivalent) and DIPEA (399 μL, 2.29 mmol, 1 equivalent) were dissolved in ACN (8 mL) followed by 2-methoxyethan-1-amine (179 μL, 2.06 mmol, 0.9 equivalents) pre-dissolved in ACN (2 mL) was added over the course of 1 h and stirred for 2 h at room temperature. The solvent was removed under reduced pressure and the crude was purified by flash chromatography (DCM/MeOH 20:1), R_f= 0.49 to afford the title compound as a colourless oil (396 mg, 44%).

^{1}H NMR δ_H (500 MHz, DMSO-d6) 7.23 (t, J = 5.8 Hz, 1H), 4.61–4.54 (m, 2H), 4.18 (t, J = 6.2 Hz, 2H), 3.32–3.29 (m, 2H), 3.22 (s, 3H), 3.16–3.05 (m, 4H), 3.03–2.91 (m, 2H), 2.81 (s, 4H); ^{13}C NMR δ_C (126 MHz, DMSO-d6) 169.8, 156.0, 151.2, 70.6, 68.6, 61.6, 57.9, 40.0, 37.0, 35.7, 25.84. HRMS (ESI+): $[M+H]^+$ Calculated: $[C_{13}H_{21}N_2O_8S_2]^{1+}$ 397.0739, Found: 397.0737.

***tert*-butyl 16-(2-((*tert*-butoxycarbonyl)amino)ethyl)-6,15-dioxo-2,7,14-trioxa-10,11-dithia-5,16-diazanonadecan-19-oate**

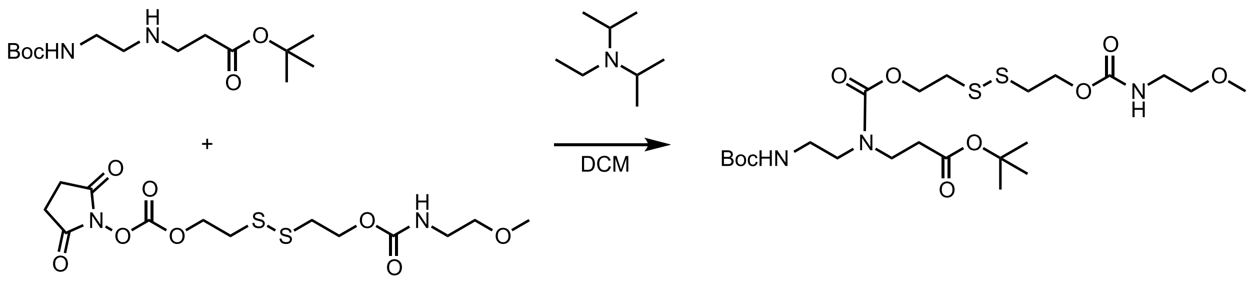

To a solution of *tert*-butyl 3-((2-((*tert*-butoxycarbonyl)amino)ethyl)amino)propanoate (188 mg, 653 μmol, 1 equivalent) and *N*-ethyl-*N*-isopropylpropan-2-amine (120 μL, 653 μmol, 1 equivalent) in dry DCM (4 mL), 2-((2-((((2,5-dioxopyrrolidin-1-yl)oxy)carbonyl)oxy)ethyl)disulfaneyl)ethyl (2-methoxyethyl)carbamate (259 mg, 653 μmol, 1 equivalent) in DCM (2 mL) was added. The mixture was stirred at room temperature for 1 h before being concentrated under reduced pressure. The title compound was isolated as a colourless oil by flash chromatography with Hexane/EtOAc (3:7), R_f= 0.36, (200 mg, 54%).

^{1}H NMR (mixture of rotamers at 298 K) δ_H (500 MHz, DMSO-d6) 7.22 (t, J = 5.7 Hz, 1H), 6.89–6.78 (m, 1H), 4.26–4.07 (m, 4H), 3.42–3.34 (m, 2H), 3.34–3.28 (m, 2H), 3.24–3.19 (m, 2H), 3.22 (s, 3H), 3.12 (q, J = 5.8 Hz, 2H), 3.08–3.00 (m, 2H), 2.99–2.90 (m,4H), 2.48–2.40 (m, 2H), 1.41–1.32 (m, 18H); ^{13}C NMR δ_C (126 MHz, DMSO-d6) 170.5, 155.9, 155.5, 155.0, 80.0, 77.6, 70.6, 62.6, 61.6, 57.8, 40.1, 39.9, 39.8 37.1, 36.8, 28.2, 27.7; HRMS (ESI+): $[M+H]^+$ Calculated: $[C_{23}H_{44}N_3O_9S_2]^{1+}$ 570.2519, Found: 570.2525.

maleimide 5

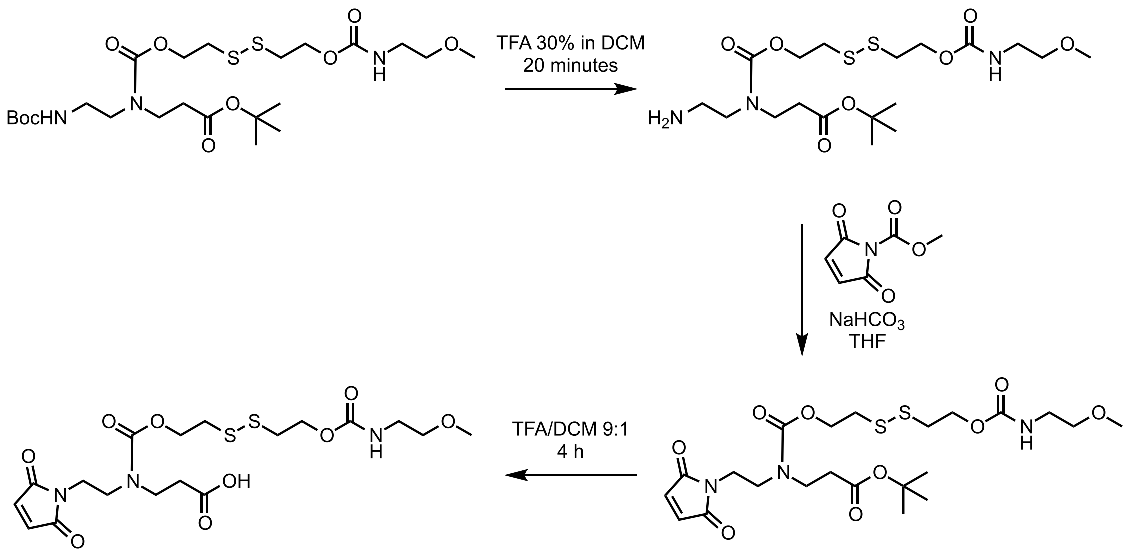

Tert-butyl 16-(2-((*tert*-butoxycarbonyl)amino)ethyl)-6,15-dioxo-2,7,14-trioxa-10,11-dithia-5,16-diazanonadeca*N*-19-oate (199 mg, 350 μmol, 1 equivalent) was dissolved in TFA/DCM (3:7) (10 mL) and stirred at room temperature for 20 min until completion of the *N*-Boc deprotection was confirmed by LC–MS. The solvent was removed under reduced pressure and the resulting pale-yellow oil was dissolved in THF (6 mL) and cooled to 0 °C before adding methyl 2,5-dioxo-2,5-dihydro-1H-pyrrole-1-carboxylate (81.4 mg, 0.53 mmol, 1.5 equivalents). When fully solubilised, cold $NaHCO_3$ solution (12 mL)was added and the reaction mixture was left to stir at 0 °C. After 40 min, the reaction was brought room temperature and stirred for an additional 50 min. The THF was then evaporated under reduced pressure, and the resulting crude residue was dissolved in 10 mL water. The intermediate was extracted from the aqueous phase with EtOAc (3 × 30 mL), and the organic phase dried over anhydrous $MgSO_4$, and concentrated under reduced pressure. The resulting mixture was dissolved in TFA/DCM (9:1) and stirred for 4 h. The solvent was removed under reduced pressure and the title compound was isolated as a colourless oil by semi-preparative HPLC using a linear gradient 5% B(1 min)>20 min>80% (74 mg, 43%).

^{1}H NMR (mixture of rotamers at 298 K) δ_H (700 MHz, DMSO-d6) 12.25 (s, 1H), 7.24–7.19 (m, 1H), 7.07–6.96 (m, 2H), 4.18–4.08 (m, 4H), 3.59–3.53 (m, 2H), 3.41–3.36 (m, 2H), 3.32–3.29 (m, 2H), 3.22 (s, 3H), 3.15–3.09 (m, 2H), 2.96–2.84 (m, 3H), 2.50–2.43 (m, 4H); ^{13}C NMR δ_C (176 MHz, DMSO-d6) 172.9, 170.9, 170.9, 156.0, 155.3, 154.8, 134.6, 70.6, 62.8, 62.5, 61.6, 57.8, 45.3, 45.0, 43.2, 42.7, 40.0, 37.1, 37.1, 36.7, 35.5, 35.3, 33.2, 32.4; HRMS (ESI+): $[M+H]^+$ Calculated: $[C_{18}H_{28}N_3O_9S_2]^{1+}$ 494.1267, Found: 494.1270.

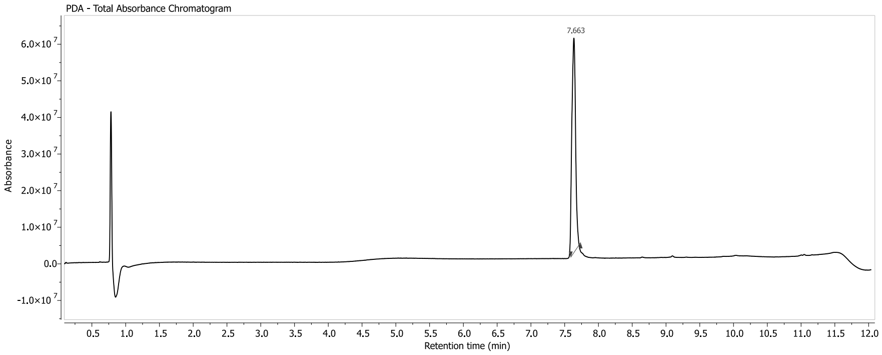

Figure S4: Total Absorbance Chromatogram of pure maleimide **5**

maleimide 6

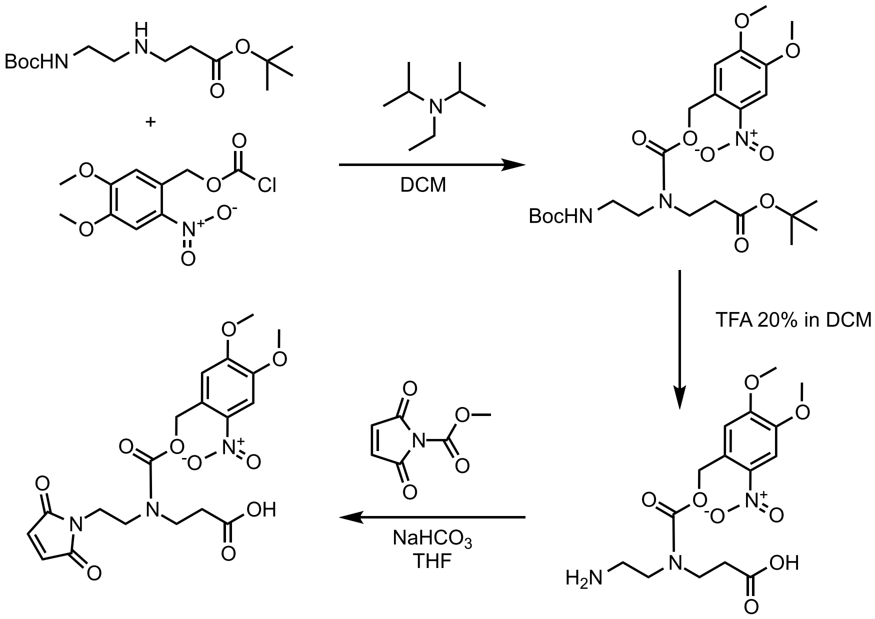

To a solution of *Tert*-butyl 3-((2-((*tert*-butoxycarbonyl)amino)ethyl)amino)propanoate (95 mg, 0.33 mmol, 1 equivalent) and DIPEA (115 μL, 0.66 mmol, 2 equivalents) in dry DCM (7 mL), a solution of 4,5-dimethoxy-2-nitrobenzyl carbonochloridate (100 mg, 0.36 mmol, 1.1 equivalents) in DCM (3 mL) was added over 5 min with stirring. The reaction mixture was stirred at room temperature for 1 h, followed by addition of TFA (2.5 mL). Stirring at room temperature for additional 4 h led to complete *N*-Boc and *tert*-butyl ester deprotection, as confirmed by LC-MS. The solvent was removed under reduced pressure and the resulting yellow oil was dissolved in THF (12 mL) and cooled to 0 °C before adding methyl 2,5-dioxo-2,5-dihydro-1H-pyrrole-1-carboxylate (76.5 mg, 0.49 mmol, 1.5 equivalents). Once in solution, cold saturated $NaHCO_3$ solution (12 mL) was added and the reaction mixture was left to stir at 0 °C. After 40 min, the reaction was brought to room temperature and stirred for additional 50 min. THF was removed from the aqueous mixture by evaporating under reduced pressure and the resulting mixture was diluted with water (10 mL). The aqueous solution was then brought to pH 3 by drop-wise addition of aqueous 5% HCl solution, and the product was extracted with EtOAc (3 × 30 mL). The combined organic layers were

dried over anhydrous $MgSO_4$, and the solvent evaporated under reduced pressure. The title compound was isolated as a pale yellow oil by semi-preparative HPLC using a linear gradient 5%B(1min)>20min>80% B (27 mg, 18%).

^{1}H NMR (mixture of rotamers at 298 K) δ_H (500 MHz, DMSO-d6) 12.31 (s, 1H), 7.70 (d, J=2.4 Hz, 2H), 7.11–7.05 (m, 2H), 6.94–6.89 (m, 2H), 5.34–5.20 (d, J=26.2 Hz, 2H), 3.94 (s, 3H), 3.88 (s, 3H), 3.62–3.55 (m, 2H), 3.50–3.35 (m, 4H), 2.55–2.45 (m, 2H); ^{13}C NMR δ_C (126 MHz, DMSO-d6) 172.8, 170.9, 153.3, 147.7, 139.3, 134.5, 134.4, 127.2, 110.6, 108.1, 63.4, 56.11, 56.09, 45.4, 42.7, 35.3, 33.1; HRMS (ESI+): $[M+H]^+$ Calculated: $[C_{19}H_{22}O_{10}]^{1+}$ 452.1348, Found: 452.1305.

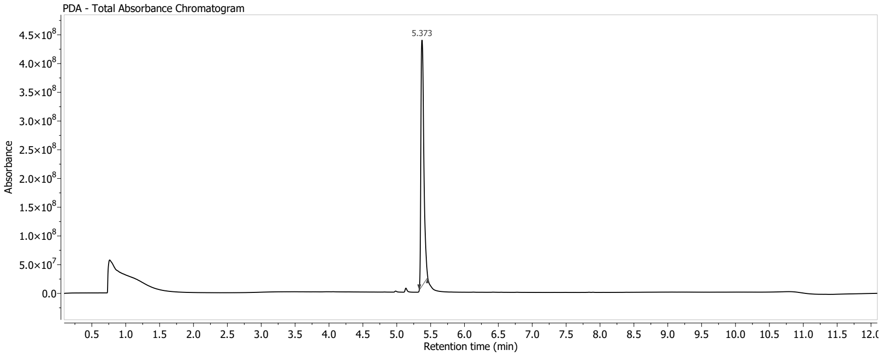

Figure S5: Total Absorbance Chromatogram of pure maleimide **6**

maleimide 7

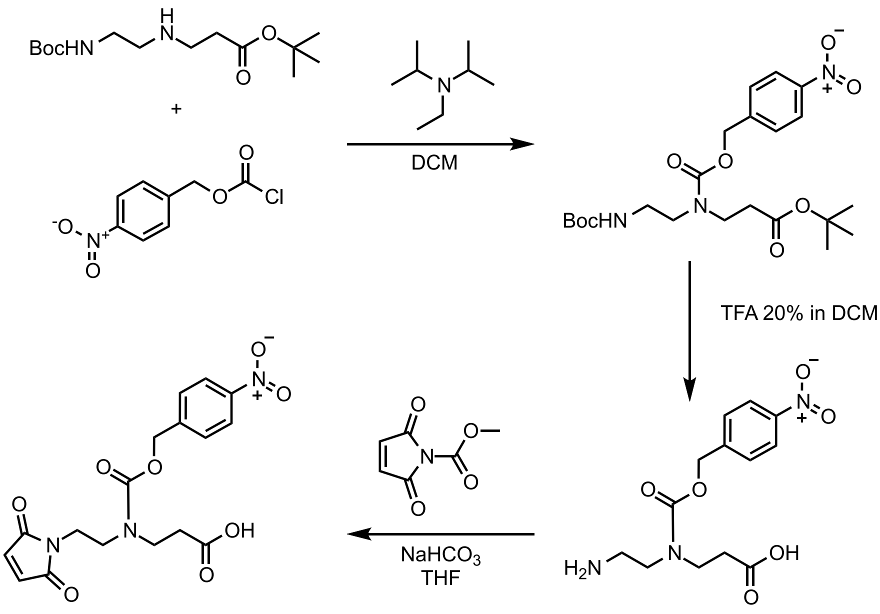

To a solution of *tert*-butyl 3-((2-((*tert*-butoxycarbonyl)amino)ethyl)amino)propanoate (245 mg, 0.85 mmol, 1 equivalent) and DIPEA (296 µL, 1.70 mmol, 2 equivalents) in dry DCM (15 mL), a solution of 4-nitro-benzylchloroformate (202 mg, 0.94 mmol, 1.1 equivalent) in DCM (5 mL) was added over 15 min with stirring. The reaction mixture was stirred at room temperature for 1 h, and the solvent was removed under reduced pressure and the residue was dissolved in TFA/DCM 1:4 (5 mL) solution and stirred at room temperature for 4 h until complete removal of the *N*-Boc and *tert*-butyl ester was confirmed by LC-MS. The solvent was removed under reduced pressure, and the residue washed with Et_2O (20 mL) followed by evaporating. This step was repeated twice. The resulting yellow oil was dissolved in THF (12 mL) and cooled to 0 °C before adding methyl 2,5-dioxo-2,5-dihydro-1H-pyrrole-1-carboxylate (198 mg, 1.27 mmol, 1.5 equivalents). After dissolving the residue THF, cold saturated $NaHCO_3$ solution (12 mL) was added and the reaction stirred for 40 min at 0 °C. The reaction was brought to room temperature and stirred for additional 50 min, followed by THF removal under reduced pressure. The resulting mixture was diluted in water (10 mL) and brought to pH 3 by drop-wise addition of aqueous 5% HCl solution, and the prod-

uct was extracted with EtOAc (3 × 30 mL). The combined organic layers were dried over anhydrous $MgSO_4$, and the solvent removed under reduced pressure. The title compound was isolated as an amorphous white solid by semi-preparative HPLC using a linear gradient 5%B(1min)>20min>80% B (103 mg, 31%).

^{1}H NMR (mixture of rotamers at 298 K) δ_H (500 MHz, DMSO-d6) 12.40–11.45 (bs, 1H), 8.22 (dd, J = 8.8, 2.5 Hz, 2H), 7.60–7.52 (m, 2H), 6.96–6-88 (m, 2H), 5.16–5.05 (m, 2H), 3.60–3.54 (m, 2H), 3.47–3.32 (m, 4H), 2.53–2.45 (m, 2H); ^{13}C NMR δ_C (126 MHz, DMSO-d6) 173.3, 171.4, 155.2, 145.3, 144.7, 135.02, 134.95, 128.8, 128.4, 124.0, 65.8, 65.5, 45.8, 43.1, 36.0, 35.7, 33.7, 32.9. ^{1}H NMR (single rotamer at 353 K) δ_H (500 MHz, DMSO-d6) 12.70–11.80 (bs, 1H), 8.23–8.17 (m, 2H), 7.58 (d, J = 8.8 Hz, 2H), 6.90 (s, 2H), 5.15 (s, 2H), 3.60(dd, J = 6.5, 5.1 Hz 2H), 3.52–3.40 (m, 4H), 2.53–2.47 (m, 2H); ^{13}C NMR δ_C (126 MHz, DMSO-d6) 172.0, 170.3, 154.7, 146.9, 144.1, 134.1, 127.8, 123.0, 64.9, 45.0, 43.0, 35.2, 32.7; HRMS (ESI+): $[M+H]^+$ Calculated: $[C_{17}H_{18}N_3O_8]^{1+}$ 392.1094, Found: 392.1104.

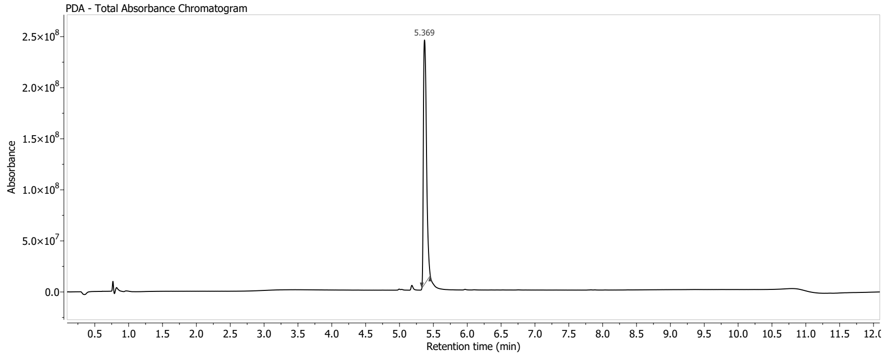

Figure S6: Total Absorbance Chromatogram of maleimide **7**

maleimide 8

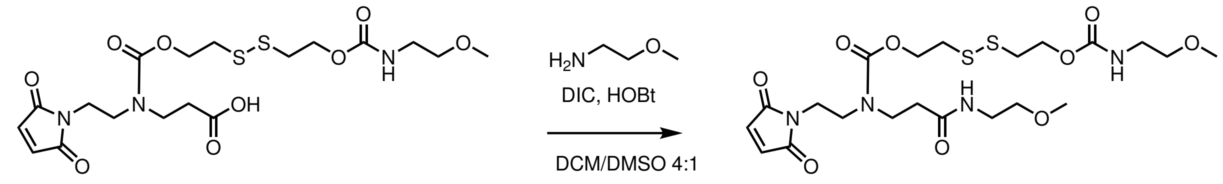

To a solution of 16-(2-(2,5-dioxo-2,5-dihydro-1H-pyrrol-1-yl)ethyl)-6,15-dioxo-2,7,14-trioxa-10,11-dithia-5,16-diazanonadecan-19-oic acid (10.0 mg, 20.3 μmol, 1 equivalent) and HOBt (3.41 mg, 22.3 μmol, 1.1 equivalents) in DCM/DMSO (4:1) (1 mL), *N, N′*-diisopropylcarbodiimide (3.49 μL, 22.3 μmol, 1.1 equivalents) was added. After stirring for 5 min at room temperature, 2-methoxyethan-1-amine (1.76 μL, 20.3 μmol, 1 equivalent) was added. The reaction mixture was stirred at room temperature for 2 h and the solvent was subsequently removed under reduced pressure. The title compound was isolated as a white amorphous solid by semi-preparative HPLC using a linear gradient 5% B(1 min)>20 min>80% B (2.8 mg, 25%).

^{1}H NMR (mixture of rotamers at 298 K δ_H (500 MHz, DMSO-d6) 8.00–7.93 (m, 1H), 7.22 (t, $J = 5.7$ Hz, 1H), 7.09–6.95 (m, 2H), 4.20–4.07 (m, 4H), 3.60–3.50 (m, 2H), 3.39–3.25 (m, 8H), 3.23 (s, 3H), 3.22 (s, 3H), 3.21–3.16 (m, 2H), 3.15–3.09 (m, 2H), 2.96–2.84 (m, 4H), 2.35–2.27 (m, 2H). ^{13}C NMR δ_C (126 MHz, DMSO-d6) 170.8, 170.2, 156.0, 134.6, 70.60, 70.59, 62.7, 61.7, 57.9, 57.8, 44.9, 43.7, 43.3, 39.9, 38.3, 37.0, 36.6, 35.5, 33.9; HRMS (ESI+): $[M+H]^+$ Calculated: $[C_{21}H_{35}N_4O_9S_2]^{1+}$ 551.1845, Found: 551.1852.

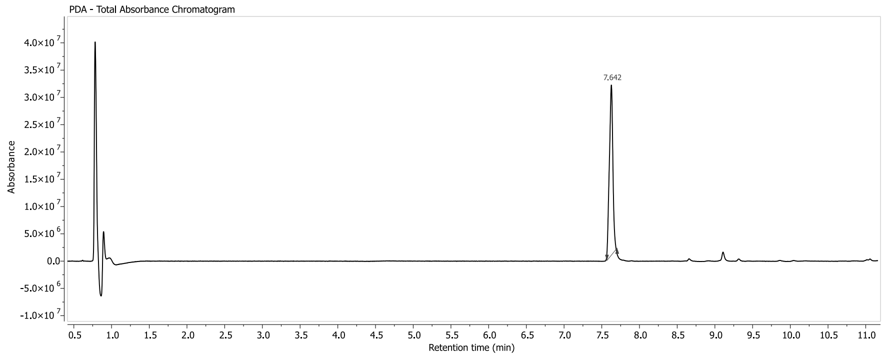

Figure S7: Total Absorbance Chromatogram of pure maleimide **8**

1-(9H-fluoren-9-yl)-3,12-dioxo-2-oxa-7,8-dithia-4,11-diazahexadecan-16-oic acid

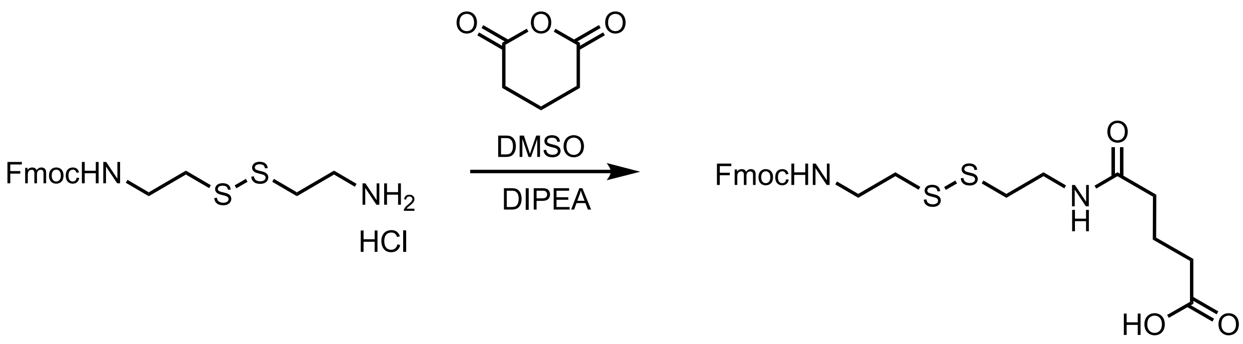

(9H-fluoren-9-yl)methyl (2-((2-aminoethyl)disulfaneyl)ethyl)carbamate, HCl (500 mg, 1 equiv., 1.22 mmol) and dihydro-2H-pyran-2,6(3H)-dione (139 mg, 1 equiv., 1.22 mmol) were weighted into a 10 mL flask and dissolved in 4 mL DMSO(HPLC analysis showed 4% conversion to the final product by this point). Then DIPEA (157 mg, 212 μL, 1 equiv., 1.22 mmol) was added and the reaction was checked after 30 min to observe full conversion. The final crude was dried overnight under nitrogen flow and used directly for the resin functionalization in the synthesis of **Linker II** without further purification. ESI-HRMS calculated for $[C_{24}H_{29}N_2O_5S_2]^{1+}$ 489.1512, *found* 489.1544.

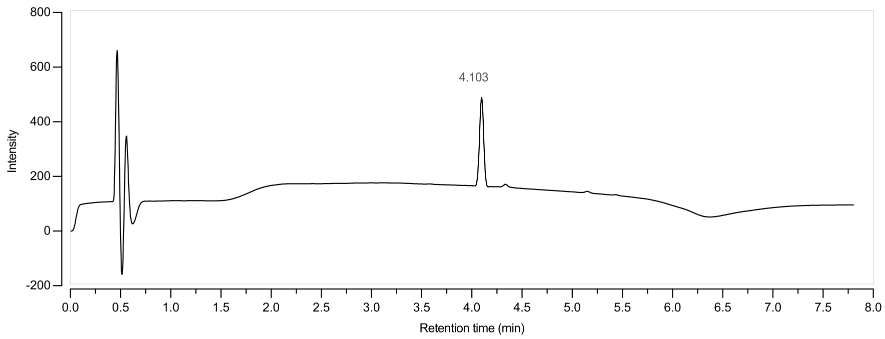

Figure S8: HPLC-UV(210 nm) Chromatogram for 1-(9H-fluoren-9-yl)-3,12-dioxo-2-oxa-7,8-dithia-4,11-diazahexadecan-16-oic acid. The chromatogram was adquired in a Thermo UltiMate 3000 HPLC System equipped with a Kinetex ® 5 μ m 50×4.6 mm LC column and using a linear gradient of B (AcN+0.1 % F.A.) in A(Water+0.1% F.A.) as follows: 5%B(1min)>6min>95% (2min)

.

1.4 Solid-phase peptide synthesis

General Procedure for solid-phase synthesis of peptides and linkers:

The linkers and peptides were assembled on solid support by a stepwise Fmoc/*t*Bu strategy and the required building blocks were either acquired from commercial sources or synthesised in-house. Unless indicated otherwise, coupling reactions were carried out manually on a 20–100 μmol scale on TG-S-RAM resin (0.23 mmol/g). *Swelling:* The resin was swelled for 20 min in DCM. *Fmoc removal:* The resin was treated with a solution of 20 % piperidine in DMF (2 × 10 min), then washed with DCM (3 × 1 min) and DMF (2 × 1 min). *Activation/Coupling:* Fmoc protected amino acid (4.0 equivalents) and HATU (3.9 equivalents) are dissolved in DMF (1 mL), then DIPEA (6 equivalents) was added. The mixture was pre-activated for 3 min, added to the resin, and stirred at room temperature for 30 min. The resin was washed with DCM (3 × 1 min) and DMF (2 × 1 min). *Final cleavage:* The resin was treated with the cocktail TFA/TIS/H_2O (95:2.5:2.5). The solvents from the cleavage cocktail was then evaporated under a constant flow of nitrogen and redissolved in MeOH before HPLC purification. For a more detailed description of the SPPS setup used, specialised literature with step-by-step protocols can be consulted.[S3,S4]

Linker I

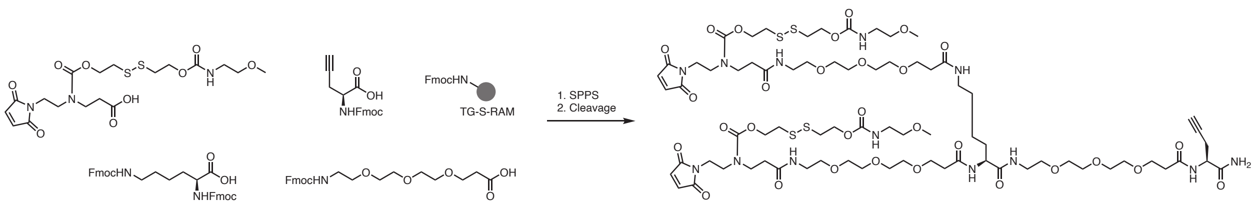

The synthesis of **Linker I** was performed on a 25 μmol scale following the *General Procedure for solid-phase synthesis of peptides and linkers. N*-terminus maleimide coupling was performed similarly to *Fmoc*-protected amino acids. The target compound was isolated as a colourless oil by HPLC purification (11.3 mg, 25%). HRMS (ESI+): $[M+H]^+$ Calculated: $[C_{74}H_{122}N_{13}O_{30}S_4]^{1+}$ 1800.7298, Found: 1800.7303.

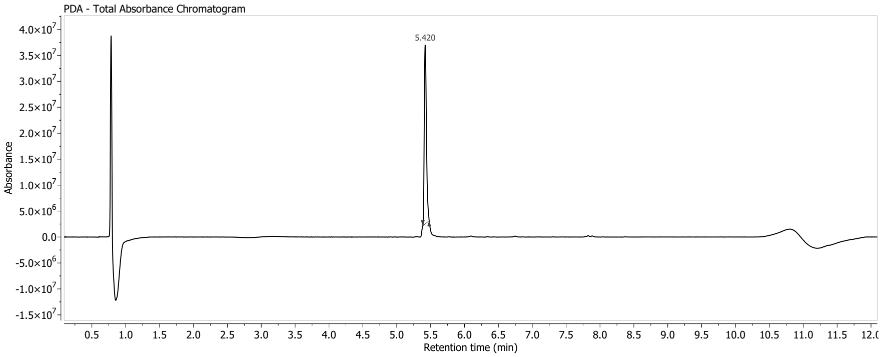

Figure S9: Total Absorbance Chromatogram of pure **Linker I**

Linker II

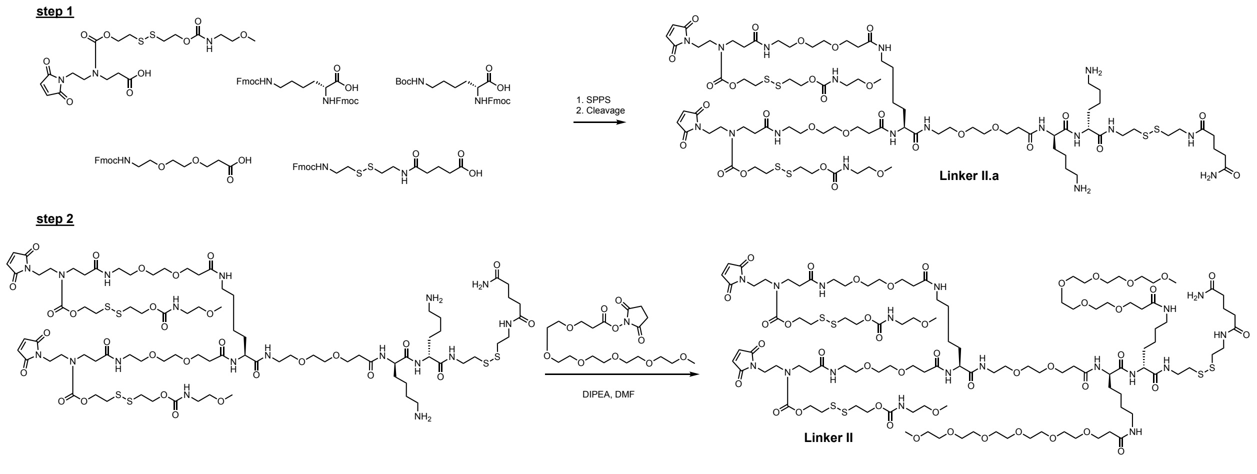

The synthesis of the linker was achieved in two steps. In the first step, intermediate **Linker II.a** was assembled on solid phase in a 25 μmol scale following the *General Procedure for solid-phase synthesis of peptides and linkers.* A total of 47.2 mg (91% yield) from the crude peptide were recovered after cleavage from the resin with a 66% purity estimated by HPLC-UV (210 nm). In a second step, the isolated crude **Linker II.a** intermediate (1 equiv., 25μmol) and m-PEG6-NHS ester (21 mg, 2 equiv., 50 μmol) were then dissolved in 4 mL dry DMF and *N*-methylmorpholine (25 mg, 27 μL, 10 equiv., 0.25 mmol) was added. Reaction completion was achieved after 30 minutes as checked by LC-MS. The crude reaction was then directly purified by preparative HPLC to afford 21 mgs of the desired compound (31% isolated yield) in the form of a colourless oil. ESI-HRMS calculated for $[C_{112}H_{197}N_{18}O_{44}S_6]^{3+}$ 896.7350, *found* 897.0511.

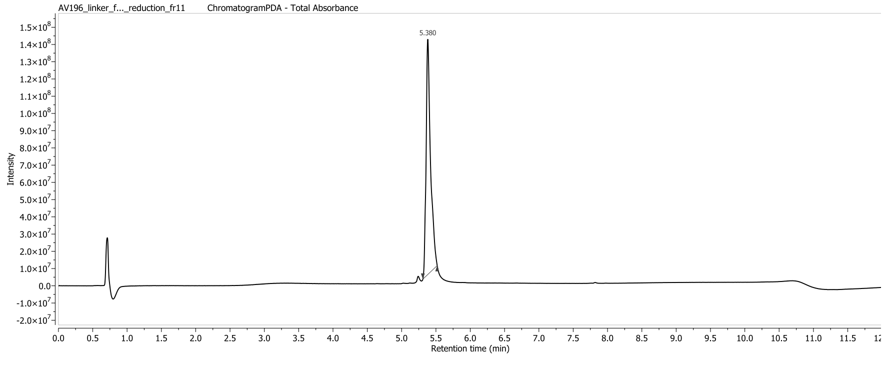

Figure S10: Total Absorbance Chromatogram of pure **Linker II**

Linker III

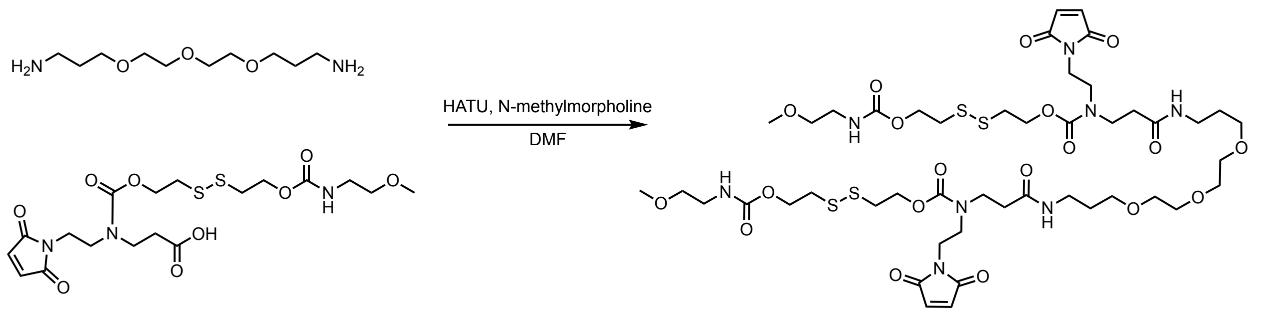

HATU (15 mg, 2 equiv., 41 μmol) and maleimide **5** (20 mg, 2 equiv., 41 μmol) were dissolved in 500 μL DMF and *N*-methylmorpholine (10 mg, 11 μL, 5 equiv., 0.10 mmol) was added. The solution was stirred for 10 minutes and subsequently the PEG-3 bis amine (4.5 mg, 1 equiv., 20 μmol) dissolved in 500 μL DMF was added. The mixture was left stirring for an additional hour at room temperature and completion was corroborated by LC-MS analysis. The crude was then directly purified by preparative HPLC to afford the pure bis-maleimide linker (9.1 mg, 38% yield) as a colorless oil. ESI-HRMS calculated for $[C_{46}H_{75}N_8O_{19}S_4]^+$ 1171.4026, *found* 1171.4033.

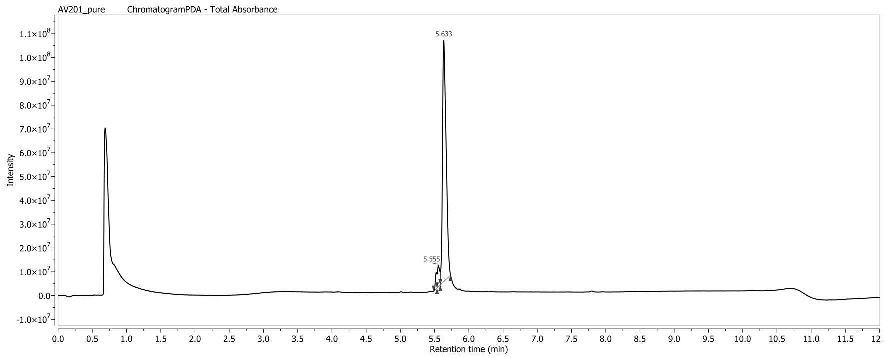

Figure S11: Total Absorbance Chromatogram of pure **Linker III**

Linker IV

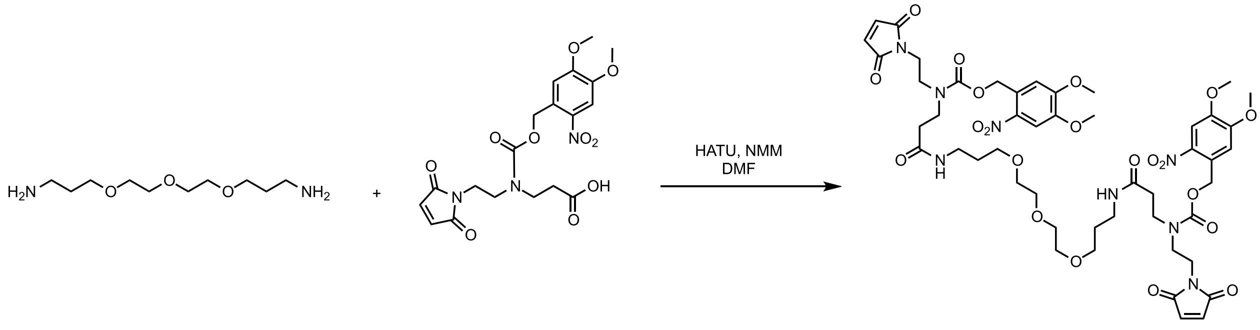

HATU (24 mg, 1 equivalent, 62 μmol) and 3-((((4,5-dimethoxy-2-nitrobenzyl)oxy)carbonyl)-(2-(2,5-dioxo-2,5-dihydro-1H-pyrrol-1-yl)ethyl)amino)propanoic acid (28 mg, 62 μmol, 1 equivalent) were dissolved in DMF (500 μL) and *N*-methylmorpholine (27 μL, 0.25 mmol, 4 equivalents) was added. The solution was stirred for 10 min, and subsequently a solution of 3,3'-((oxybis(ethane-2,1-diyl))bis(oxy))bis(propan-1-amine) (6.8 mg, 31 μmol, 0.5 equivalents) in DMF (500 μL) was added. The reaction mixture was stirred for 1 h at room temperature. The crude product was directly purified by preparative HPLC to afford the pure bis-maleimide linker as a transparent-to-pale-yellow oil (14.9 mg, 22%). HRMS (ESI+): $[M+H]^+$ Calculated: $[C_{48}H_{63}N_8O_{21}]^{1+}$ 1087.4102, Found: 1087.4105.

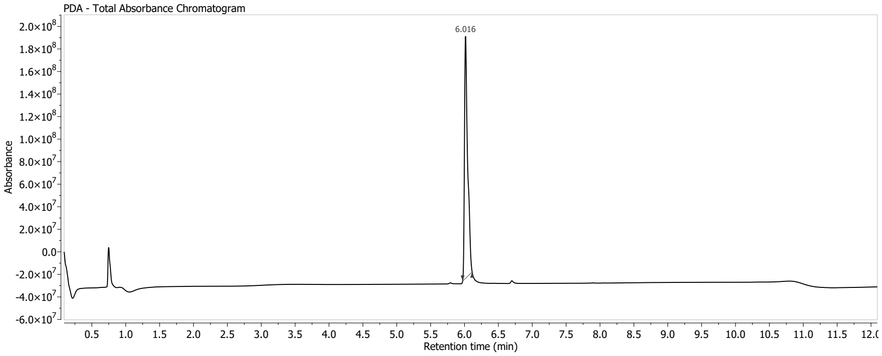

Figure S12: Total Absorbance Chromatogram of pure **Linker IV**

Linker V

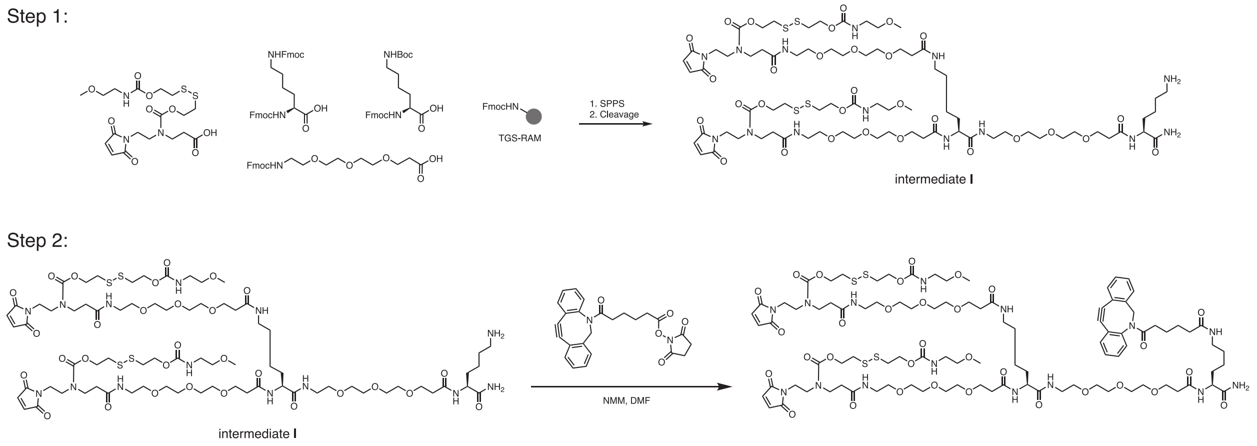

The synthesis of **Linker V** was achieved in two stages. First, Intermediate I was assembled on solid-phase on a 25 μmol scale following the *General Procedure for solid-phase synthesis of peptides and linkers.* Intermediate I was isolated as a colourless oil by HPLC purification (12.7 mg, 27%) and was found to be unstable upon long term storage at -20 °C. In the second stage, pure Intermediate I (5.5 mg, 3.00 μmol ,1 equivalent) and DBCO-NHS (1.94 mg, 4.50 μmol, 1.5 equivalents) were dissolved in DMF (1 mL). After stirring for 2 min at room temperature, 4-methylmorpholine (3.3 μL, 30 μmol, 10 equivalents) was added. The reaction mixture was stirred for additional 2 h at room temperature, after which time, full conversion to the DBCO-containing linker was confirmed by LC-MS. The target compound was subsequently isolated as a white amorphous solid upon direct purification by HPLC (4.3 mg, 67%

(one step)). HRMS (ESI+): $[M+H]^+$ Calculated: $[C_{96}H_{146}N_{15}O_{32}S_4]^{1+}$ 2148.9136, Found: 2148.9180.

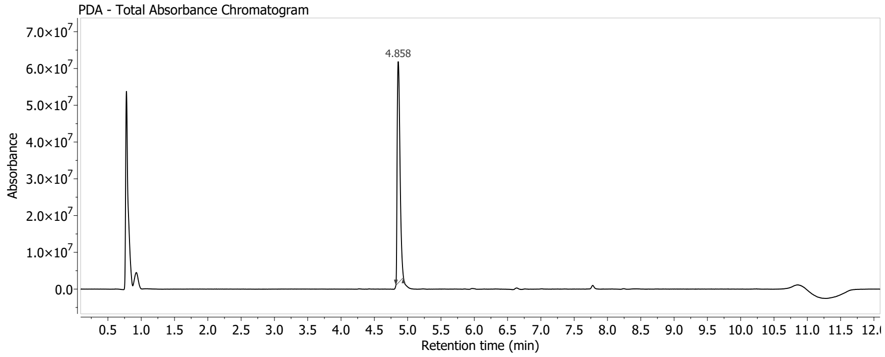

Figure S13: Total Absorbance Chromatogram of pure Intermediate I

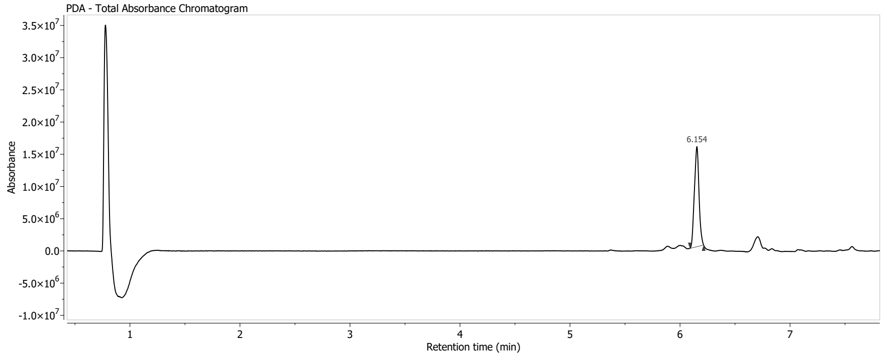

Figure S14: Total Absorbance Chromatogram of pure **Linker V**

Ac-Leu-Val-Cys-Ala-Phe-NH$_2$:

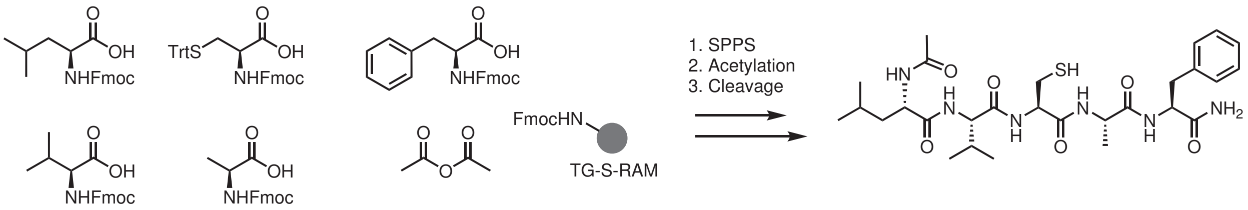

Synthesis of pentapeptide Ac-Leu-Val-Cys-Ala-Phe-NH$_2$ was performed on a 25 μmol scale following the *General Procedure for solid-phase synthesis of peptides and linkers. N*-terminus acetylation was performed before resin cleavage by reaction of the *N*-terminal-deprotected

peptide with DIPEA (43.5 μL, 250 μmol, 10 equivalents) and Ac_2O (23.6 μL, 250 μmol, 10 equivalents) in DMF for 30 min. The target compound was isolated as a white amorphous solid by HPLC purification (6.7 mg, 45%). HRMS (ESI+): $[M+H]^+$ Calculated: $[C_{28}H_{45}N_6O_6S_1]^{1+}$ 593.3116, Found: 593.3121.

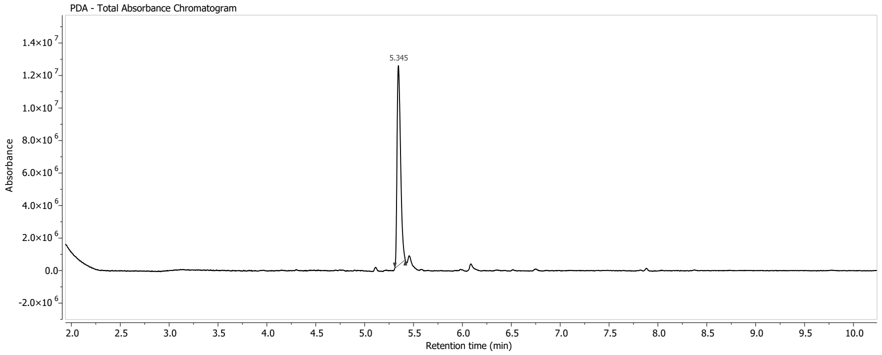

Figure S15: Total Absorbance Chromatogram of pure pentapeptide Ac-Leu-Val-Cys-Ala-Phe-NH_2

1.5 Maleimide hydrolysis studies

A stock solution of pentapeptide Ac-Leu-Val-Cys-Ala-Phe-NH_2 was prepared (10 mM, DMF) and diluted to a final concentration of 400 μM in NaPi (pH 7.0 20 mM). Stock solutions of maleimides, **1**, **2**, **3**, **4**, **5**, **6**, **7**, and **8** were prepared (20 mM, DMSO). To 200 μL of peptide, 16 μL of each maleimide solution was added (4 equivalents) and incubated at 25 °C. Multiple time points were taken from 0–30 h by diluting 5 μL of reaction mixture with 5 μL of formic acid solution (0.2%) to quench maleimide and succinimide hydrolysis. Hydrolysis was monitored by LC–MS and percentage hydrolysis of maleimide was determined by integrating the respective areas under their peaks in the full-PDA (190-500 nm) UPLC chromatogram relative to the non-hydrolysed species, unless indicated otherwise (Figure S19).

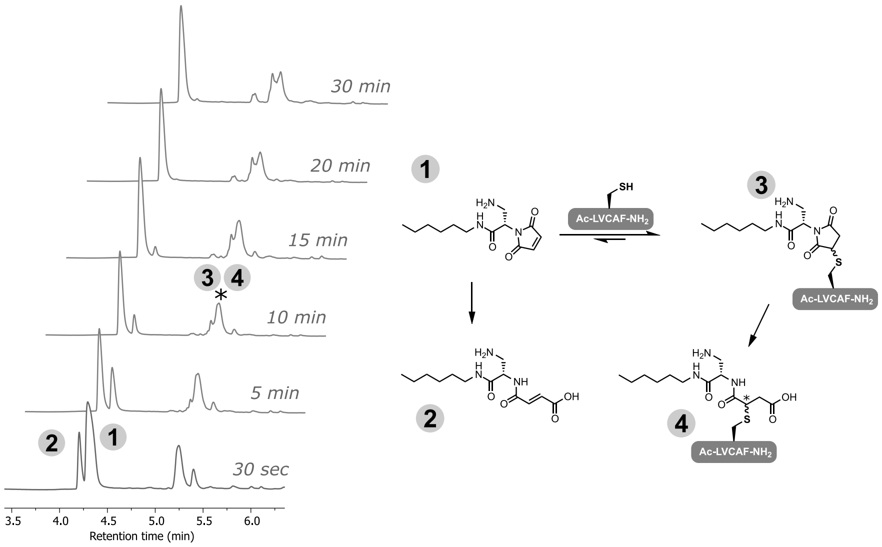

Figure S16: Hydrolysis of maleimide **1** and its corresponding conjugated succinimide as followed by LC-MS analysis. *Succinimide hydrolysis could lead to both 1,4- or 1,3-thio-substituted products.

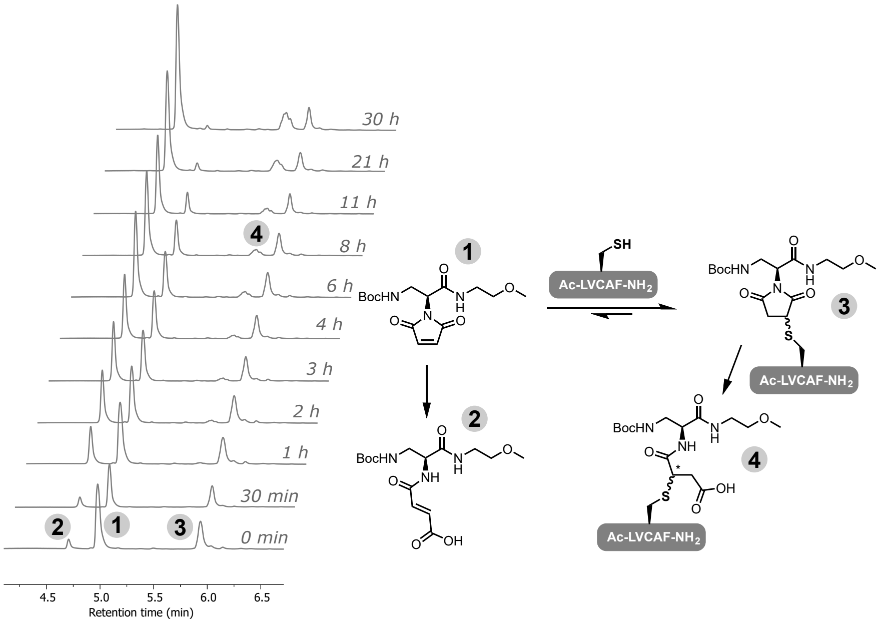

Figure S17: Hydrolysis of maleimide **2** and its corresponding conjugated succinimide as followed by LC-MS analysis.

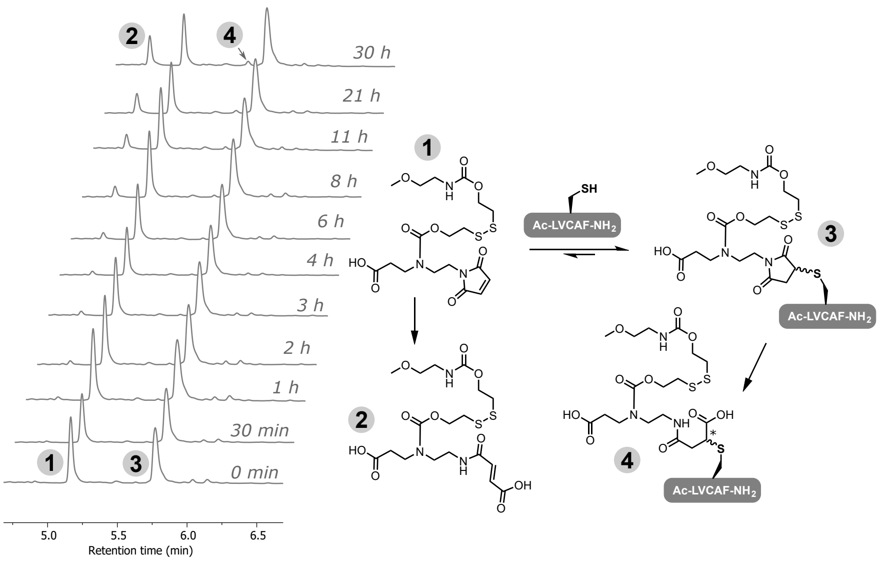

Figure S18: A representative chromatogram series for carbamate protected maleimide and thiosuccinimide in the form of maleimide **5** and its corresponding conjugated succinimide as followed by LC-MS analysis.

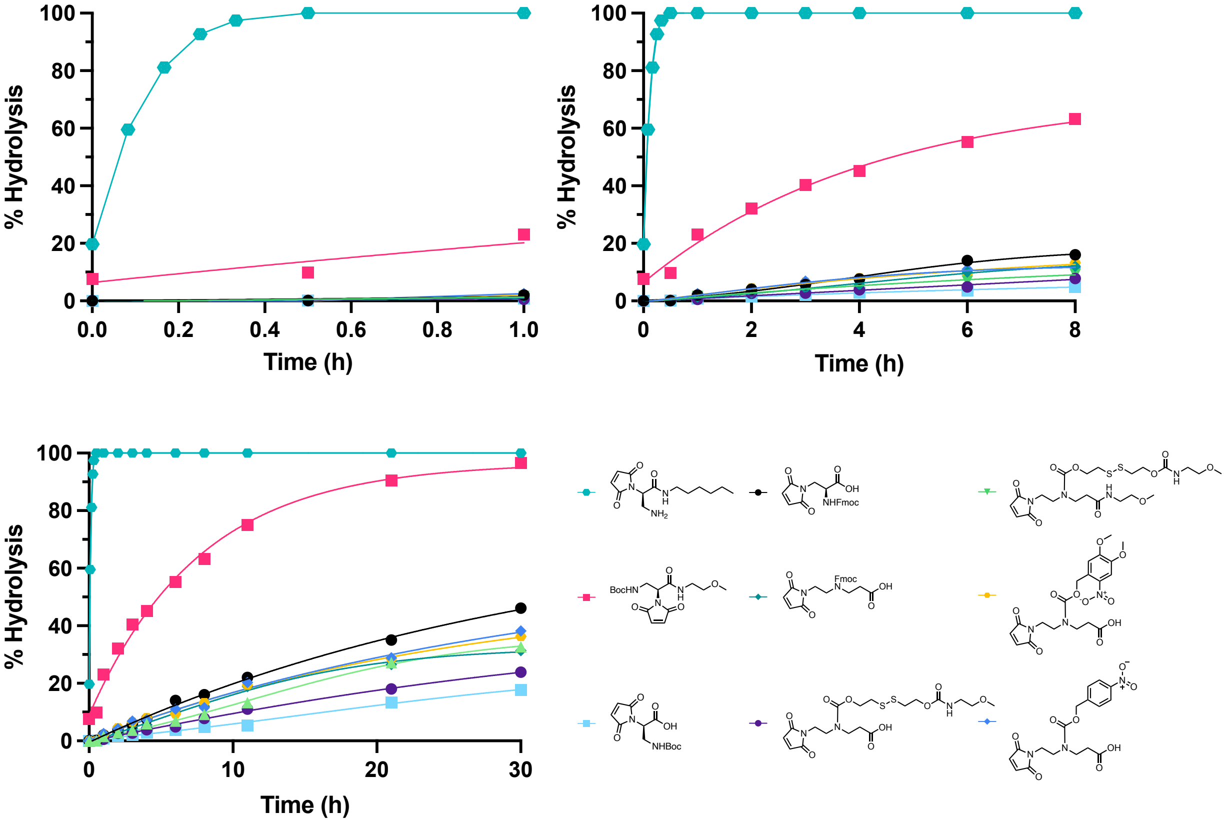

Figure S19: Rate of hydrolysis of unconjugated carbamate protected maleimides as determined by integration of the Full-PDA (190-500 nm) chromatogram. Data displayed on each graph is identical viewing a shorter time window on the x-axis.

1.6 Self-immolation and on-demand hydrolysis studies

1.6.1 Reductively triggered immolation

A stock solution of pentapeptide Ac-Leu-Val-Cys-Ala-Phe-NH_2 was prepared (10 mM, DMF) and diluted to a final concentration of 400 μM (200 μL) in NaPi pH 7.0, 7.5, 8.0 and Tris-HCl pH 8.5 (20 mM) buffers. To these solutions, maleimide **5** (16 μL, 20 mM DMSO, 4 equivalents) was added and incubated at 25 °C for 10 min. A sample of each reaction mixture was quenched upon 1:1 dilution with 0.2% formic acid and complete conjugation was verified. The samples were subsequently divided into two aliquots of 100 μL, and 10 μL of aqueous pH adjusted TCEP (20 mM, NaPi 20 mM, pH 7.5, 10 equivalents) was added and one sample of each pH incubated at 25 °C and 37 °C. Time points were taken and the reaction was quenched upon 1:1 dilution with 0.2% formic acid. Self immolation progress was monitored by LC–MS by integrating the relative areas under the peaks of reduced and self-immolated peptide–succinimide conjugates in the UV chromatogram.

*Note– shift in retention time between reduced, and self-immolated peaks was significant and integration was facile. However, upon immolation the peak of hydrolysed and ring-closed succinimides was not sufficiently resolved to integrate. It was clear that upon self-immolation, hydrolysis was extremely rapid as this was the major species observed. Self-immolation was deemed to be the rate-limiting step and upon completion of immolation after 3.5 h, the only mass detected was that of the hydrolysed species. (Figure S20a).

(a)

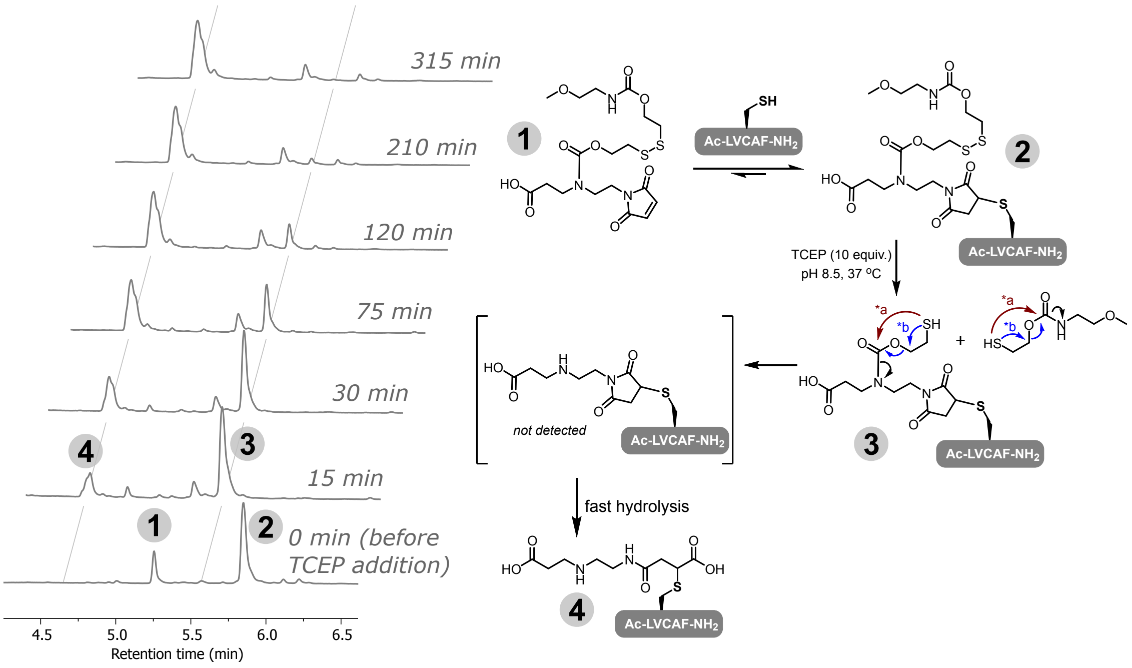

(b)

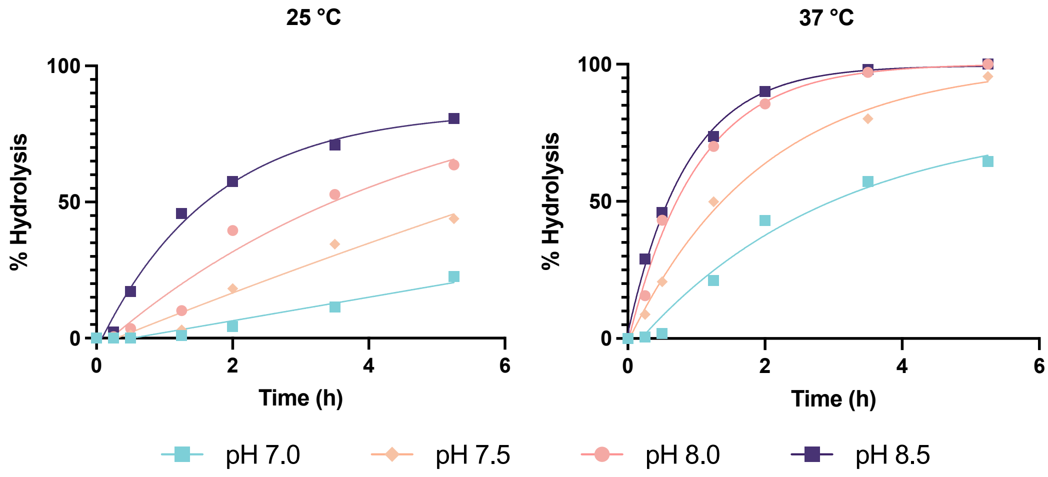

Figure S20: a) Immolation and hydrolysis of maleimide **5** derived thio-succinimide as followed by LC–MS analysis at pH 8.5 and 37 °C. Possible mechanisms for self-immolation upon disulfide reduction via 5- or 3-membered ring formation are represented by *a and *b, respectively.[S5–S7] The peak at 5.4 min in the chromatogram corresponds to peptide and arose from the oxidised peptide prior to treatment with TCEP. b) Percentage of self-immolation and hydrolysis of maleimide **5** derived thio-succinimide at pH 7.0-8.5 and 25 or 37 °C upon reductive triggering.

1.6.2 UV-decaging

A stock solution of pentapeptide Ac-Leu-Val-Cys-Ala-Phe-NH_2 was prepared (10 mM, DMF) and diluted to a final concentration of 150 μM (400 μL) in NaPi pH 7.5 (20 mM). To this solution, maleimide **6** (2.49 μL, 17.7 mM DMSO, 1.1 equivalents) was added and incubated at 25 °C for 10 min. A sample of each reaction mixture was quenched upon 1:1 dilution with 0.2% formic acid and complete conjugation was verified. The reaction mixture was subsequently irradiated under UV light at 365 nm using (EvoluChem LED 365DX, 25 mW/cm^2) at a 1 cm distance. Time points were taken and the reaction was quenched upon 1:1 dilution with 0.2% formic acid. The self immolation progress was monitored by LC–MS by integrating the relative areas under the peaks of self-immolated peptide–succinimide conjugates in the UV chromatogram. After 150 s immolation was complete, and the sample was incubated at 37 °C and hydrolysis was monitored and deemed complete when only hydrolysed peptide–succinimide conjugate could be detected by MS (Figure S21).

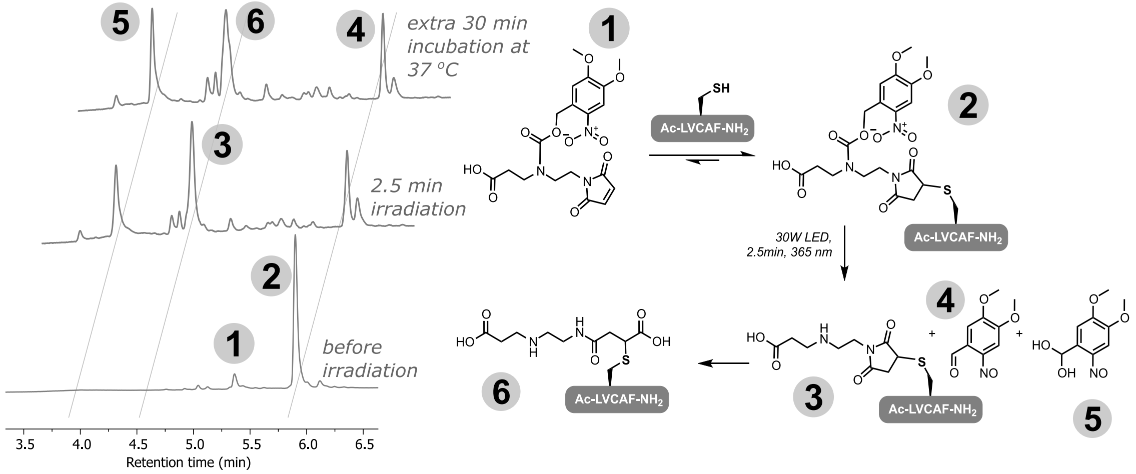

Figure S21: Immolation and hydrolysis of maleimide **6** derived thio-succinimide as followed by LC–MS analysis. A 2.5 min irradiation was sufficient for the full immolation and partial hydrolysis of the thio-succinimide, while full hydrolysis was achieved after incubation for 30 min at 37 °C as determined from the mass spectrum.

1.6.3 Enzymatic-decaging

A stock solution of pentapeptide Ac-Leu-Val-Cys-Ala-Phe-NH_2 was prepared (10 mM, DMF) and diluted to a final concentration of 100 μM (800 μL) in PBS pH 7.4 containing NADH (1 mM). To this solution, maleimide **7** (8 μL, 20 mM DMSO, 2 equivalents) was added and incubated at 25 °C for 10 min. A sample of each reaction mixture was quenched upon 1:1 dilution with 0.2% formic acid and complete conjugation was verified. The reaction mixture was divided into 6 aliquots of 100 μL and to these Nitroreductase enzyme (Sigma Aldrich: N9284) was added at 0, 0.4, 0.8, 2, 4 and 8 μM and incubated for 2.5 h at 37 °C, after which time the reactions were quenched upon 1:1 dilution with 0.2% formic acid. The reaction and self immolation conversion percentages were determined by LC–MS by integrating the relative areas under the peaks corresponding to non-hydrolysed and hydrolysed peptide–succinimide conjugates in the UV chromatogram (Figure S22a).

(a)

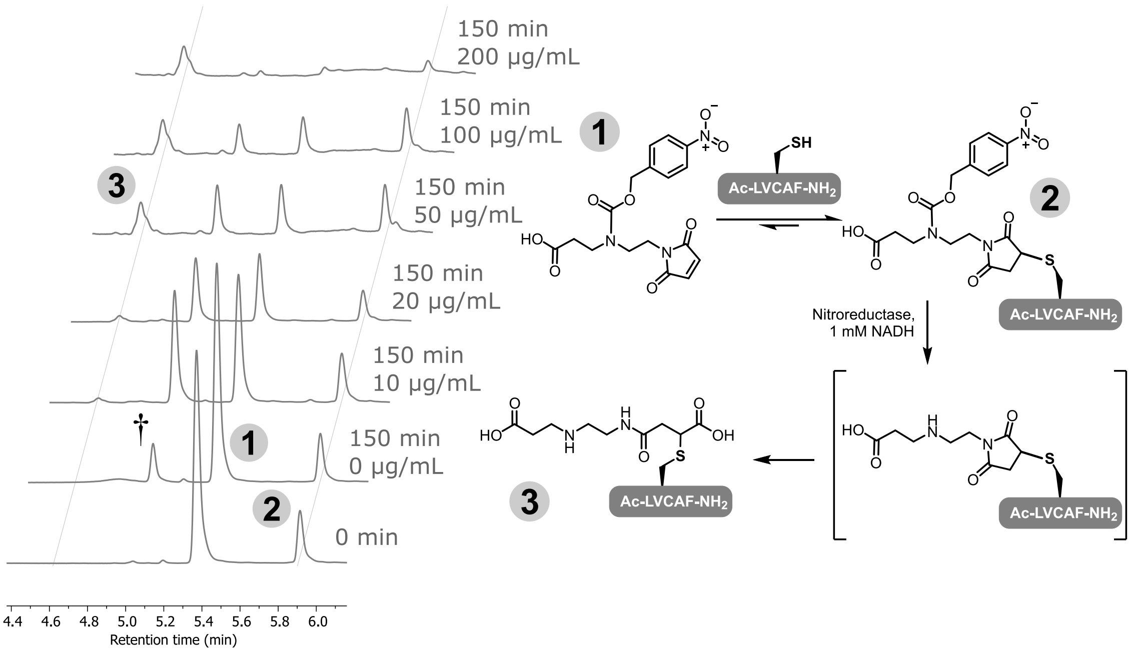

(b)

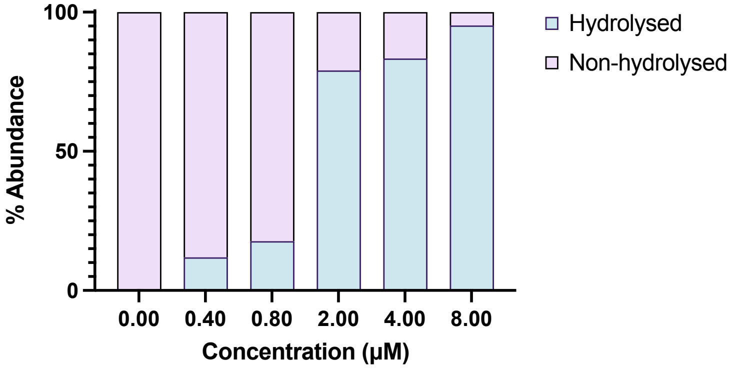

Figure S22: a) Immolation and hydrolysis of maleimide **7** derived thio-succinimide with increasing concentrations of nitroreductase, as followed by LC–MS analysis. † Corresponds to hydrolysed maleimide which has not been deprotected by nitroreductase. b) Percentage abundance of hydrolysed vs non-hydrolysed maleimide **7** derived thio-succinimide with increasing concentrations of nitroreductase.

1.7 Expression and purification of proteins

1.7.1 Expression of Nanobodies

Supplementary solutions for expression protocol:

20× P salts solution

1 M Na_2HPO_4

1 M KH_2PO_4

0.5 M $(NH_4)_2SO_4$

$MgCl_2$ stock 2 M

Glucose (40% w/v)

All solutions were made in MilliQ water and sterile filtered through 0.22 μm filter.

All plasmids used in this section were purchased from GenScript and the expression protocol was adapted from the publication entitled - 'Improving the yield of recalcitrant Nanobodies by simple modifications to the standard protocol'[S8]

Recombinant pET-26b(+) plasmids containing the genes for sdAbs targeting CD3, CTLA-4, HER2 and PD-L1 were transformed into BL21(DE3) competent *E. coli* (New England Biolabs: C2527H) and overnight cultures of these were grown in 2×YT media (30 mL) containing, 1× P salts, $MgCl_2$ (2 mM), glucose (0.5% w/v) and kanamycin (50 µg/mL) at 30 °C and 220 rpm. Overnight cultures were harvested by centrifugation at 3200×g for 10 min at 20 °C and the pellets resuspended in 2×YT media (6 mL). 3 mL of pre culture was used to inoculate 2×YT media (0.5 L) in 2 L baffled flasks containing, 1 × P salts, $MgCl_2$ (2 mM), glucose (0.5% w/v) and kanamycin (50 µg/mL).These were incubated at 37 °C, 220 rpm until an OD_{600} of 0.8 was reached. At which point, expression was induced upon addition of IPTG (250 µM final concentration). Cultures were subsequently incubated at 37 °C and 220 rpm for a further 4 h.

The cells were subsequently harvested by centrifugation at 11305×g and 4 °C for 30 min. The pellet was resuspended in 12 mL of TES buffer (200 mM Tris-HCl pH 8.0, 0.5 mM EDTA, 500 mM sucrose) per 1 L of expression media containing cOmplete^{TM} protease inhibitor cocktail tablets (Roche: 11836153001) and DNAse (10 μg/L of bacterial culture). The suspension was gently mixed by rolling in 50 mL falcon tubes overnight at 4 °C. To the resuspended pellet, 24 mL of ice cold $MgSO_4$ (5 mM) was added to initiate osmotic shock and gently mixed by rolling for 2 h at 4 °C. The cells were then harvested by centrifugation at 11305×g and 4 °C for 30 min and the supernatant was collected. The cell pellet was collected and the procedure repeated by addition of ice-cold TES (12 mL) and incubation for 1 h followed by 24 mL of ice-cold $MgSO_4$ (5 mM) for 2 h. The supernatant of the second osmotic shock was collected inline with the first round of osmotic shock. Ni-NTA resin equilibrated with PBS (pH 7.4) was added into the periplasmic extracts (2 mL/L of bacterial culture). The mixture was incubated at room temperature with gentle mixing on rollers for 3 h. The resin was subsequently loaded into an empty PD-10 column and then washed with 20 column volumes of PBS (pH 7.4) supplemented with NaCl (500 mM). The bound protein was eluted with an imidazole gradient consisting of 10 mM (3 mL), 40 mM (5 mL), 200 mM (2 mL) and 200 mM (5 mL) (all imidazole solutions were in PBS pH 7.4). The fractions were collected and analysed by SDS–PAGE before combining pure protein containing fractions.

Following combination of fractions, all samples were desalted using HiPrep^{TM} 26/10 Desalting column (Cytiva, Little Chalfont, UK) eluting with PBS (pH 7.4). Protein concentrations were subsequently determined by the absorption at 280 nm using a NanoDrop 2000c UV-Vis spectrophotometer. Theoretical extinction coefficients were calculated by ProtParam-Tool ExPASy (http://expasy.org/tools/prot-param.html) using the proteins' amino acid sequences.

1.7.2 Expression of Affibody

BL21(DE3) cells inoculated with the recombinant pET-28a(+) plasmid containing the gene for the affibody (R10C variant) were cultured overnight in 2×YT media (5 mL) supplemented with 50 mg/mL kanamycin (5 μL) at 37 °C. The pre-culture was added to a non-baffled flask containing LB media (500 mL) supplemented with 50 mg/mL kanamycin (500 μL), until reaching an OD600 of 0.02 and the cells were cultured at 37 °C, 200 rpm, and monitored every 30 min until an OD600 of 0.9 was reached. Expression was induced upon addition of 250 mg/mL IPTG (500 μL) and cultured at 37 °C for 4 h. The cells were harvested by centrifugation at 8000 rpm for 10 min. The pelleted cells were resuspended in 30 mL of lysis buffer (NaPi pH 7.5 50 mM, 500 mM NaCl, supplemented with DNAse and a Pierce complete protease tablet) and lysed over by sonicating (60% amplitude, 2 s on, 4 s off, for 5 min [15 min total]). The lysed cells were centrifuged at 5000 rpm (2× 10 min), and the supernatant was collected and filtered through a 0.22-μM filter. The filtered supernatant was applied onto a pre-equilibrated 10 mL Ni-excel column (0.5 mL of resin/column) and the capped column was incubated with gentle shaking for 1.5 h at 4 °C. The bound protein was eluted with an imidazole gradient consisting of 10 mM (6 mL), 40 mM (6 mL) and 200 mM (6 mL) (all imidazole solutions were in PBS [pH 7.4]). The 200 mM imidazole, protein containing, fraction was dialysed against HEPES buffer (HEPES 50 mM, 200 mM NaCl, pH 7.5, 5% ethylene glycol).

1.8 Protein bioconjugation experiments

1.8.1 Optimising dimerisation conditions

Anti-PD-L1 sdAb (33.2 μM, 50 μL) in PBS (pH 7.4) was reduced for 1 h at 37 °C using 10 equivalents of TCEP. After checking completion of the reduction step by LC–MS, the reaction mixture was buffer exchanged into PBS (pH 7.4) using Zeba^{TM} spin desalting columns to remove excess TCEP. A range from 0.6–20 equivalents of **Linker I** was added and the reactions were left to incubate at 25 °C for 10 min. The samples were quenched by

diluting 1:1 with formic acid (0.2%) and analysed by SDS-PAGE (Figure S23b).

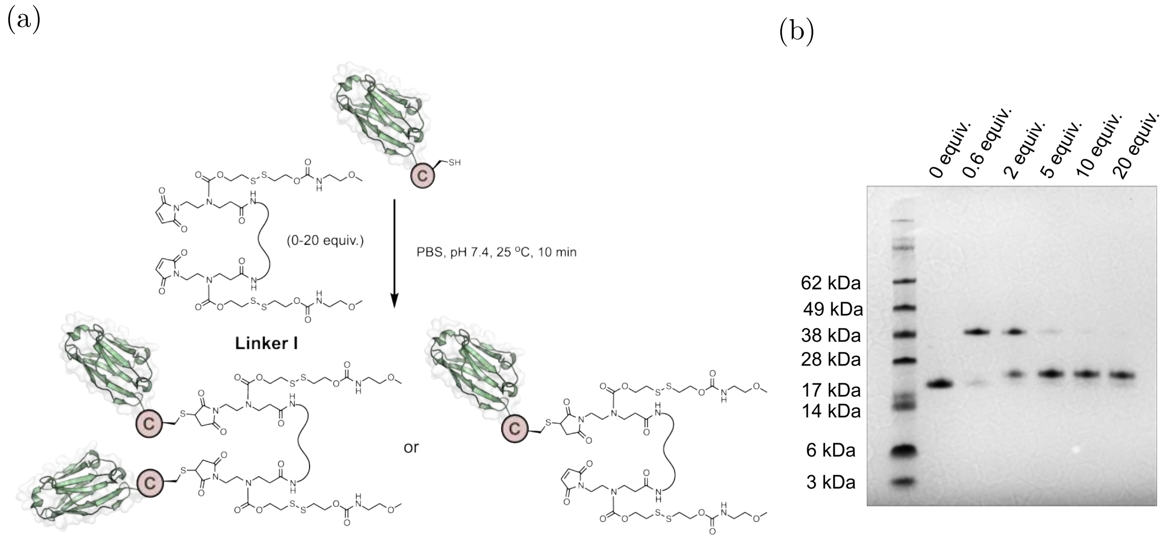

Figure S23: a) Scheme of **anti-PD-L1 sdAb** reaction with increasing equivalents (0–20 equivalents) of the homobifunctional **Linker I** b) SDS-PAGE analysis of the reaction after 10 min. In order to achieve fully functionalised monomer with no homodimer formation, 10–20 equivalents of linker were required.

1.8.2 Optimised method for the assembly and stabilisation of homodimeric protein–protein conjugates

A 100-3370 μL protein solution in PBS (pH 7.4) (concentration ranging from 33.2-230 µM) was reduced for 1 h at 37 °C using 10 equivalents of TCEP. After checking completion of the reduction step by LC–MS, the reaction mixture was buffer exchanged into PBS (pH 7.4) using Zeba^{TM} spin desalting columns to remove excess TCEP. Following this, the bis-maleimide linker was added (0.6 equivalents). In the case of **anti-HER2 sdAb**, which was found to rapidly re-oxidise to form intermolecular disulfides when using Zeba^{TM} spin desalting columns and PBS (pH 7.4), Pierce^{TM} strong cation exchange mini spin columns were used to remove excess TCEP. The protein was immobilised using histidine-acetate buffer 20 mM (pH 5.5) eluted directly into bis-maleimide linker, with PBS + 500 mM NaCl (pH 7.4).The reaction was incubated for a further 10 min and the pH of the mixture was raised to pH 8.2 by

diluting 9:1 with Tris-HCl (pH 8.2, 500 mM). Finally, thio-succinimide self-immolation and hydrolysis was triggered by addition of 10 equivalents of TCEP and incubation at 37 °C for a period of 1-2 h. After accomplishing the full thio-succinimide hydrolysis (as determined by LC–MS analysis), the dimer was purified by size exclusion chromatography using a 10/300 Superdex Increase 75 GL column (Cytiva, Little Chalfont, UK) and PBS (pH 7.4) as an elution buffer.

(a)

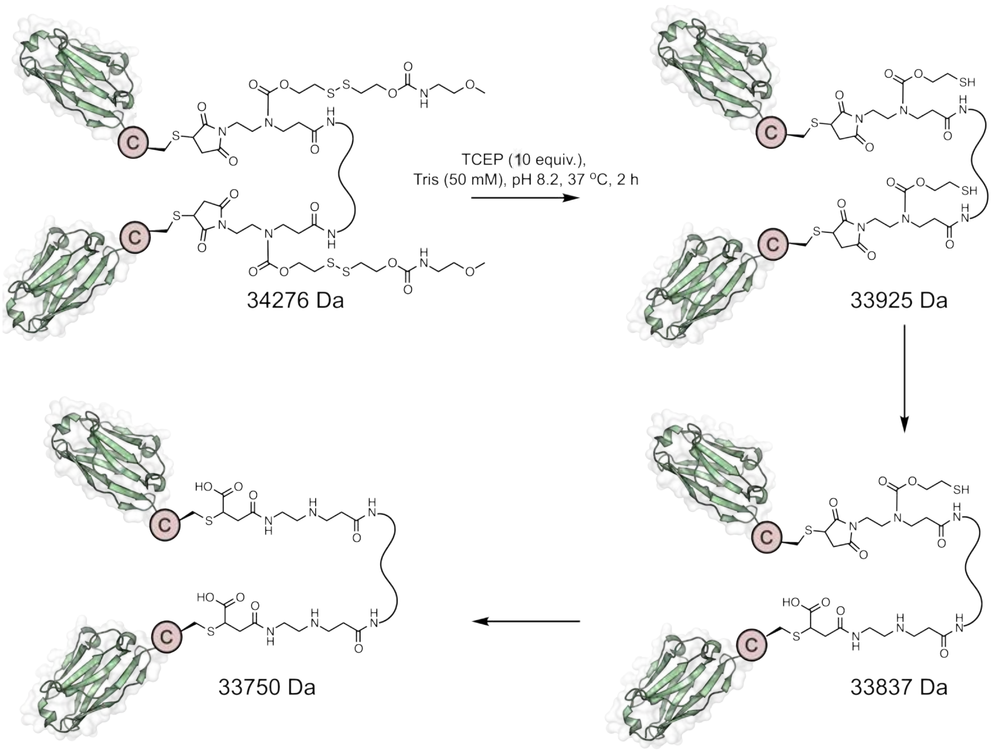

(b)

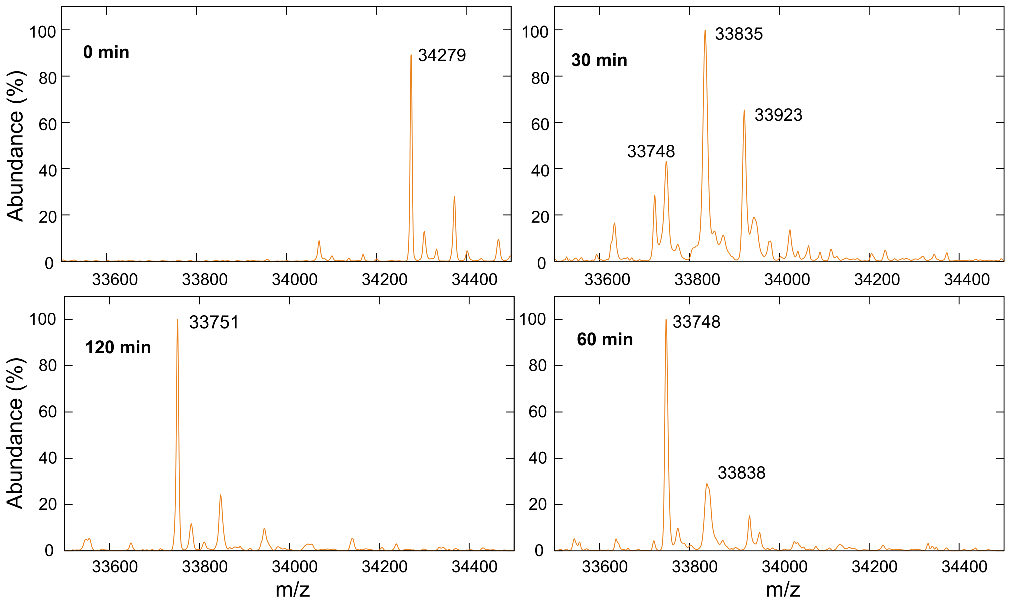

Figure S24: a) Scheme representing the reductively triggered immolation and hydrolysis of **anti-PD-L1 homodimer** with intermediate states illustrated alongside calculated masses. b) The deconvoluted LC–MS spectra over a 120 min period. 0 min corresponds to the intact dimer prior to addition of TCEP. Complete immolation and hydrolysis was complete within 120 min (Calculated mass: 33750 Da).

(a)

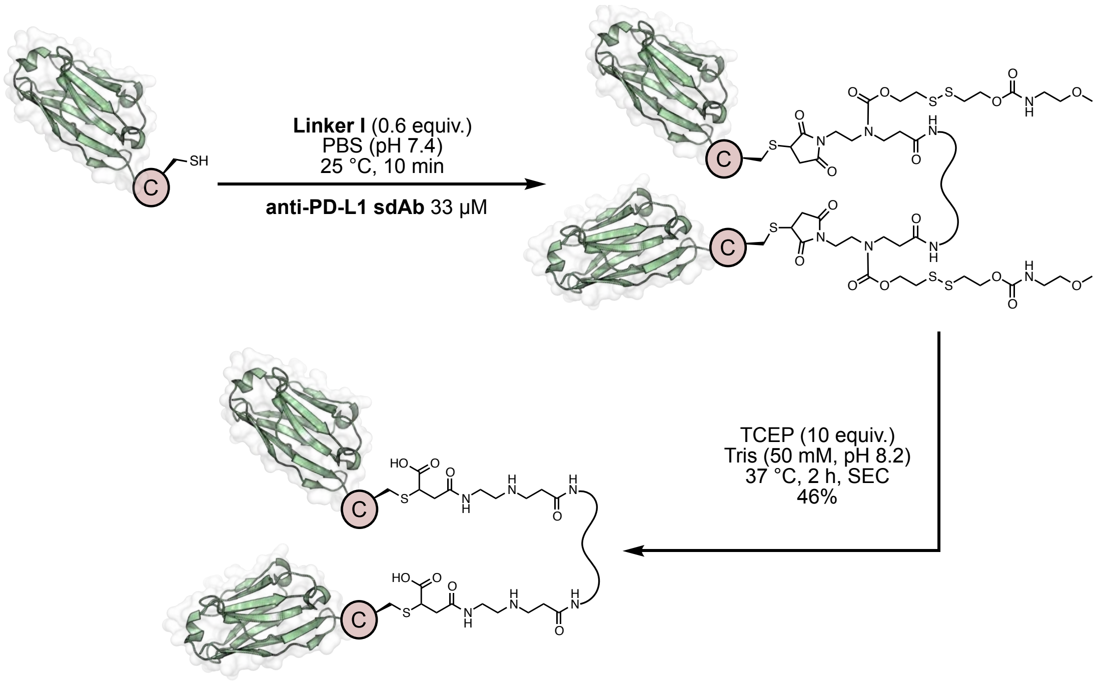

(c)

(b)

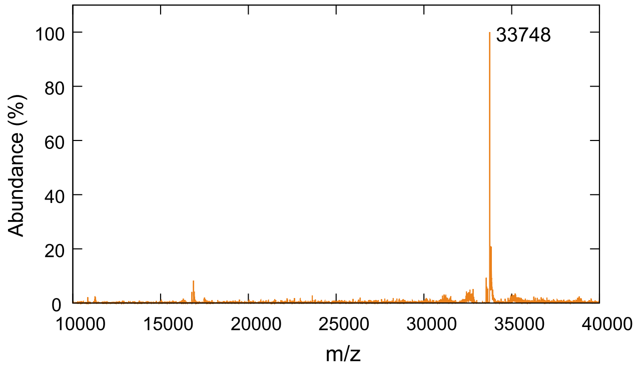

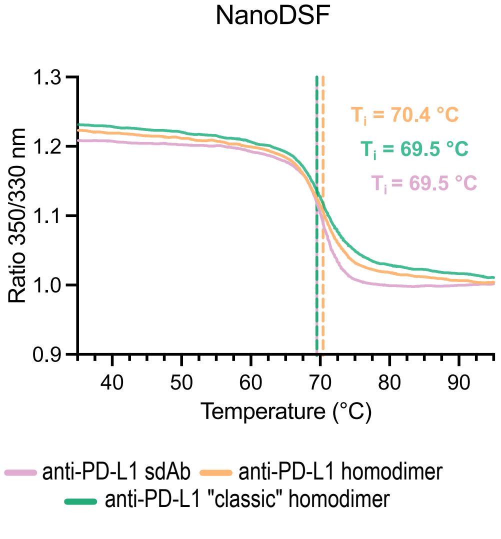

Figure S25: a) Scheme showing the reaction conditions for **anti-PD-L1 sdAb** homodimerisation and stabilisation with **Linker I**, via reductively triggered hydrolysis. b) Deconvoluted LC–MS spectrum of stabilised **anti-PD-L1 homodimer** after purification by SEC (Calculated mass: 33750 Da) (*continued...*)

...c) The T_i values and thermal denaturation profiles measured by nanoDSF of anti-PD-L1 Nb homodimers, derived from homobifunctional **Linker I** and a commercial bis-maleimide linker ('classic' homodimer) compared to parental Nb monomer. These confirmed that structural integrity of the Nb was maintained upon dimerisation and subsequent hydrolysis.

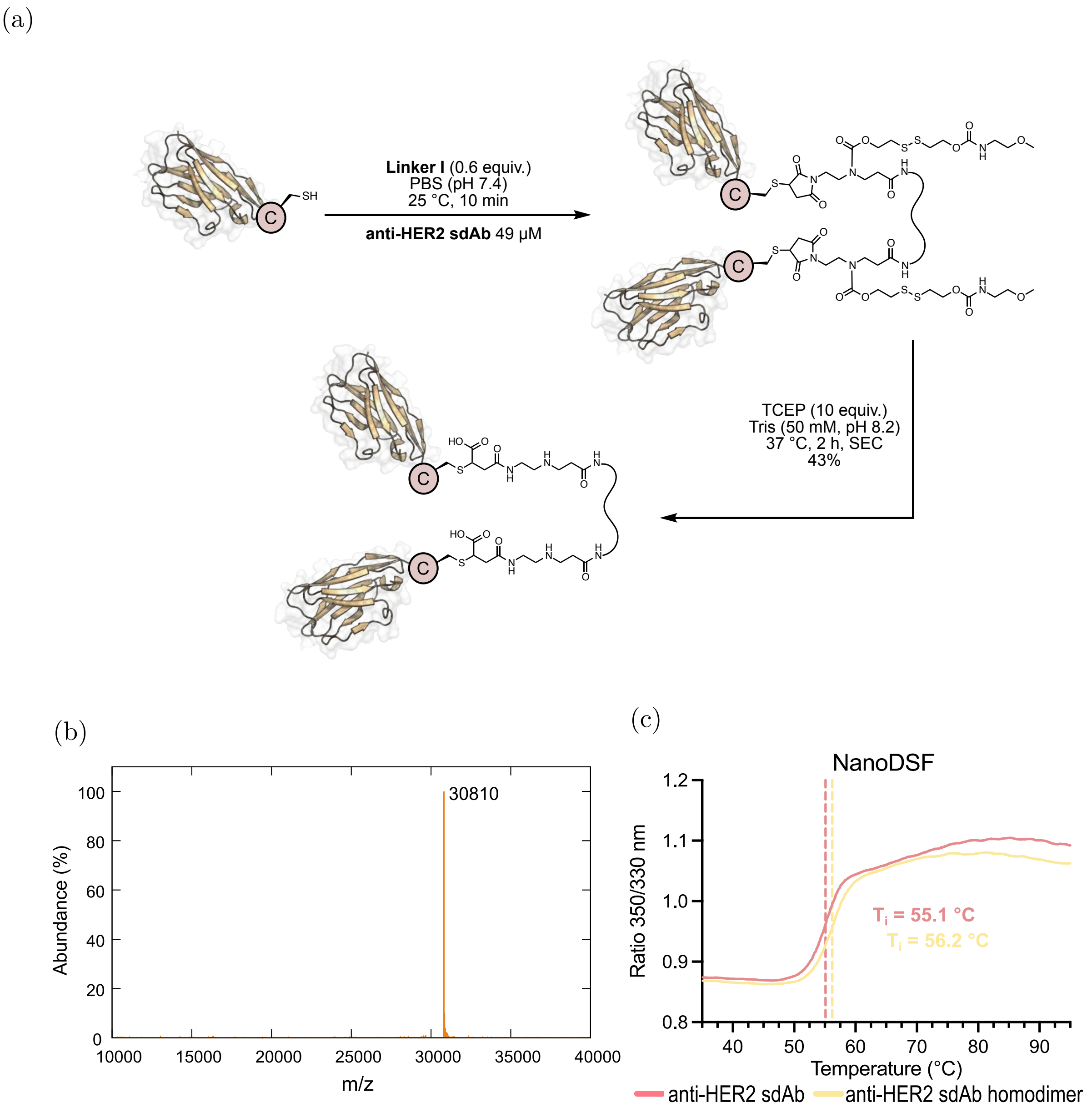

Figure S26: a) Reaction conditions for **anti-HER2 sdAb** homodimerisation with **Linker I**, followed by stabilisation via reductively triggered immolation and thiosuccinimide hydrolysis. b) Deconvoluted LC–MS analysis of stabilised **anti-HER2 sdAb homodimer** after preparative SEC (Calculated mass: 30810 Da). c) NanoDSF analysis of **anti-HER2 sdAb homodimer** compared to parental sdAb monomer confirmed that structural integrity of the sdAb was maintained upon dimerisation and subsequent hydrolysis with consistent T_i values.

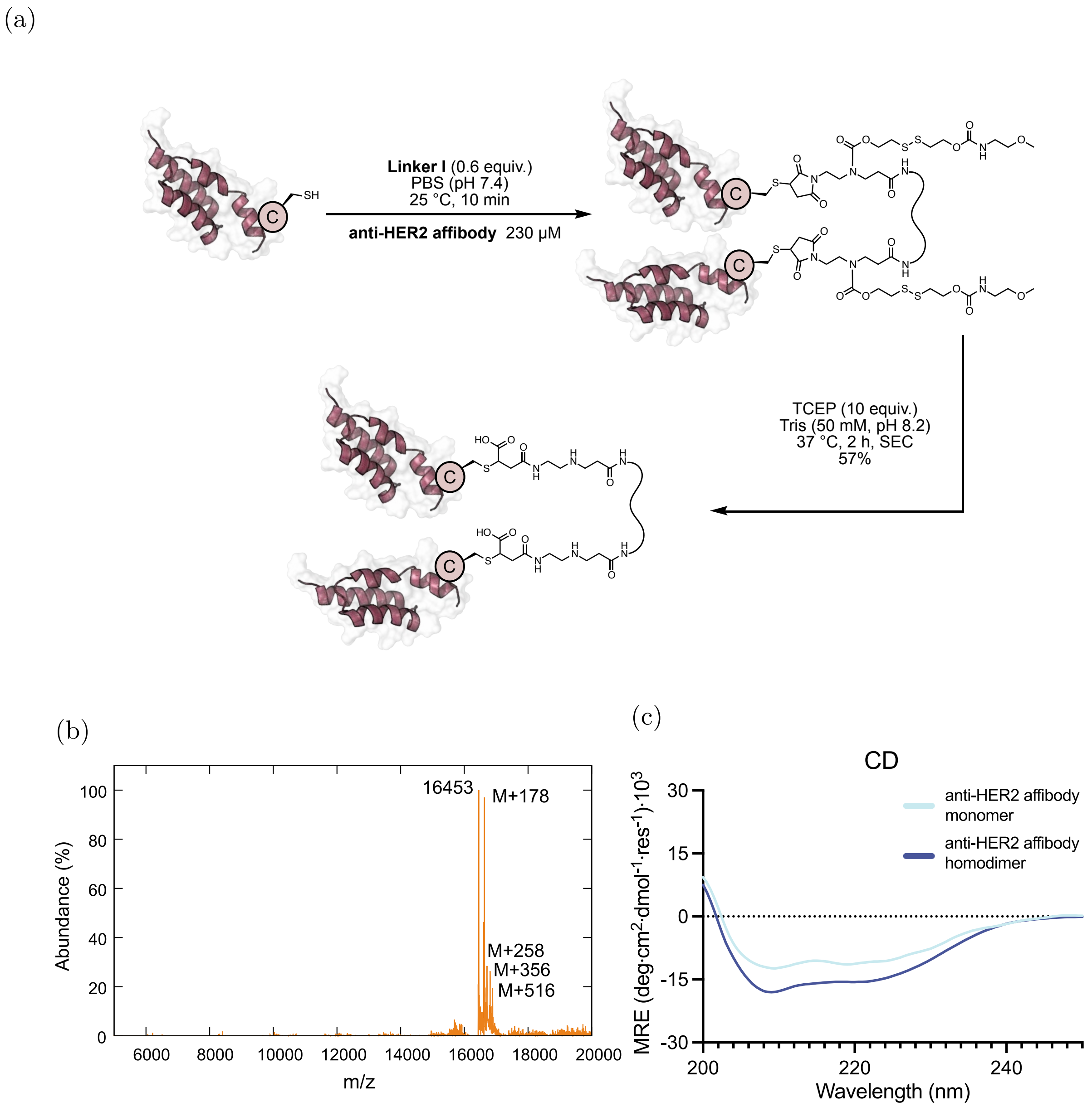

Figure S27: a) Reaction conditions for **anti-HER2 affibody** homodimerisation with **Linker I**, followed by stabilisation via reductively triggered immolation and thiosuccinimide hydrolysis. b) Deconvoluted LC–MS spectrum of stabilised **anti-HER2 affibody homodimer** after purification by SEC (Calculated mass: 16452 Da). M+178, M+258, M+356 and M+516 Da peaks correspond to partial α-N-6-phosphogluconoylation at the hexahistidine tag.[S9] c) CD analysis of the **anti-HER2 affibody homodimer** compared to its parental monomer confirmed that the mild nature of dimerisation strategy did not disrupt the α-helical secondary structure of the affibody domain.

Table S1: Homodimerisation reaction conditions and isolated yields after SEC.

Homodimer	Reaction conditions		Yield		% Yield
	Conc. (µM)	Vol. (µL)	Conc. (µM)	Vol. (µL)	
**anti-PD-L1 sdAb**	33.2	3370	8.23	6240	46
**anti-PD-L1 sdAb classic**	33.2	250	2.11	800	28
**anti-HER2 sdAb**	49	250	2.21	1200	43
**anti-HER2 affibody**	230	100	2.50	2630	57
**anti-HER2 sdAb/DBCO**	39	300	2.10	1000	36
**GFP Linker III**	109	100	2.47	1000	45

1.8.3 Optimised method for the assembly of heterodimeric protein–protein conjugates

A 250-480 µL HER2 sdAb in PBS (pH 7.4) (49 µM) was reduced for 1 h at 37 °C using 10 equivalents of TCEP. Excess TCEP was removed using Pierce^{TM} strong cation exchange mini spin columns as described in the homodimerisation protocol, and the protein eluted directly into bis-maleimide linker (20 equivalents). After 10 min, the reaction was purified by size exclusion chromatography using a 10/300 Superdex Increase 75 GL column (Cytiva, Little Chalfont, UK) and PBS (pH 7.4) as an elution buffer to remove any excess linker and disulfide dimerised protein. The protein–linker conjugate was added to a solution of a second cysteine-tagged protein (1.1 equivalents) and left to react for 30 min at room temperature. After 30 min, the pH of the mixture was raised to pH 8.2 by diluting 9:1 with Tris-HCl (pH 8.2, 500 mM). Finally, thio-succinimide self-immolation and subsequent hydrolysis was triggered by addition of 10 equivalents of TCEP and further incubation at 37 °C for a period of 1-2 h. After hydrolysis was complete (as determined by LC–MS analysis), the dimer was purified by size exclusion chromatography using a 10/300 Superdex Increase 75 GL column (Cytiva, Little Chalfont, UK) and PBS (pH 7.4) as an elution buffer. *Note: In case that the second protein required a reduction step, any excess of TCEP was removed prior to this step using a PD-10 column.*

(a)

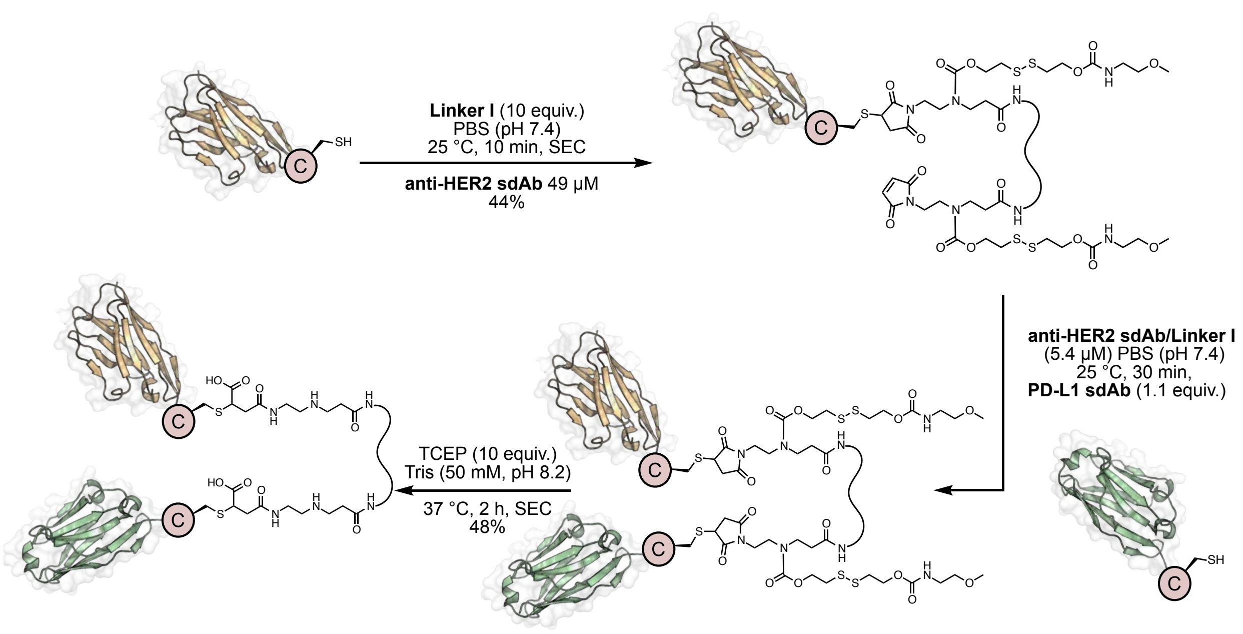
Linker I (10 equiv.)
PBS (pH 7.4)
25 °C, 10 min, SEC
anti-HER2 sdAb 49 µM
44%
anti-HER2 sdAb/Linker I
(5.4 µM) PBS (pH 7.4)
25 °C, 30 min,
PD-L1 sdAb (1.1 equiv.)
TCEP (10 equiv.)
Tris (50 mM, pH 8.2)
37 °C, 2 h, SEC
48%

(b)

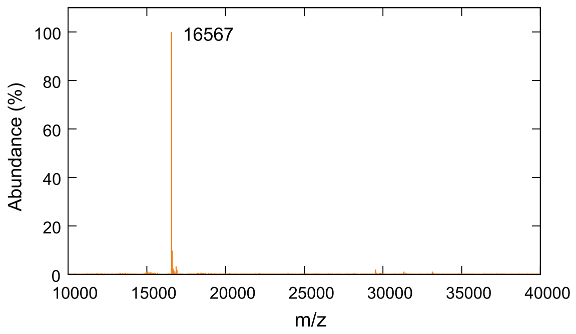
16567
Abundance (%)
m/z

(c)

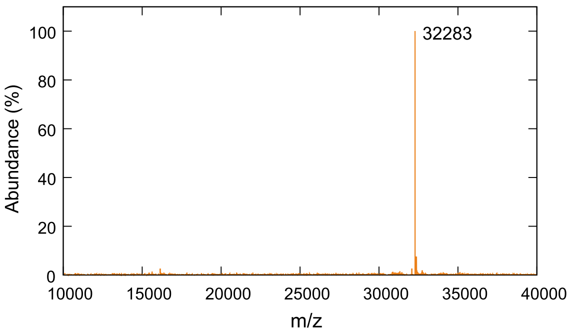
32283
Abundance (%)
m/z

(d)

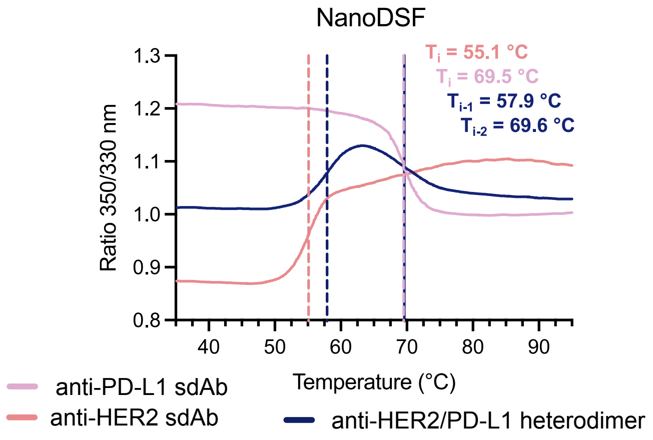
NanoDSF
Ti = 55.1 °C
Ti = 69.5 °C
Ti-1 = 57.9 °C
Ti-2 = 69.6 °C
Ratio 350/330 nm
Temperature (°C)
anti-PD-L1 sdAb
anti-HER2 sdAb
anti-HER2/PD-L1 heterodimer

Figure S28: a) Reaction conditions for **anti-HER2 sdAb** modification to produce **anti-HER2 sdAb/Linker I** monomer, proceeded by heterodimerisation upon incubation with **anti-PD-L1 sdAb** to produce **anti-HER2/PD-L1 heterodimer** and subsequent stabilisation by reductively triggered immolation and thiosuccinimide hydrolysis. Deconvoluted LC–MS spectra of b) **anti-HER2 sdAb** monomer functionalised with excess **Linker I** (Calculated mass: 16568 Da) and c) stabilised **anti-HER2/PD-L1 heterodimer** after purification by SEC (Calculated mass: 32281 Da). d) NanoDSF analysis of HER2–PD-L1 heterodimer, compared to parental sdAb monomers confirmed that structural integrity of the sdAb was maintained upon dimerisation and subsequent hydrolysis. The heterodimer exhibits two separate inflection points as it is the combination of monomers with distinct denaturation profiles.

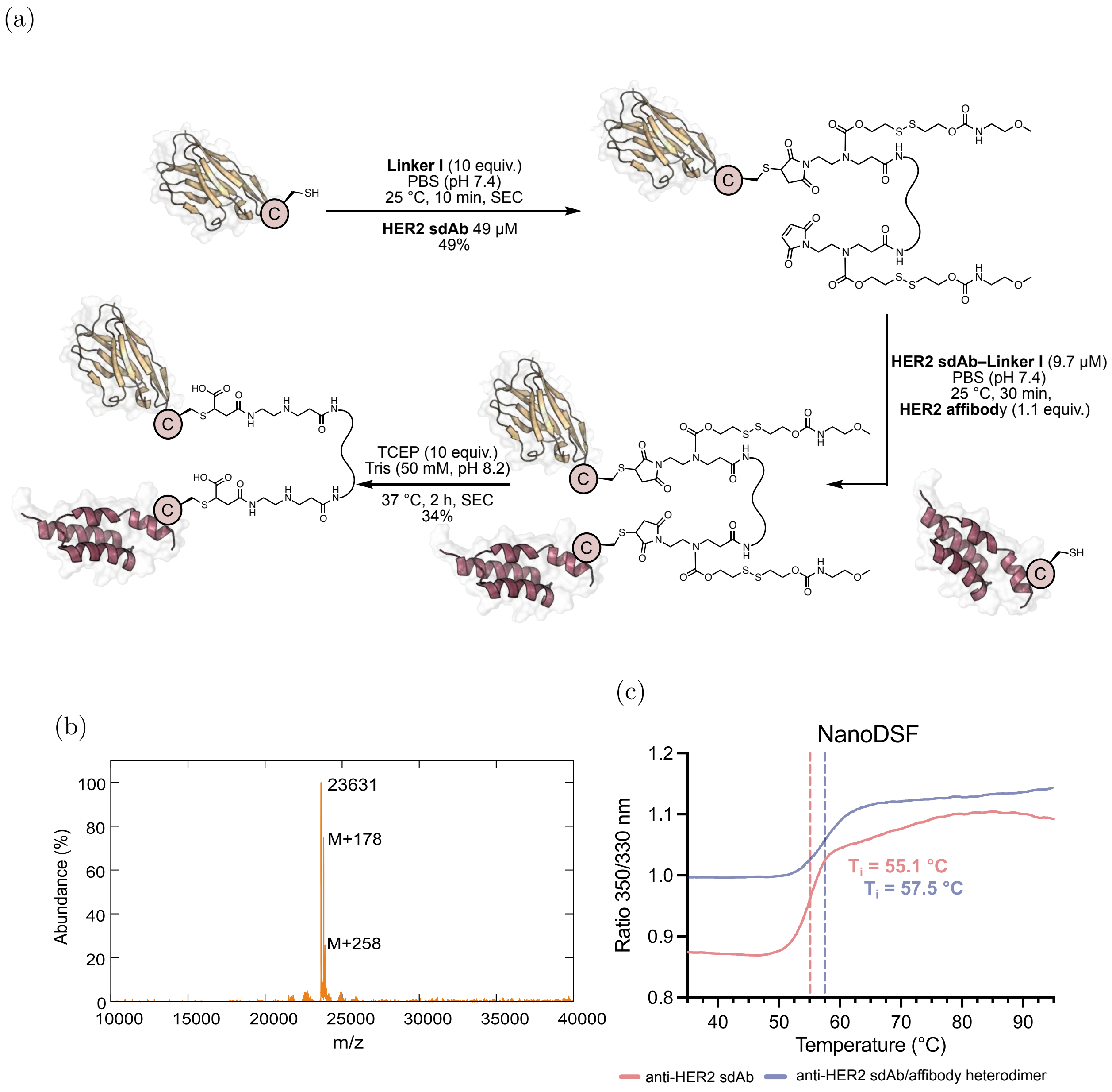

Figure S29: a) Reaction conditions for **anti-HER2 sdAb** modification to produce **anti-HER2 sdAb/Linker I** monomer, proceeded by heterodimerisation upon incubation with **anti-HER2 affibody** to produce **anti-HER2 biparatopic heterodimer** and subsequent stabilisation by reductively triggered immolation and thiosuccinimide hydrolysis. b) Deconvoluted LC–MS spectrum of stabilised **anti-HER2 biparatopic heterodimer** after preparative SEC (Calculated mass: 23631 Da) (*continued...*)

... c) NanoDSF analysis of **anti-HER2 sdAb/affibody** heterodimer compared to parental sdAb monomer confirmed that structural integrity of the sdAb was maintained upon dimerisation and subsequent hydrolysis with consistent T_i values. The affibody did not display a clear inflection point to determine stability, however, the upwards shift in the **anti-HER2 biparatopic heterodimer** profile was attributed to a greater 350/310 nm ratio of the affibody domain.

Table S2: Heterodimerisation reaction conditions and isolated yields after SEC.

Heterodimer	Reaction conditions		Yield		% Yield
	Conc. (µM)	Vol. (µL)	Conc. (µM)	Vol. (µL)	
**anti-HER2/PD-L1**					
**anti-HER2 sdAb/Linker I**	49	250	5.39	1000	44
Dimerisation	5.39	1000	2.26	1200	48
Overall yield	-	-	-	-	21
**anti-HER2 sdAb/affibody**					
**anti-HER2 sdAb/Linker I**	49	480	9.67	1200	49
Dimerisation	9.67	1200	2.47	1600	34
Overall yield	-	-	-	-	17

1.8.4 General method for cysteine capping with *N*-methyl maleimide

For BLI studies, to prevent dimerisation of proteins through their exposed cysteine residues, the monomers were capped prior to use with *N*-methyl maleimide. 200-500 μL of protein solution in PBS (concentration ranging from 33-230 µM) was reduced for 1 h at 37 °C using 10 equivalents of TCEP. After checking completion of the reduction step by LC–MS, the reaction was brought to room temperature and 10 equivalents of *N*-methyl maleimide were added. The reaction was incubated for a further 5 min. After 5 min, the reaction progress was assessed by LC–MS analysis, the capped monomers were purified by size exclusion chromatography using a 10/300 Superdex Increase 75 GL column (Cytiva, Little Chalfont, UK) and PBS (pH 7.4) as an elution buffer (Section 3.5).

1.8.5 General method for cysteine modification with Alexa Fluor 488 maleimide

200-500 μL of protein solution in PBS (concentration ranging from 49-230 µM) was reduced for 1 h at 37 °C using 10 equivalents of TCEP. After checking completion of the reduction

step by LC–MS, the reaction was buffer exchanged into PBS (pH 7.4) using Zeba^{TM} spin desalting columns to remove excess TCEP and 4 equivalents of Alexa Fluor 488 C_5 maleimide (Invitrogen, catalogue number: A10254) (10 equivalents, from a 5 mM stock in DMF) were added. The reaction was incubated for a further 5 min at room temperature. After 5 min, the reaction progress was assessed by LC–MS analysis, the labelled proteins were purified by size exclusion chromatography using a 10/300 Superdex Increase 75 GL column (Cytiva, Little Chalfont, UK) and PBS (pH 7.4) as an elution buffer.

1.8.6 Thiomab modification with Alexa Fluor 488 maleimide

To 42.7 μL of Thiomab in storage buffer (11.7 mg/mL), Tris-HCl (1 M, pH 8.0, 3.3 μL) was added to give a final Tris-HCl concentration of 75 mM. Alexa Fluor 488 C_5 maleimide (Invitrogen, catalogue number: A10254) (3 equivalents, 2 μL, from a 5 mM stock in DMF) were added and the final DMF concentration was brought to 10% upon addition of pure DMF (3 μL). After 20 min, the reaction progress was assessed by LC–MS analysis, free Alexa Fluor 488 was removed by UF/DF against PBS (pH 7.4) five times, followed by a further desalting step into PBS (pH 7.4) using Zeba^{TM} spin desalting columns.

Note: [a] To analyse the Thiomab by LC–MS, full reduction into heavy chain (HC) and light chain (LC) needed to be complete. To achieve this, 1 μL of the reaction mixture was diluted with 8 μL of PBS and DTT (1 μL, 100 mM stock) was added and the sample was reduced at 37 °C for 20 min. [b] The approximate molar concentration of the Thiomab was 65 μM.

1.8.7 General method for CuAAC labelling with Alexa Fluor 488

To 210 µL solution containing pure protein (1 equivalent) in PBS at a concentration ranging from 1-25 µM and Alexa Fluor 488 azide bis(triethylammonium salt), (Invitrogen, catalogue number: A10266) (5 equivalents, from a 5 mM stock in DMF), 60 µL of a premixed solution of 10 mM $CuSO_4.5H_2O$ and 40 mM THPTA (30 µL) was added, to achieve a final concentration of 1 mM Cu^{2+} and 4 mM THPTA, followed by 30 µL of a freshly prepared solution of ascorbic

acid at 100 mM (10 mM final concentration). The reaction was heated at 37 °C. Reaction completion was verified after 20 min by LC–MS. The reaction was quenched upon addition of the copper chelator bathocuproinedisulfonic acid disodium salt hydrate (Sigma-Aldrich, catalogue number B1125) (24 μL, 50 mm stock in PBS, final concentration 4 mM). The mixture was subsequently buffer exchanged into PBS (pH 7.4) using Zeba^{TM} spin desalting columns twice to ensure complete buffer exchange.

1.8.8 Thiol-based trimerisation procedure

Dimerisation of **anti-HER2 sdAb** with **Linker II** was carried out as described in the optimised method for assembly of homodimers and the resulting homodimer was SEC purified prior to immolation. Separately, reduced **anti-CD3 sdAb** (9.8 μM, 2.2 mL) in PBS (10 mM, pH 7.4) was reacted with **Linker III** (10 equivalents) and left to incubate at 25 ° for 10 min prior SEC purification. To pure **anti-HER2 sdAb homodimer** bearing a thiol handle (8.4 μM, 350 μL) desalted **anti-CD3/Linker III** was added (4.5 μM, 784 μL, 1.2 equivalents) and the reaction was left to incubate for 1 h at 25 °C, at which point the reaction was deemed to not be proceeding further, as determined by LC–MS. At this point, the pH of the mixture was raised by diluting 9:1 with Tris-HCl (pH 8.2, 500 mM) to achieve Tris-HCl (pH 8.2, 50 mM). Finally, thio-succinimide self-immolation and subsequent hydrolysis was triggered by addition of 10 equivalents of TCEP and further incubation at 37 °C for 2 h. The reaction was analysed by SDS–PAGE and LC–MS.

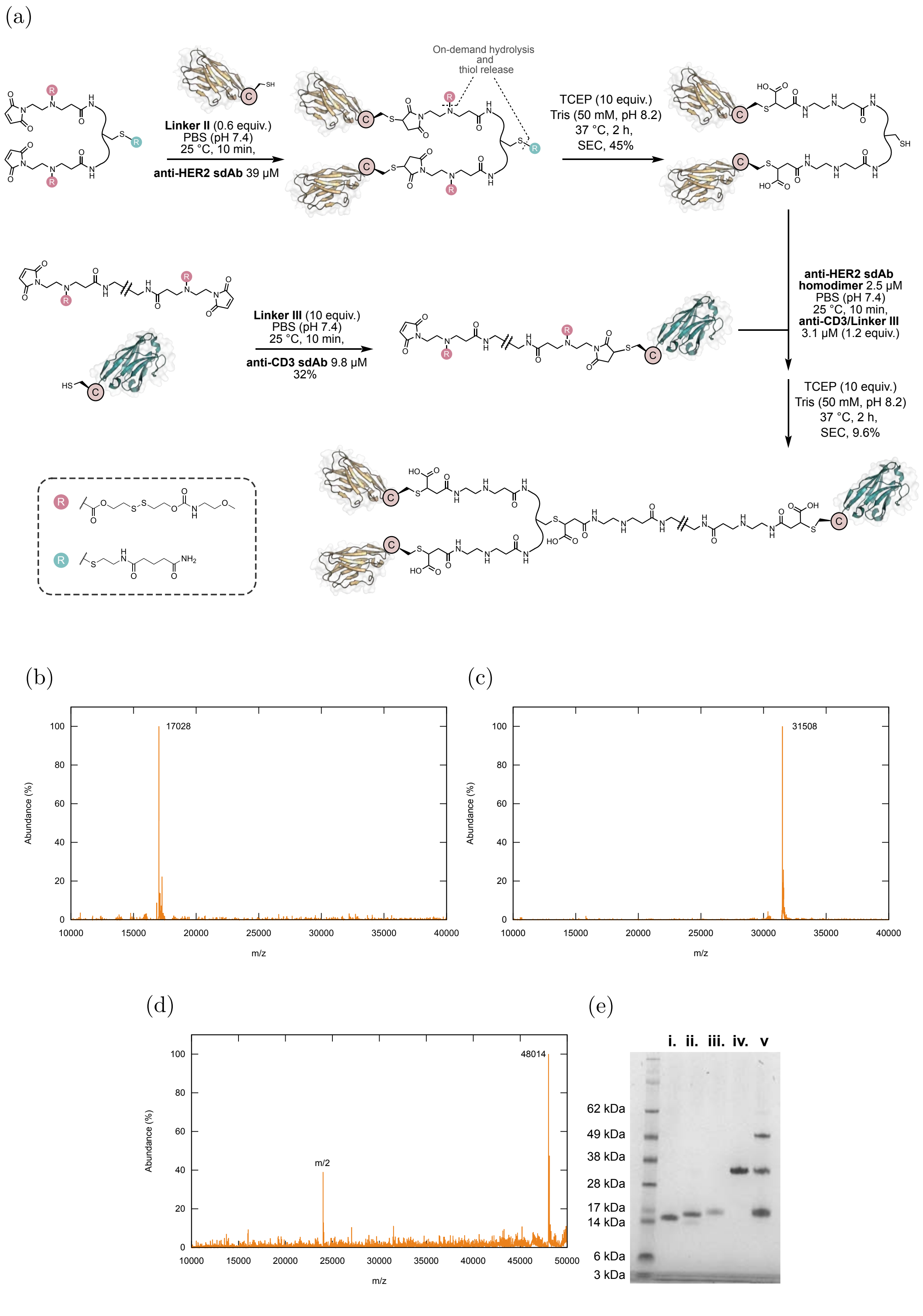

Figure S30: a) Reaction conditions for **anti-HER2 sdAb** homodimerisation (*continued...*)

...with thiol containing **Linker II**, followed by trimerisation via thiol-maleimide with **anti-CD3/Linker III** presenting free maleimide functionality, followed by reductively triggered stabilisation. b) Deconvoluted LC–MS spectrum of **anti-CD3/Linker III** monomer after preparative SEC (Calculated mass: 17029 Da. c) Deconvoluted LC–MS spectrum of **anti-HER2 sdAb homodimer** after preparative SEC (Calculated mass: 31513 Da). d) Deconvoluted LC–MS spectrum of **anti-HER2/CD3 trimer** after hydrolytic stabilisation and SEC purification (Calculated mass: 48017 Da). e) Reducing SDS-PAGE gel of; **i. anti-HER2 sdAb** monomer, **ii. anti-CD3 sdAb** monomer, **iii. anti-CD3/Linker III** monomer, **iv. anti-HER2 sdAb homodimer**, and **v.** trimerisation reaction.

1.8.9 Thiol-based tetramerisation procedure

Dimerisation of **anti-PD-L1 sdAb** with **Linker II** was carried out as described in the optimised method for assembly of homodimers and the resulting homodimer was immolated prior SEC purification. Separately, SEC purified **anti-HER2 homodimer** (8.4 μM, 750 μL), previously obtained during the synthesis of thiol-based **anti-HER2/CD3 trimer**, was reacted with **Linker III** (10 equivalents) and left to incubate at 25 ° for 10 min. Following, excess **Linker III** was removed using Pierce^{TM} strong cation exchange mini spin columns following the steps described in the homodimerization protocol. Subsequently, pure **anti-PD-L1 homodimer** bearing a thiol handle (8.2 μM, 156 μL, 1.0 equivalents) was incubated with maleimide containing **anti-HER2 homodimer–Linker III** (6.9 μM, 185 μL, 1.0 equivalents) and the reaction was left to incubate for 1 h at 25 °C. At this point, the pH of the mixture was raised by diluting 9:1 with Tris-HCl (pH 8.2, 500 mM) to achieve Tris-HCl (pH 8.2, 50 mM). Finally, thio-succinimide self-immolation and subsequent hydrolysis was triggered by addition of 10 equivalents of TCEP and further incubation at 37 °C for 2 h. SDS–PAGE analysis of the reaction confirmed the conversion towards the **anti-HER2/PD-L1 tetramer** product.

(a)

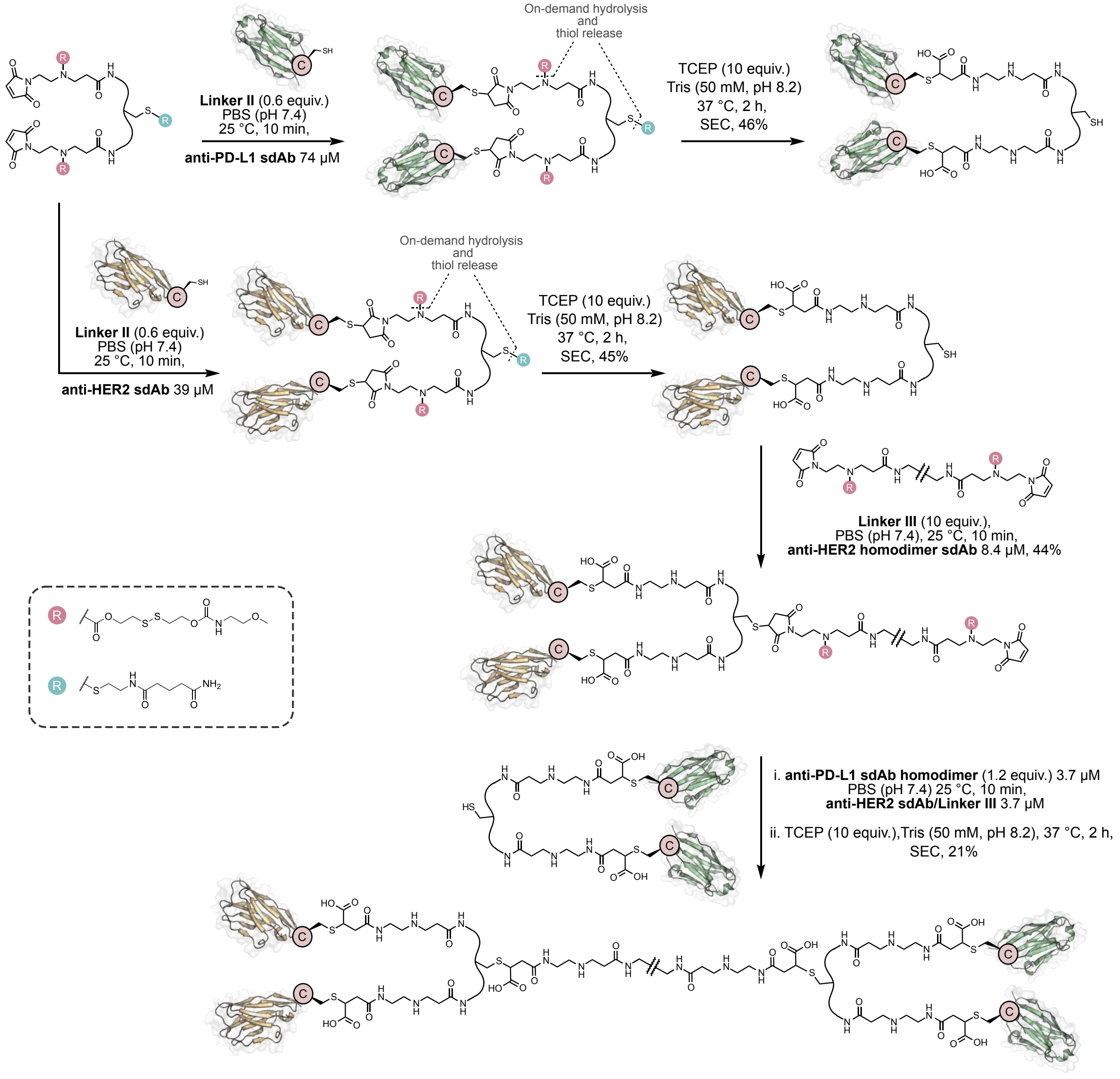

On-demand hydrolysis
and
thiol release
Linker II (0.6 equiv.)
PBS (pH 7.4)
25 °C, 10 min,
anti-PD-L1 sdAb 74 μM
TCEP (10 equiv.)
Tris (50 mM, pH 8.2)
37 °C, 2 h,
SEC, 46%
anti-HER2 sdAb 39 μM
SEC, 45%
Linker III (10 equiv.),
PBS (pH 7.4), 25 °C, 10 min,
anti-HER2 homodimer sdAb 8.4 μM, 44%
i. anti-PD-L1 sdAb homodimer (1.2 equiv.) 3.7 μM
PBS (pH 7.4) 25 °C, 10 min,
anti-HER2 sdAb/Linker III 3.7 μM
ii. TCEP (10 equiv.),Tris (50 mM, pH 8.2), 37 °C, 2 h,
SEC, 21%

(b)

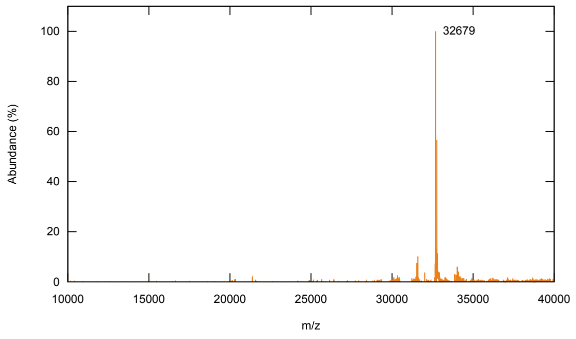

32679
Abundance (%)
m/z

(c)

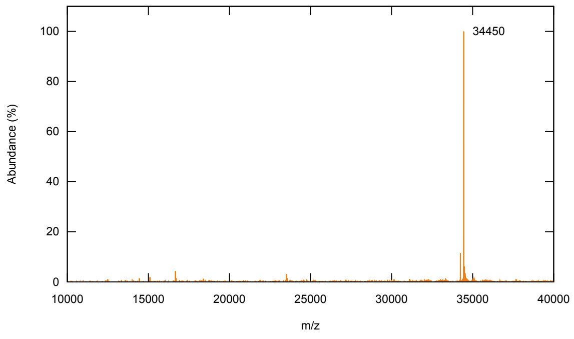

34450
Abundance (%)
m/z

(d)

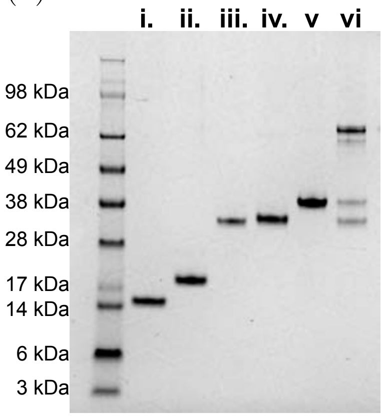

Figure S31: a) Reaction conditions for **anti-PD-L1 sdAb** homodimerisation with thiol containing **Linker II**, followed by tetramerisation via thiol-maleimide with maleimide containing **anti-HER2 sdAb homodimer–Linker III**, and final reductively triggered stabilisation. b) Deconvoluted LC–MS spectrum of **anti-HER2 sdAb homodimer–Linker III** after cation exchange purification (Calculated mass: 32684 Da. c) Deconvoluted LC–MS spectrum of **anti-PD-L1 homodimer** after preparative SEC (Calculated mass: 34455 Da). d) Reducing SDS-PAGE gel of; **i. anti-HER2 sdAb** monomer, **ii. anti-PD-L1 sdAb** monomer, **iii. anti-HER2 sdAb homodimer** , **iv. anti-HER2 sdAb homodimer–Linker II**, **v. anti-PD-L1 homodimer**, and **vi.** tetramerisation reaction. *Note: LC-MS of crude tetramer was not obtained, this was attributed to poor ionisation*

Table S3: Thiol-trimerization and tetramerization reaction conditions and isolated yields after SEC.

Construct	Reaction conditions		Yield		% Yield
	Conc. (µM)	Vol. (µL)	Conc. (µM)	Vol. (µL)	
**Trimer**					
**anti-HER2 sdAb homodimer**	31	1800	8.4	1500	45
**CD3/Linker III**	9.8	2200	4.5	1550	32
HER2/CD3 Trimerisation	8.4	350	0.45	600	9.6
**Tetramer**					
**HER2 sdAb homodimer** plus **Linker III**	8.4	750	6.9	400	44^{*a}
PD-L1 dimerization	74	900	8.16	1260	31
HER2/PD-L1 Tetramerization	6.93	185			56^{*b}

^{*a} refers to isolated yield upon purification via Pierce^{TM} strong cation exchange mini spin columns;^{*b} refers to reaction conversion as inferred by SDS-PAGE band densitometry using Fiji (Fiji ImageJ2 (version 2.14.0/1.54f) version 2.14.0/1.54f).[S1]

1.8.10 DBCO-based trimerisation procedure

Dimerisation of **anti-HER2 sdAb** with **Linker V** was carried out as described in the optimised method for assembly of homodimers and the resulting homodimer was SEC purified prior to immolation. Separately, **anti-CD3 sdAb** (32 μM, 500μL) in Tris-HCl (50 mM, pH 8.0) was reduced with TCEP (5 equivalents) and bromoacetamido-dPEG$_{11}$-azide was added (15 equivalents) and left to incubate at 30 ° for 4 h prior using Zeba^{TM} spin desalting columns to remove the excess of small molecules using PBS (pH 7.4) as an elution buffer. To pure **anti-HER2 sdAb homodimer** bearing a DBCO handle (6 μM, 100 μL) desalted **anti-CD3 sdAb** bearing an azide was added (3 equivalents) and the reaction was left to incubate for 48 h at 37 °C, at which point the reaction was deemed to not be proceeding further, as determined by LC–MS. At this point, the pH of the mixture was raised by diluting 9:1 with Tris-HCl (pH 8.2, 500 mM) to achieve Tris-HCl (pH 8.2, 50 mM). Finally, thio-succinimide self-immolation and subsequent hydrolysis was triggered by addition of 10 equivalents of TCEP and further incubation at 37 °C for 2 h. The reaction was analysed

by SDS–PAGE and LC–MS.

(a)

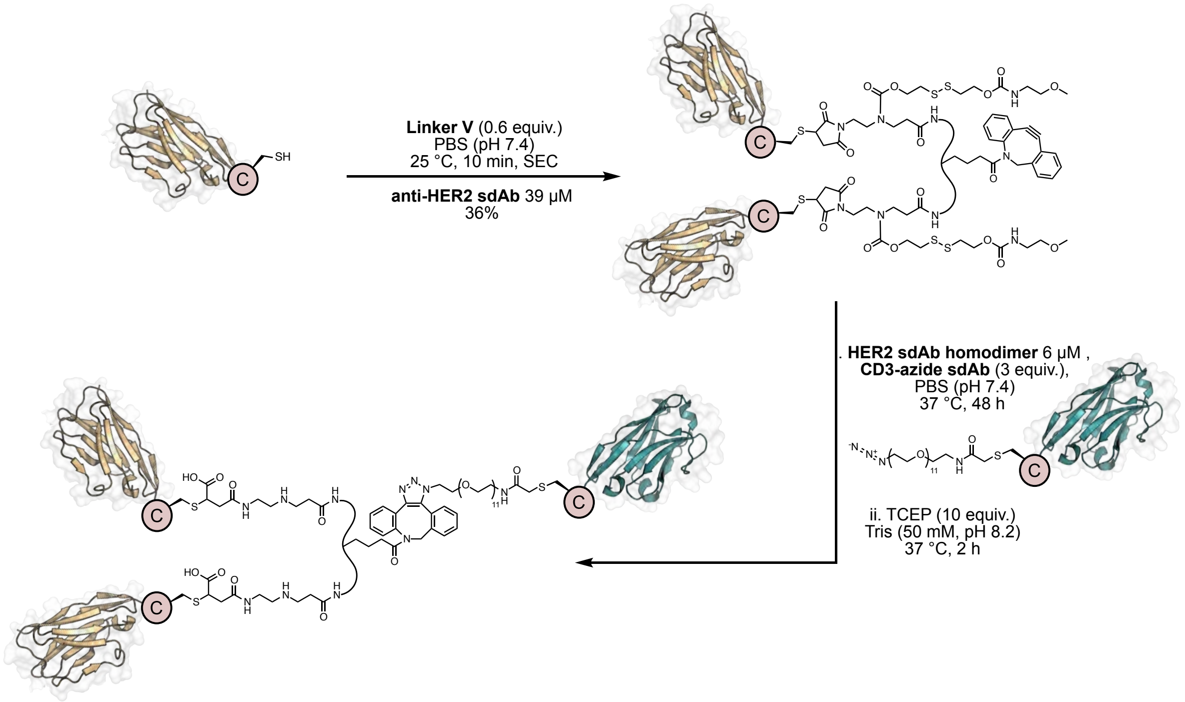

(b)

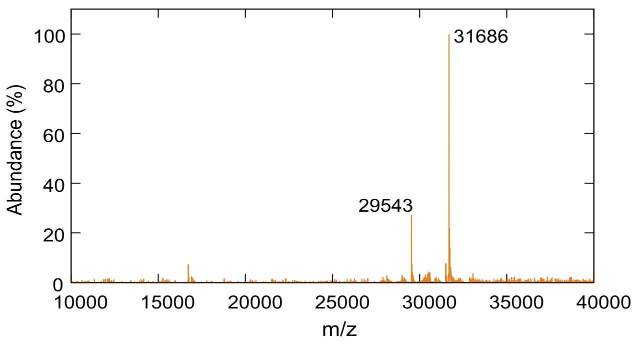

(c)

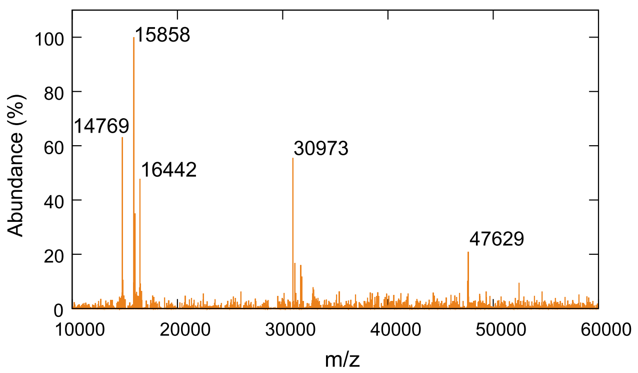

(d)

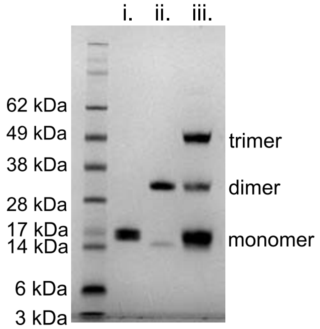

Figure S32: a) Reaction conditions for **anti-HER2 sdAb** homodimerisation with DBCO containing **Linker V**, followed by trimerisation via SPAAC with **anti-CD3 sdAb** presenting an azide functionality, followed by reductively triggered stabilisation. b) Deconvoluted LC–MS spectrum of **anti-HER2 sdAb homodimer** after preparative SEC (Calculated mass: 31685 Da). The peak at 29543 Da corresponds to intermolecular disulfide mediated dimerisation of the **anti-HER2 sdAb**, as SEC was carried out in the absence of reducing agent. c) Deconvoluted LC–MS spectrum of **anti-HER2/CD3 trimer** after hydrolytic stabilisation (Calculated mass: 47629 Da). Additional peaks: 14769 Da: reduced intermolecular disulfide-dimerised **anti-HER2 sdAb**, 15858 Da: reduced intermolecular disulfide-dimerised **anti-CD3 sdAb** (Figure S72, 16442 Da: TCEP reduced **anti-CD3 sdAb** azide conjugate (-N_2) (Figure S72), and 30973 Da: hydrolytically decomposed DBCO containing dimer. d) Reducing SDS-PAGE gel of; **i. anti-CD3 sdAb/azide** monomer, **ii. HER2 sdAb homodimer/DBCO** and **iii.** trimerisation reaction.

Although the DBCO approach allowed the production of trimers, its efficiency was impeded by the relatively slow reaction between DBCO and azides with a second order rate constant of $k_2 = 10^{-2} - 1\ \mathrm{M^{-1}s^{-1}}$.[S10] For sufficient trimerisation to take place, the reaction required 48 h at elevated temperature. Once again, this brings the protein–protein coupling problem and the requirement for transformations with high reaction velocity, when producing higher order protein–protein conjugates, into sharp focus,. These unfavourable bioconjugation conditions led to the formation of a side product in the form of **anti-HER2 sdAb homodimer** in which the DBCO moiety was hydrolysed at the amide bond, render-

ing it unreactive. This hydrolytic decomposition has previously been attributed to proton catalysed degradation of DBCO (Figure S33) and further highlights the requirement for an alternative chemistry to SPAAC when producing trimers.[S11]

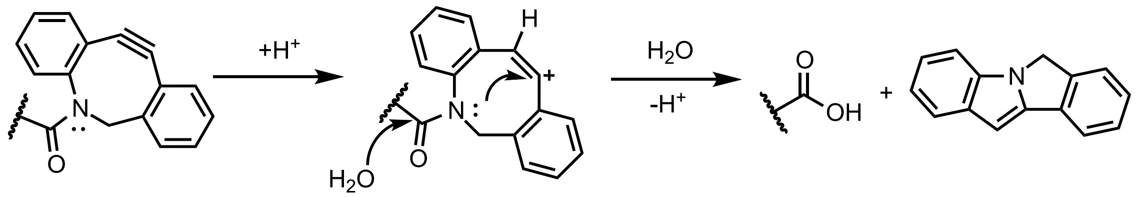

Figure S33: Scheme outlining the mechanism for proton catalysed hydrolytic degradation, rendering DBCO unreactive, as proposed by Janson and co-workers.[S11]

1.8.11 Anti-PD-L1 sdAb–Thiomab conjugation

To reduced **anti-PD-L1 sdAb** in PBS (pH 7.4, 1.38 mL, 74 μM) 76.6 μL of DMSO and a solution of **Linker IV** (76.6 μL, 20 mM stock in DMSO, 15 equivalents) were added (DMSO concentration 10% *v/v*). The reaction went to completion within 2 min as observed by LC–MS, and excess linker was removed using a PD-10 desalting column followed by concentration using an amicon 10 kDa MWCO spin filter and adjusted to 168 μM. To Thiomab (85.5 μL, 11.7 mg/mL) in storage buffer, Tris-HCl (1 M, pH 8.0, 15 μL) was added to give Thiomab in Tris-HCl (56 μM, pH 8.0). **Anti-PD-L1 sdAb** monomer modified with bis-maleimide linker was subsequently added (200 μL, 168 μM, 3 equivalents with respect to Thiomab cysteine residues). The reaction progress was analysed by LC–MS and deemed to have gone to completion within 20 min. Following completion, the reaction mixture was irradiated under UV light at 365 nm using an EvoluChem LED 365DX, 25 mW/cm^2 at a 1 cm distance for 2 min. Full immolation and hydrolysis was observed after 10 min at room temperature. The resulting bispecific conjugate was purified from excess **anti-PD-L1 sdAb** by UF/DF against PBS (pH 7.4) five times using an amicon 100 kDa MWCO spin filter to isolate (105 μL, 19.6 μM, 37%) with 64% modified LC as determined by SDS-PAGE band densitometry using Fiji (Fiji ImageJ2 (version 2.14.0/1.54f) version 2.14.0/1.54f).[S1]

(a)

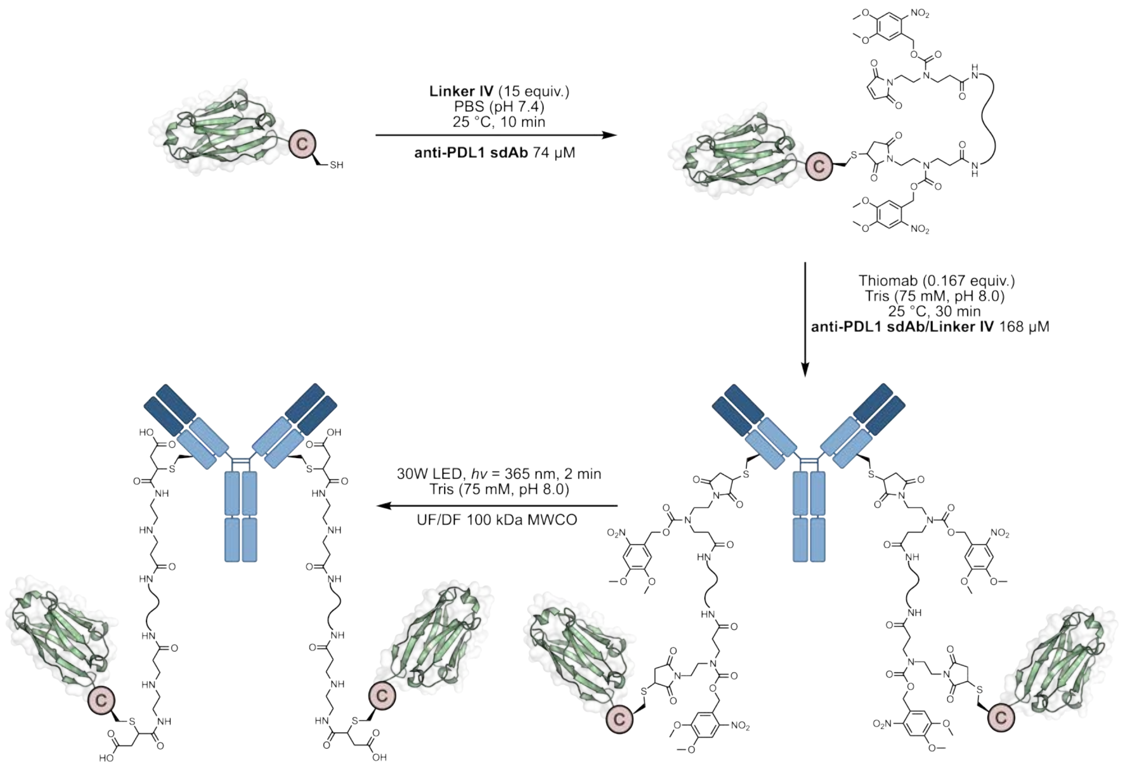

Linker IV (15 equiv.)
PBS (pH 7.4)
25 °C, 10 min
anti-PDL1 sdAb 74 μM
Thiomab (0.167 equiv.)
Tris (75 mM, pH 8.0)
25 °C, 30 min
anti-PDL1 sdAb/Linker IV 168 μM
30W LED, hv = 365 nm, 2 min
Tris (75 mM, pH 8.0)
UF/DF 100 kDa MWCO

(b)

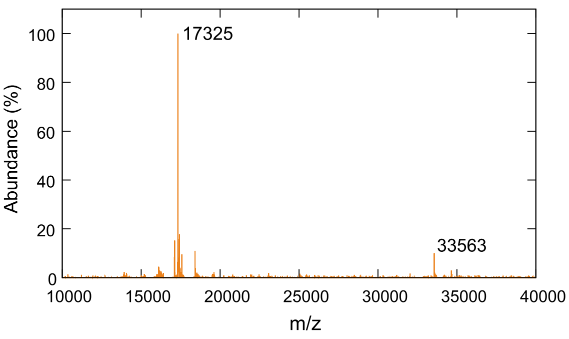

Abundance (%)
m/z
17325
33563

(c)

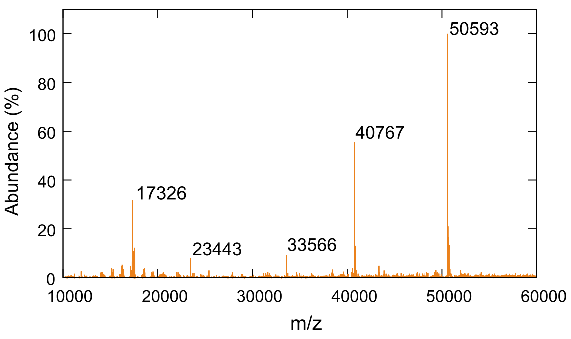

Abundance (%)
m/z
17326
23443
33566
40767
50593

(d)

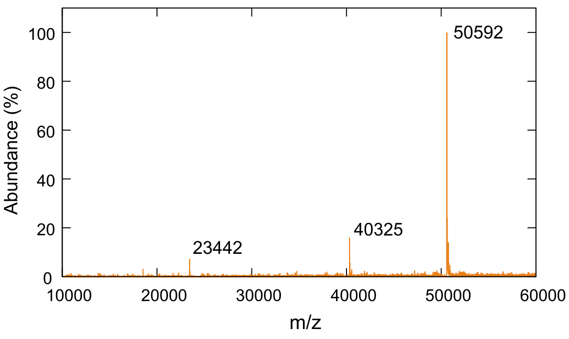

Abundance (%)
m/z
23442
40325
50592

(e)

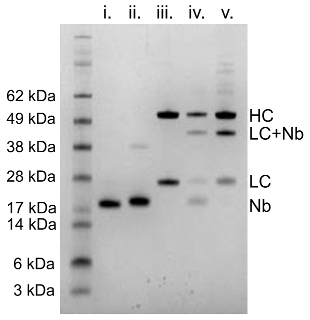

i.
ii.
iii.
iv.
v.
62 kDa
49 kDa
38 kDa
28 kDa
17 kDa
14 kDa
6 kDa
3 kDa
HC
LC+Nb
LC
Nb

Figure S34: a) Reaction conditions for the production of IgG–sdAb conjugate from **anti-PD-L1 sdAb** and Thiomab, and subsequent UV-triggered hydrolytic stabilisation. Deconvoluted LC–MS spectrum of b) **anti-PD-L1 sdAb/Linker IV** intermediate after desalting (Calculated mass: 17325 Da); c) **anti-PD-L1 sdAb** conjugated to the LC of Thiomab prior to UV-triggered hydrolytic stabilisation (Calculated masses: LC: 40767 Da, HC: 50595 Da). The peak at 17326 Da corresponds to **anti-PD-L1 sdAb-Linker IV** intermediate; 23443 Da to unmodified Thiomab LC; and 33566 Da to **anti-PD-L1 sdAb homodimer** formed via **Linker IV** and d) **anti-PD-L1 sdAb** conjugated to the LC of Thiomab after UV-triggered hydrolytic stabilisation (Calculated masses: LC: 40325 Da, HC: 50595 Da). The peak at 23442 Da corresponds to unmodified LC. e) Reducing SDS-PAGE gel of; **i. anti-PD-L1 sdAb**, **ii. anti-PD-L1 sdAb/Linker IV** conjugate, **iii.** unmodified Thiomab, **iv.** crude reaction mixture and **v.** the reaction mixture after purification by UF/DF.

1.9 Stability assays

1.9.1 Glutathione stability assay

Samples of anti-PD-L1 homodimers generated using **Linker I** and the commercial maleimide linker, 1,11-bis-maleimidotriethyleneglycol (Thermofisher, catalogue number: 22337) (95 µL, 2 µM, PBS pH 7.4) were prepared. 5 µL of a 40 mM glutathione (GSH) solution (6.14 mg GSH in 500 µL PBS, adjusted to pH 7.4) was added at room temperature and mixed thoroughly by pipetting up and down, resulting in a final GSH concentration of 2 mM. The resulting mixture was incubated at 37 °C. Time points were taken at 0, 1, 3, 5 and 7 days, and immediately flash frozen in liquid N_2. Samples were analysed by SDS–PAGE to observe thiol exchange of the linker over time and concomitant regeneration of monomeric species (Figure S35A). The total percentage of remaining dimer was quantified by densitometry using Fiji ImageJ2 (version 2.14.0/1.54f) software and plotted against time (Figure S35B).[S1]

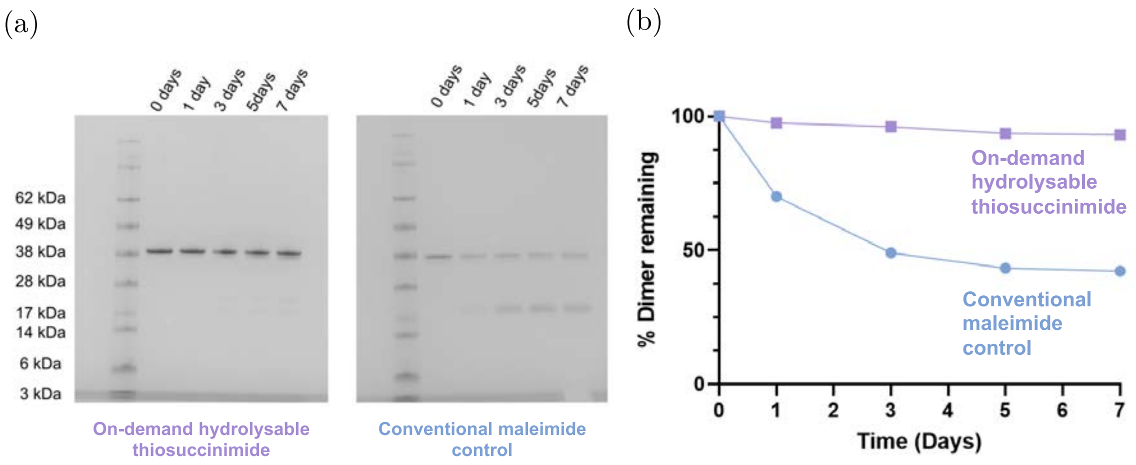

Figure S35: Stability analysis of anti-PD-L1 homodimers generated using **Linker I** and the commercial maleimide linker by SDS–PAGE under reducing conditions. The homodimers were incubated for 7 days in the presence of reduced GSH (2 mM) in PBS (pH 7.4) at 37 °C. a) SDS–PAGE gels, b) Percentage of remaining dimer plotted against time, as determined by band densitometry analysis of SDS-PAGE gels.

1.9.2 Human plasma stability assay

A sample of GFP homodimer generated using **Linker III** (200 µL, 2.5 µM, PBS pH 7.4) were prepared. 200 µL of human plasma (Sigma-Aldrich, Cat. H4522) was added at room temperature and mixed thoroughly by pipetting up and down, resulting in a final plasma concentration of 50%. The resulting mixture was incubated at 37 °C. Time points were taken at 0, 1, 2, 4 and 7 days, and immediately flash frozen in liquid N_2. Samples were analysed by SDS–PAGE under reducing conditions with a constant voltage of 125 V. Sample preparation was done without boiling to maintain integrity of GFP. The SDS–PAGE gel was visualised by blue LED epi-illumination source and a 532 nm/28 mm band pass filter (Alexa-488 settings) prior to staining and subsequently coomassie stain using a ChemiDoc Imaging System (Bio-Rad) (Figure S36).

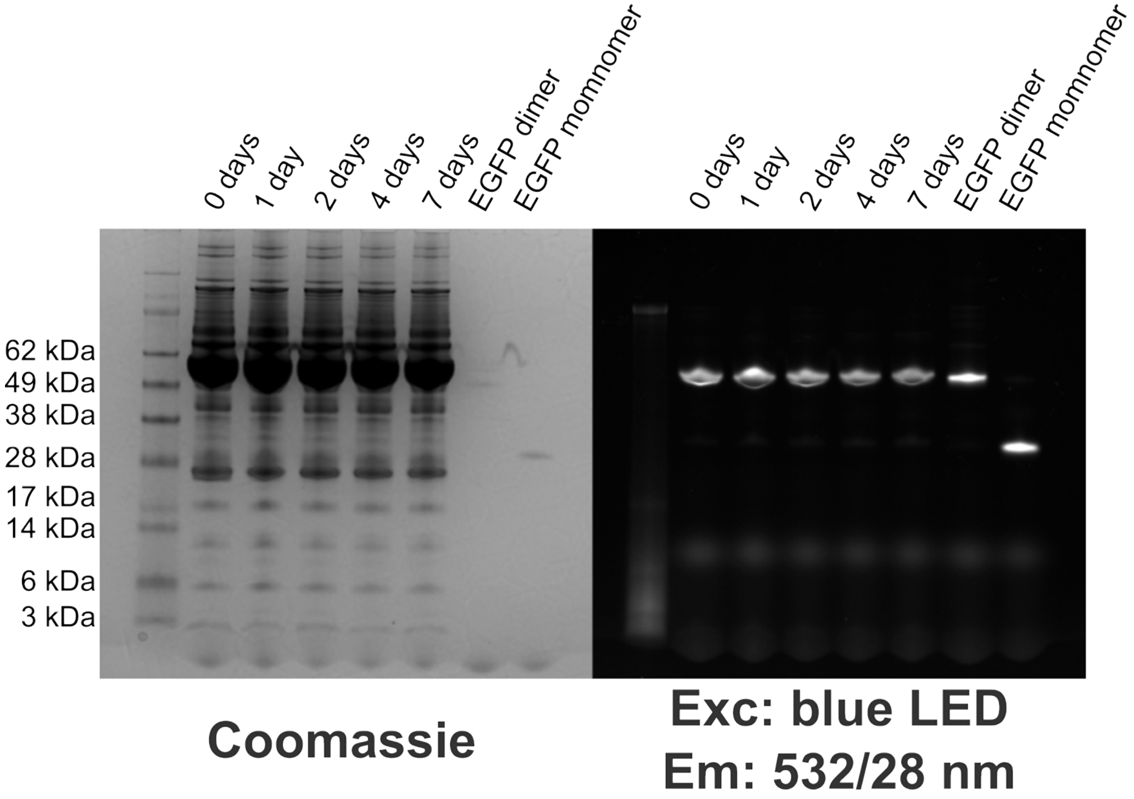

Figure S36: Plasma stability of **EGFP homodimer** as inferred by SDS–PAGE (under reducing conditions) analysis upon incubation with 50% human plasma at 37 °C. Left: coomassie stained gel and Right: 532 nm/28, blue Epi channel showing EGFP fluorescence

1.10 Biophysical characterisation

1.10.1 Circular dichroism

Far ultraviolet (UV) circular dichroism (CD) spectroscopy of all expressed proteins was carried out, measurements were recorded using a JASCO J-810 spectrophotometer equipped with a thermally controlled Peltier holder using a quartz cuvette with a 0.1 cm path length. Protein samples were diluted in PBS (pH 7.4) to approximately 6 µM and 3 µM protein for all monomers and the **anti-HER2 affibody homodimer**, respectively. CD spectra were obtained over a wavelength range of 200 to 250 nm at 25 °C with a bandwidth of 1 nm, a data pitch of 0.025 nm, a scanning speed of 50 nm/min and a response time of 4 s. All measurements were obtained as θ (mdeg) and the buffer spectrum was systematically subtracted. All data was subsequently normalised to mean residue ellipticity (MRE)

(Equation 1) in (deg·cm^{-2}·dmol^{-1}·res^{-1}) to generate CD plots. Spectra were generated by averaging the data acquired from 30 scans for each sample.

$$MRE = \frac{\theta(mdeg)}{10 \cdot C(M) \cdot l(cm) \cdot \#_{residues}} \qquad (1)$$

Note: It was not possible to measure affibody T_i by nanoDSF and for this reason, a CD spectrum for the **anti-HER2 affibody homodimer** was acquired to confirm structural integrity of the chemically modified protein. The **anti-PD-L1 homodimer** had its CD spectrum acquired to confirm the chemical linking strategy did not disrupt secondary structure of sdAb scaffolds. All other sdAb containing dimers had stability after dimerisation confirmed by nanoDSF thermal denaturation.

1.10.2 NanoDSF thermal denaturation assays

Expressed and subsequently dimerised proteins structural integrity were assessed using Tycho NT.6 (NanoTemper Technologies). All samples were measured in PBS (pH 7.4) at approximately 5 µM and 2.5 µM protein for monomers and dimers, respectively. Intrinsic fluorescence of tryptophan and tyrosine residues was recorded at 330 nm and 350 nm on a 30 °C/min temperature ramp from 35–95 °C. The ratio of fluorescence intensity (350/330 nm) and the inflection temperature (T_i) was calculated by software on the Tycho NT.6 instrument.

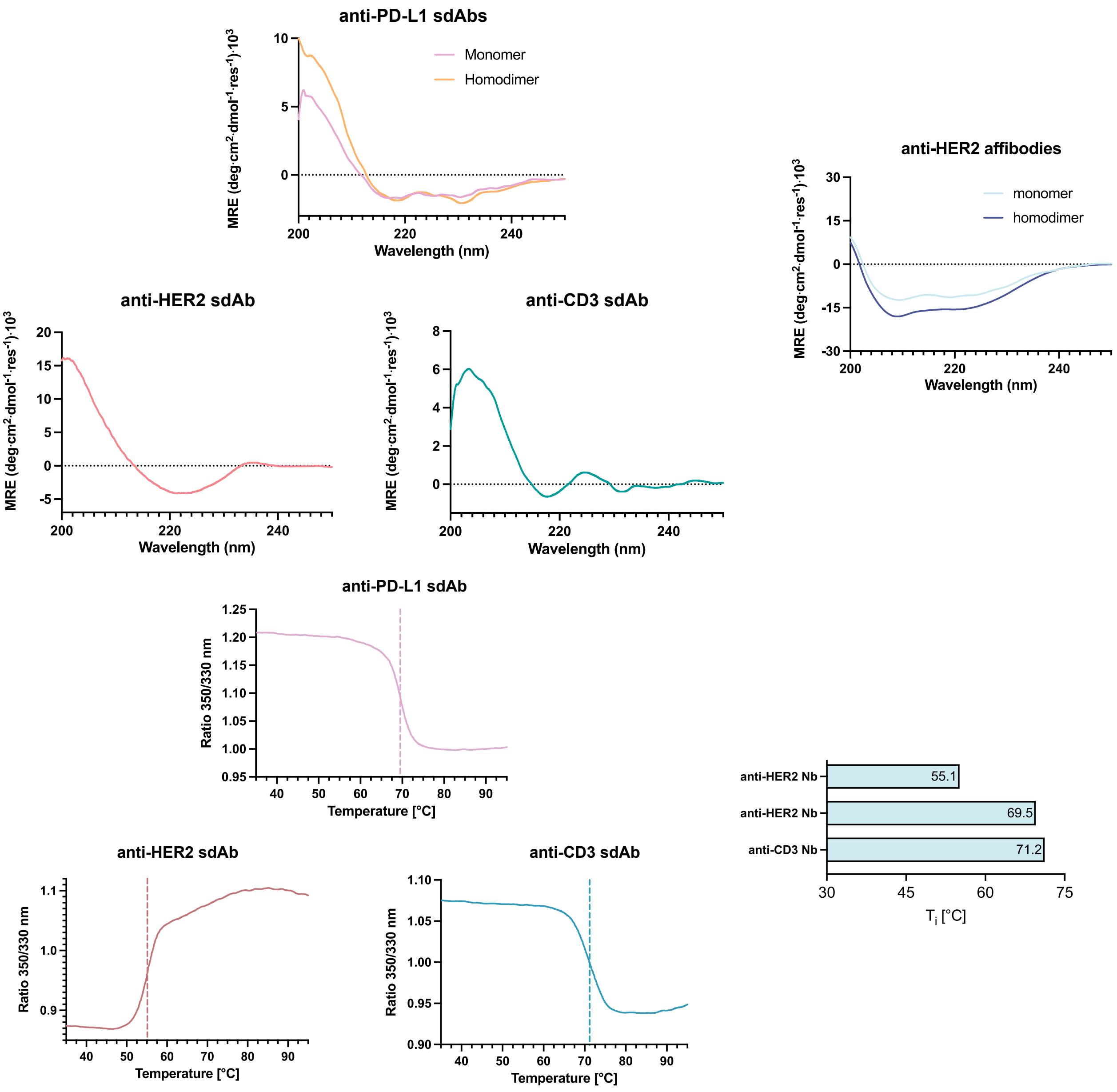

Figure S37: CD and nanoDSF spectra of all proteins expressed with additional CD spectra for **anti-PD-L1 homodimer** and **anti-HER2 affibody homodimer**, confirming negligible perturbation of protein secondary structure when using the homobifunctional linking strategy.

1.11 Binding assays

1.11.1 Biolayer Interferometry

BLI analyses were carried out using an Octet-BLI K2 instrument (ForteBio). Assays were performed at a constant temperature of 25 °C. throughout. Black flat-bottomed 96-well plates were used and filled with 200 μL per well. Streptavidin coated, Octet SA Biosensors (Sartorius) were pre-hydrated in assay buffer (PBS pH 7.4 + Tween-20 (0.02%) + BSA (0.05%)) for 20 min prior to use. For all assays, two biosensors (sample and reference) were used for each concentration assayed and in all cases to control for non-specific binding of the nanobodies to the sensor surface, the reference sensor with no antigen loaded was run in parallel and subtracted at each sdAb concentration. A sensor loaded with antigen and the association step containing only assay buffer was subtracted from all concentrations in order to account for any drift observed from loaded sensors in buffer. Analysis of the data were carried out to derive the k_a, k_d and K_D values for each binder using a kinetic association then dissociation model. A 1:1 binding model was used to fit data to extract the kinetic parameters for each constructs (K_Ds determined for bivalent binders are apparent values due to the avidity effect). The assays were carried out as follows:

PD-L1: 3 × regeneration 5 s cycles in glycine (10 mM, pH 1.8) followed by 5 s neutralisation in assay buffer, followed by a 30 s wash in assay buffer, 120 s loading using 2 ng/μL biotinylated hPD-L1 (ACROBiosystems, catalogue number: PD1-H82F3) for the sample sensor and buffer for the reference sensor, a second 30 s wash in assay buffer, then 30 s baseline in buffer; 60 s association in 2 μM, 1 μM, 500 nM, 250 nM and 125 nM of **anti-PD-L1 sdAb** monomer and **anti-HER2/PD-L1 heterodimer**; and 120 nM, 40 nM, 13.3 nM and 4.44 nM of **anti-PD-L1 homodimer**; followed by 90 s dissociation in assay buffer.

HER2: All neutralisation and wash steps were carried out as described for PD-L1, followed by 70 s loading using 2 ng/μL biotinylated HER2 (ACROBiosystems, catalogue number: HE2-H82E2) for the sample sensor and buffer for the reference sensor, a second 30 s wash in assay buffer, then 30 s baseline in buffer for all HER2 binding assays. For **anti-HER2 sdAb**

monomer and **anti-HER2/PD-L1 heterodimer** this was followed by 200 s association in 30 nM, 10 nM, 3.33 nM, 1.11 nM and 0.37 nM of **anti-HER2 sdAb** monomer and **anti-HER2/PD-L1 heterodimer**; and 500 s dissociation in assay buffer.

For **anti-HER2 sdAb homodimer**, a 360 s loading using 0.15 ng/μL biotinylated HER2 was carried out, whilst all other wash steps were the same. This was followed by 900 s association in 4 nM, 2 nM, 1 nM, 1 nM, 0.5 nM, 0.25 nM and 0.125 nM of **anti-HER2 sdAb homodimer**; and 900 s dissociation in assay buffer.

For the **anti-HER2 affibody** monomer and **anti-HER2 affibody homodimer** a 180 s loading using 2 ng/μL biotinylated HER2 was carried out, whilst all other wash steps were the same. This was followed by a 700 s association in 200 nM, 66.7 nM, 22.2 nM and 7.40 nM of **anti-HER2 affibody monomer**; and 150 nM, 75 nM, 37.5 nM, 18.8 nM and 9.40 nM; and a 700 s dissociation in assay buffer.

For **anti-HER2 biparatopic heterodimer**, a 100 s loading using 2 ng/μL biotinylated HER2 was carried out, whilst all other wash steps were the same. This was followed by 2500 s association in 5 nM, 1.67 nM, 0.556 nM and 0.185 nM of **anti-HER2 biparatopic heterodimer**; and 2500 s dissociation in assay buffer.

Note: All monomers had their cysteine residue capped with NMM as described in the bioconjugation methods.

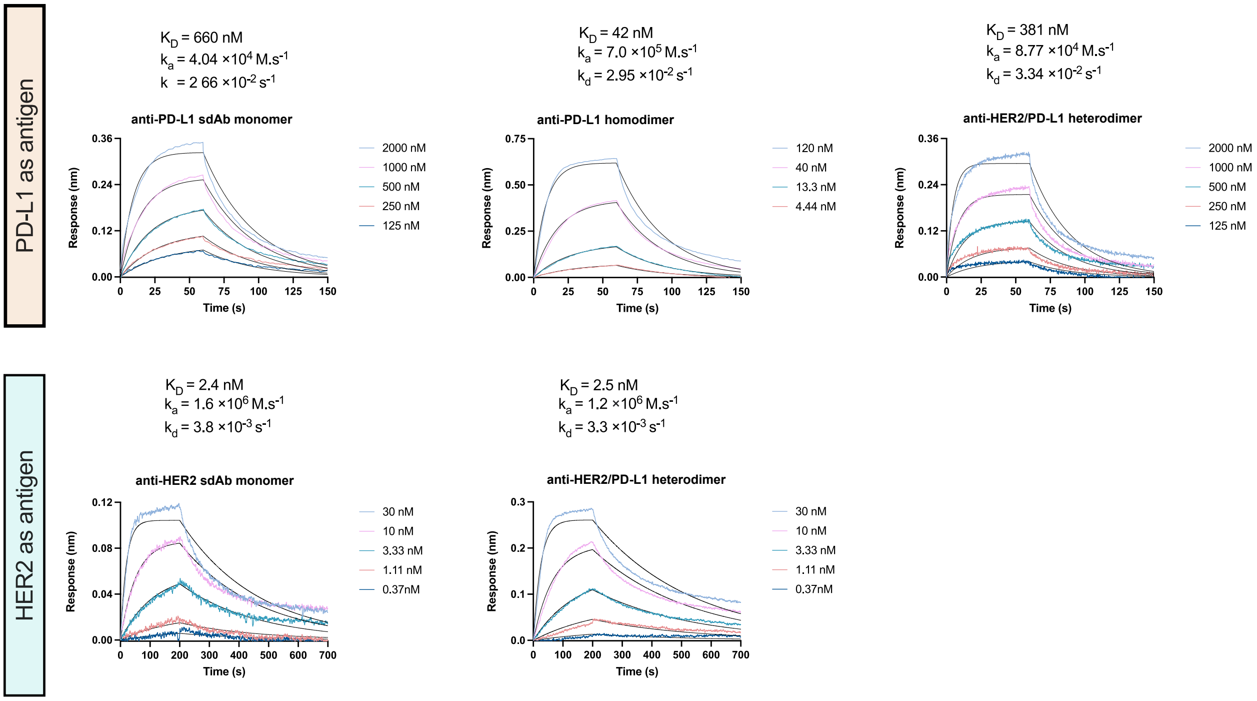

Figure S38: BLI association and dissociation sensograms obtained for **anti-PDL1 sdAb** derived homo- and heterodimers and their substituent monomers binding to PD–L1 and HER2. Analysis of the data were carried out to derive the k_a, k_d and K_D values for each binder using a kinetic association then dissociation model, shown above each sensogram. A 1:1 binding model was used to fit data to extract the kinetic parameters for each binder using well established methods in Octet Analysis Studio.

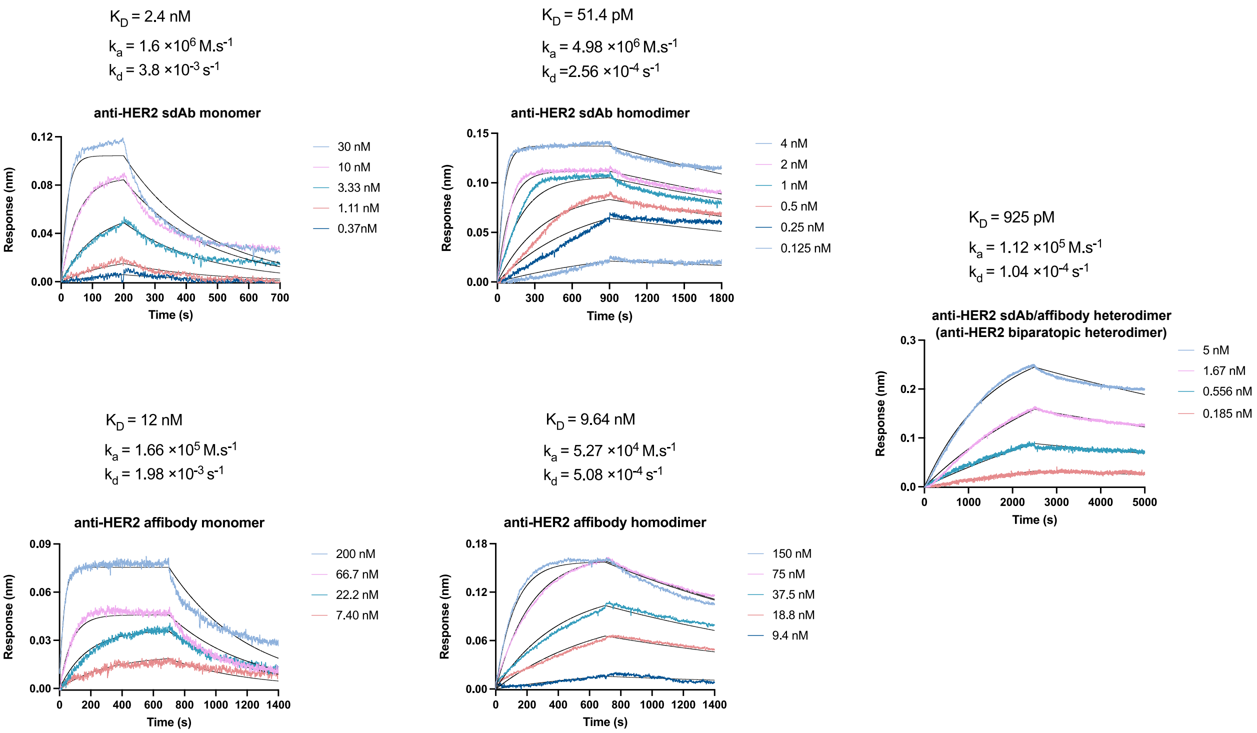

Figure S39: BLI association and dissociation sensograms obtained for all anti-HER2 monomer, homodimer and heterodimeric binders. Analysis of the data was carried out to derive the k_a, k_d and K_D values for each binder using a kinetic association then dissociation model, shown above each sensogram. A 1:1 binding model was used to fit data to extract the kinetic parameters for each binder using well established methods in Octet Analysis Studio. Note: K_Ds determined for bivalent binders are apparent values due to the avidity effect.

Table S4: Kinetic parameters (k_a, k_d and K_D) for all dimers produced extracted from BLI sensograms. A 1:1 binding model was used to fit data to extract the kinetic parameters for each binder using well established methods, therefore, parameters extracted for bivalent binders are apparent values due to the avidity effect.

Binder	Antigen	k_a ($M.s^{-1}$)	k_d (s^{-1})	K_D (nM)
**anti-PD-L1 sdAb** monomer	hPD-L1	4.0×10^4	2.7×10^{-2}	660
**anti-HER2/PD-L1 heterodimer**	hPD-L1	8.8×10^4	3.3×10^{-2}	381
**anti-PD-L1 homodimer**	hPD-L1	7.0×10^5	3.0×10^{-2}	42
**anti-HER2 sdAb** monomer	HER2	1.6×10^6	3.8×10^{-3}	2.4
**anti-HER2/PD-L1 heterodimer**	HER2	1.2×10^6	3.3×10^{-3}	2.5
**anti-HER2 sdAb homodimer**	HER2	5.0×10^6	2.6×10^{-4}	0.051
**anti-HER2 affibody** monomer	HER2	1.7×10^5	2.0×10^{-3}	12
**anti-HER2 affibody homodimer**	HER2	5.3×10^4	5.1×10^{-4}	9.6
**anti-HER2 biparatopic heterodimer**	HER2	1.1×10^5	1.0×10^{-4}	0.93

1.11.2 Biolayer Interferometry dual engagement binding assay

Octet SA Biosensors (Sartorius) were pre-hydrated in assay buffer (PBS pH 7.4 + Tween-20 (0.02%) + BSA (0.05%)) for 20 min prior to use. Each tip was loaded with HER2 in an analogous way to that described in the standard BLI experiments, and subsequently placed into an association well for 800 s. Each association well contained different combinations of dimers and mPD-L1 (ACROBiosystems, catalogue number: PD1-M5251) as follows; 25 nM **anti-HER2/PD-L1 heterodimer** pre-incubated with 1 μM mPD-L1; 25 nM **anti-HER2/PD-L1 heterodimer** in assay buffer; 25 nM **anti-HER2 sdAb homodimer** preincubated with 1 μM mPD-L1, 25 nM **anti-HER2 sdAb homodimer** in assay buffer; 1 μM mPD-L1 in assay buffer; and pure assay buffer. The relative response for each

combination was observed and the sensograms overlaid (Figure S40) to allow the effect of simultaneous engagement to be assessed.

(a)

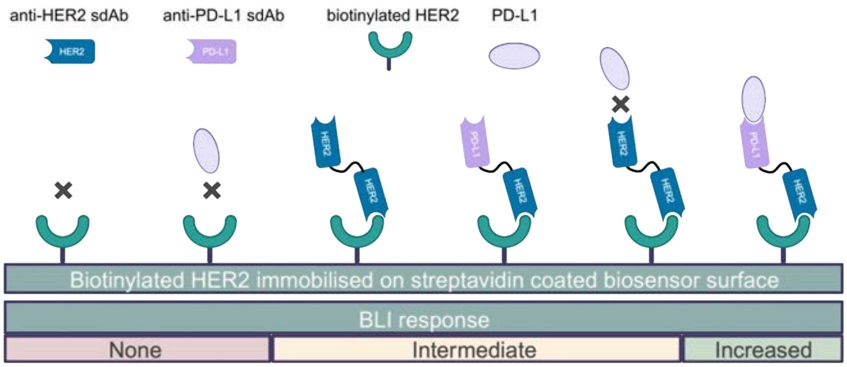

(b)

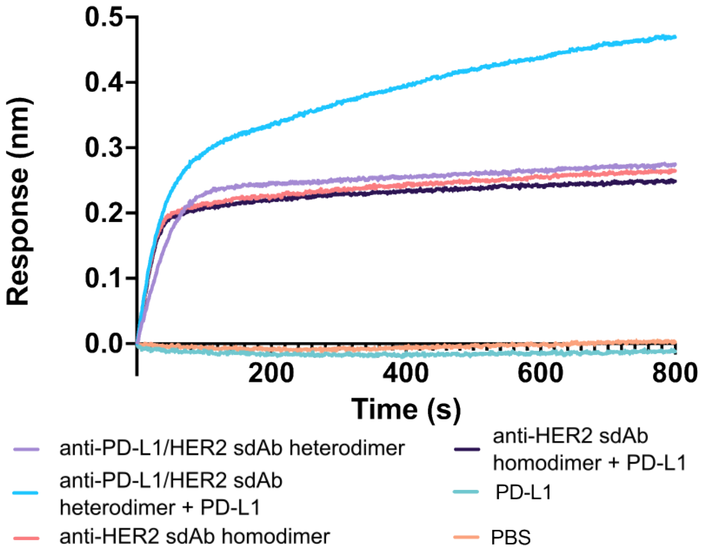

Figure S40: a) A graphical representation of the dual engagement BLI assay indicating the expected relative magnitude of BLI response dependent on the size of the species binding to HER2 immobilised on the biosensor. b) The experimental BLI sensograms for all assayed conditions were in line with the theoretical predictions.

1.12 Cell imaging protocol

SKBR3 and MCF-7 cells were seeded at a density of 60000 cells/well, in 13 mm round cover slips (# 1.5, 0.16–0.19 mm) placed in 24-well plates and coated with poly-D lysine. The HER2 binders (100 nM in PBS [pH 7.4], 250 μL/well) were added on the second day. After 4 h of incubation, the cells were fixed with a 4% paraformaldehyde solution in PBS (500 μL/well) for 20 min, protected from light. After this time, the cells were treated with Phalloidin-iFluor^{TM} 647 Conjugate (250 μL/well) for 20 min, protected from light. The cover slips were removed and mounted with ProLong Gold Antifade Mountant with DAPI. After each treatment step, the cells were washed twice with PBS (pH 7.4). Cells were imaged using a Leica DMi8 confocal microscope with a 40× oil immersion objective, using the DAPI, Alexa Fluor 488 and Alexa Fluor 647 filter cubes. Raw images were imported using "default" colour mode into Fiji ImageJ2 (version 2.14.0/1.54f).[S1] for visualization and analysis. For comparative purposes the maximum colour for Channel 3 (Alexa Fluor 488) was manually set to a value of 30 for every image using the colour balance tool, while Channels 1 and 2 (nuclei and cytoskeleton) were adjusted automatically.

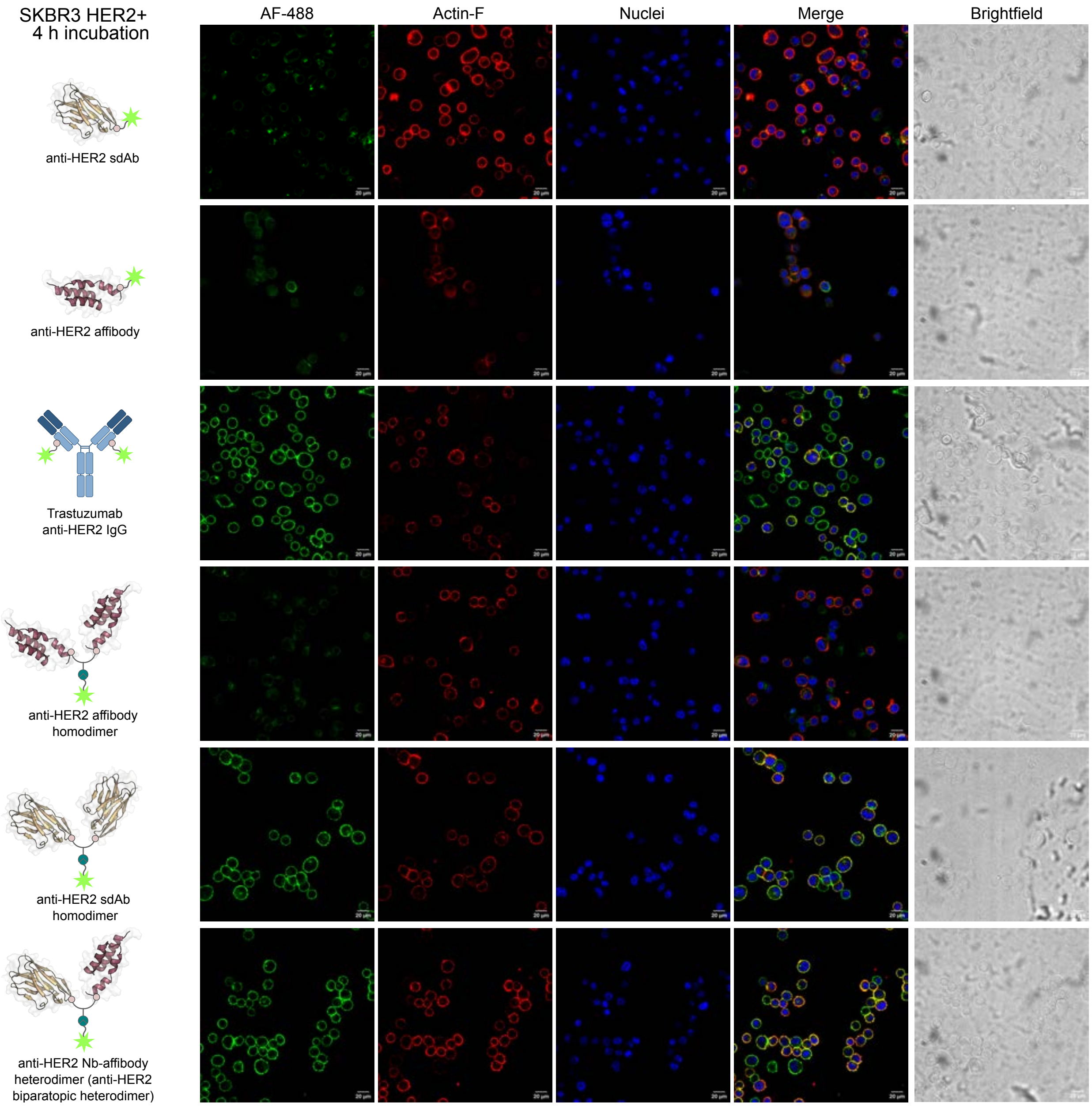

Figure S41: Confocal images of HER2+ SKBR3 cells treated with a panel of HER2 binders labelled with Alexa Fluor 488 for 4 h. No significant variation in internalisation was observed when comparing monovalent and bivalent binders. However, a clear increase in avidity mediated membrane localisation after multiple washing steps was observed in trastuzumab, **anti-HER2 sdAb homodimer**, and **anti-HER2 biparatopic heterodimer**, with colocalisation of Alexa Fluor 488 labelled binders and membrane specific Phalloidin-iFluor^{TM} 647. Interestingly, the **anti-HER2 affibody homodimer** did not display avidity mediated binding in the context of cell membrane presented receptors. (Green: Alexa Fluor 488 labelled binder, Red: Phalloidin-iFluor^{TM} 647 stain for F-Actin in the cytoskeleton, Blue: DAPI nuclear stain.)

.

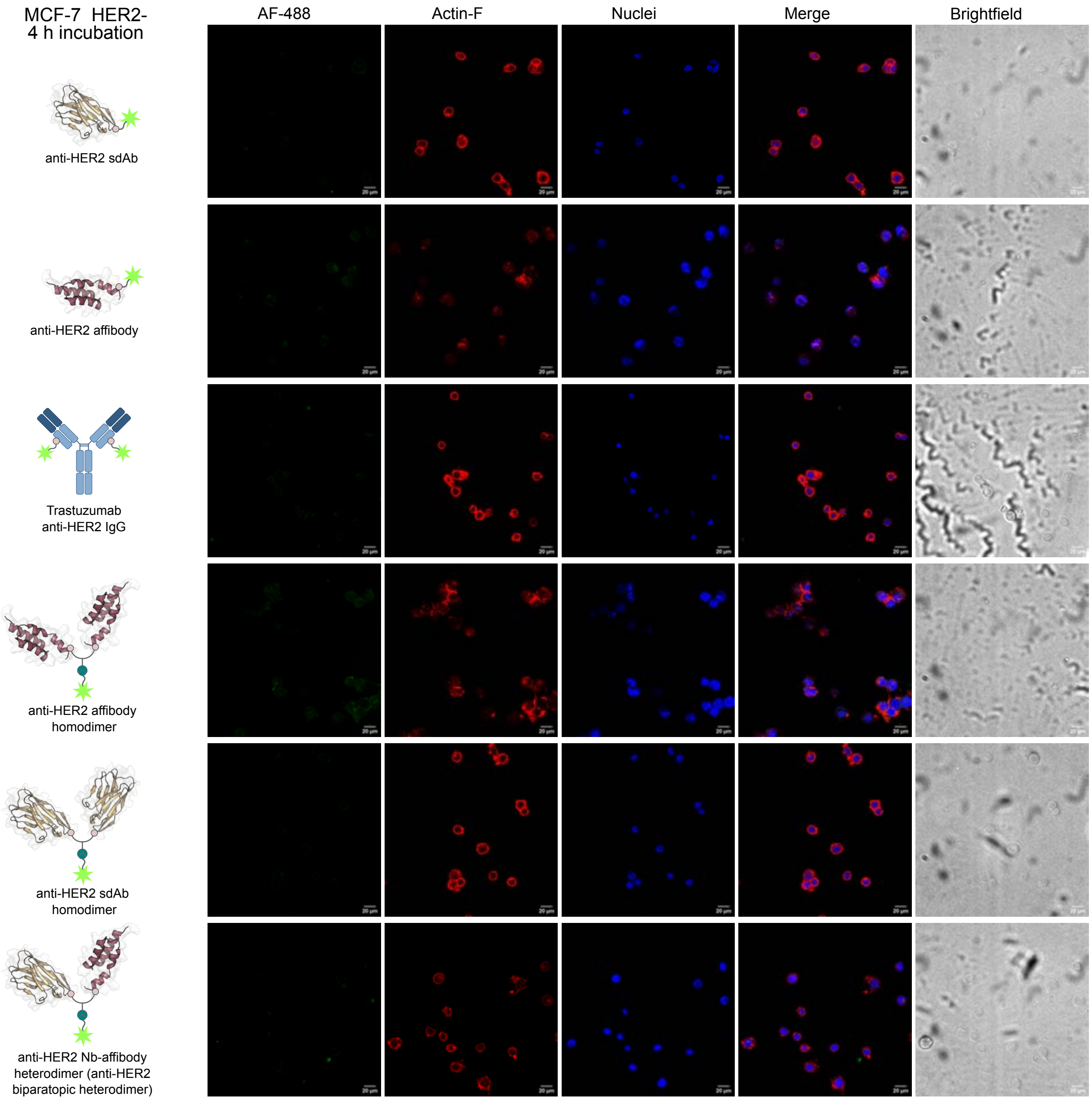

Figure S42: Confocal images of HER2- MCF-7 cells treated with a panel of HER2 binders labelled with Alexa Fluor 488 for 4 h. Serving as a negative control, the lack of fluorescence in the Alexa Fluor 488 channel confirms the binding and internalisation observed in SKBR3 cells was specific for HER2 receptors. (Green: Alexa Fluor 488 labelled binder, Red: Phalloidin-iFluor^{TM} 647 stain for F-Actin in the cytoskeleton, Blue: DAPI nuclear stain.)

2 Protein amino acid sequences

anti-PD-L1

MAQVQLVETGGGLVQPGGSLRLSCTASGFTFSMHAMTWYRQAPGKQRELVAVITS
HGDRANYTDSVRGRFTISRDNTKNMVYLQMNSLKPEDTAVYYCNVPRYDSWGQG
TQVTVSSSPSTPPTPSPSTPPCGENLYFQGLEHHHHHH

Calculated molecular weight: 16239 Da

Theoretical extinction coefficient: 21555 $M^{-1}cm^{-1}$

anti-HER2 sdAb (2Rb17c)

EVQLQESGGGLVQPGGSLRLSCAASGFIFSNDAMTWVRQAPGKGLEWVSSINWSGT
HTNYADSVKGRFTISRDNAKRTLYLQMNSLKDEDTALYYCVTGYGVTKTPTGQGT
QVTVSSDCEGSENLYFQGHHHHHH

Calculated molecular weight: 14768 Da

Theoretical extinction coefficient: 25565 $M^{-1}cm^{-1}$

anti-HER2 affibody (ZHER2)

GHHHHHHGVDNKFNKEMCNAYWEIALLPNLNNQQKRAFIRSLYDDPSQSANLLAE
AKKLNDAQAPK

Calculated molecular weight: 7589 Da

Theoretical extinction coefficient: 8480 $M^{-1}cm^{-1}$

anti-CD3

EVQLVESGGGPVQAGGSLRLSCAASGRTYRGYSMGWFRQAPGKEREFVAAIVWSG
GNTYYEDSVKGRFTISRDNAKNTMYLQMTSLKPEDSATYYCAAKIRPYIFKIAGQY
DYWGQGTQVTVSSDCEGSENLYFQGHHHHHH

Calculated molecular weight: 15859 Da

Theoretical extinction coefficient: 33015 $M^{-1}cm^{-1}$

EGFP-Cys

MRGSHHHHHHGMASMTGGQQMGRDLYENLYFQCSSMVSKGEELFTGVVPILVELD
GDVNGHKFSVSGEGEGDATYGKLTLKFICTTGKLPVPWPTLVTTLTYGVQCFSRY
PDHMKQHDFFKSAMPEGYVQERTIFFKDDGNYKTRAEVKFEGDTLVNRIELKGIDF
KEDGNILGHKLEYNYNSHNVYIMADKQKNGIKVNFKIRHNIEDGSVQLADHYQQNT
PIGDGPVLLPDNHYLSTQSALSKDPNEKRDHMVLLEFVTAAGITLGMDELYK

Calculated molecular weight: 30953 Da

Theoretical extinction coefficient: 24870 $M^{-1}cm^{-1}$

3 LC–MS spectra

3.1 LC–MS spectra of unmodified proteins

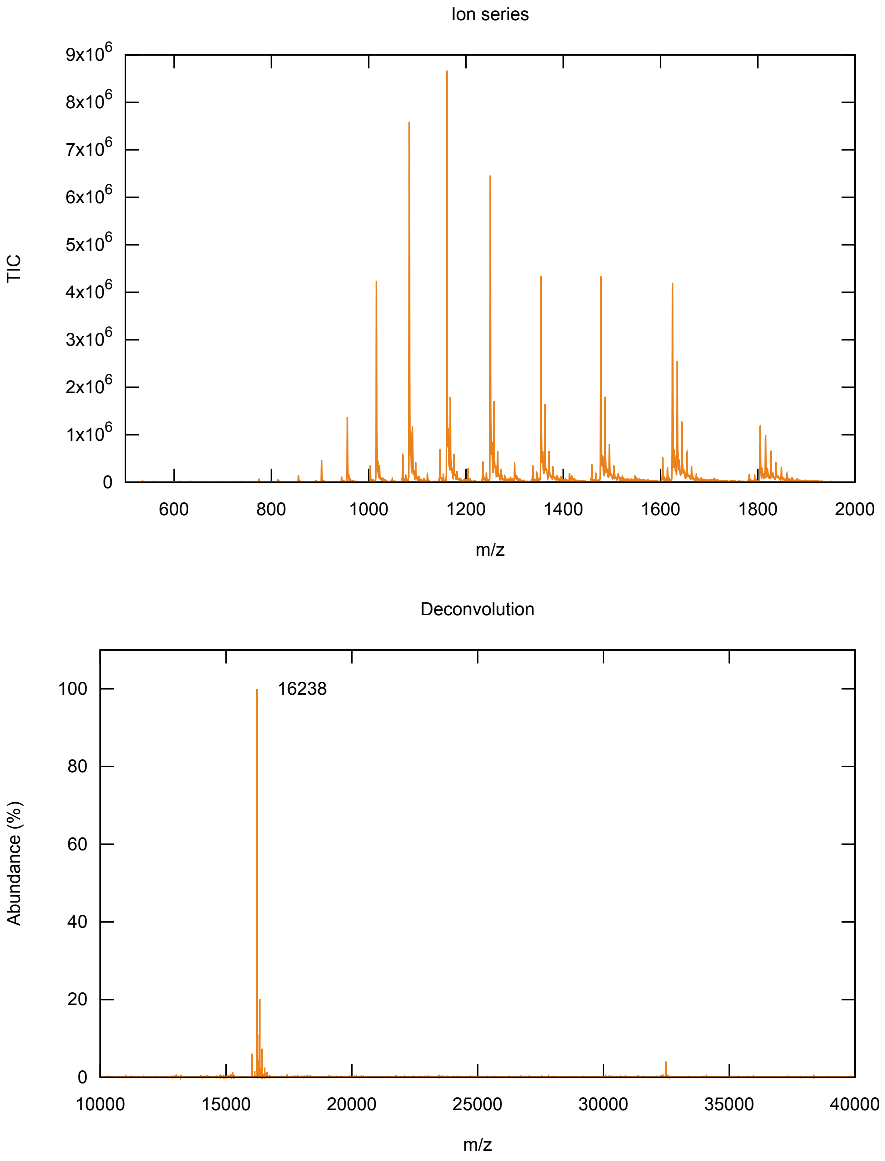

Figure S43: LC–MS spectra of **anti-PD-L1 sdAb** monomer; ion series and deconvoluted spectra (Calculated mass: 16239 Da).

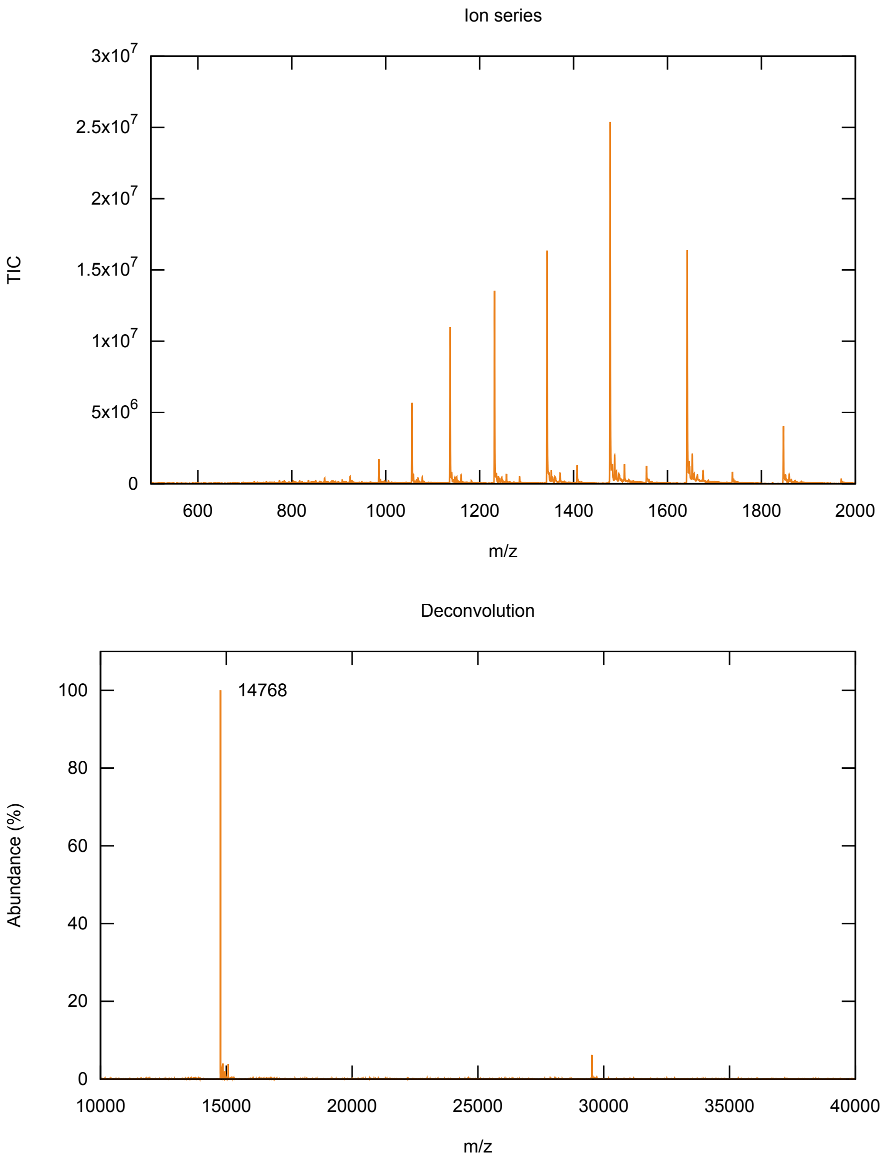

Figure S44: LC–MS spectra of **anti-HER2 sdAb** (2Rb17c) monomer; ion series and deconvoluted spectra (Calculated mass: 14768 Da).

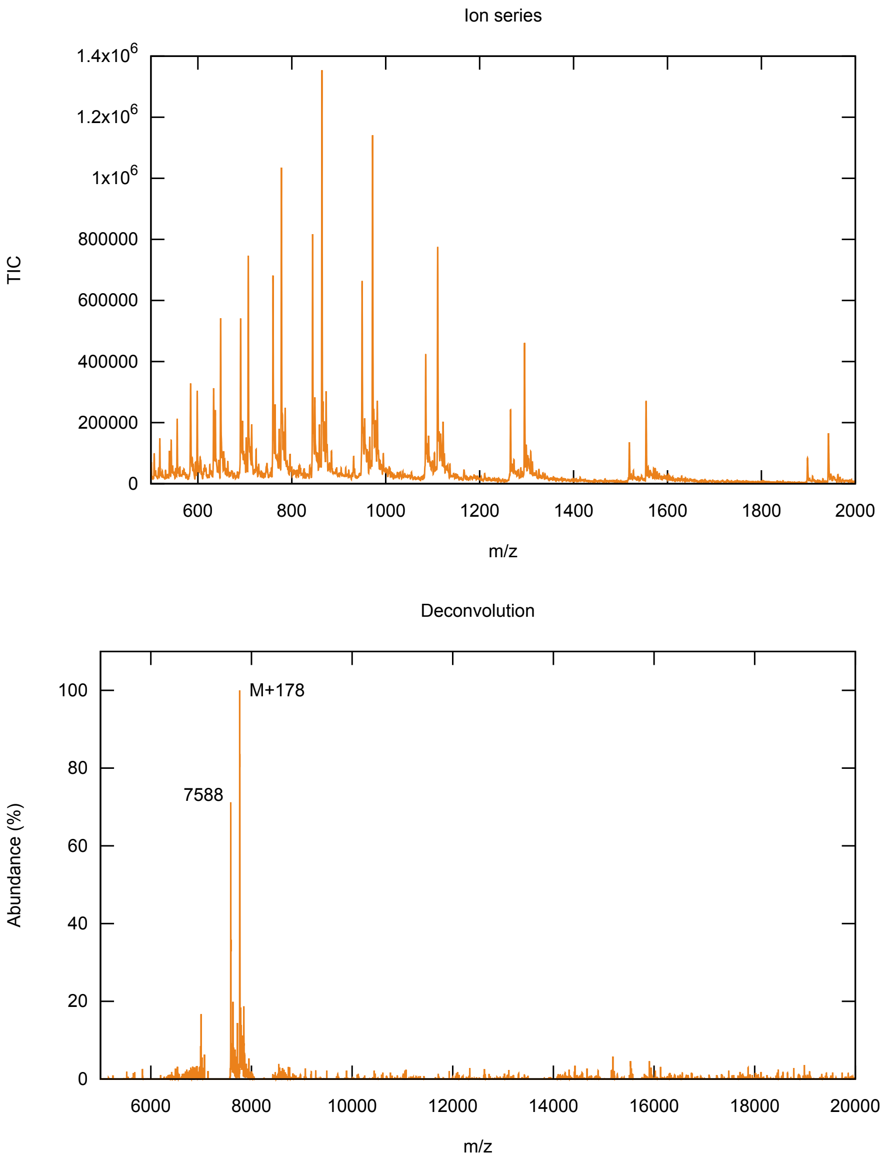

Figure S45: LC–MS spectra of **anti-HER2 affibody monomer**; ion series and deconvoluted spectra (Calculated mass: 7589 Da). M+178 and M+258 Da peaks correspond to partial α-N-6-phosphogluconoylation of the hexahistidine tag.[S9]

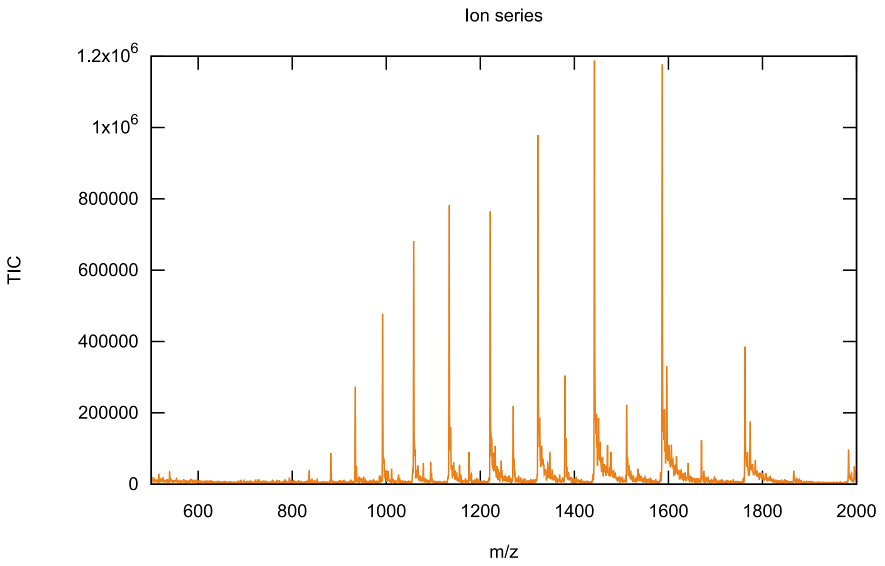

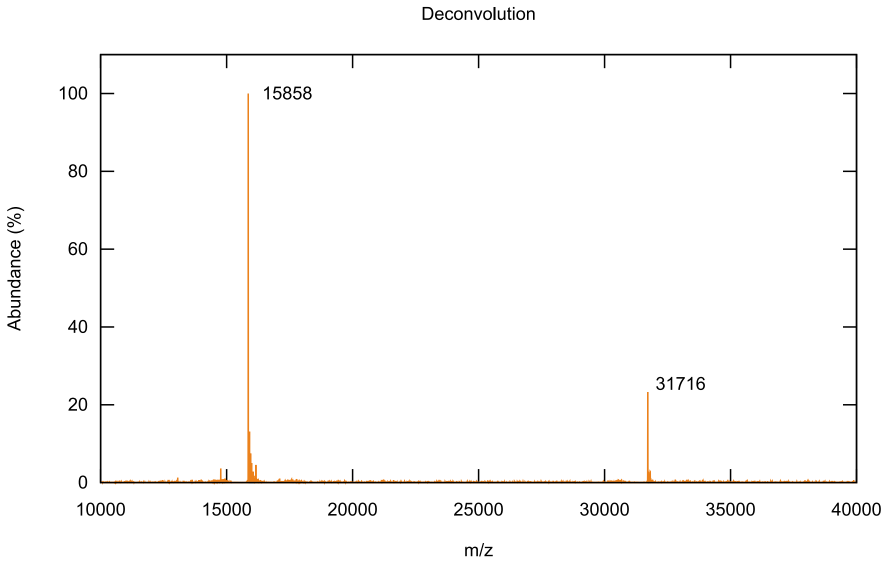

Figure S46: LC–MS spectra of **anti-CD3 sdAb** monomer; ion series and deconvoluted spectra (Calculated mass: 15858 Da). 31716 Da peak corresponds to dimerisation via disulfide bond formation.

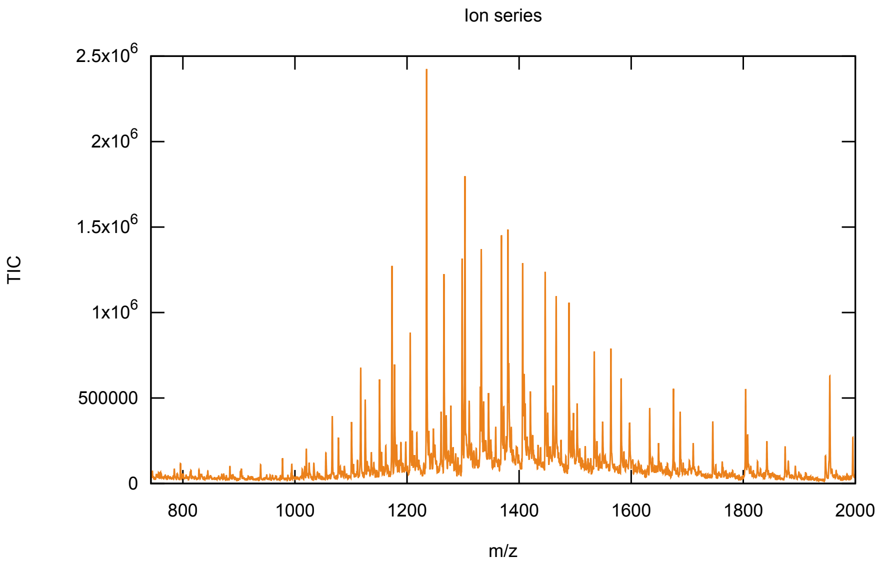

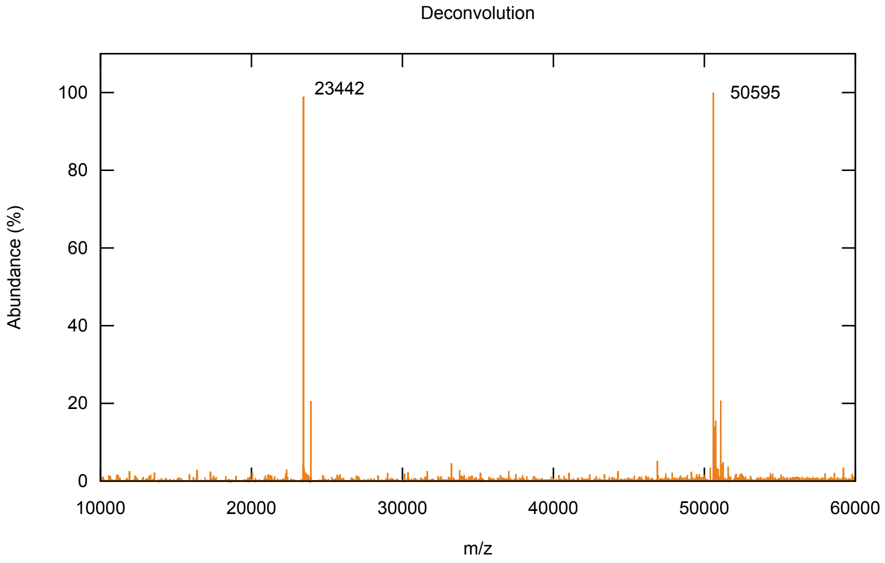

Figure S47: LC–MS spectra of reduced, commercially sourced Thiomab (Trastuzumab V205C (LC)); ion series and deconvoluted spectra. (The antibody was supplied by Genentech)

3.2 Optimisation of homodimerisation and monomer-linker conjugation

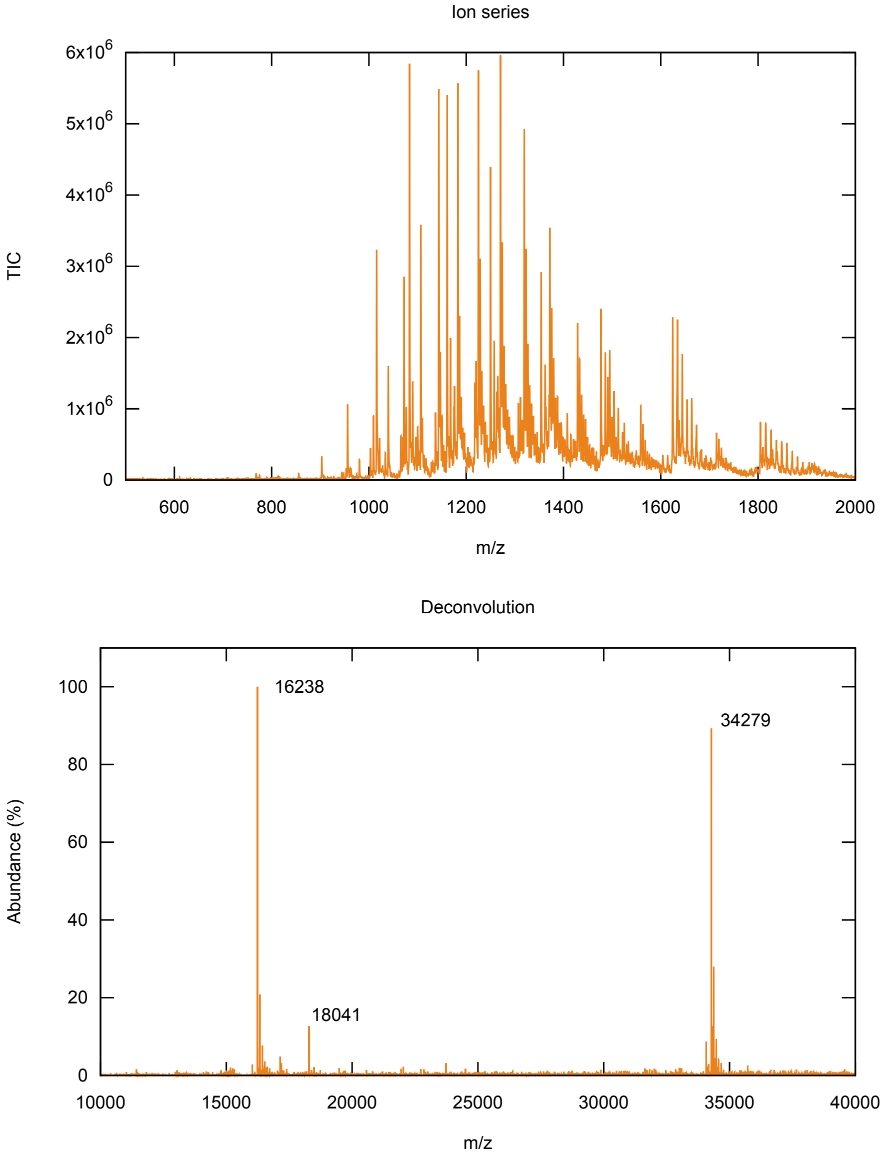

Figure S48: LC-MS analysis of **anti-PD-L1 sdAb** homodimerisation in the presence of 0.6 equivalents of homobifunctional **Linker I** after 10 min; ion series and deconvoluted spectra (Calculated mass: 34278 Da).

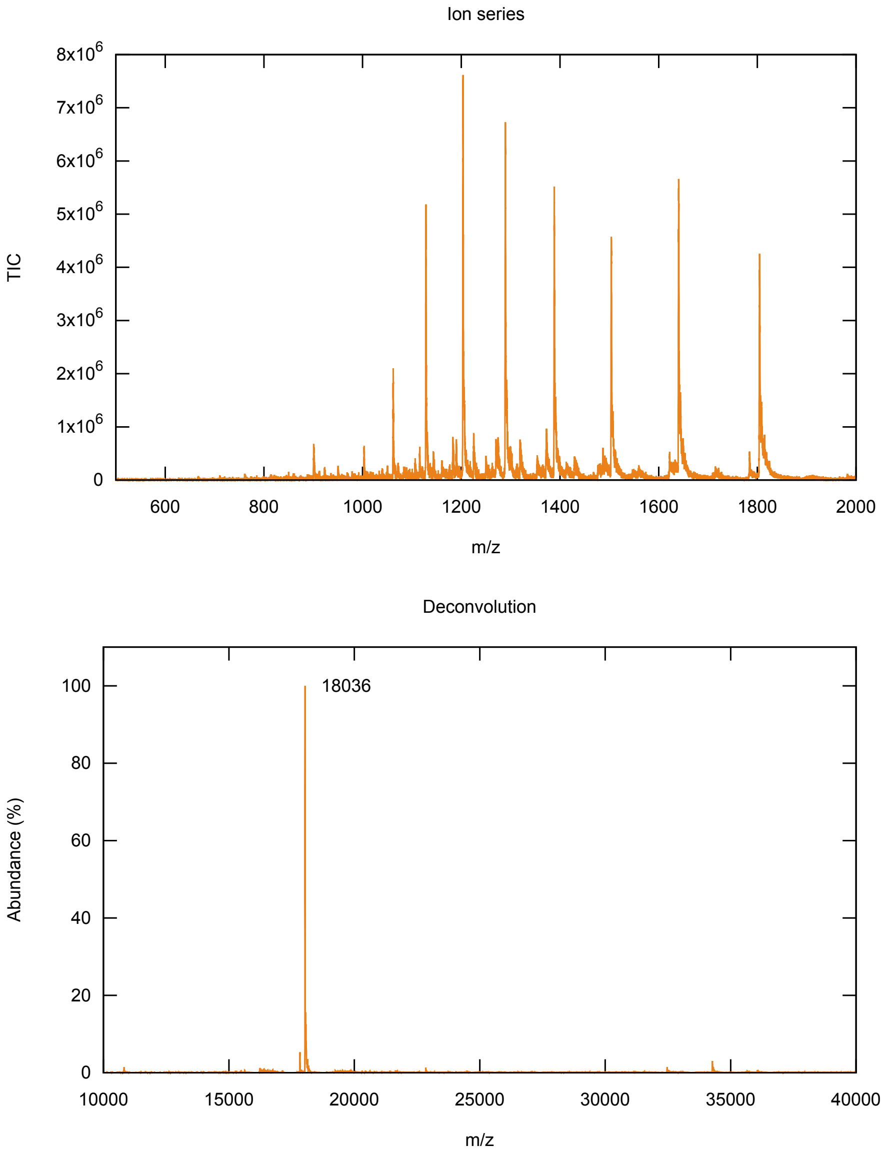

Figure S49: Deconvoluted LC–MS spectrum of **anti-PD-L1 sdAb** monomer functionalised with excess bis-maleimide linker; ion series and deconvoluted spectra (Calculated mass: 18039 Da).

3.3 LC–MS spectra homodimeric protein–protein conjugates

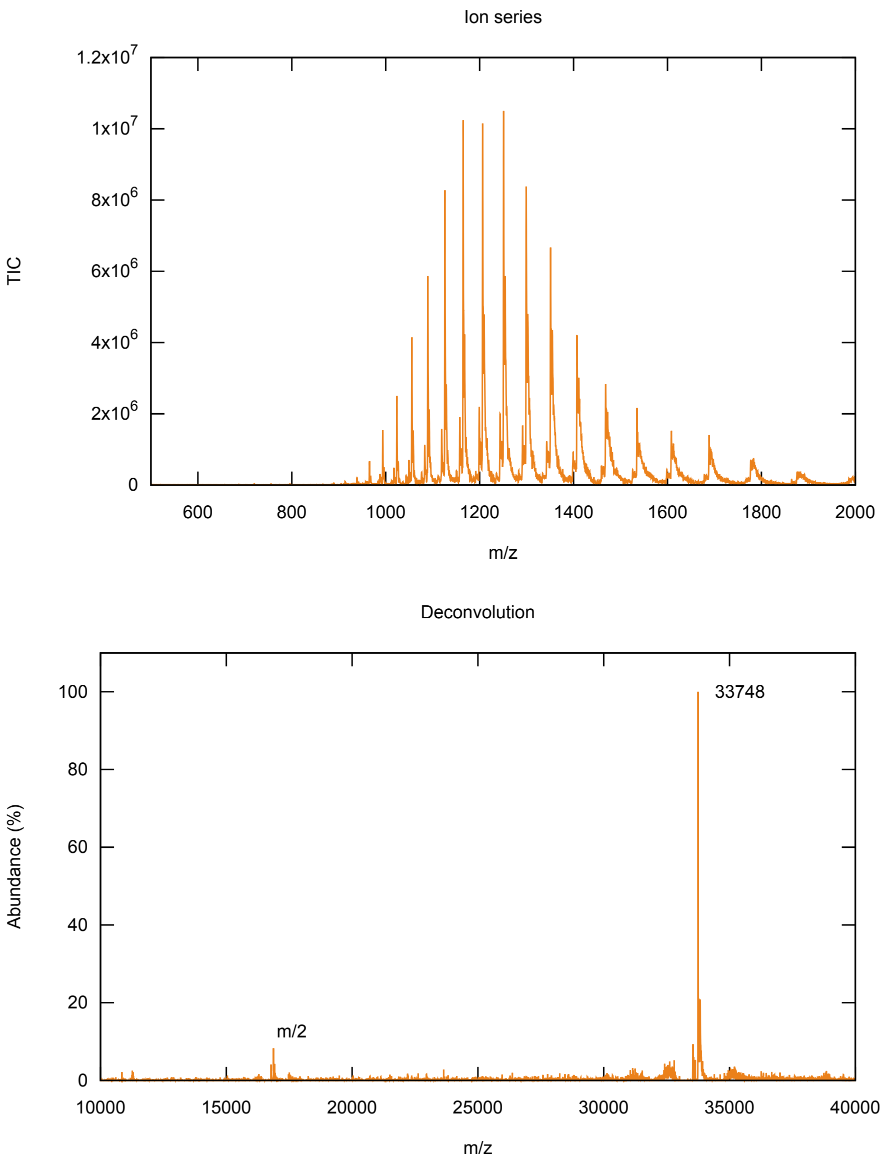

Figure S50: LC-MS spectra of stabilised **anti-PD-L1 homodimer** from **Linker I** after preparative size exclusion chromatography; ion series and deconvoluted spectra (Calculated mass: 33750 Da).

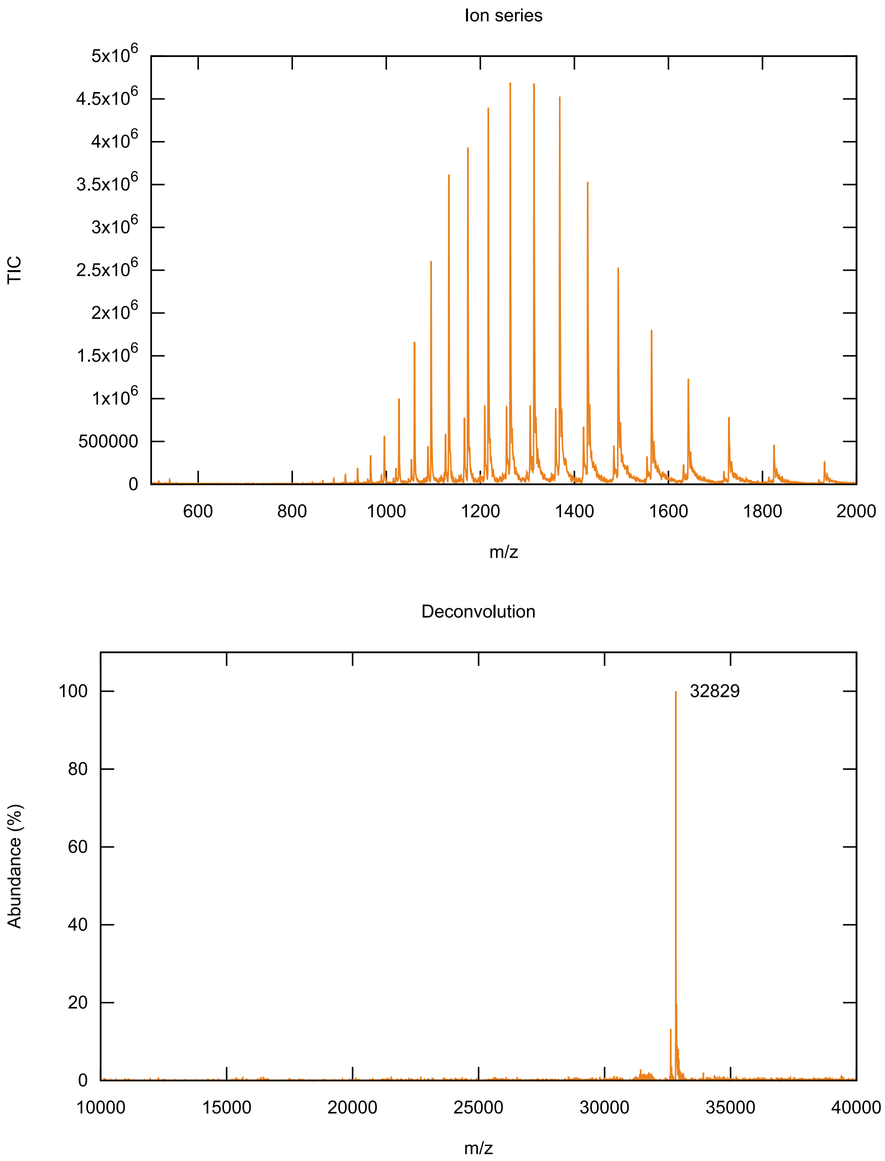

Figure S51: LC-MS spectra of **anti-PD-L1 homodimer** from commercial bis-maleimide linker (1,11-bismaleimidotriethyleneglycol) after preparative size exclusion chromatography; ion series and deconvoluted spectra (Calculated mass: 32830 Da).

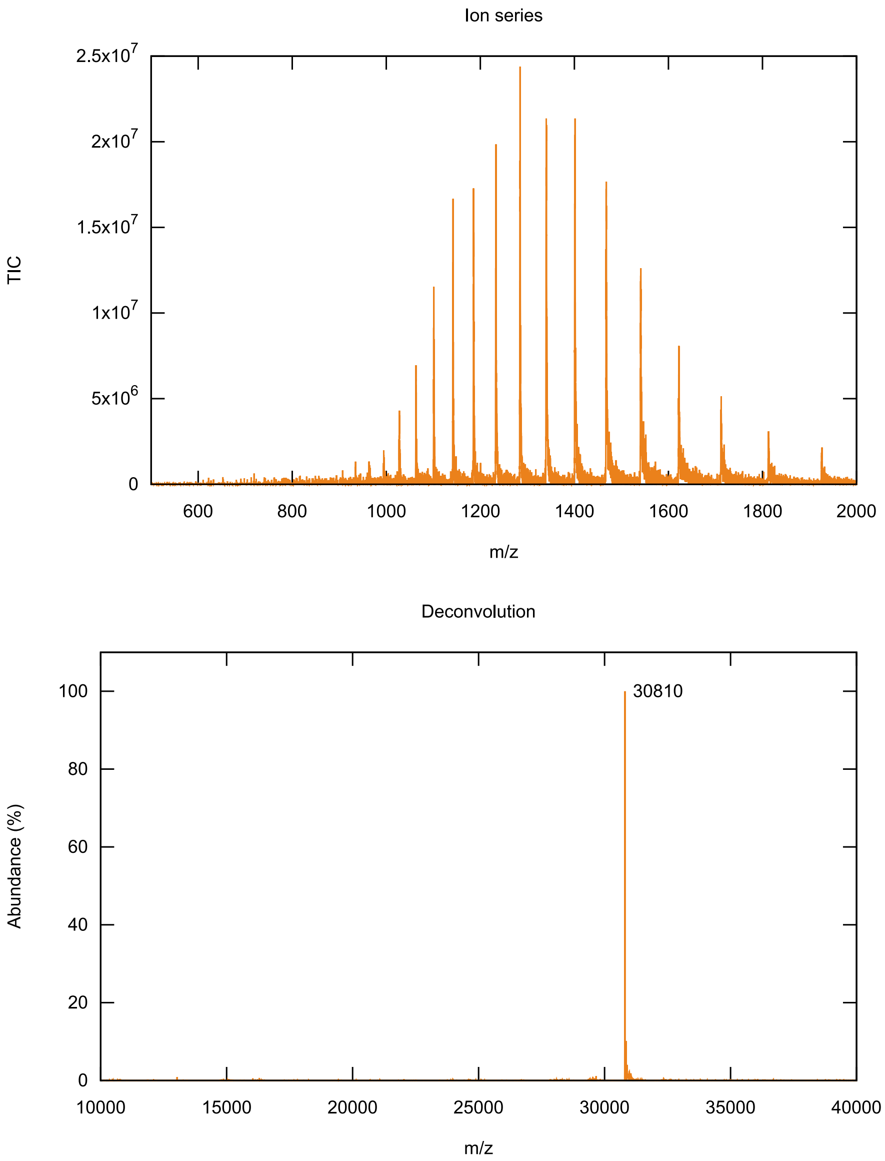

Figure S52: LC-MS spectra of stabilised **anti-HER2 sdAb homodimer** after preparative size exclusion chromatography; ion series and deconvoluted spectra (Calculated mass: 30810 Da).

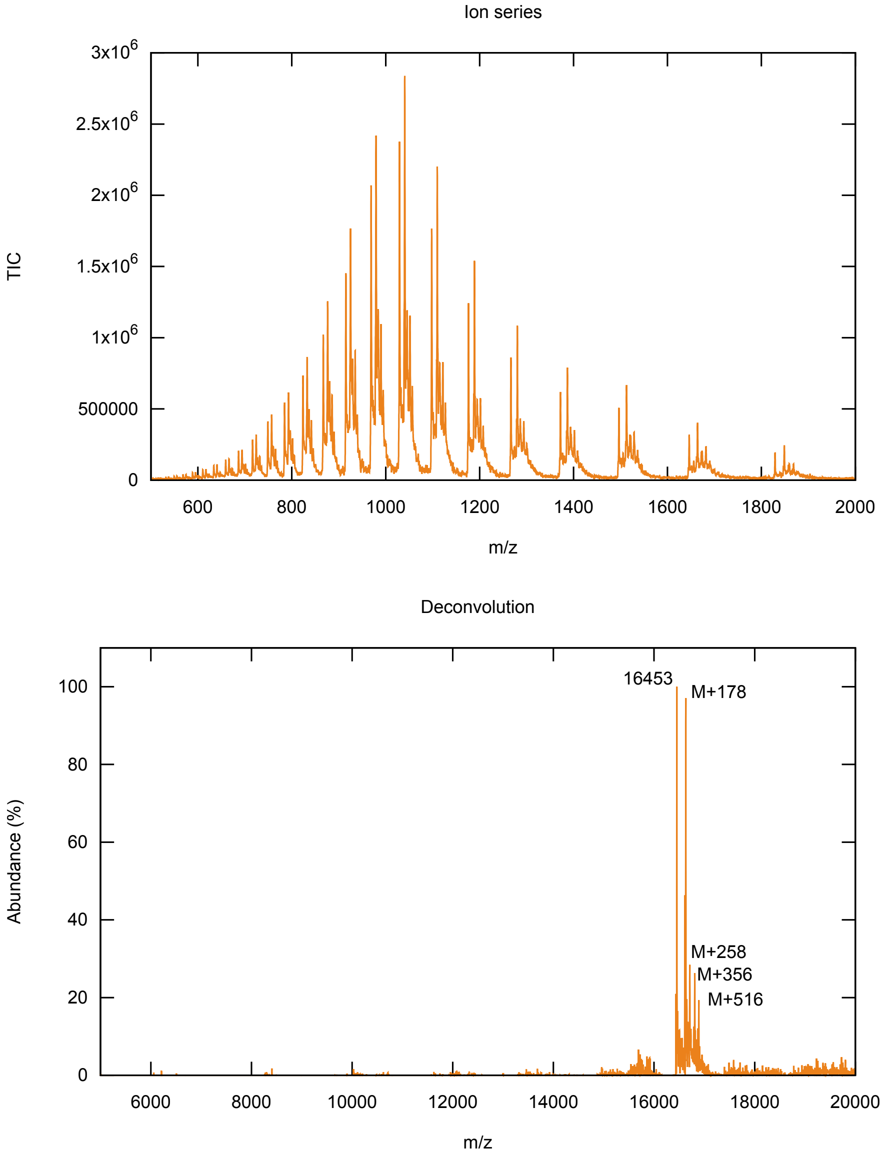

Figure S53: LC-MS spectra of stabilised **anti-HER2 affibody homodimer** after preparative size exclusion chromatography; ion series and deconvoluted spectra (Calculated mass: 16452 Da). M+178, M+258, M+356 and M+516 Da peaks correspond to partial α-N-6-phosphogluconoylation of the hexahistidine tag.[S9]

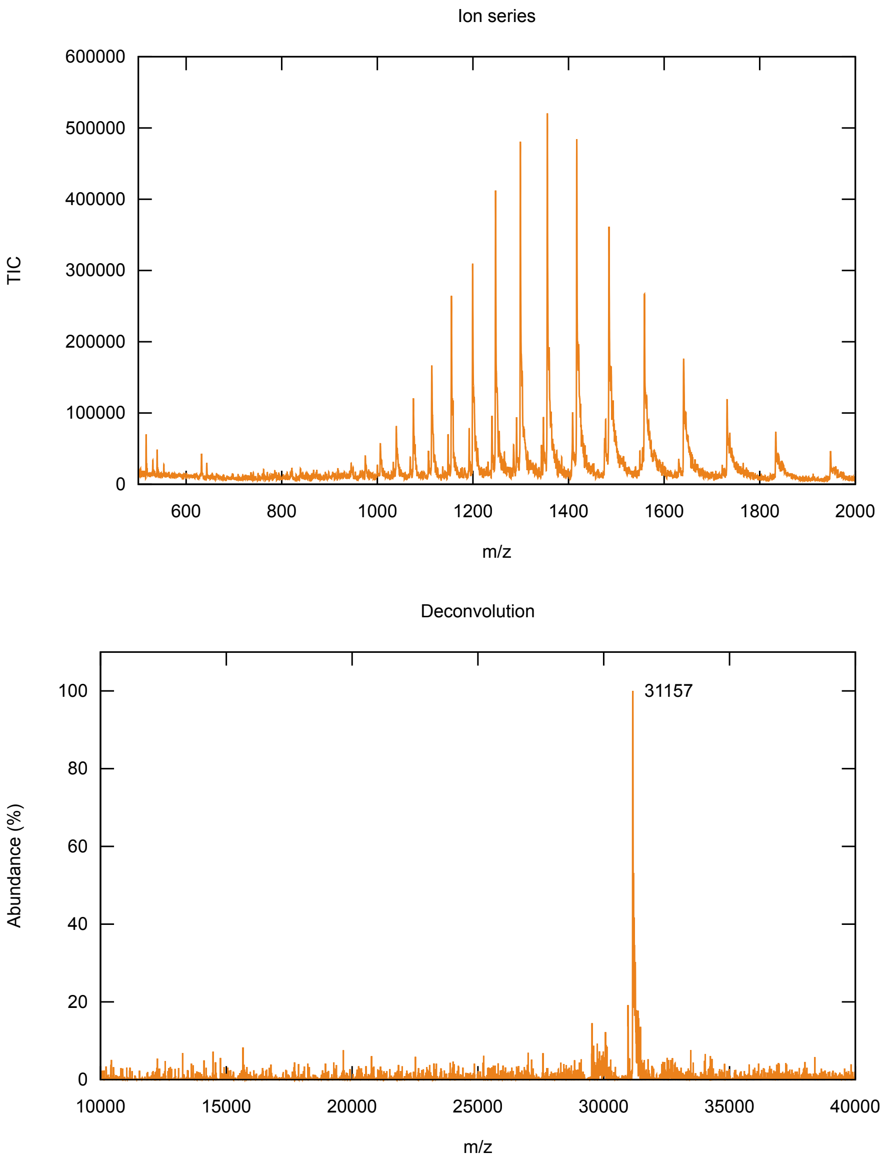

Figure S54: LC-MS spectra of stabilised **anti-HER2 sdAb homodimer–DBCO** generated from **Linker V**, after preparative size exclusion chromatography; ion series and deconvoluted spectra (Calculated mass: 31158 Da).

3.4 LC–MS analysis of heterodimers and their intermediates

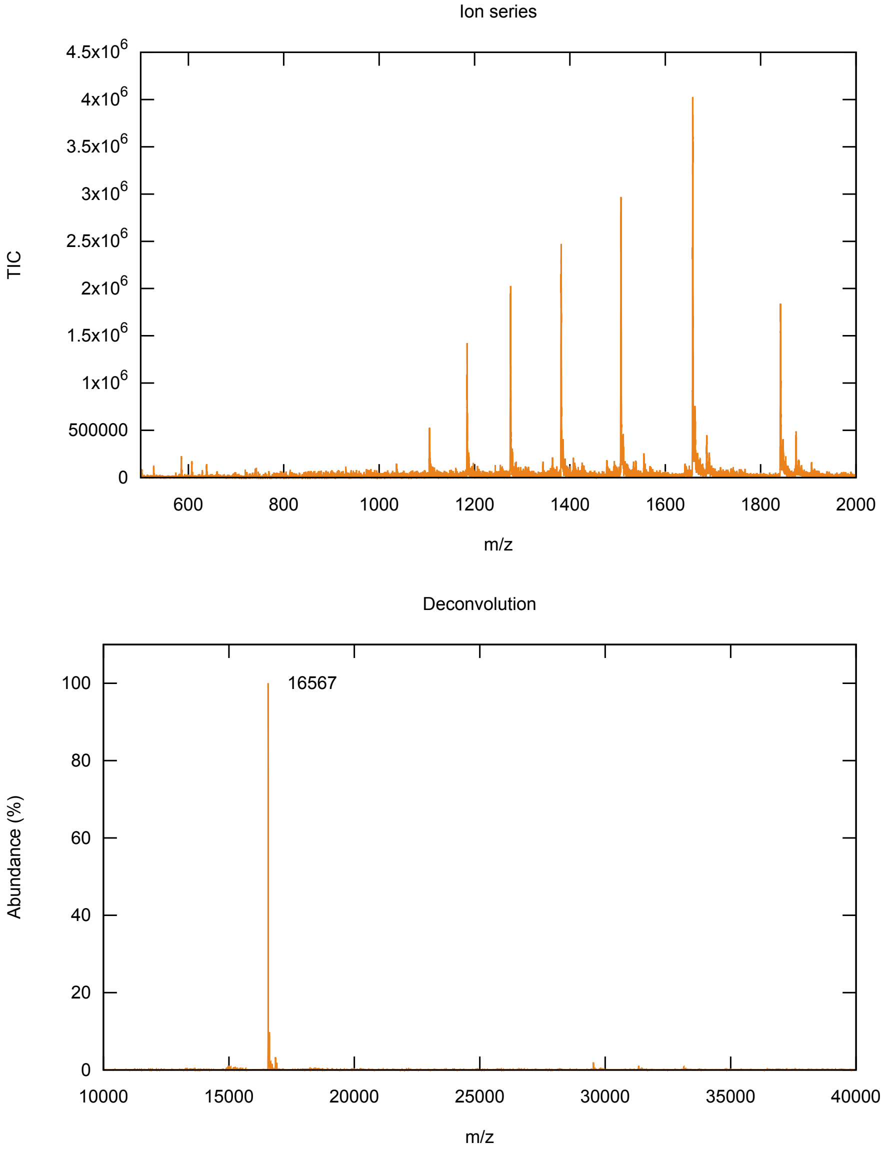

Figure S55: LC-MS spectra of **anti-HER2 sdAb** monomer functionalised with excess bis-maleimide **Linker I** ; ion series and deconvoluted spectra (Calculated mass: 16568 Da).

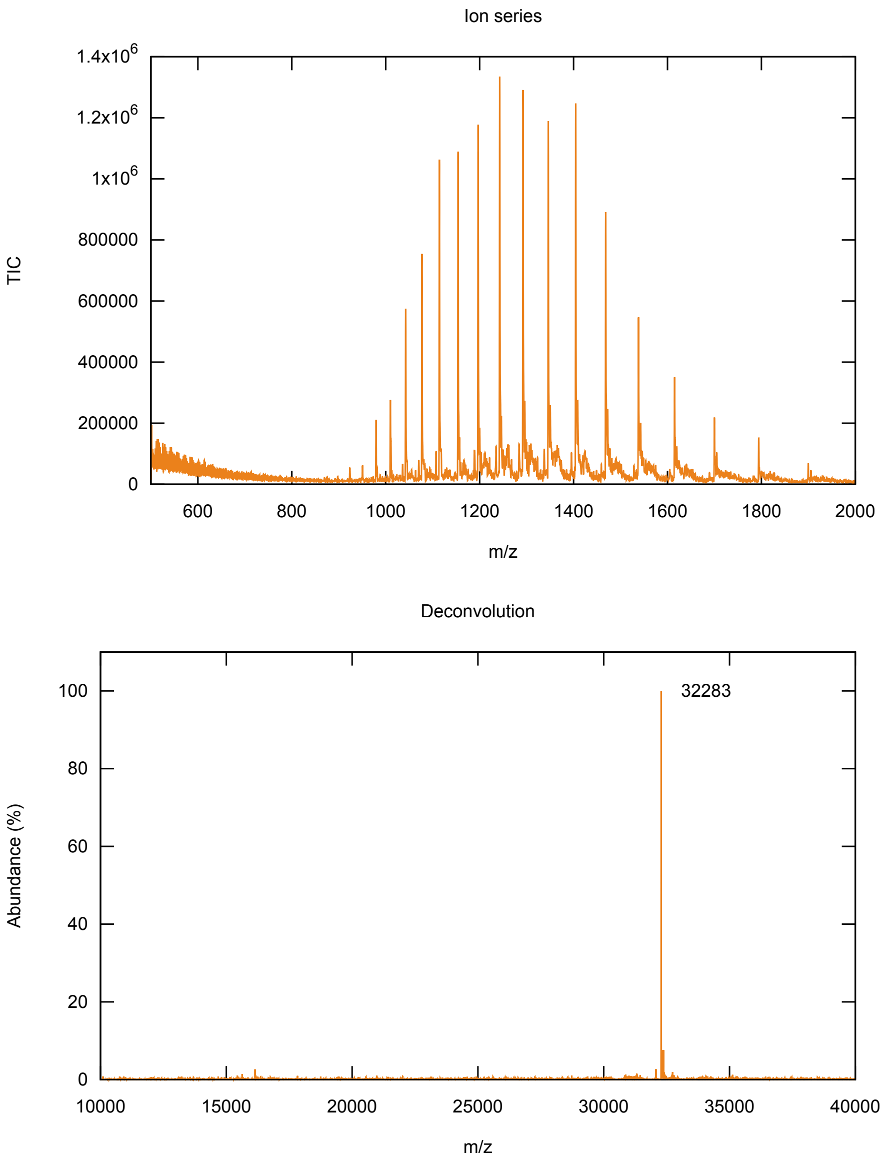

Figure S56: LC-MS spectra of stabilised **anti-HER2/PD-L1 heterodimer** after preparative size exclusion chromatography; ion series and deconvoluted spectra (Calculated mass: 32281 Da).

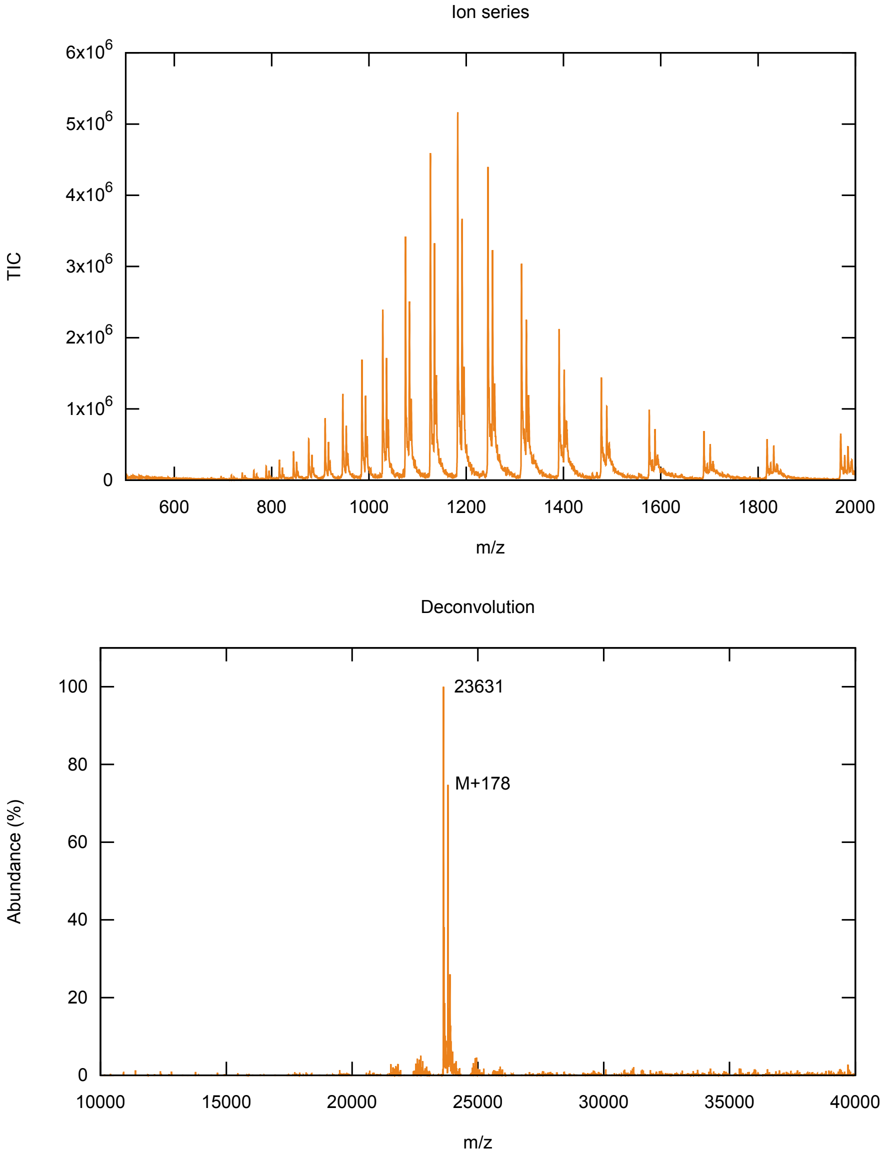

Figure S57: LC-MS spectra of stabilised **anti-HER2 biparatopic heterodimer** after preparative size exclusion chromatography; ion series and deconvoluted spectra (Calculated mass: 23631 Da). M+178 and M+258 Da peaks correspond to partial α-N-6-phosphogluconoylation of the hexahistidine tag.[S9]

3.5 LC–MS analysis of *N*-methyl maleimide capped proteins for BLI

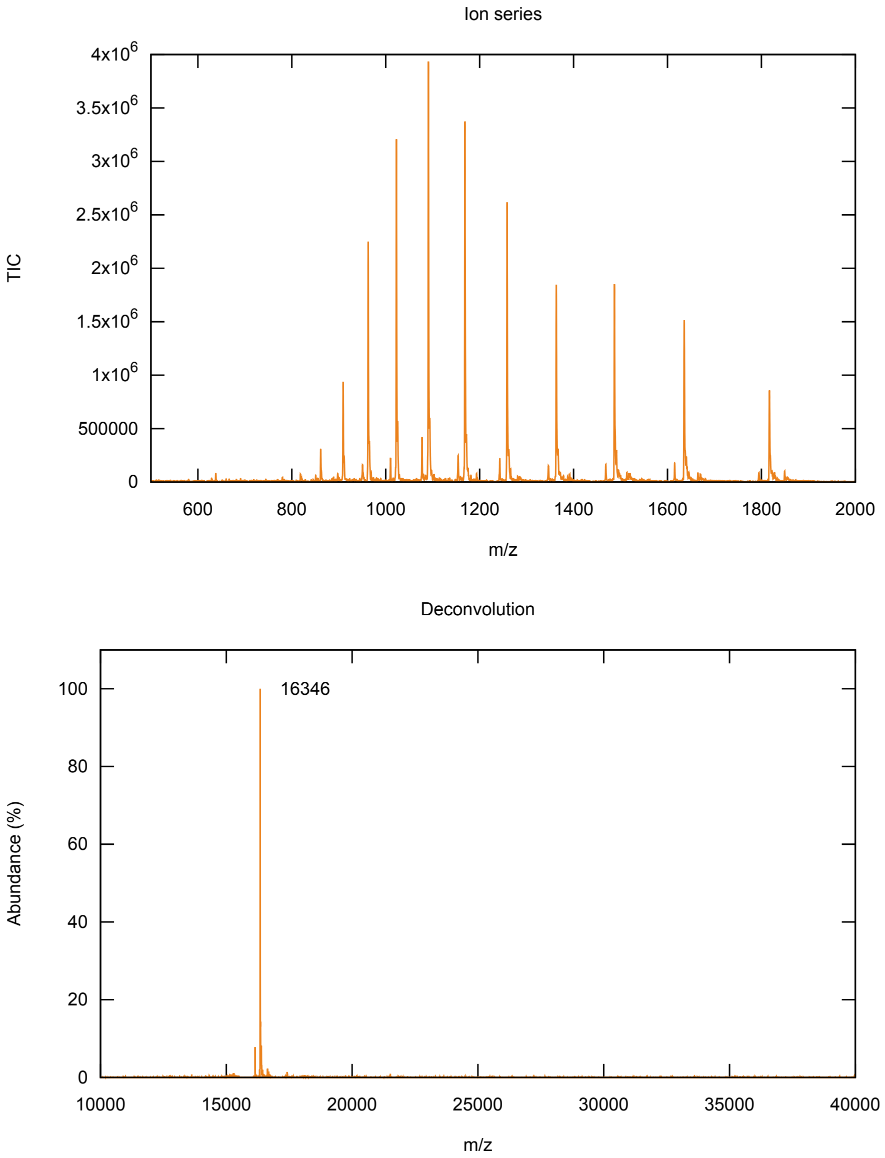

Figure S58: LC-MS spectra of **anti-PD-L1 sdAb** monomer capped by NMM after preparative size exclusion chromatography; ion series and deconvoluted spectra (Calculated mass: 16350 Da).

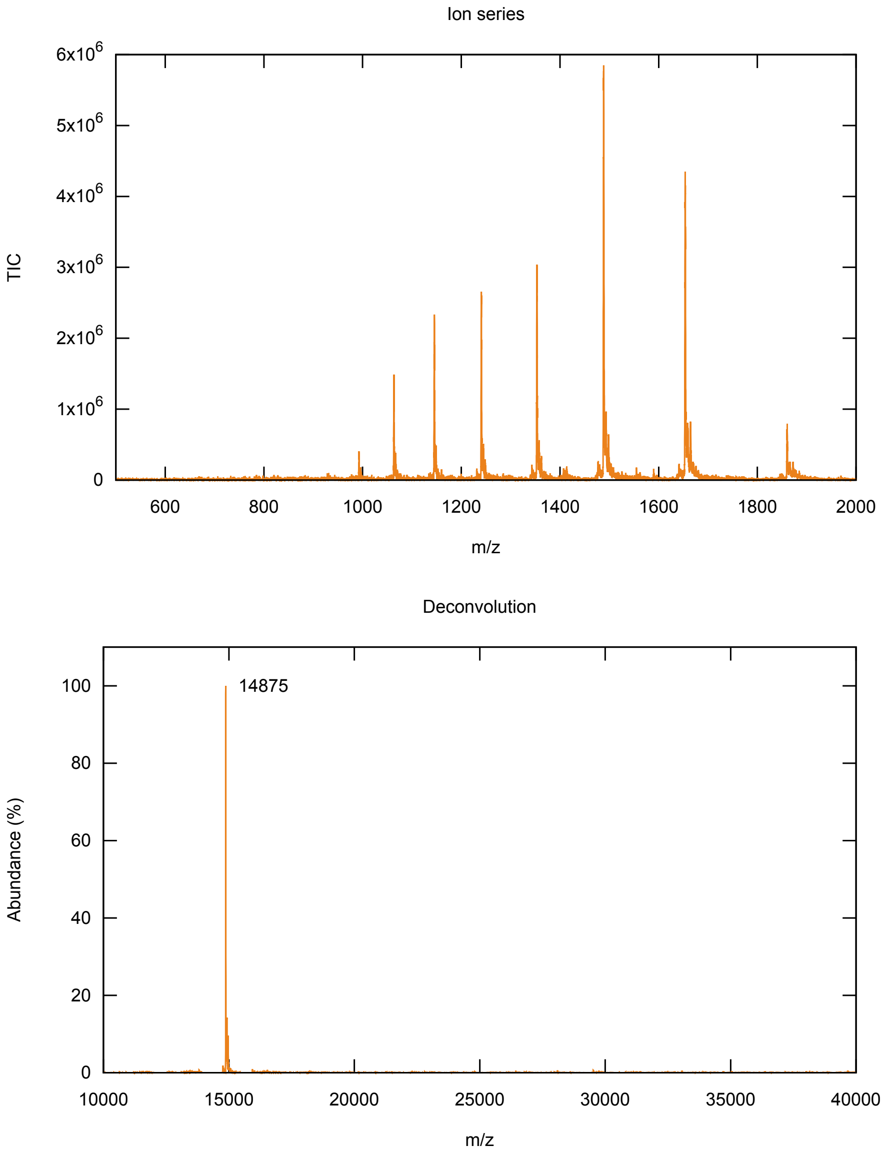

Figure S59: LC-MS spectra of **anti-HER2 sdAb** monomer capped by NMM after preparative size exclusion chromatography; ion series and deconvoluted spectra (Calculated mass: 14879 Da).

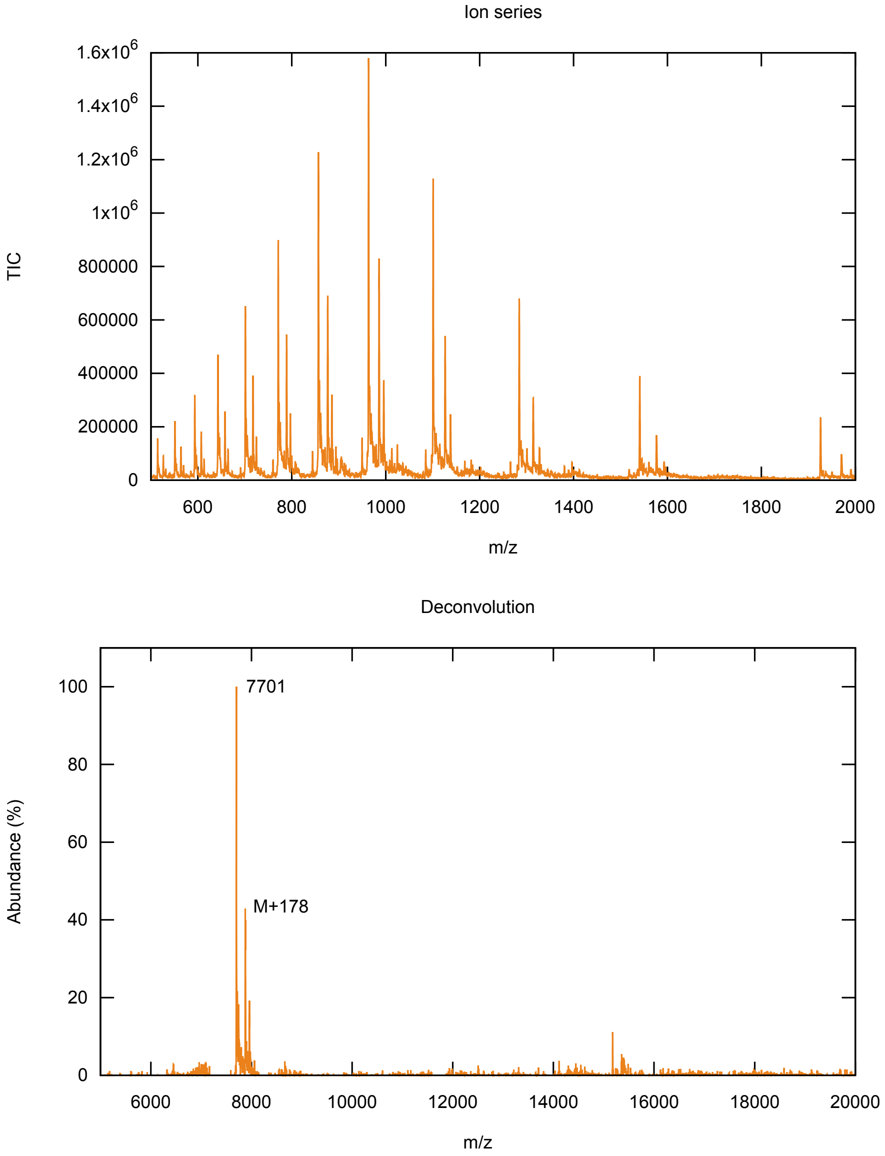

Figure S60: LC-MS spectra of **anti-HER2 affibody** monomer capped by NMM after preparative size exclusion chromatography; ion series and deconvoluted spectra (Calculated mass: 7700 Da). M+178 and M+258 Da peaks correspond to partial α-N-6-phosphogluconoylation of the hexahistidine tag.[S9]

3.6 LC–MS analysis of Alexa Fluor 488 maleimide labelled proteins

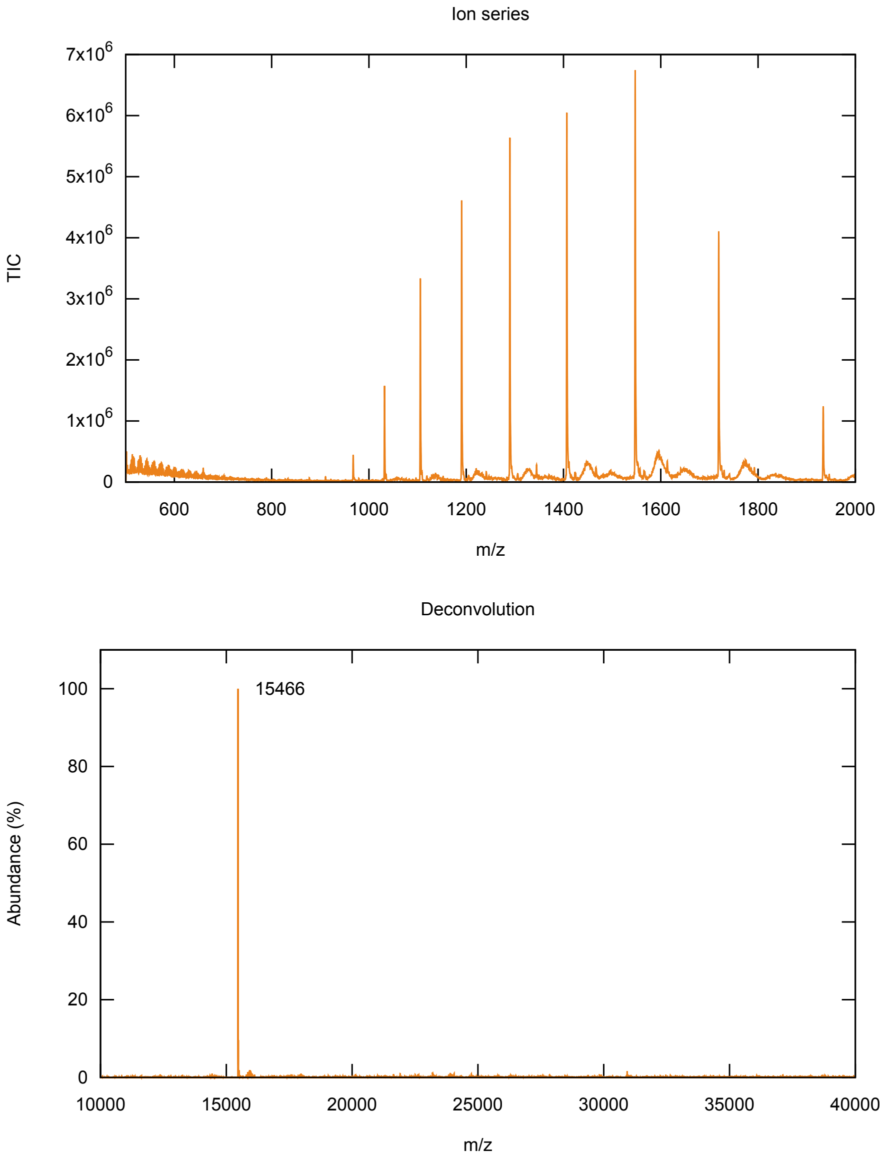

Figure S61: LC-MS spectra of **anti-HER2 sdAb** monomer labelled with Alexa Fluor 488 maleimide after preparative size exclusion chromatography; ion series and deconvoluted spectra (Calculated mass: 15466 Da).

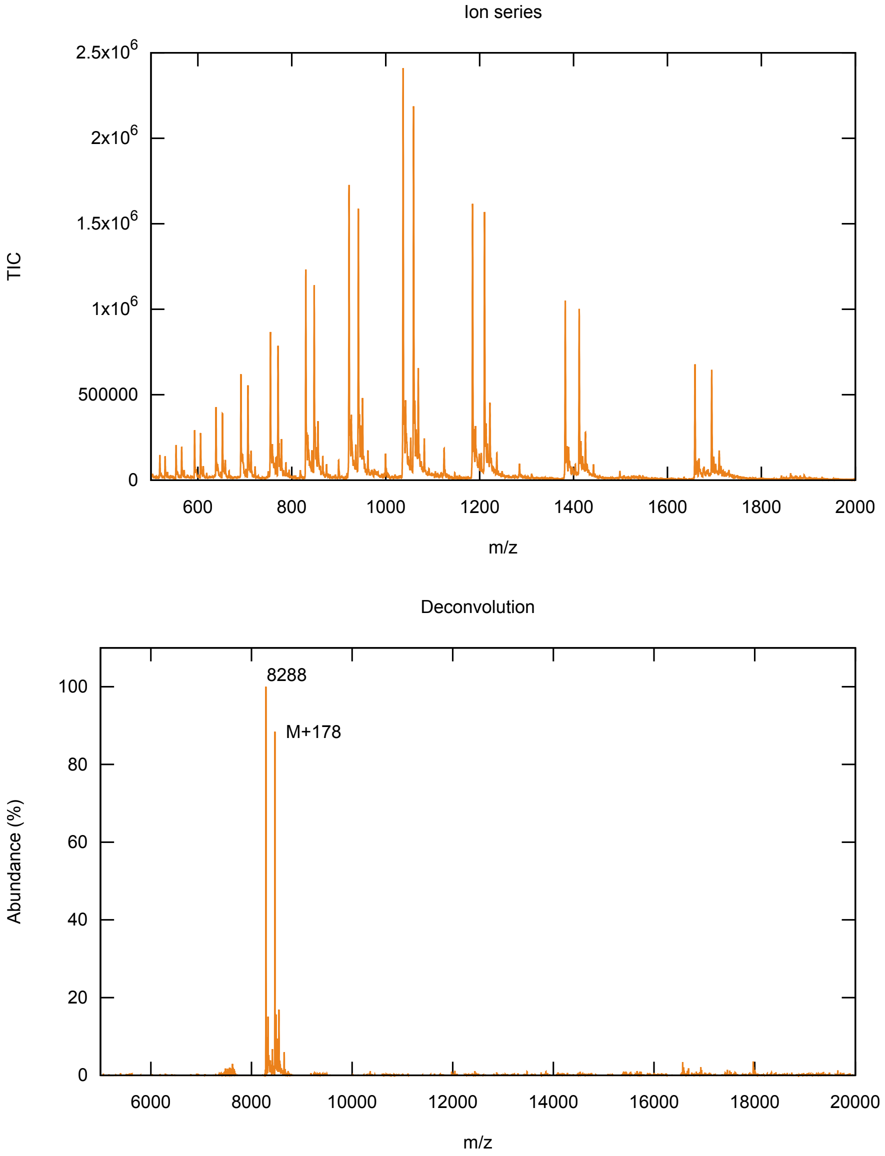

Figure S62: LC-MS spectra of **anti-HER2 affibody** monomer labelled with Alexa Fluor 488 maleimide after preparative size exclusion chromatography; ion series and deconvoluted spectra (Calculated mass: 8287 Da). M+178 and M+258 Da peaks correspond to partial α-N-6-phosphogluconoylation of the hexahistidine tag.[S9]

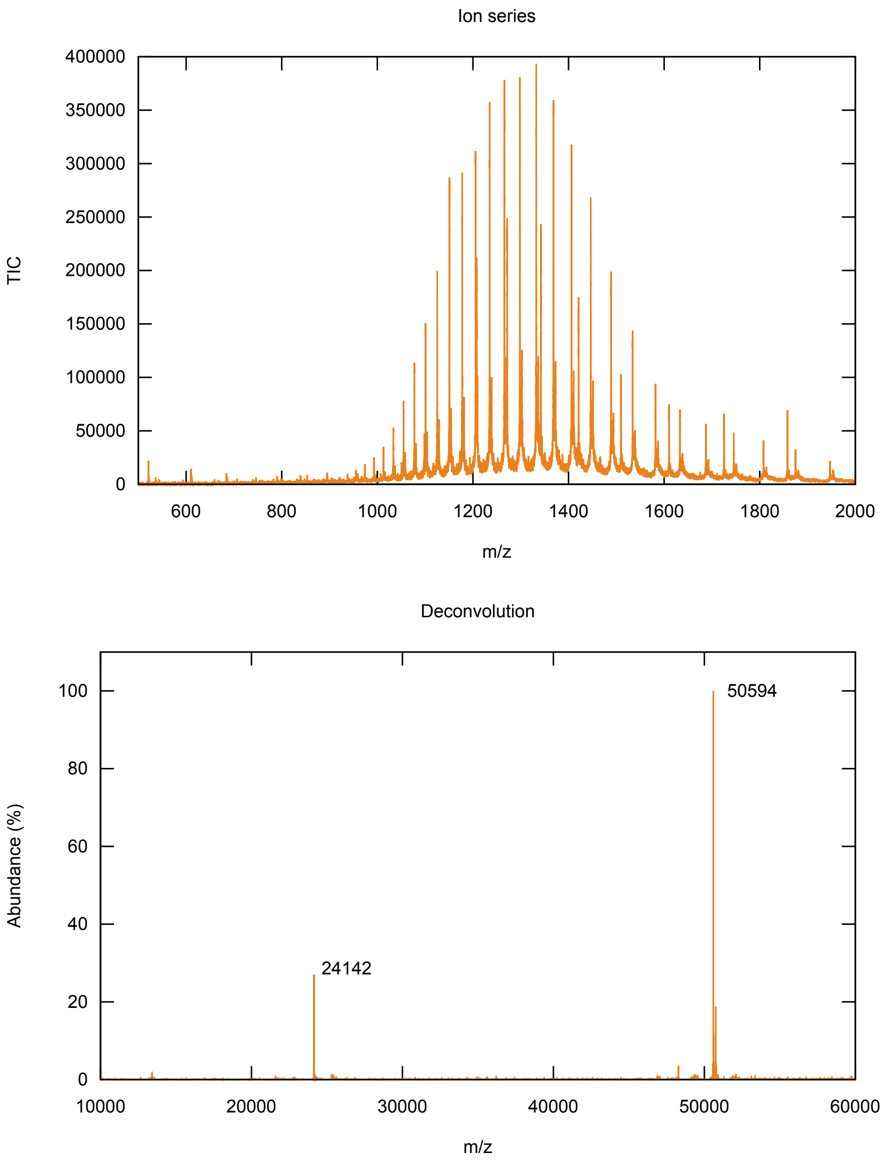

Figure S63: LC-MS spectra of thiomab labelled with Alexa Fluor 488 maleimide after UF/DF; ion series and deconvoluted spectra (Calculated masses: LC: 24140 Da, HC: 50595 Da).

3.7 LC–MS analysis of Alexa Fluor 488 labelled dimers

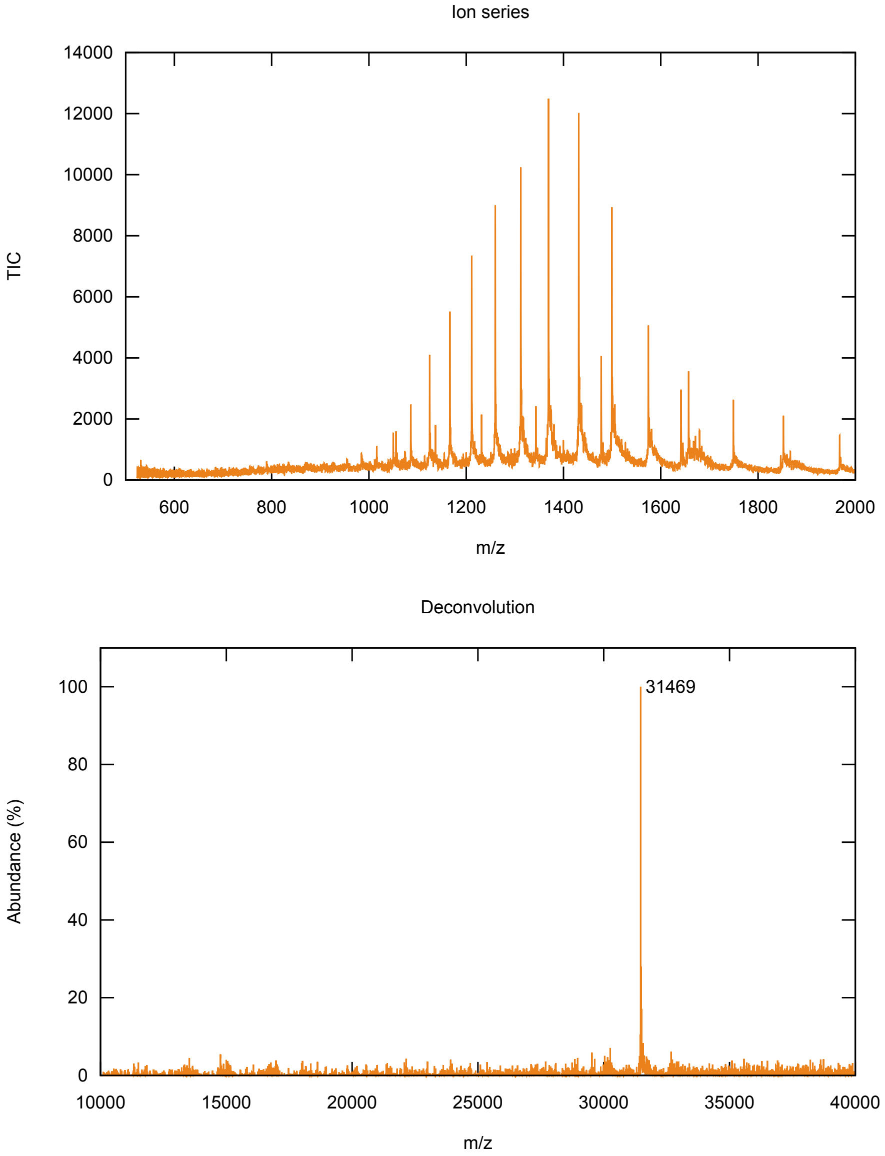

Figure S64: LC-MS spectra of stabilised **anti-HER2 sdAb** homodimer labelled with Alexa Fluor 488 azide after preparative size exclusion chromatography; ion series and deconvoluted spectra (Calculated mass: 31467 Da).

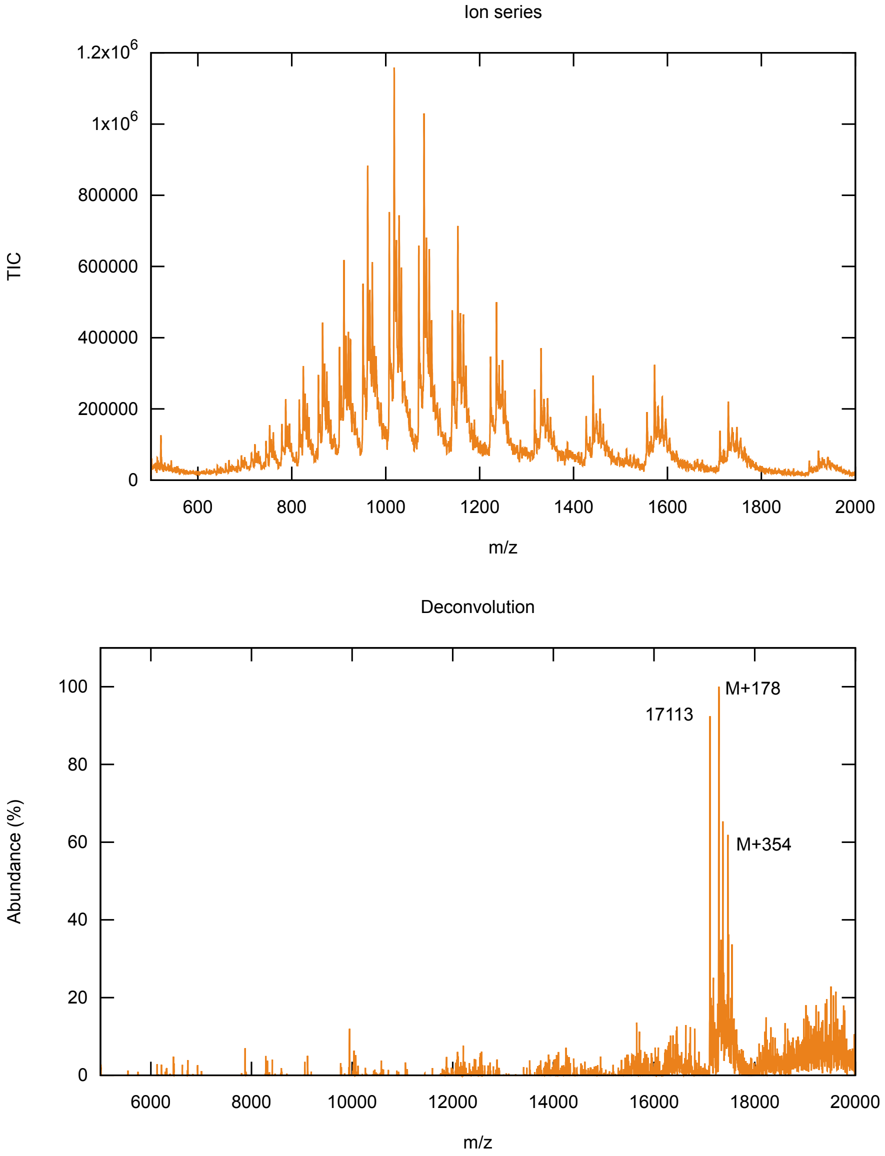

Figure S65: LC-MS spectra of stabilised **anti-HER2 affibody homodimer** labelled with Alexa Fluor 488 azide after preparative size exclusion chromatography; ion series and deconvoluted spectra (Calculated mass: 17109 Da). M+178, M+258, M+356 and M+516 Da peaks correspond to partial α-N-6-phosphogluconoylation of the hexahistidine tag.[S9]

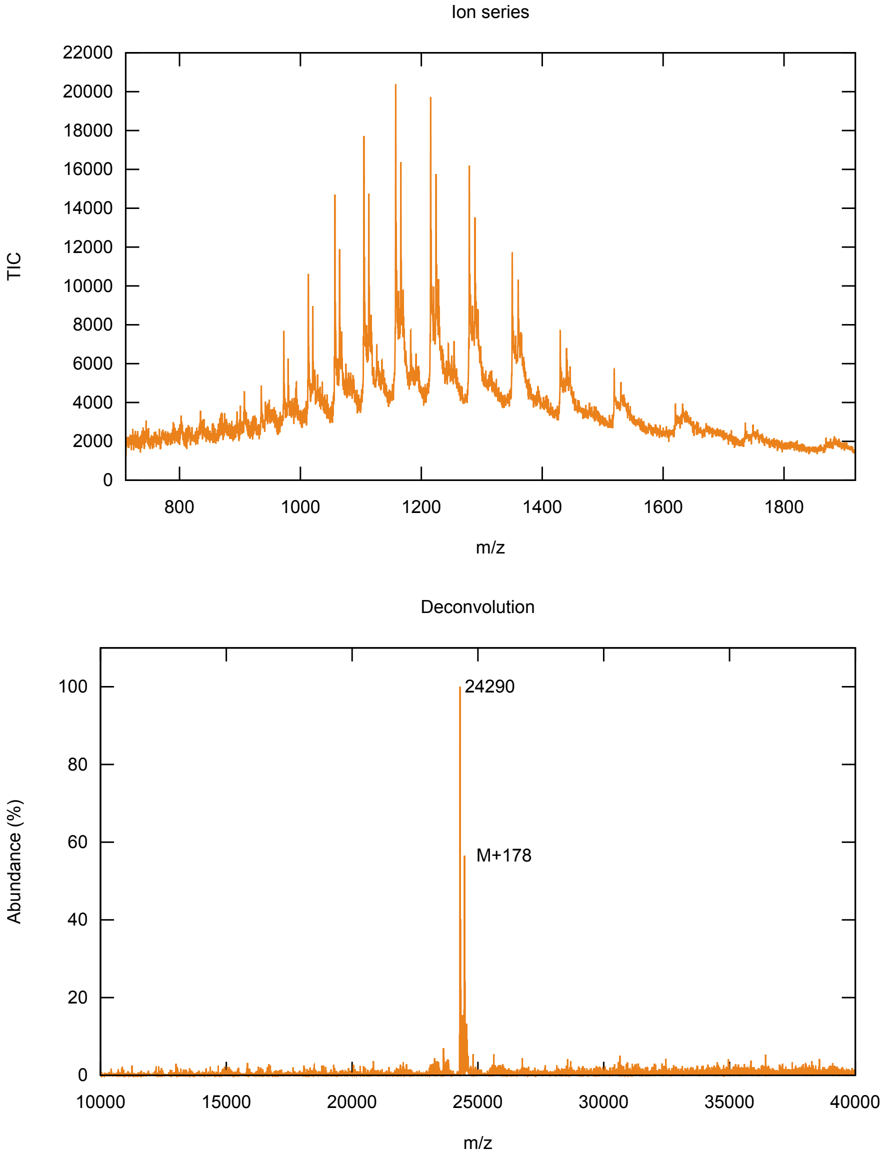

Figure S66: LC-MS spectra of stabilised **anti-HER2 biparatopic heterodimer** labelled with Alexa Fluor 488 azide after preparative size exclusion chromatography; ion series and deconvoluted spectra (Calculated mass: 24288 Da). M+178 and M+258 Da peaks correspond to partial α-N-6-phosphogluconoylation of the hexahistidine tag.[S9]

3.8 Thiol-based trimerisation LC–MS

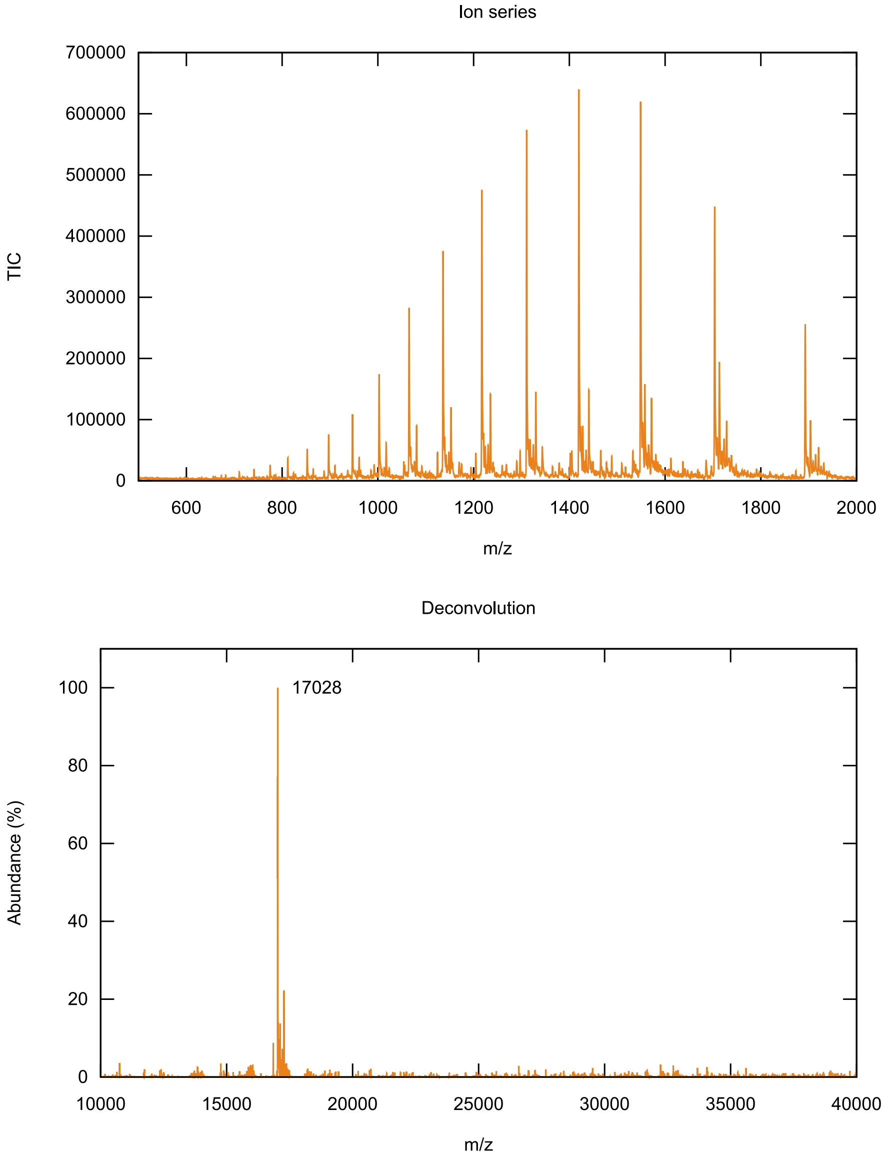

Figure S67: LC–MS spectra of **anti-CD3/Linker III** monomer after preparative SEC; ion series and deconvoluted spectra (Calculated mass: 17029 Da).

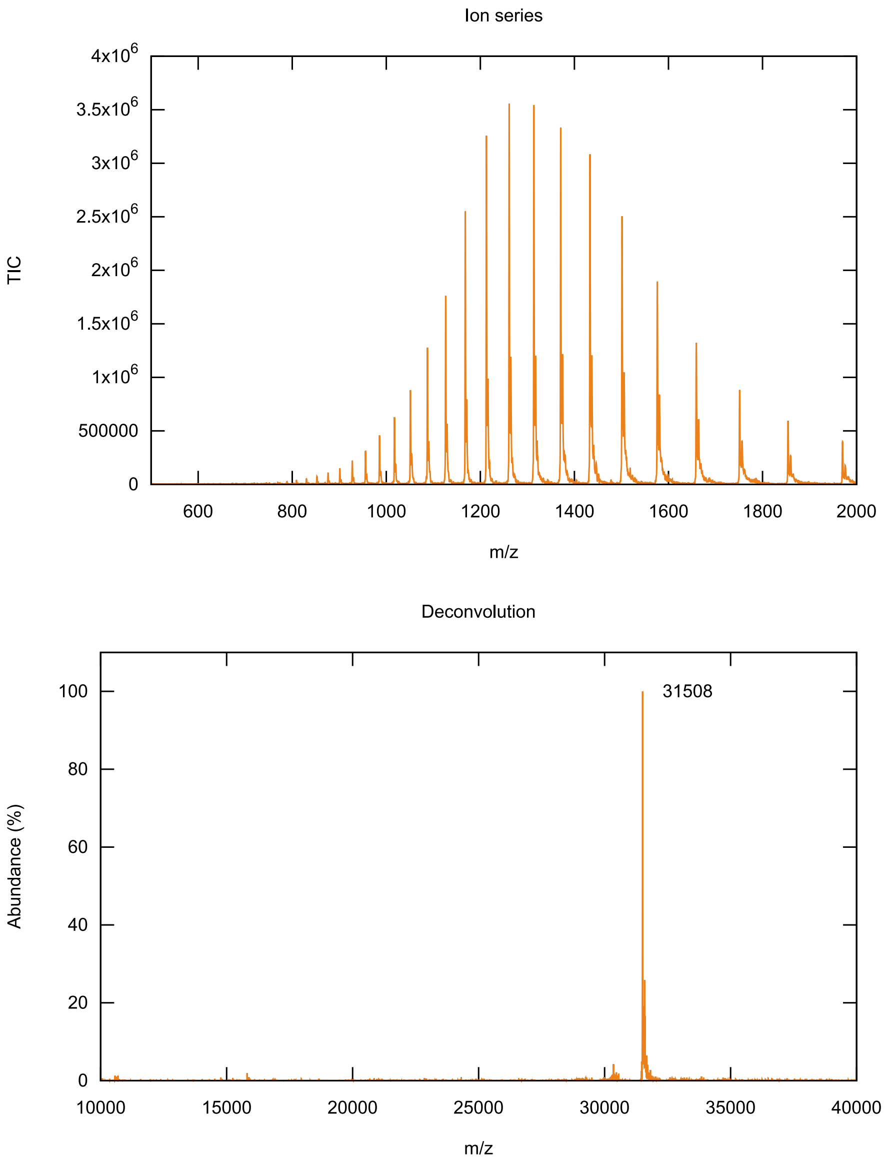

Figure S68: LC–MS spectra of **anti-HER2 sdAb homodimer** after preparative SEC purification; ion series and deconvoluted spectra (Calculated mass: 31513 Da).

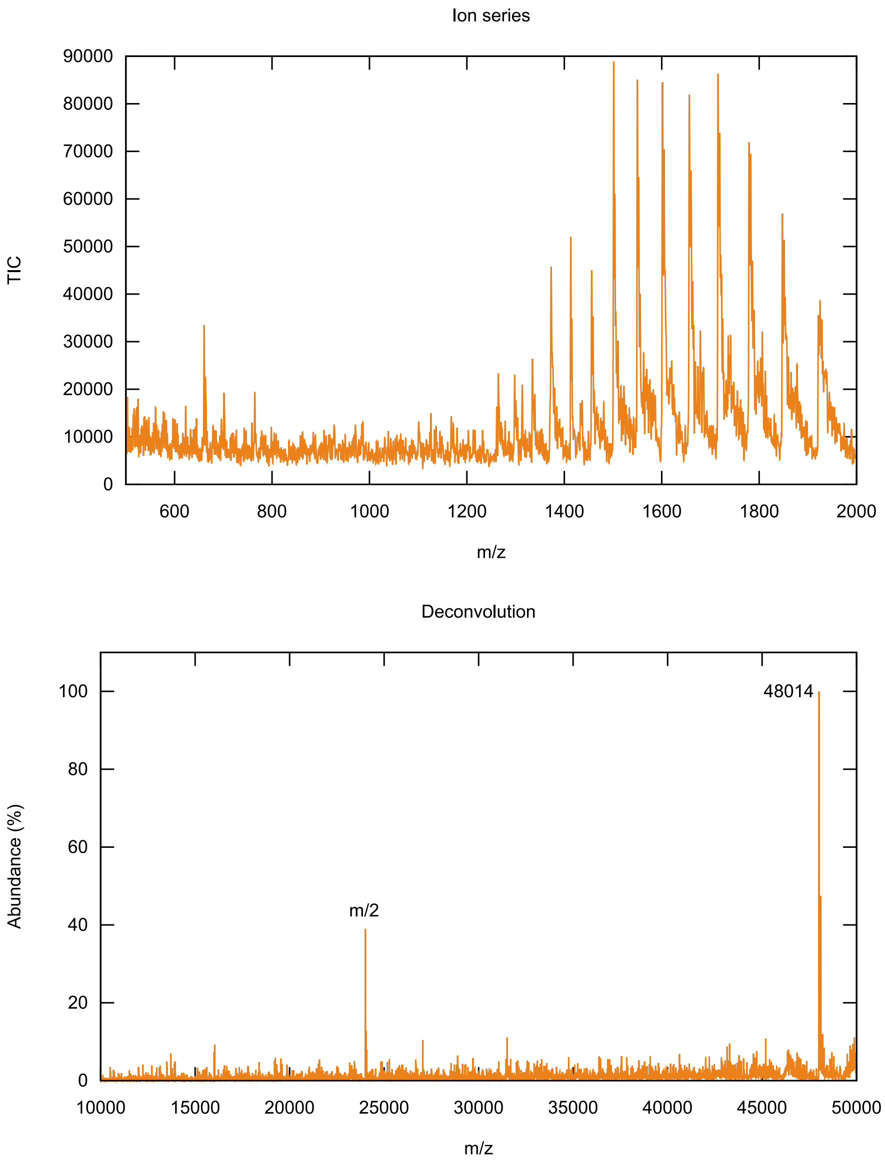

Figure S69: LC–MS spectra of **anti-HER2/CD3 trimer** after hydrolytic stabilisation and preparative SEC purification; ion series and deconvoluted spectra (Calculated mass: 48017 Da).

3.9 Tetramerisation LC–MS

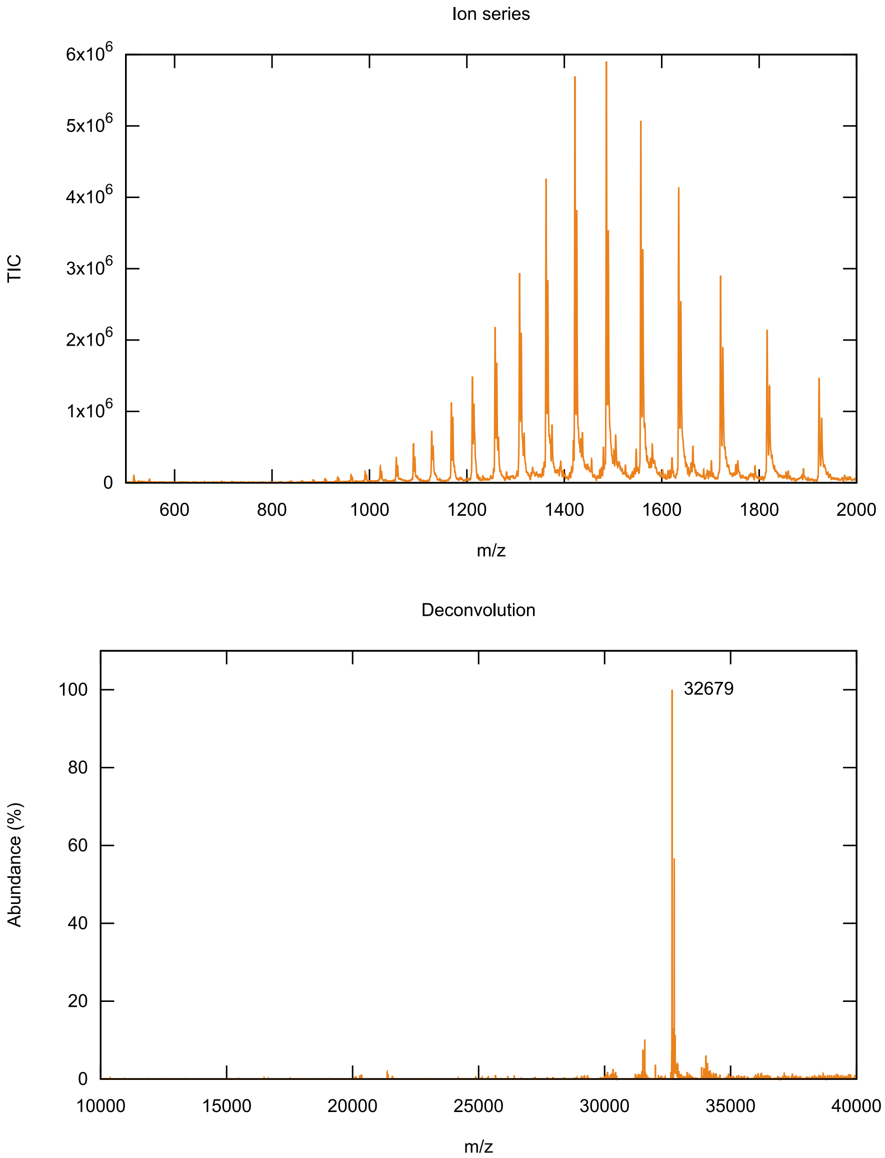

Figure S70: LC–MS spectra of **anti-HER2 sdAb homodimer–Linker II** after cation exchange purification; ion series and deconvoluted spectra (Calculated mass: 32684 Da).

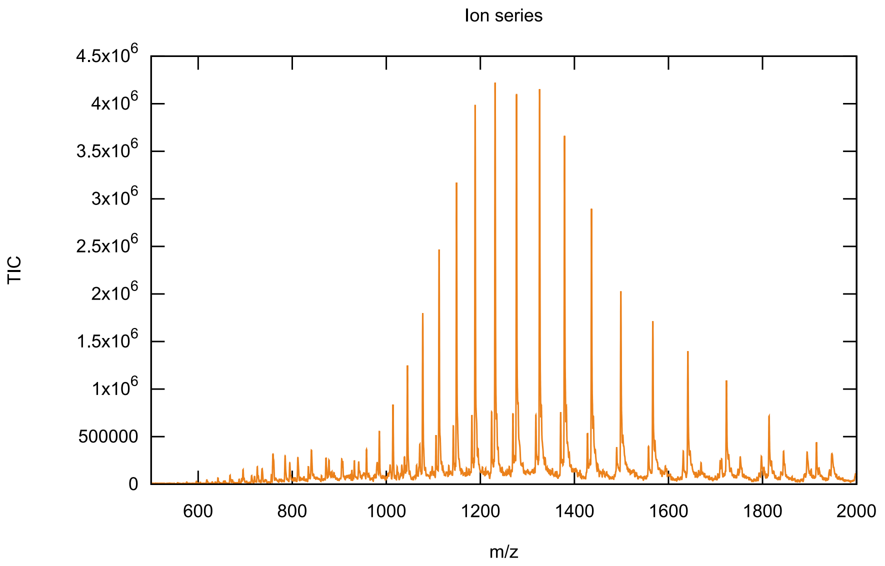

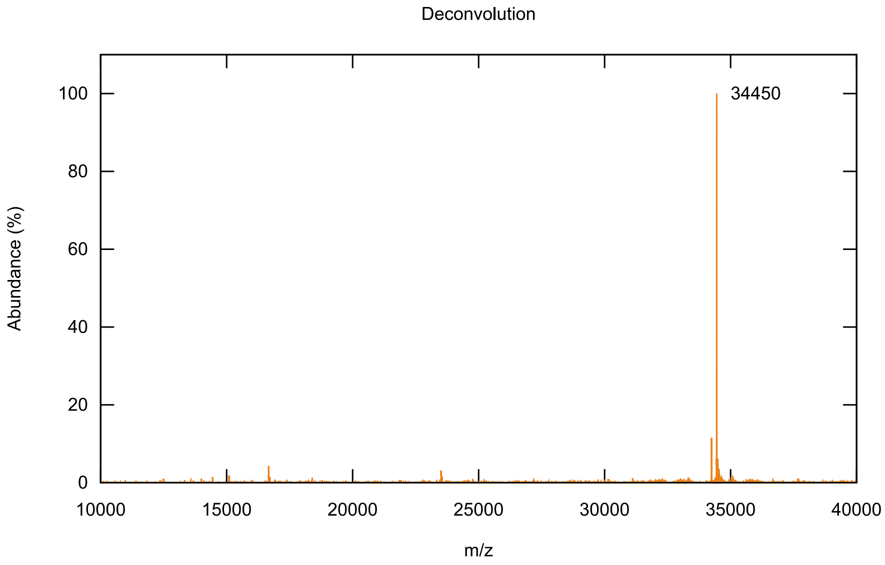

Figure S71: LC–MS spectra of **anti-PD-L1 homodimer** after preparative SEC purification; ion series and deconvoluted spectra (Calculated mass: 34455 Da).

3.10 DBCO-based trimerisation LC–MS

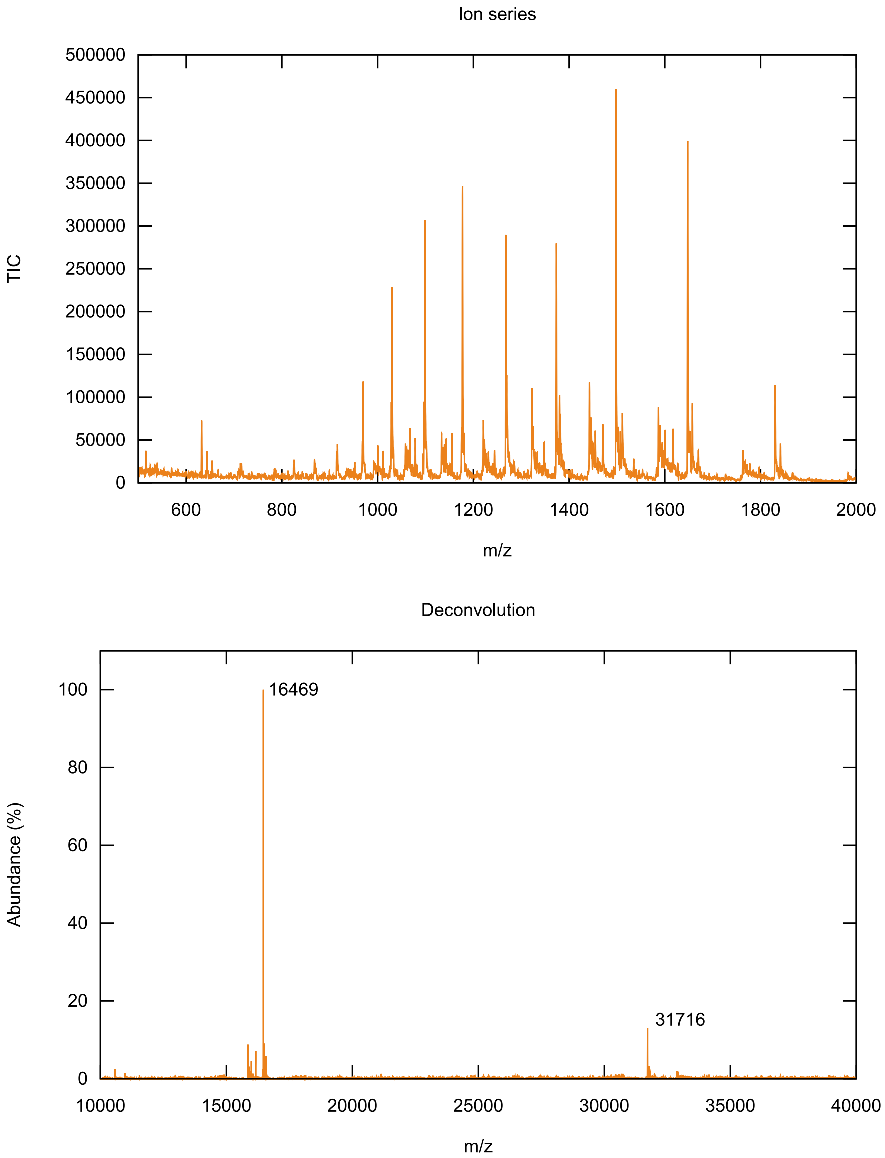

Figure S72: LC–MS spectra of **anti-CD3 sdAb** modified with bromoacetamido-PEG-11-azide (Calculated mass: 16470 Da). The peak at 31716 Da corresponds to intermolecular disulfide mediated dimerisation of the **anti-CD3 sdAb**.

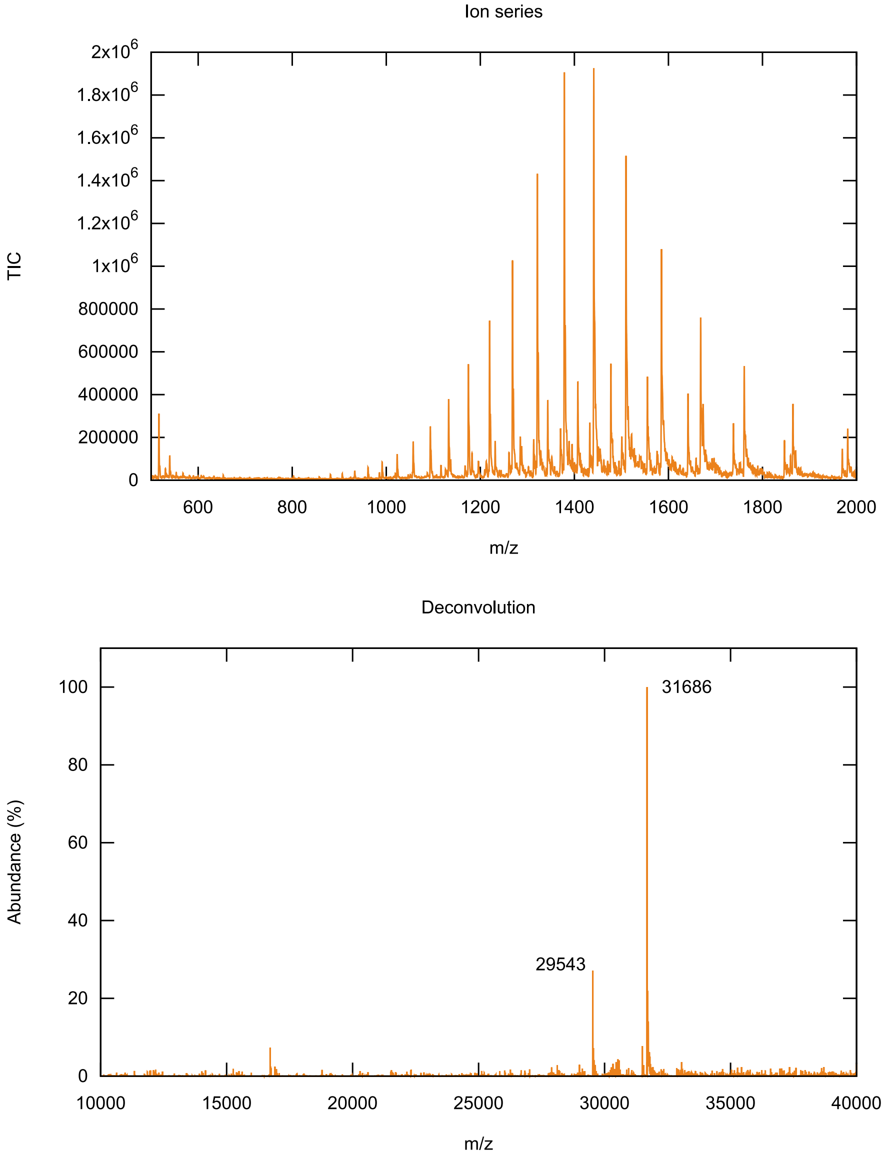

Figure S73: LC–MS spectra of **anti-HER2 sdAb homodimer** generated from **Linker V** after preparative SEC; ion series and deconvoluted spectra (Calculated mass: 31685 Da). The peak at 29543 Da corresponds to intermolecular disulfide mediated dimerisation of the **anti-HER2 sdAb**, as SEC was carried out in the absence of reducing agent.

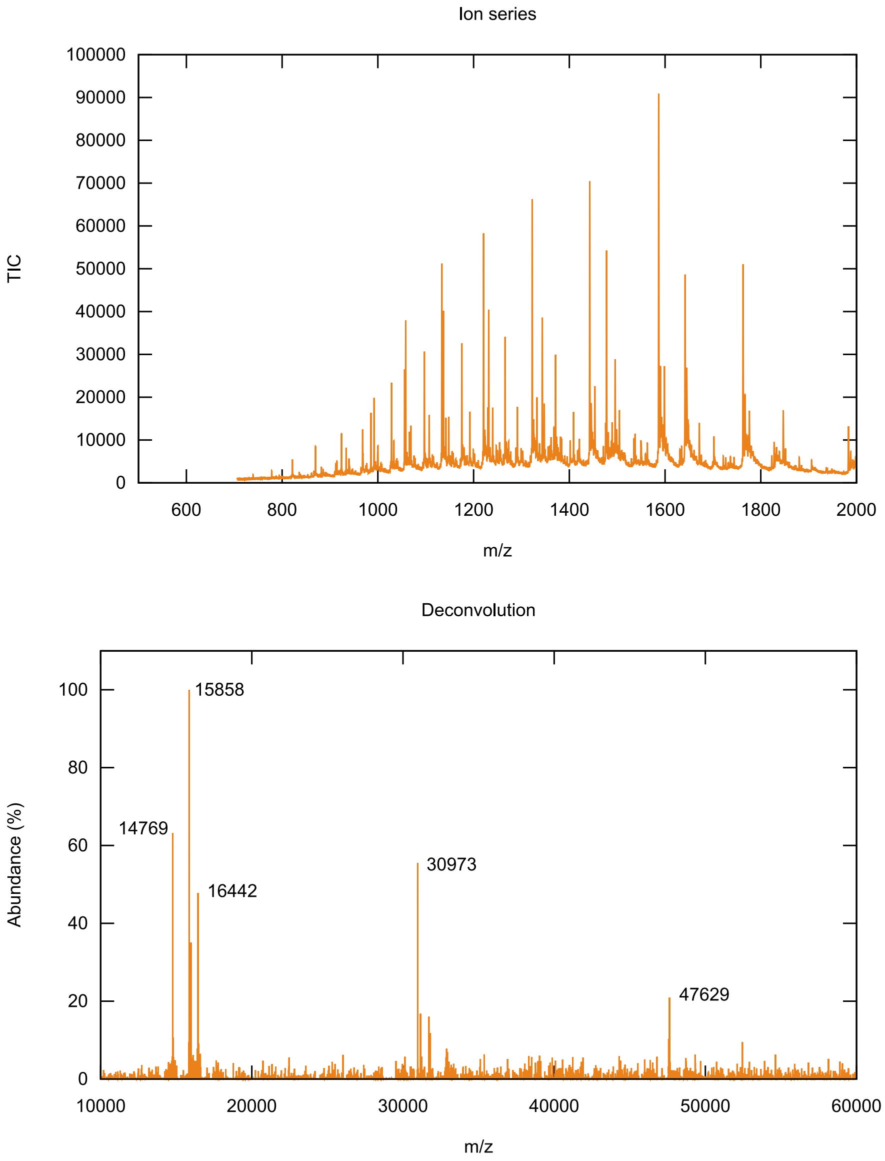

Figure S74: LC–MS spectra of **anti-HER2/CD3 sdAb trimer** after reductively triggered hydrolytic stabilisation; ion series and deconvoluted spectra (Calculated mass: 47629 Da). The other peaks corresponded to the following species species, 14769 Da: reduced intermolecular disulfide-dimerised **anti-HER2 sdAb**, 15858 Da: 16442 Da: TCEP reduced **anti-CD3 sdAb–azide** conjugate (-N_2), 16442 Da: unreactive, reduced **anti-CD3 sdAb–azide** conjugate (-N_2), and 30973 Da: hydrolytically decomposed DBCO, rendering the **anti-HER2 sdAb homodimer** unreactive in the context of SPAAC (Figure S33).

3.11 LC–MS spectra of anti-PD-L1 nanobody–Thiomab conjugates

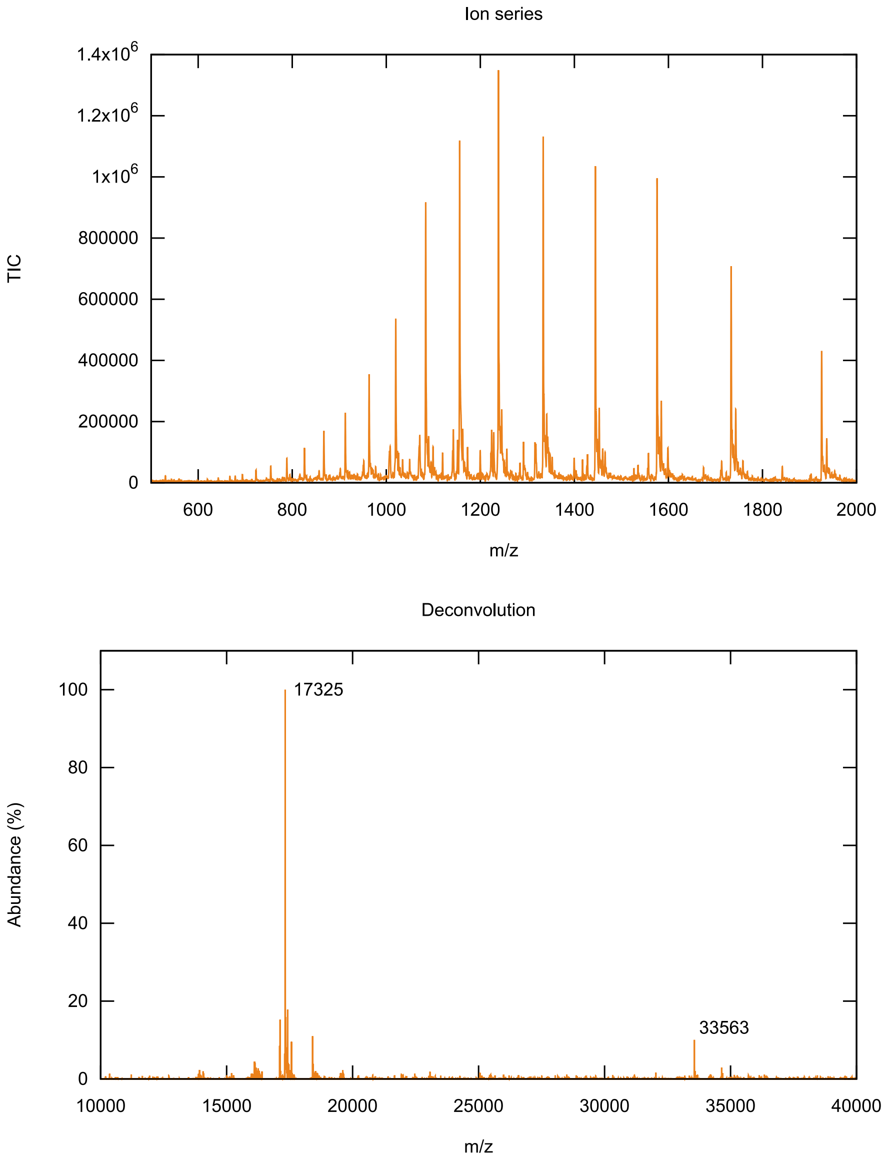

Figure S75: LC–MS spectra of **anti-PD-L1 sdAb/Linker IV** intermediate after desalting; ion series and deconvoluted spectra (Calculated mass: 17325 Da).

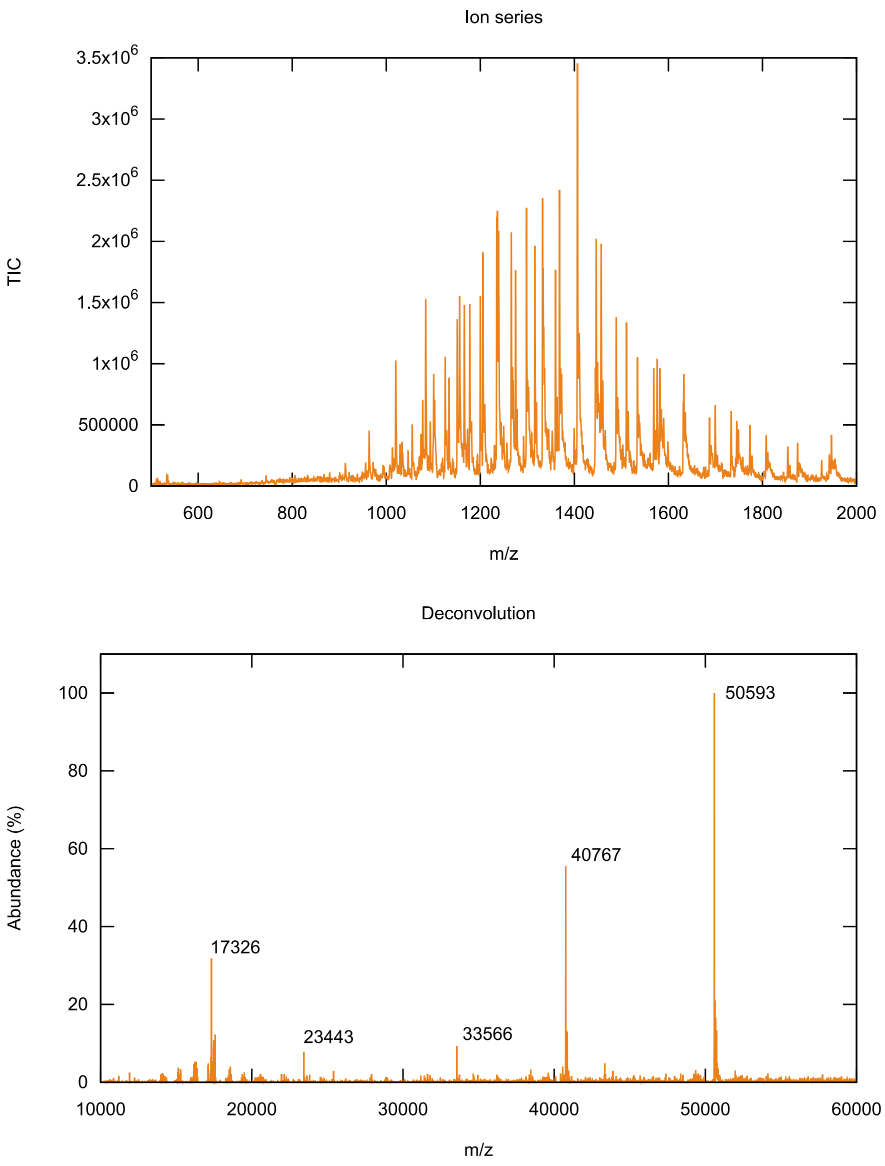

Figure S76: LC–MS spectrum of **anti-PD-L1 sdAb** conjugated to the LC of thiomab prior to UV-triggered hydrolytic stabilisation; ion series and deconvoluted spectra (Calculated masses: LC: 40767 Da, HC: 50595 Da). The peak at 17326 Da corresponds to **anti-PD-L1 sdAb/Linker IV** intermediate; 23443 Da to unmodified thiomab LC; and 33566 Da to **anti-PD-L1 homodimer** formed via **Linker IV**.

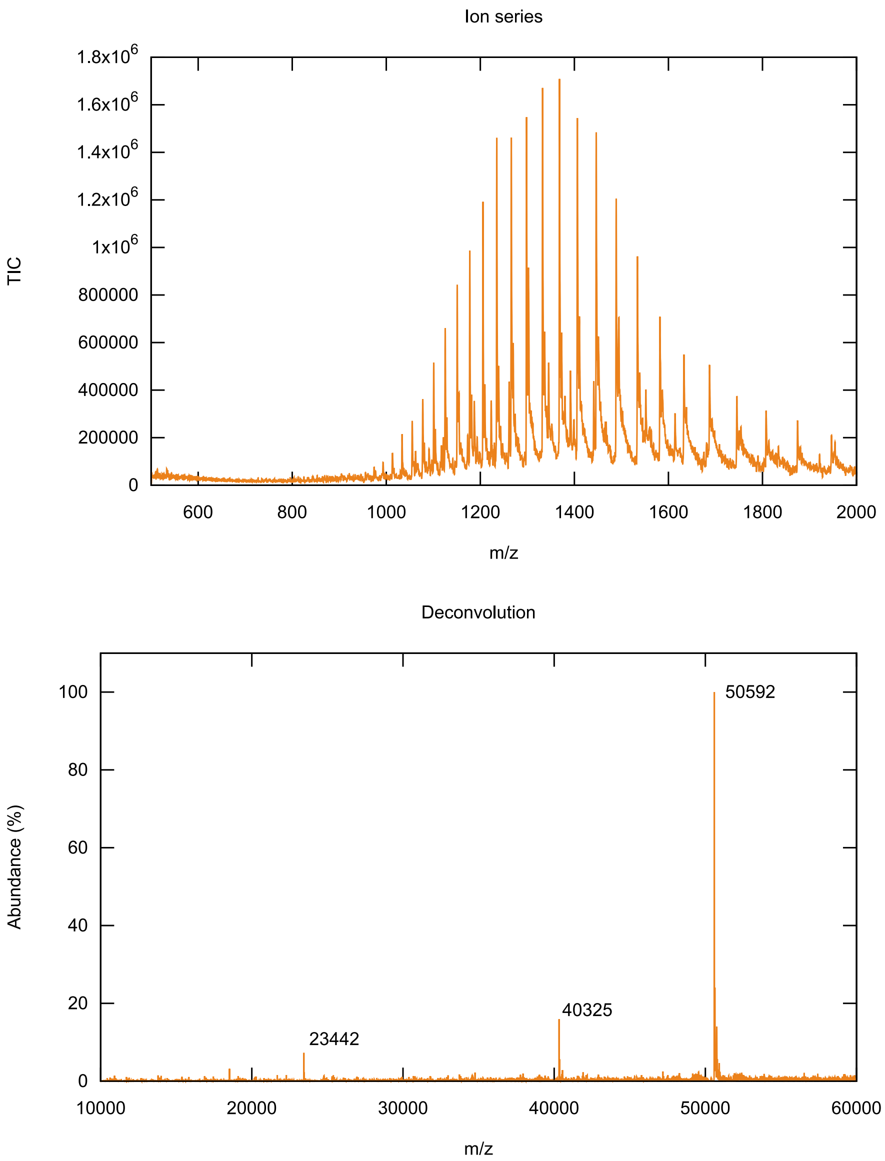

Figure S77: LC–MS spectra of **anti-PD-L1 sdAb** conjugated to the LC of thiomab after UV-triggered hydrolytic stabilisation and purification by UF/DF; ion series and deconvoluted spectra (Calculated masses: LC: 40325 Da, HC: 50595 Da). The peak at 23442 Da corresponds to unmodified LC.

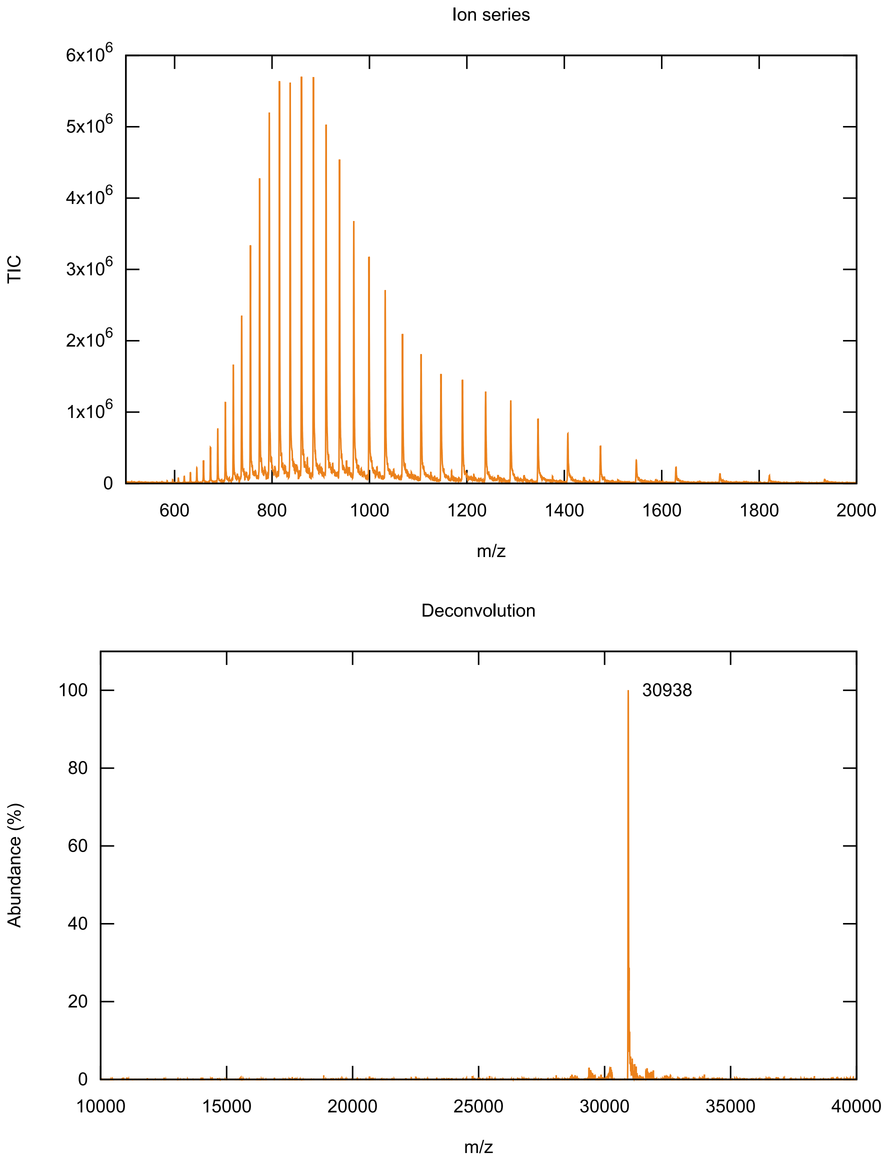

Figure S78: LC–MS spectrum of Cysteine–tagged EGFP; ion series and deconvoluted spectra (Calculated mass: 30953).

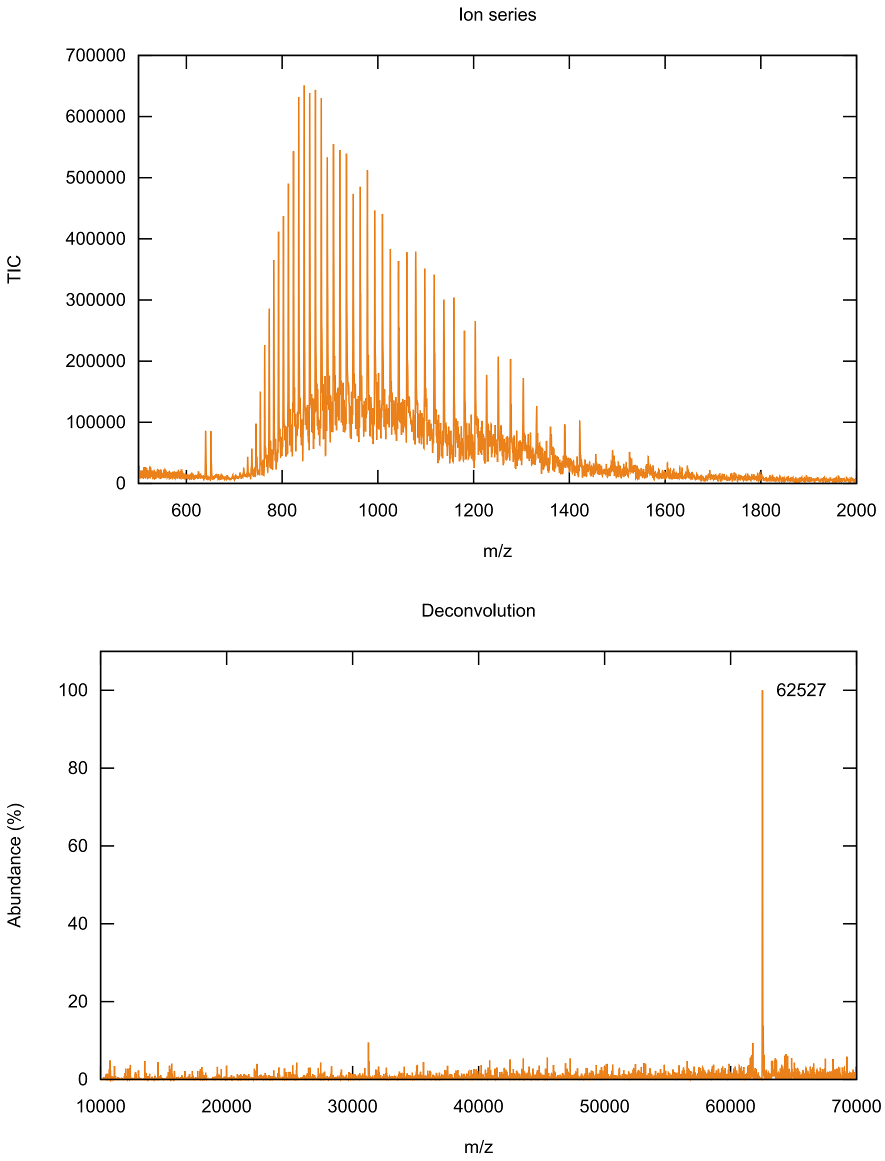

Figure S79: LC–MS spectrum of **EGFP homodimer** after immolation and SEC purification; ion series and deconvoluted spectra (Calculated mass: 62551).

4 SEC purification

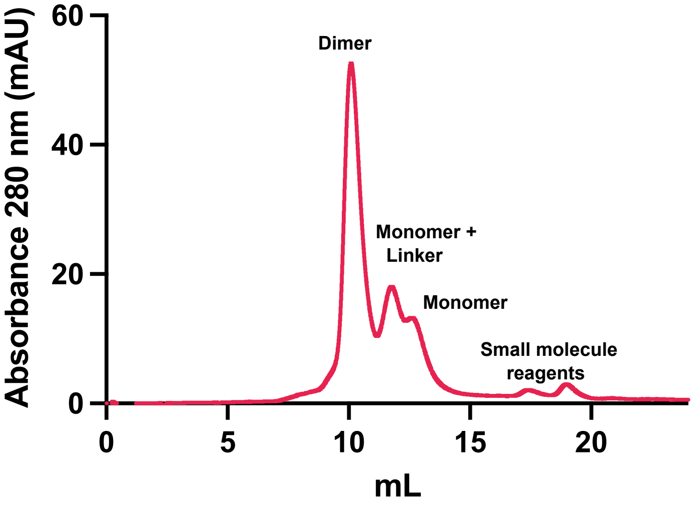

Figure S80: A representative size exclusion chromatogram for the purification of dimers from monomeric species of **anti-PD-L1 homodimer**.

5 ^{1}H and ^{13}C NMR spectra

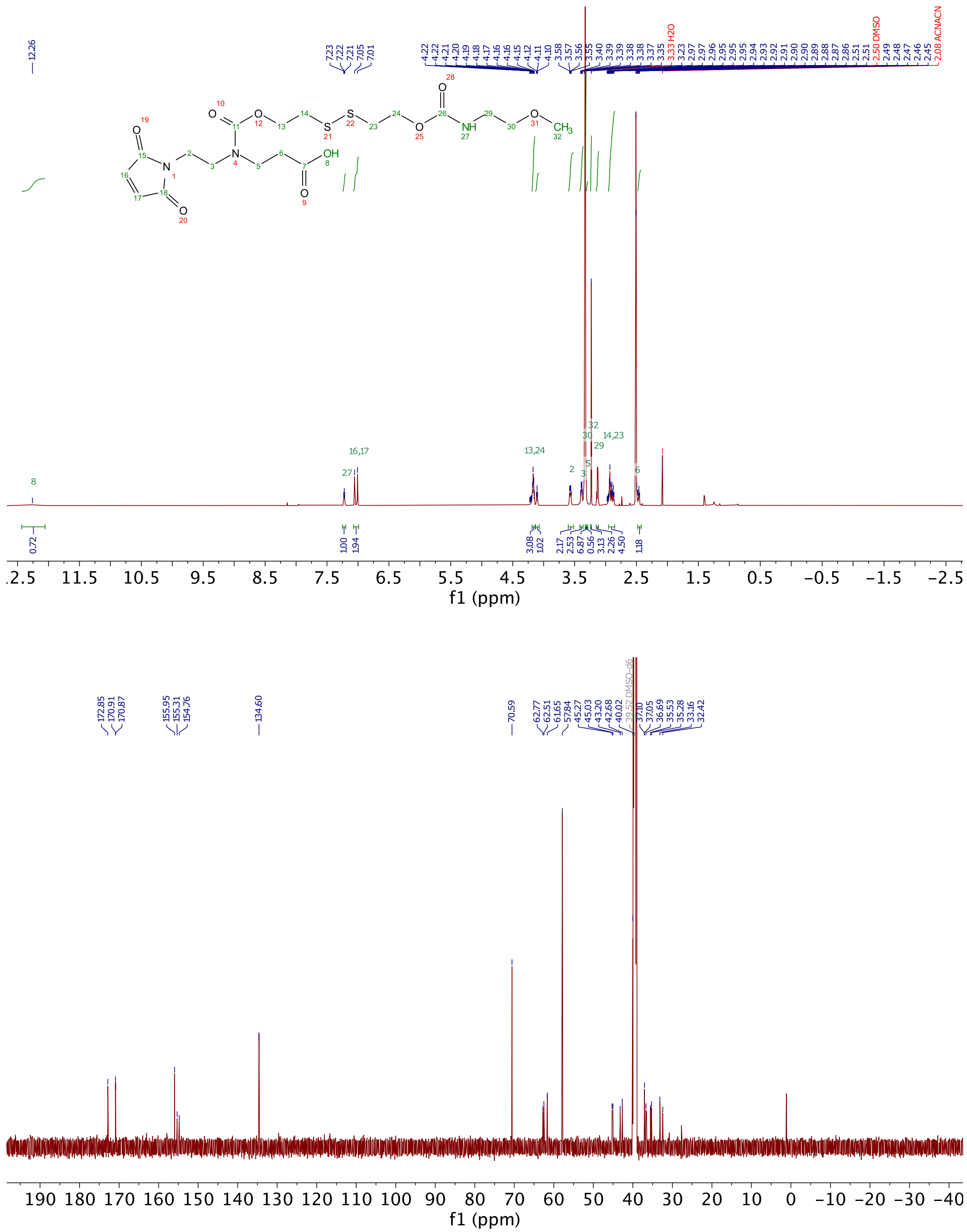

Figure S81: ^{1}H and ^{3}C NMR spectra of maleimide **5**.

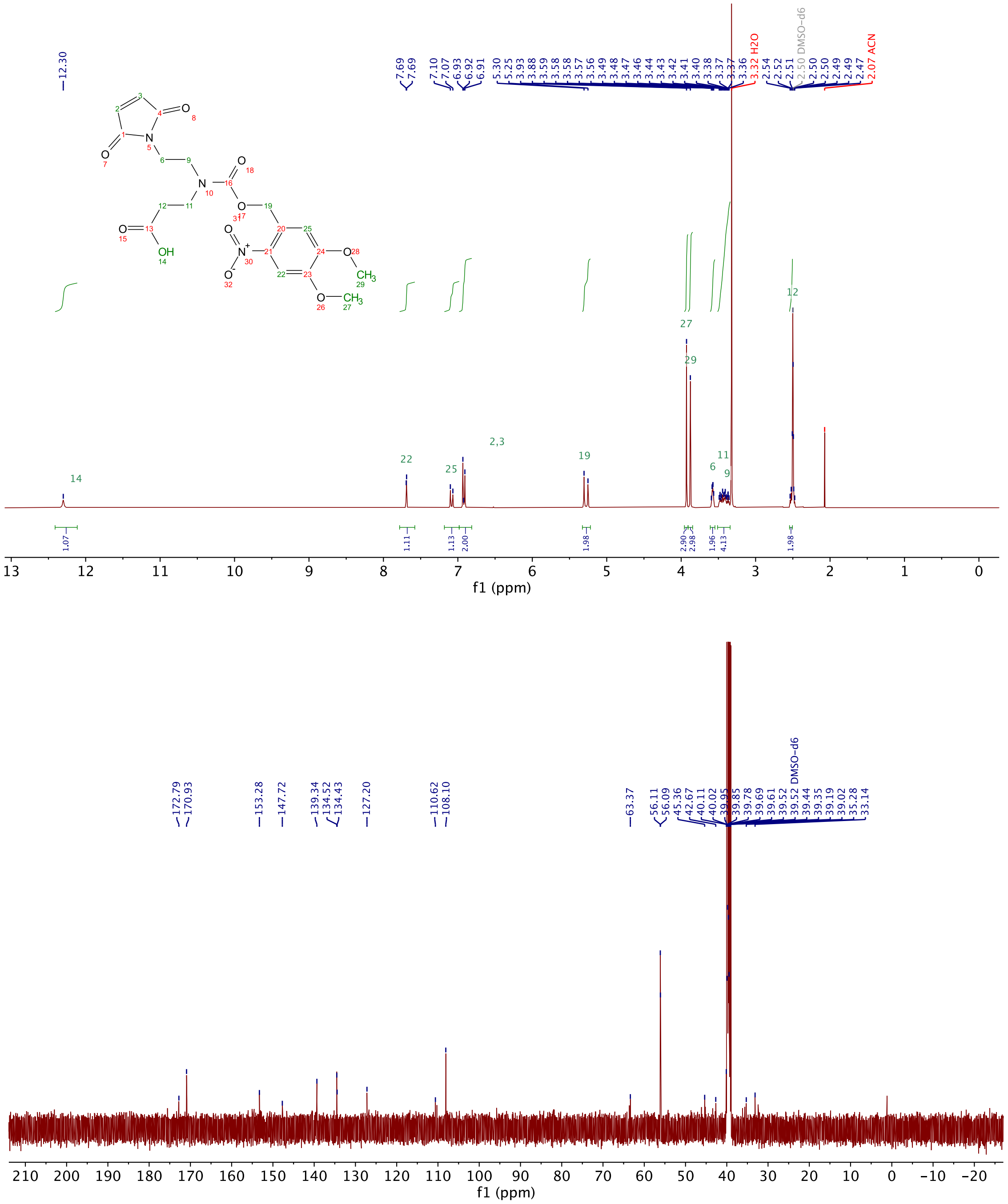

Figure S82: ^{1}H and ^{3}C NMR spectra of maleimide **6**.

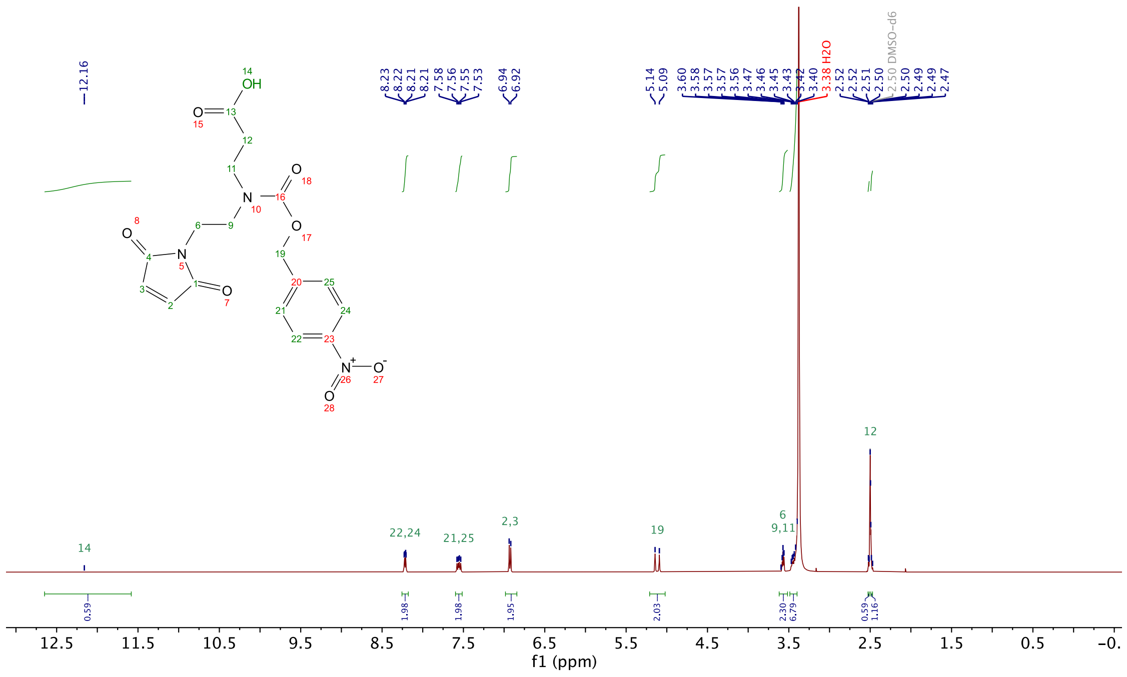

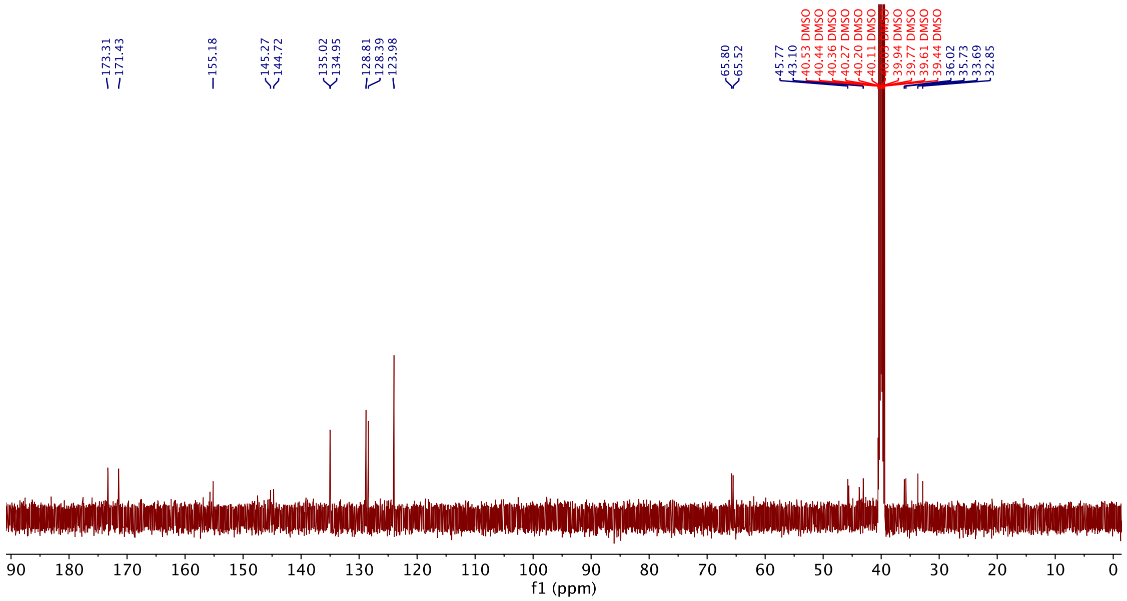

Figure S83: ^{1}H and ^{3}C NMR spectra of maleimide **7** at 298 K.

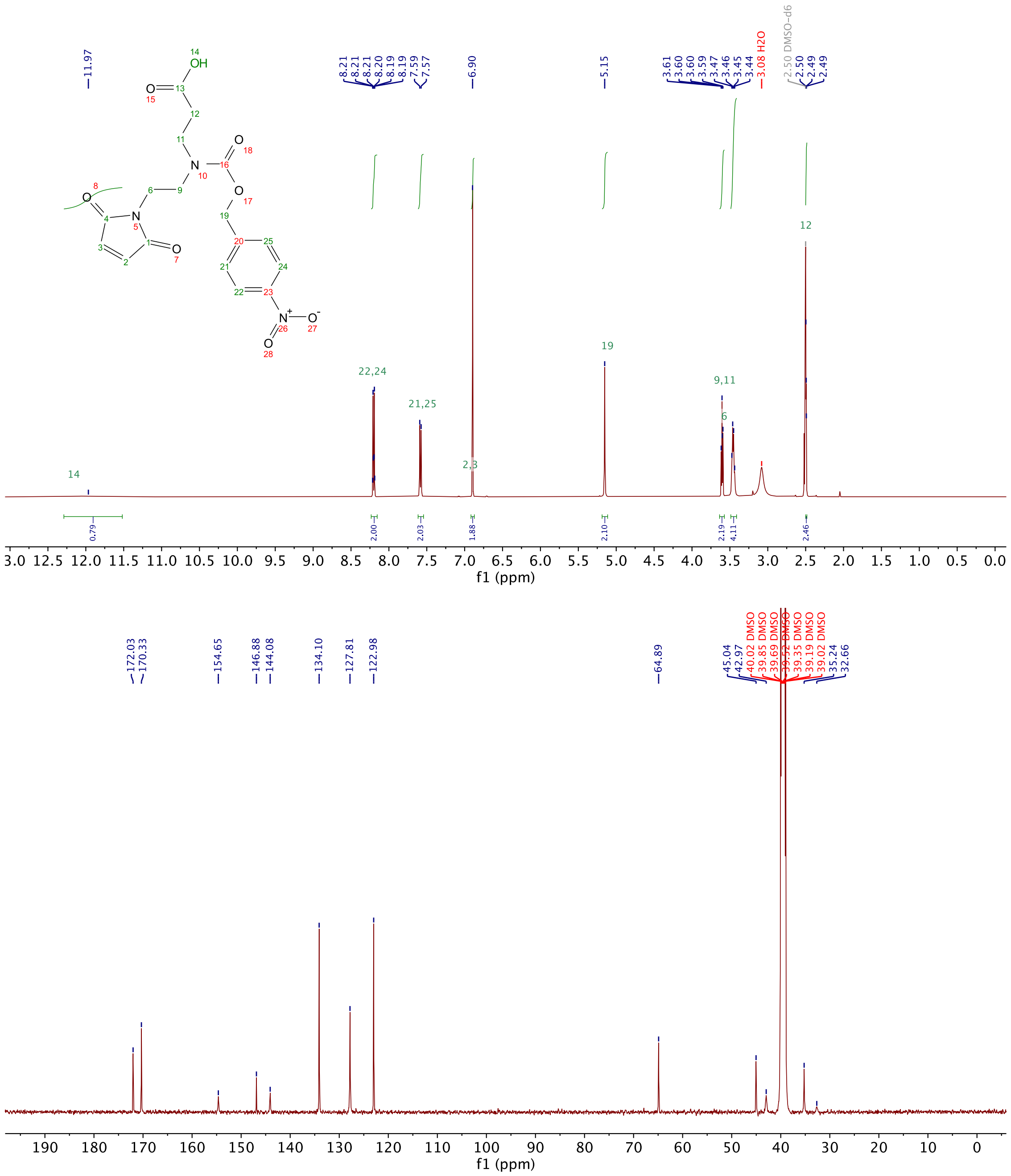

Figure S84: ^{1}H and ^{3}C NMR spectra of maleimide **7** at 353 K.

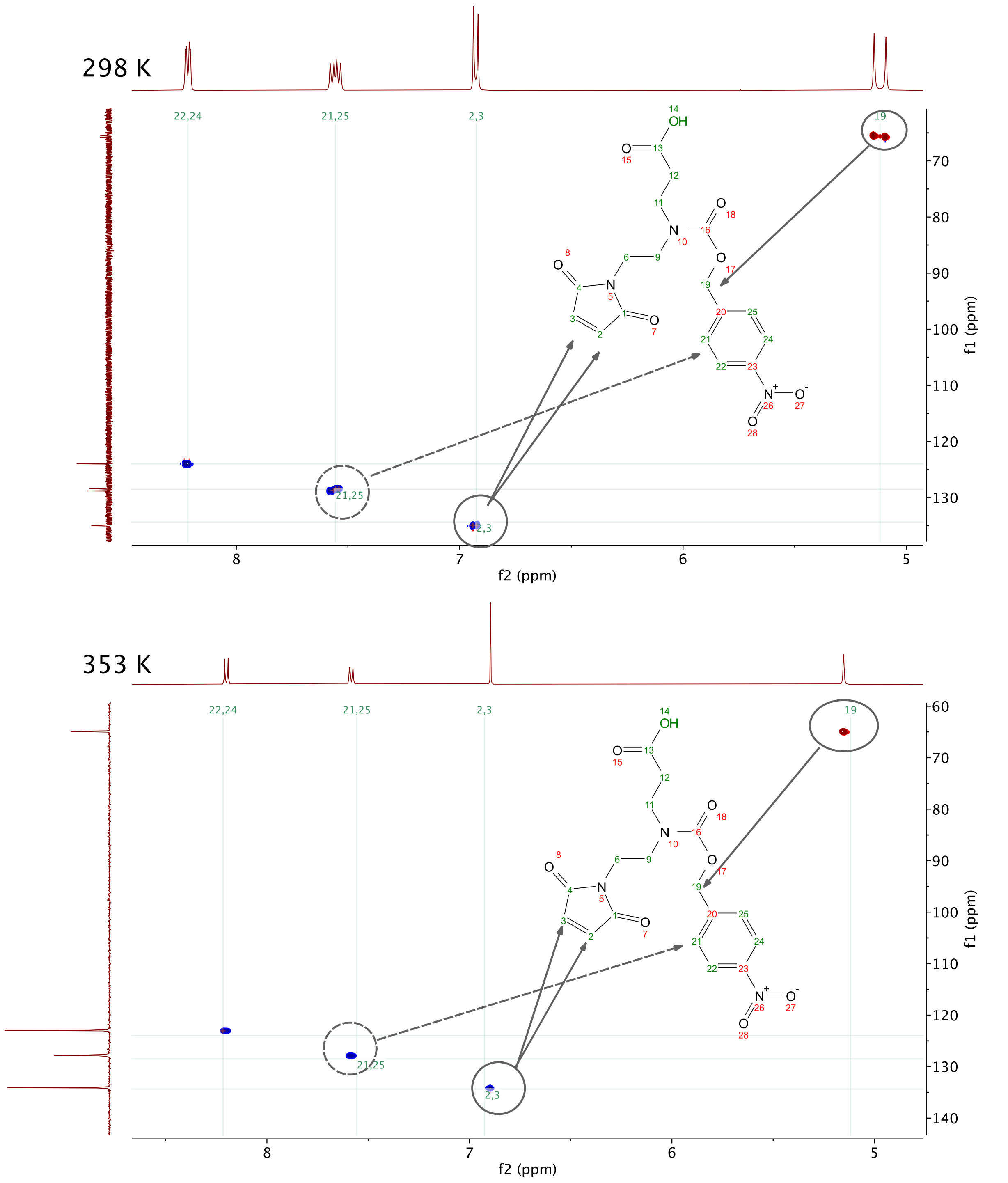

Figure S85: HSQC NMR spectra of maleimide **7** at 298 and 353 K highlighting key signals that show the conversion from two to one rotamers as temperature is increased.

References

(S1) Schindelin, J.; Arganda-Carreras, I.; Frise, E.; Kaynig, V.; Longair, M.; Pietzsch, T.; Preibisch, S.; Rueden, C.; Saalfeld, S.; Schmid, B.; Tinevez, J.-Y.; White, D. J.; Hartenstein, V.; Eliceiri, K.; Tomancak, P.; Cardona, A. Fiji: an open-source platform for biological-image analysis. *Nat. Methods* **2012**, *9*, 676–682.

(S2) Li, K.; Dong, W.; Qiu, L.; Liu, Q.; Lv, G.; Peng, Y.; Xie, M.; Lin, J. A new GSH-responsive prodrug of 5-aminolevulinic acid for photodiagnosis and photodynamic therapy of tumors. *Eur. J. Med. Chem.* **2019**, *181*, 111582.

(S3) Walker, J. M.; Ditor, S. E. E. In *Peptide Synthesis and Applications*; Howl, J., Ed.; Humana Press Inc., 2005.

(S4) Vasco, A. V.; Ricardo, M. G.; Rivera, D. G.; Wessjohann, L. A. *Methods Mol Biol*, 2021st ed.; 2022; Vol. 2371; pp 143–157.

(S5) Deng, Z.; Hu, J.; Liu, S. Disulfide-Based Self-Immolative Linkers and Functional Bioconjugates for Biological Applications. *Macromol Rapid Commun* **2020**, *41*, 1900531.

(S6) Kularatne, S. A.; Venkatesh, C.; Santhapuram, H.-K. R.; Wang, K.; Vaitilingam, B.; Henne, W. A.; Low, P. S. Synthesis and Biological Analysis of Prostate-Specific Membrane Antigen-Targeted Anticancer Prodrugs. *J. Med. Chem.* **2010**, *53*, 7767–7777.

(S7) Jain, A. K.; Gund, M. G.; Desai, D. C.; Borhade, N.; Senthilkumar, S. P.; Dhiman, M.; Mangu, N. K.; Mali, S. V.; Dubash, N. P.; Halder, S.; Satyam, A. Mutual prodrugs containing bio-cleavable and drug releasable disulfide linkers. *Bioorg. Chem.* **2013**, *49*, 40–48.

(S8) Kariuki, C. K.; Magez, S. Improving the yield of recalcitrant Nanobodies by simple modifications to the standard protocol. *Protein Expr. Purif.* **2021**, *185*, 105906.

(S9) Geoghegan, K. F.; Dixon, H. B. F.; Rosner, P. J.; Hoth, L. R.; Lanzetti, A. J.; Borzilleri, K. A.; Marr, E. S.; Pezzullo, L. H.; Martin, L. B.; LeMotte, P. K.; McColl, A. S.; Kamath, A. V.; Stroh, J. G. Spontaneous -N-6-Phosphogluconoylation of a His Tag inEscherichia coli:The Cause of Extra Mass of 258 or 178 Da in Fusion Proteins. *Anal. Biochem.* **1999**, *267*, 169–184.

(S10) Oliveira, B. L.; Guo, Z.; Bernardes, G. J. L. Inverse electron demand Diels–Alder reactions in chemical biology. *Chem. Soc. Rev.* **2017**, *46*, 4895–4950.

(S11) Janson, N.; Krüger, T.; Karsten, L.; Boschanski, M.; Dierks, T.; Müller, K. M.; Sewald, N. Bifunctional Reagents for Formylglycine Conjugation: Pitfalls and Breakthroughs. *ChemBioChem* **2020**, *21*, 3580–3593.
